# Chemistry-based molecular signature underlying the atypia of clozapine

**DOI:** 10.1038/tp.2017.6

**Published:** 2017-02-21

**Authors:** T Cardozo, E Shmelkov, K Felsovalyi, J Swetnam, T Butler, D Malaspina, S V Shmelkov

**Affiliations:** 1 Department of Biochemistry and Molecular Pharmacology, NYU School of Medicine, New York, NY, USA; 2 GeneCentrix Inc., New York, NY, USA; 3 Google Inc., Mountain View, CA, USA; 4 Department of Neurology, NYU School of Medicine, New York, NY, USA; 5 Department of Psychiatry, NYU School of Medicine, New York, NY, USA; 6 Department of Neuroscience and Physiology, NYU School of Medicine, New York, NY, USA

## Abstract

The central nervous system is functionally organized as a dynamic network of interacting neural circuits that underlies observable behaviors. At higher resolution, these behaviors, or phenotypes, are defined by the activity of a specific set of biomolecules within those circuits. Identification of molecules that govern psychiatric phenotypes is a major challenge. The only organic molecular entities objectively associated with psychiatric phenotypes in humans are drugs that induce psychiatric phenotypes and drugs used for treatment of specific psychiatric conditions. Here, we identified candidate biomolecules contributing to the organic basis for psychosis by deriving an *in vivo* biomolecule-tissue signature for the atypical pharmacologic action of the antipsychotic drug clozapine. Our novel *in silico* approach identifies the ensemble of potential drug targets based on the drug's chemical structure and the region-specific gene expression profile of each target in the central nervous system. We subtracted the signature of the action of clozapine from that of a typical antipsychotic, chlorpromazine. Our results implicate dopamine D4 receptors in the pineal gland and muscarinic acetylcholine M1 (CHRM1) and M3 (CHRM3) receptors in the prefrontal cortex (PFC) as significant and unique to clozapine, whereas serotonin receptors 5-HT_2A_ in the PFC and 5-HT_2C_ in the caudate nucleus were common significant sites of action for both drugs. Our results suggest that D4 and CHRM1 receptor activity in specific tissues may represent underappreciated drug targets to advance the pharmacologic treatment of schizophrenia. These findings may enhance our understanding of the organic basis of psychiatric disorders and help developing effective therapies.

## Introduction

Psychiatry has a diagnostic and classification system that is, in general, not based on etiology, pathophysiology, epidemiology or genetics, but rather on a constellation of human behavioral signs and symptoms.^1, 2^ Moreover, psychiatric diseases are not easily studied *in vitro* or in animal models, perhaps because many of them are, arguably, uniquely human. Thus, understanding of the molecular mechanisms of psychiatric disorders remains relatively limited despite many years of research, and, concomitantly, the record of discovery of new classes of drugs in psychiatry has been historically quite poor.^3^

The majority of drugs that are used to treat psychiatric disorders were discovered by serendipity (for example, observation of phenotypic effects of ingestion of the drug). However, these drugs are used successfully and selectively to treat distinct psychiatric conditions. Thus, defined, organic molecular entities exist to which phenotypes in psychiatry may be matched, namely drugs that induce psychiatric phenotypes (for example, lysergic acid diethylamide (LSD)) and drugs that are used for treatment of specific psychiatric conditions. Since drugs used to treat schizophrenia, for example, incontrovertibly have a symptomatic effect in affected individuals and little or no effect in unaffected individuals, the probability is high that the molecular physiologic basis of their *in vivo* effects at least partly overlaps with the organic basis of schizophrenia itself. As such, the drugs themselves could be used to identify significant clues as to the organic basis of psychiatric phenotypes. A good example of a precise, psychiatric phenotype is illustrated by the reproducible phenotype produced in patients by the antipsychotic drug clozapine (Clozaril), which differs reliably from phenotypes produced in patients by other antipsychotic drugs.

Historically, antipsychotic drugs have been grouped observationally according to both their pattern of clinical activity and their suspected mechanism of action. The original antipsychotic drugs, such as chlorpromazine (Thorazine), are considered ‘typical', exhibiting reliable antipsychotic actions accompanied by extrapyramidal and endocrine side effects that are ascribed to their dopamine D2 receptor antagonism.^4^ The second generation, ‘atypical' antipsychotic drug clozapine is often effective in patients who have been refractory to typical antipsychotics. Clozapine is associated with fewer extrapyramidal and possibly fewer cognitive side effects.^5^ Clozapine has lower affinity for D2 receptors and, at therapeutic concentrations, occupies only 40–60% of D2 receptors, whereas typical antipsychotics occupy >80%, suggesting that inhibition of D2 receptors only partly explains clozapine's mechanism of action.^6^ 5-HT_2A_ antagonism is also implicated, but the precise basis of Clozapine's atypicality (or ‘atypia') remains unknown and is likely polypharmacologic.^6, 7^ Although it appears to be a superior drug for psychosis, clozapine is not the first-line therapy because it idiosyncratically causes agranulocytosis, which can be fatal without supportive medical care,^8^ and has other significant side effects, including orthostatic hypotension and debilitating hypersalivation.^9^

Although numerous affinities of antipsychotic drugs for various individual receptors have been recorded and reported in the literature, these data have not sufficiently illuminated the mechanism of action. *In vivo*, polypharmacologic views of the actions of these drugs have not been extensively proposed. Such views would consist of weighted ensembles of all the receptors expressed by the human genome and affected by specific drugs,^10, 11, 12, 13, 14, 15, 16, 17, 18^ further stratified by the differential anatomic expression of these receptors.

We recently proposed such a technology to describe drug action, wherein a target-tissue (historeceptomic)^19^ profile provides a complex, *in vivo*, molecular signature of drug action. We sought to generate such a signature for clozapine that might illuminate its unique (atypical) actions. Notably, the input data for our approach were restricted to highly validated bioactivities of drugs against a comprehensive collection of human receptors combined with reproducible gene expression levels of those receptors in a variety of human tissues. As such, this approach may yield previously obscure organic bases for psychiatric phenotypes that are closer to the clinical phenomena than any other investigative method.

## Materials and methods

### Drug bioactivity data

The data on *in vitro* binding affinities of a drug to a target protein were downloaded from ChEMBL (https://www.ebi.ac.uk/chembl/, accessed on 2 May 2013) and filtered according to the following protocol.^20^

1. All records with the ‘STANDARD_TYPE' other than ‘Ki' were excluded;
2. All records with the ‘RELATION' other than ‘=' were excluded;
3. All records with the ‘STANDARD_UNITS' other than ‘nM' or ‘μM' were excluded;
4. All records with the ‘TARGET_TYPE' other than ‘single protein' were excluded;
5. All records with ‘ACTIVITY_COMMENTS' equal to ‘inactive' or ‘inconclusive' were excluded.

For each protein target in the filtered data set only a single smallest affinity value was then retained. If multiple human bioactivity records were available for a given protein target, the lowest human affinity value was used; if no human data were available, the lowest affinity from other mammals (for example, rat, mouse and so on) was retained. Note that even though a rule of thumb expectation is that a drug with an affinity to a given receptor higher than 1–10 μM would likely be inactive, no strict universal affinity cutoff value has ever been reported in the literature. Accordingly, no affinity cutoff value was imposed in the current analysis.

### Tissue-specific gene expression data

Gene expression data characterizing the expression levels of genes that encode the protein targets in different tissues were obtained through the BioGPS web tool^21, 22^ (http://biogps.org/, accessed on 7 May 2013). Specifically, human gene expression data from the data set ‘GeneAtlas U133A, gcrma' were used in the current study.^23^ For the muscarinic acetylcholine receptor M1 (CHRM1) no human gene expression data were available; the rat data were used instead. If for a given gene the data from multiple probes were available, the median of those values was used. As the goal of the current study was to obtain target-tissue fingerprints of clozapine and chlorpromazine in non-diseased human tissues, the data related to cell lines and diseased tissues (cancer) assessed in the ‘GeneAtlas U133A, gcrma' data set were excluded from the analysis.

### Target-tissue scores

The combined tissue-molecular scoring of drugs was performed as follows. First, affinity value of each drug for each receptor was projected into the logarithmic scale. Second, levels of the expression of each target protein in different tissues were normalized with regard to the level of expression of that same target protein in all assayed tissues (that is, *Z*-score). Finally, the combined score was calculated according to the following formula:


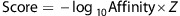


Thus, the target-tissue fingerprint of a drug could be described by those tissue-specific drug–target interactions that have significantly higher target-tissue scores than background.

### Statistical analysis

Multiple approaches to novelty detection have been proposed in the literature.^24^ In the current study the following statistical model was used. First, the distribution of all scores was assumed to be approximately normal with outliers, where outliers represent the true signal (that is, tissue-specific drug–target interactions responsible for the physiological phenotype), and the rest of the data are a normally distributed background (that is, interactions that are not physiologically significant). Then, the generalized extreme Studentized deviate test^25^ was applied to statistically detect those outliers (*α*=0.0001). Finally, in order to reduce the number of false-positives and obtain very specific pharmacological profiles of the studied drugs, only the interactions in the tissues of the central nervous system were tested.

### Principal component analysis

We assembled a list of 37 antipsychotic drugs (Supplementary Table S1). Eleven of these drugs did not have ChEMBL bioactivity data fulfilling the criteria described above. We then performed target-tissue analysis (with human targets only) on the remaining 26 drugs, and 25 of them had outlying target-tissue scores (outliers). As a reference, we also analyzed LSD, a drug of abuse that induces psychosis, with the same method.

The principal component analysis (PCA) was done using the resulting outlier target-tissue pairs for the 25 antipsychotic drugs and LSD. For each drug, we assembled an array of scores for all derived target-tissue pairs: for each pair, either the outlier target-tissue score if it was an outlier for the given drug; or zero if the target-tissue pair was not an outlier for the drug. These arrays of target-tissue scores were then analyzed by PCA. The calculation and visualization were done with the R data analysis software package (http://www.r-project.org/).

## Results

**Figure 1:**
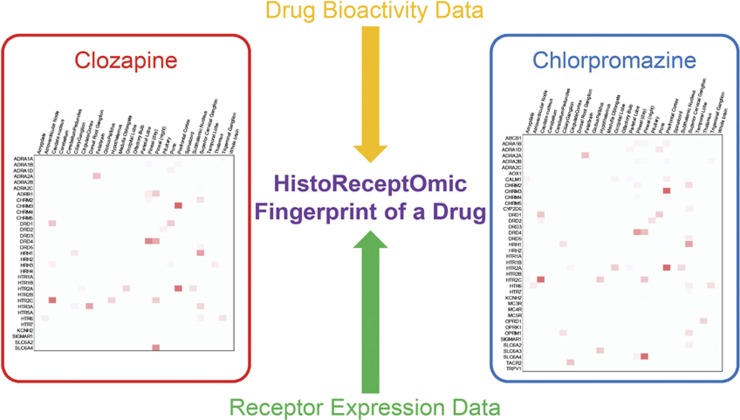
Integration of drug bioactivity and receptor expression data into target-tissue fingerprints. Experimental binding affinities of the clozapine and chlorpromazine to 34 and 41 target proteins, respectively, were integrated with the gene expression data for those protein targets in 77 normal human tissues in order to generate the target-tissue signatures for clozapine (left panel) and chlorpromazine (right panel).

We analyzed the comprehensive data set of potential human receptors of clozapine and chlorpromazine and obtained reliable affinity (*K_i_*) data for all of the proteins targeted by each of these drugs (Figure 1). Numerous measurements of both drugs against the anecdotally associated D2 and 5-HT_2A_ receptors from earlier approaches are represented within the data set, and their affinity profiles differ for each drug. We combined the affinity and gene expression data for each protein target in 77 normal human tissues to obtain target-tissue scores (see Materials and methods) for both drugs against all receptors (Figure 1, Supplementary Table S2 and Supplementary Figure S1). An outlier detection statistical model (see Materials and methods and Supplementary Figure S1) was used to identify statistically significant scores, the full set of which represents the target-tissue fingerprint, or signature, for the polypharmacologic, multi-tissue mechanism of action of each drug.

**Figure 2:**
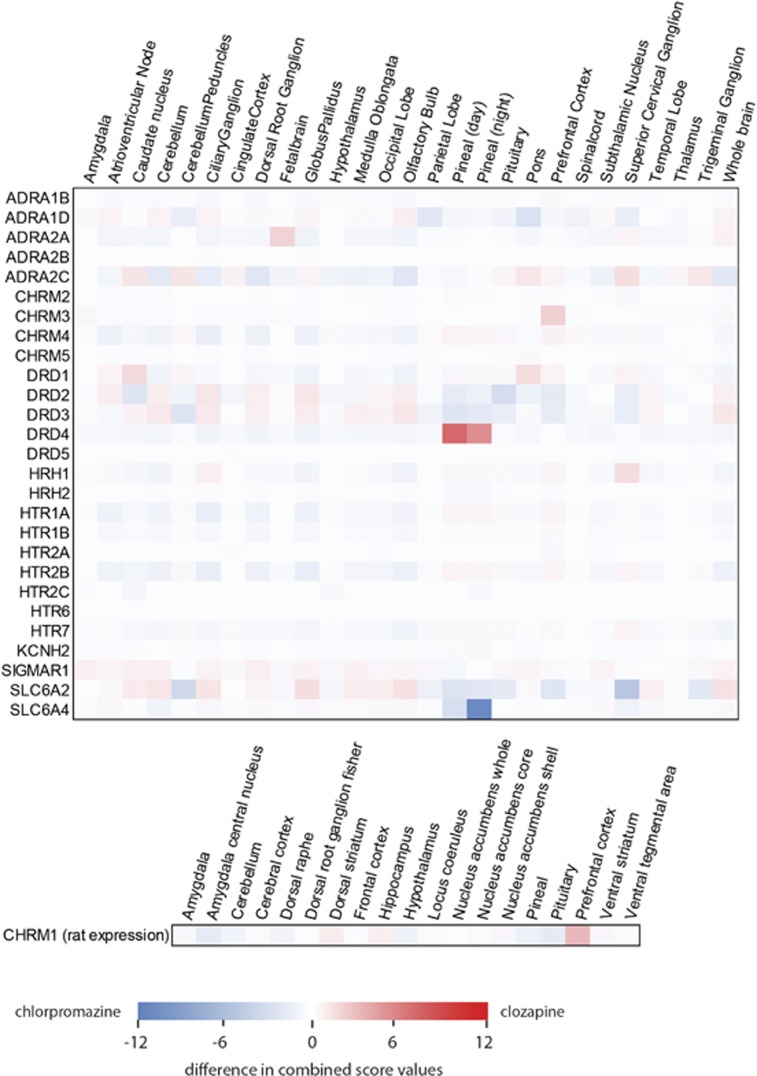
Difference between the target-tissue signatures of clozapine and chlorpromazine. The heatmap shows the difference in the combined score between clozapine and chlorpromazine from blue for negative to red for positive values (close to zero values are shown in white). CHRM1 scores are presented in a separate heatmap, as human expression data for that receptor were not available, and rat expression used in the study was measured for a different set of tissues.

**Figure 3:**
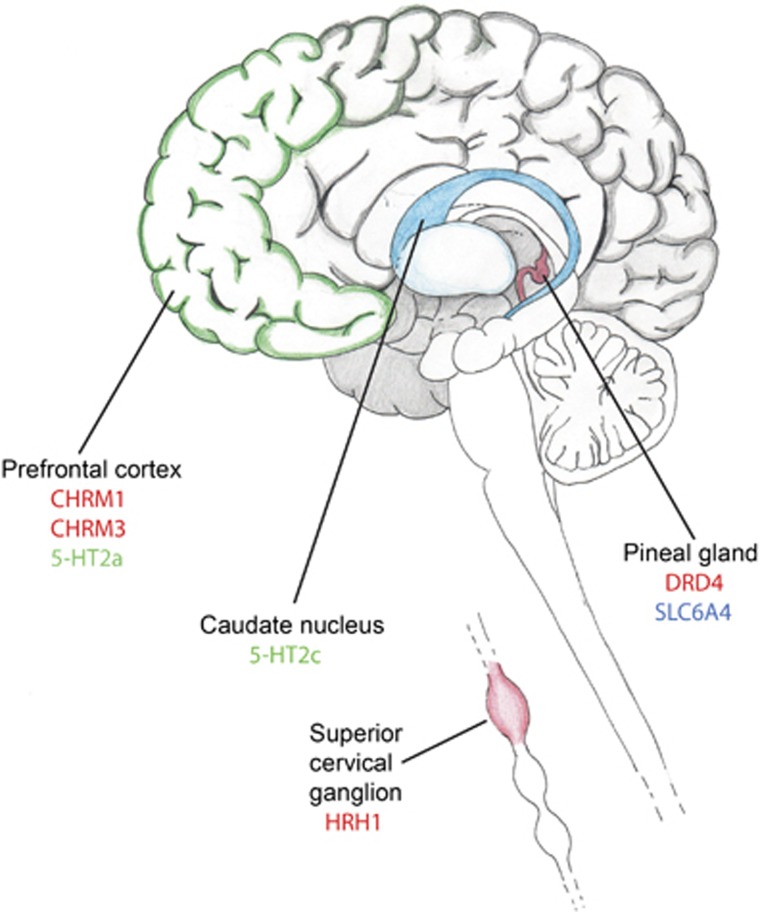
Proposed targets for the atypical action of clozapine. Protein targets that are responsible for atypical action of clozapine are shown in red. Protein targets that are common for clozapine and chlorpromazine are shown in green and targets specific for the action of chlorpromazine are shown in blue.

The signature for the ‘atypia' of clozapine was visualized by subtracting the chlorpromazine target-tissue signature from that of clozapine (Figure 2). The common antipsychotic effect of clozapine and chlorpromazine is represented by the overlap between these two signatures, and was determined to be serotonin 5-HT_2A_ and 5-HT_2C_ receptors in prefrontal cortex (PFC) and caudate nucleus, respectively (Figure 3 and Table 1). The notable targets that are specific to clozapine are the dopamine D4 receptor in the pineal gland, the muscarinic acetylcholine receptors M1 and M3 in PFC and the histamine H1 receptor in superior cervical ganglion (SCG; Figure 3 and Table 1). The highest scoring D2 tissue pair for either drug was for the pituitary gland.

**Figure 4:**
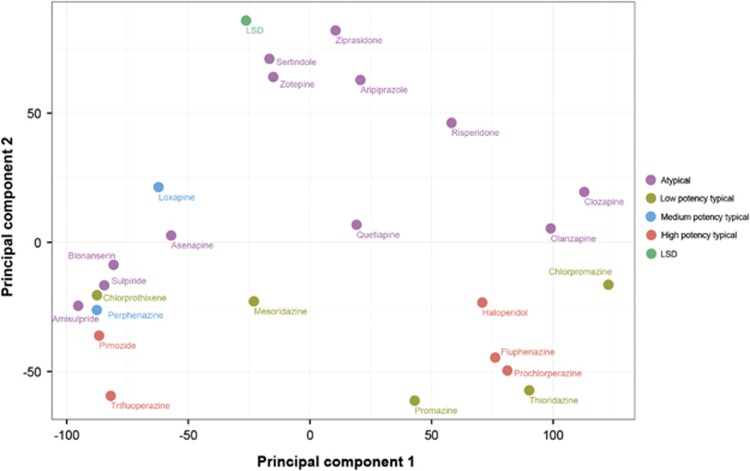
Principal component analysis (PCA) of the target-tissue signatures of antipsychotic drugs. For each analyzed drug, an array of the significant target-tissue scores was made. These drug arrays (that is, signatures) were analyzed with PCA and rendered to visualize clusters of related drugs, using the first two principal components (37% and 14% explained variance, respectively). Each drug is labeled and color-coded according to its category (atypical, high potency typical, medium potency typical or low potency typical). Lysergic acid diethylamide (LSD), a drug of abuse that induces psychosis, was included in the analysis as a reference point.

Target-tissue signatures for all common typical and atypical antipsychotic drugs as well as LSD, a drug that induces psychosis, were generated. These signatures were transformed into vectors and visualized by PCA (Figure 4 and Supplementary Table S1). Newer atypical antipsychotics derived from clozapine cluster with LSD, whereas newer typical antipsychotics occupy a different region of the target-tissue space.

## Discussion

Prior polypharmacologic approaches to drug action have successfully predicted new physiologically relevant targets for known psychiatric drugs as well as side effects of drugs.^10, 11, 12, 13, 14, 15, 16, 17, 18, 26, 27^ These studies demonstrate that polypharmacologic understanding of drug action (that is, based on the full set of relevant targets) is superior to the single-target view. However, drug action is incontrovertibly the product of both direct chemical activity against targets and the expression pattern of those targets in specific tissues in the human body. Accordingly, the important targets of a drug are most likely those that are expressed in disease-relevant tissues.^28^ This combined target-tissue view of drug action was previously pioneered by us, and has been applied to a specific question in this report.^19^ It is important to note that our approach operates in target-tissue space, and therefore any result should be viewed *exclusively* in these two dimensions (that is, drugs acting on a certain receptor in the particular tissue), in contrast to the common view in the literature in which target affinities and tissue expression of targets are, almost universally, independently discussed.

The target-tissue signature identified herein for clozapine reinforces one of the leading theories about the action of antipsychotics. The serotonin 5-HT_2A_ receptor acting in the PFC was classified by our approach as a common component of the antipsychotic effect of both clozapine and chlorpromazine. Notably, 5-HT_2A_ is the target receptor of LSD, which produces symptoms in normal individuals and animal models similar to the psychosis symptoms in schizophrenia.^29, 30^ Furthermore, neuroimaging data localize schizophrenia-specific brain activity to the PFC.^31^

The signature also provides some insight into side effects of clozapine. Clozapine's atypical effect mapped to the histamine receptor H1 in the SCG. This could explain the drug's propensity to cause severe orthostatic hypotension (mediated by SCG), which is one reason clozapine must be started at a very low initial dose. The action in the SCG could also relate to hypersalivation—a debilitating side-effect of clozapine—as SCG fibers innervate sublingual salivary glands. Interestingly, the most recent Cochrane review of the clinical evidence for hypersalivation treatment found that the only two effective drugs for clozapine-induced hypersalivation were astemizole and diphenhydramine (Benadryl).^32^ Both are H1 antagonists, although, remarkably, the Cochrane review did not identify them as such. Thus, our purely molecular method is consistent with a specific, non-molecular, clinical observation.

Target-tissue signatures provide a means to generate a novel, comparative visualization of the space of this class of drugs (Figure 4); however, the utility of such visualization has not yet been established. Nevertheless, our approach produces very specific, unprecedented *in vivo* signatures for complex drug actions that, in the case of clozapine, correlate closely with disparate observations related to the drug's use in human subjects. As a prototype, this new approach could be used to investigate many drugs and phenotypes, but has several limitations. First, the recorded bioactivities do not cover the space of all possible interactions between these drugs and all drug targets expressed by the human genome; therefore, many significant target-tissue pairs may be omitted. Second, we used gene expression data from non-diseased individuals. However, gene expression in some of these tissues may differ in afflicted individuals, because of the disease as well as its treatment; thus, data from individuals with schizophrenia may improve the signature. Third, gene expression levels do not always reflect the expression levels of the corresponding proteins, which are the true targets of the drugs, and, as such, our follow-up studies intend to include proteomics data. Fourth, for one target out of hundreds, M1, rat gene expression data were used, because human expression data were not available. Ironically, M1 emerged as an outlier; therefore, the results for the M1 receptor would be best considered with caution and may need additional statistical or experimental verification in future studies. Finally, the approach is based on differential gene expression across tissues, which is not sensitive to ubiquitously expression targets.

The aforementioned limitations are opportunities for future improvements of this first-of-class reported target-tissue concept; however, these limitations are expected to be reflective of a method with high specificity, but suboptimal sensitivity. This expectation is best illustrated by considering just the pairwise comparison between the outlier score for 5-HT_2A_ in the PFC and the insignificant score for D2 (at *α*=0.0001) in its highest scoring tissue: the former combined score of affinity and gene expression is much higher than D2's in pituitary gland and is significant compared to the population of scores, whereas the latter is not at the chosen significance level. In order to argue that there is a problem in this *pairwise* comparison, one would have to argue that either the affinities recorded in ChEMBL for D2 and 5-HT_2A_ receptors are incorrect, which is unsupported since they have been reproduced many times in the literature, or that the expression pattern of D2 and 5-HT_2A_ in tissues/the PFC recorded in BioGPS are misleading, which goes against many publications. If this pairwise comparison is unassailable, then the whole network of comparisons, which were done in exactly the same way, is also unassailable.

The *in vivo* molecular basis of clozapine has never previously been viewed in target-tissue space; thus, it is not surprising that several results emerged that are either underappreciated in the field, or run counter to prevailing theories. The most notable of these is that D2 receptors, which are widely cited in the literature as being involved in both psychosis and the action of these drugs, are not the strongest contributors to the action of either drug in differential target-tissue space, although D2 receptors appear on the list at more sensitive *P*-values. D2 has strong evidence linking it to schizophrenia, and the antipsychotics have incontrovertibly, high affinity for D2 receptors. Similarly, N-methyl-D-aspartate (NMDA) receptors are not top-ranked in our lists, and NMDA receptors also has strong evidence linking it to schizophrenia. The most likely explanation for this discrepancy is that D2 and NMDA receptors are ubiquitously expressed, to which our method is insensitive. The outliers we have identified are likely to be more (relatively) physiologically important than D2 activity in any brain region. Importantly, direct and indirect D2 and NMDA receptors activity may still be absolutely physiologically important in the drug and/or the disease. On the other hand, it is also possible that the activity of D2 and NMDA receptors *in vivo* in both schizophrenia and in the action of these drugs are indirect effects or are overstated by the field. Given the extremely poor track record of the discovery of new classes of antipsychotics,^3^ which has been strongly driven by D2 and NMDA theories, it is plausible that the importance of D2 and NMDA receptors, at least, have historically been inflated in the field and has confounded drug discovery.

Thus, previously unsuspected or underappreciated *in vivo* hypotheses for the action of these psychiatric drugs have been identified in this report. First, the activity of the serotonin 5-HT_2C_ receptor in the caudate is associated with the bioactivity of both drugs. This association has not previously been widely proposed as a primary component of the antipsychotic action of these drugs. The interaction with the caudate is interesting because the caudate is both involved in the pathogenesis of schizophrenia and associates with motor side effects.^33^ Second, the basis for clozapine's effects on mood has not previously been deciphered. The signature for clozapine's atypia strongly implicates D4 receptors in the pineal gland, which produces the hormone melatonin and thus strongly influences mood via circadian rhythms. Indeed, melatonin has previously been studied for its mood-stabilizing (antidepressive) effects and the first melatonergic drug for the treatment of depression has been approved for human use.^34^ This suggests that the combination of typical antipsychotics with melatonergic agonists may capture some of the beneficial, antidepressive, atypical antipsychotic effects of clozapine, whereas avoiding its limiting side effects.^8^ Finally, the signature for the atypia of clozapine includes CHRM1 and CHRM3 in PFC. These receptors have not previously been singled out as targets for antipsychotic treatment; however, there may be no effective way to test this finding, which by our method is a human *in vivo* hypothesis, without clinical trials. Notably, M1 agonists were found to be one of the few pharmaceutics ever to result in improved cognitive symptoms in schizophrenia patients.^35^

Our approach has broad implications for therapy in psychiatry. Focusing on a specific target-tissue pair, like the underappreciated ones that we have identified in this report, requires both a drug specific for the target and selective targeting of the drug to the tissue to take full advantage of our finding. This is an unprecedented concept in translational science outside of cancer therapies,^36^ but is conceptually similar to interventional neuropsychiatry and stereotactic neuro–radio–surgery approaches, which are precisely tissue-specific.

## Figures and Tables

**Table 1:** Difference between pharmareceptomics fingerprints of clozapine and chlorpromazine

*Gene symbol*	*Gene name*	*Tissue*	*Affinity difference*	*Score difference*
*SLC6A4*	Serotonin transporter	Pineal (night)	−0.14	−8.78
*HTR2C*	Serotonin 2c (5-HT_2C_) receptor	Caudate nucleus	−0.06	−0.43
*HTR2A*	Serotonin 2a (5-HT_2A_) receptor	Prefrontal cortex	−0.04	−0.26
*HRH1*	Histamine H1 receptor	Superior cervical ganglion	0.54	2.55
ADRA2A*	Alpha-2a adrenergic receptor	Fetal brain	0.74	3.30
*CHRM3*	Muscarinic acetylcholine receptor M3	Prefrontal cortex	0.41	3.50
*CHRM1*	Muscarinic acetylcholine receptor M1	Prefrontal cortex	1.31	5.38
*DRD4*	Dopamine D4 receptor	Pineal (night)	1.73	8.80
*DRD4*	Dopamine D4 receptor	Pineal (day)	1.73	11.77
HTR3A*	Serotonin 3a (5-HT_3A_) receptor	Dorsal root ganglion	n/a	n/a

Abbreviation: n/a, not applicable. The significant (*α*=0.0001) drug:receptor interactions composing the pharmareceptomics fingerprints of clozapine and chlorpromazine are shown. The interactions labeled with * are exclusive to the clozapine profile under the selected *α*-level (note that no affinity data of chlorpromazine to Serotonin 3a (5-HT_3A_) receptor are available). Values in 'Affinity difference' and 'Score difference' columns are calculated by subtracting the corresponding affinity or combined score values specific to chlorpromazine from those specific to clozapine.

## References

[bib1] Gould TD, Gottesman II. Psychiatric endophenotypes and the development of valid animal models. Genes Brain Behav 2006; 5: 113–119. 16507002 10.1111/j.1601-183X.2005.00186.x

[bib2] Agid Y, Buzsaki G, Diamond DM, Frackowiak R, Giedd J, Girault JA et al. How can drug discovery for psychiatric disorders be improved? Nat Rev Drug Discov 2007; 6: 189–201. 17330070 10.1038/nrd2217

[bib3] Marder SR, Roth B, Sullivan PF, Scolnick EM, Nestler EJ, Geyer MA et al. Advancing drug discovery for schizophrenia. Ann N Y Acad Sci 2011; 1236: 30–43. 22032400 10.1111/j.1749-6632.2011.06216.x PMC3787879

[bib4] Nord M, Farde L. Antipsychotic occupancy of dopamine receptors in schizophrenia. CNS Neurosci Ther 2011; 17: 97–103. 21143431 10.1111/j.1755-5949.2010.00222.x PMC6493889

[bib5] Meltzer HY. Treatment-resistant schizophrenia—the role of clozapine. Curr Med Res Opin 1997; 14: 1–20. 9524789 10.1185/03007999709113338

[bib6] Wenthur CJ, Lindsley CW. Classics in chemical neuroscience: clozapine. ACS Chem Neurosci 2013; 4: 1018–1025. 24047509 10.1021/cn400121z PMC3715841

[bib7] Miyamoto S, Duncan GE, Marx CE, Lieberman JA. Treatments for schizophrenia: a critical review of pharmacology and mechanisms of action of antipsychotic drugs. Mol Psychiatry 2005; 10: 79–104. 15289815 10.1038/sj.mp.4001556

[bib8] Wu C, Orozco C, Boyer J, Leglise M, Goodale J, Batalov S et al. BioGPS: an extensible and customizable portal for querying and organizing gene annotation resources. Genome Biol 2009; 10: R130. 19919682 10.1186/gb-2009-10-11-r130 PMC3091323

[bib9] Raja M, Raja S. Clozapine safety, 40 years later. Curr Drug Safety 2014; 9: 163–195. 10.2174/1574886309666140428115040 24809463

[bib10] Hao M, Wang Y, Bryant SH. Improved prediction of drug-target interactions using regularized least squares integrating with kernel fusion technique. Anal Chim Acta 2016; 909: 41–50. 26851083 10.1016/j.aca.2016.01.014 PMC4744621

[bib11] Amelio I, Landré V, Knight RA, Lisitsa A, Melino G, Antonov AV. Polypharmacology of small molecules targeting the ubiquitin–proteasome and ubiquitin-like systems. Oncotarget 2015; 6: 9646–9656. 25991664 10.18632/oncotarget.3917 PMC4496386

[bib12] Zhao Z, Xie L, Xie L, Bourne PE. Delineation of polypharmacology across the human structural kinome using a functional site interaction fingerprint approach. J Med Chem 2016; 59: 4326–4341. 26929980 10.1021/acs.jmedchem.5b02041 PMC4865454

[bib13] Wang X, Pan C, Gong J, Liu X, Li H. Enhancing the enrichment of pharmacophore-based target prediction for the polypharmacological profiles of drugs. J Chem Inform Model 2016; 56: 1175–1183. 10.1021/acs.jcim.5b00690 27187084

[bib14] Wang Y, Cornett A, King FJ, Mao Y, Nigsch F, Paris CG et al. Evidence-based and quantitative prioritization of tool compounds in phenotypic drug discovery. Cell Chem Biol 2016; 23: 862–874. 27427232 10.1016/j.chembiol.2016.05.016

[bib15] Lavecchia A, Cerchia C. In silico methods to address polypharmacology: current status, applications and future perspectives. Drug Discov Today 2016; 21: 288–298. 26743596 10.1016/j.drudis.2015.12.007

[bib16] Li YH, Wang PP, Li XX, Yu CY, Yang H, Zhou J et al. The human kinome targeted by FDA approved multi-target drugs and combination products: a comparative study from the drug-target interaction network perspective. PLoS ONE 2016; 11: e0165737. 27828998 10.1371/journal.pone.0165737 PMC5102354

[bib17] Kibble M, Saarinen N, Tang J, Wennerberg K, Makela S, Aittokallio T. Network pharmacology applications to map the unexplored target space and therapeutic potential of natural products. Nat Prod Rep 2015; 32: 1249–1266. 26030402 10.1039/c5np00005j

[bib18] Gilberg E, Jasial S, Stumpfe D, Dimova D, Bajorath J. Highly promiscuous small molecules from biological screening assays include many pan-assay interference compounds but also candidates for polypharmacology. J Med Chem 2016; 59: 10285–10290. 27809519 10.1021/acs.jmedchem.6b01314

[bib19] Shmelkov E, Grigoryan A, Swetnam J, Xin J, Tivon D, Shmelkov SV et al. Historeceptomic fingerprints for drug-like compounds. Front Physiol 2015; 6: 371. 26733872 10.3389/fphys.2015.00371 PMC4683199

[bib20] Gaulton A, Bellis LJ, Bento AP, Chambers J, Davies M, Hersey A et al. ChEMBL: a large-scale bioactivity database for drug discovery. Nucleic Acids Res 2012; 40: D1100–D1107. 21948594 10.1093/nar/gkr777 PMC3245175

[bib21] Wu C, Orozco C, Boyer J, Leglise M, Goodale J, Batalov S et al. BioGPS: an extensible and customizable portal for querying and organizing gene annotation resources. Genome Biol 2009; 10: R130. 19919682 10.1186/gb-2009-10-11-r130 PMC3091323

[bib22] Wu C, Macleod I, Su AI. BioGPS and MyGene.info: organizing online, gene-centric information. Nucleic Acids Res 2013; 41: D561–D565. 23175613 10.1093/nar/gks1114 PMC3531157

[bib23] Su AI, Wiltshire T, Batalov S, Lapp H, Ching KA, Block D et al. A gene atlas of the mouse and human protein-encoding transcriptomes. Proc Natl Acad Sci USA 2004; 101: 6062–6067. 15075390 10.1073/pnas.0400782101 PMC395923

[bib24] Marsland S. Novelty detection in learning systems. Neural Comput Surv 2002; 3: 1–39.

[bib25] Rosner B. Percentage points for a generalized ESD many outlier procedure. Technometrics 1983; 25: 165–172.

[bib26] Keiser MJ, Setola V, Irwin JJ, Laggner C, Abbas AI, Hufeisen SJ et al. Predicting new molecular targets for known drugs. Nature 2009; 462: 175–181. 19881490 10.1038/nature08506 PMC2784146

[bib27] Lounkine E, Keiser MJ, Whitebread S, Mikhailov D, Hamon J, Jenkins JL et al. Large-scale prediction and testing of drug activity on side-effect targets. Nature 2012; 486: 361–367. 22722194 10.1038/nature11159 PMC3383642

[bib28] Kumar V, Sanseau P, Simola DF, Hurle MR, Agarwal P. Systematic analysis of drug targets confirms expression in disease-relevant tissues. Sci Rep 2016; 6: 36205. 27824084 10.1038/srep36205 PMC5099936

[bib29] Gonzalez-Maeso J, Sealfon SC. Psychedelics and schizophrenia. Trends Neurosci 2009; 32: 225–232. 19269047 10.1016/j.tins.2008.12.005

[bib30] Moreno JL, Gonzalez-Maeso J. Preclinical models of antipsychotic drug action. Int J Neuropsychopharmacol 2013; 16: 2131–2144. 23745738 10.1017/S1461145713000606 PMC4836391

[bib31] Liemburg EJ, Knegtering H, Klein HC, Kortekaas R, Aleman A. Antipsychotic medication and prefrontal cortex activation: a review of neuroimaging findings. Eur Neuropsychopharmacol 2012; 22: 387–400. 22300864 10.1016/j.euroneuro.2011.12.008

[bib32] Syed R, Au K, Cahill C, Duggan L, He Y, Udu V et al. Pharmacological interventions for clozapine-induced hypersalivation. Cochrane Database Syst Rev 2008; CD005579. 18646130 10.1002/14651858.CD005579.pub2 PMC4160791

[bib33] Chakos MH, Lieberman JA, Alvir J, Bilder R, Ashtari M. Caudate nuclei volumes in schizophrenic patients treated with typical antipsychotics or clozapine. Lancet 1995; 345: 456–457. 10.1016/s0140-6736(95)90441-7 7853978

[bib34] de Bodinat C, Guardiola-Lemaitre B, Mocaer E, Renard P, Munoz C, Millan MJ. Agomelatine, the first melatonergic antidepressant: discovery, characterization and development. Nat Rev Drug Discov 2010; 9: 628–642. 20577266 10.1038/nrd3140

[bib35] Pomara N. Reduction in muscarinic M1-mediated hypercholinergic state and beneficial cognitive effects of muscarinic agonists in schizophrenia. Am J Psychiatry 2009; 166: 111, author reply 111-113. 19122018 10.1176/appi.ajp.2008.08091352

[bib36] Torchilin VP. Multifunctional, stimuli-sensitive nanoparticulate systems for drug delivery. Nat Rev Drug Discov 2014; 13: 813–827. 25287120 10.1038/nrd4333 PMC4489143

