# Supplementary material for: Chemistry-based molecular signature underlying the atypia of clozapine
Source: Transl Psychiatry. 2017 Feb 21;7(2):e1036–. doi: 10.1038/tp.2017.6 (PMC5438035; doi:10.1038/tp.2017.6)
Supplement: Supplementary Figure [file tp20176x1.pdf]

Figure S1

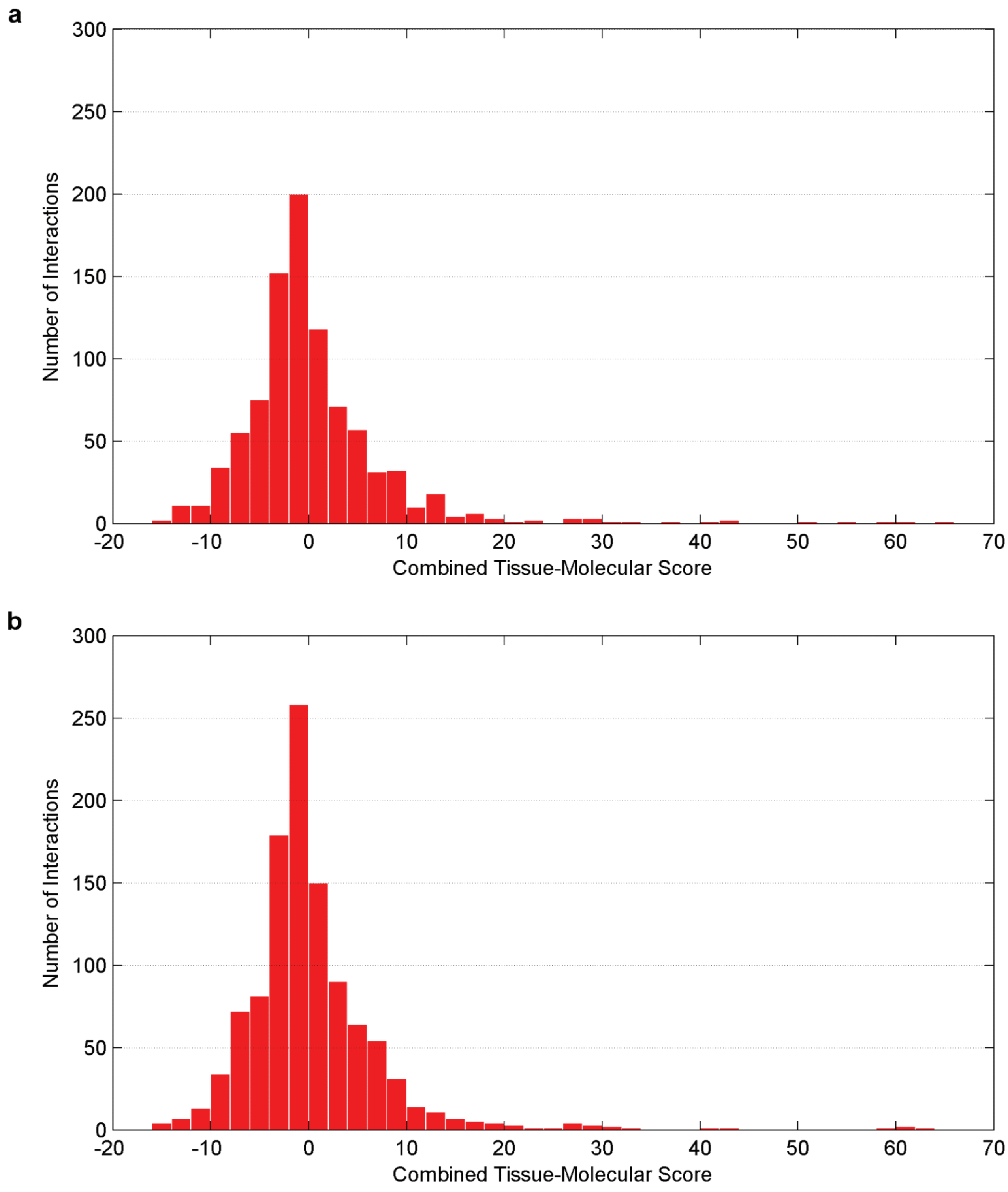

Figure S1. Distribution of all tissue-molecular drug-target interactions in the CNS by their combined score for clozapine (a) and for chlorpromazine (b). For each drug, the corresponding distribution of the scores is approximately normal with a number of outliers having substantially higher score values. The majority of the interactions are physiologically insignificant: e.g. when the binding affinity between a drug and the receptor is low, or when the receptor is not expressed in a given tissue. Such interactions are expected to be normally distributed. The tissue specific drug-target interactions responsible for the physiological phenotype, however, should have score values significantly higher than the background level, and thus are expected to be represented by the outliers.
