# Supplementary material for: Chemistry-based molecular signature underlying the atypia of clozapine
Source: Transl Psychiatry. 2017 Feb 21;7(2):e1036–. doi: 10.1038/tp.2017.6 (PMC5438035; doi:10.1038/tp.2017.6)
Supplement: Supplementary Table 1 [file tp20176x2.pdf]

S1 Table. Antipsychotic medications for which historeceptomic analysis was performed.

Category	Drug	ChEMBL identification	Number of targets	Number of outliers
Atypical	Amisulpride	CHEMBL243712	5	3
	Aripiprazole	CHEMBL1112	22	19
	Asenapine	CHEMBL1201756	3	8
	Blonanserin	CHEMBL178803	2	1
	Clozapine	CHEMBL42	34	27
	Iloperidone	CHEMBL14376	0*	N/A
	Lurasidone	CHEMBL1237021	0**	N/A
	Melperone	CHEMBL1531134	0*	N/A
	Olanzapine	CHEMBL715	27	25
	Paliperidone	CHEMBL1621	0*	N/A
	Quetiapine	CHEMBL716	22	15
	Risperidone	CHEMBL85	26	19
	Sertindole	CHEMBL12713	10	9
	Sulpiride	CHEMBL26	13	19
	Ziprasidone	CHEMBL708	13	15
	Zotepine	CHEMBL285802	9	11
Typical	Chlorpromazine	CHEMBL71	38	38
(Low potency)	Chlorprothixene	CHEMBL908	6	1
	Levomepromazine	CHEMBL1764	0*	N/A
	Mesoridazine	CHEMBL1088	0*	N/A
	Periciazine	CHEMBL251940	0*	N/A
	Promazine	CHEMBL564	21	13
	Thioridazine	CHEMBL479	26	22
Typical	Loxapine	CHEMBL831	5	5
(Medium potency)	Molindone	CHEMBL460	1	0***
	Perphenazine	CHEMBL567	2	7
	Thiothixene	CHEMBL1201	0*	N/A
Typical	Droperidol	CHEMBL1108	14	7
(High potency)	Flupentixol	CHEMBL42055, CHEMBL54661	0*	N/A
	Fluphenazine	CHEMBL726	31	27
	Haloperidol	CHEMBL54	26	32
	Pimozide	CHEMBL1423	3	1
	Prochlorperazine	CHEMBL728	23	22
	Thioproperazine	CHEMBL609109	0*	N/A
	Trifluoperazine	CHEMBL422	5	10
	Zuclopenthixol	CHEMBL87385	1	0***

List of antipsychotic medications used in the study. Each drug was searched in ChEMBL. If no bioactivity data were deposited; or if no bioactivity data matched the criteria for filtering, it is noted by stars. In some cases, at the final step of outlier detection, no historeceptomic scores were found to be outliers. * No bioactivity data matching criteria. ** No bioactivity data in ChEMBL. *** No outliers found.
