# Supplementary material for: Chemistry-based molecular signature underlying the atypia of clozapine
Source: Transl Psychiatry. 2017 Feb 21;7(2):e1036–. doi: 10.1038/tp.2017.6 (PMC5438035; doi:10.1038/tp.2017.6)
Supplement: Supplementary Table 2 [file tp20176x3.pdf]

S2 Table. Target-tissue analysis for clozapine and chlorpromazine

Gene Symbol	Gene Name	Affinity (-log10(Ki))		Tissue	Expression		Combined Score (-log10(Ki)*Z)	
		clozapine	chlorpromazine		Raw-numbers	Z-score	clozapine	chlorpromazine
ABCB1	P-glycoprotein 1	n/a	6.22184875	Adipocyte	5.65	-0.27	n/a	-1.68
ABCB1	P-glycoprotein 1	n/a	6.22184875	AdrenalCortex	360.35	7.45	n/a	46.38
ABCB1	P-glycoprotein 1	n/a	6.22184875	Adrenalgland	173.08	3.38	n/a	21.00
ABCB1	P-glycoprotein 1	n/a	6.22184875	Amygdala	10.23	-0.17	n/a	-1.06
ABCB1	P-glycoprotein 1	n/a	6.22184875	Appendix	7.03	-0.24	n/a	-1.49
ABCB1	P-glycoprotein 1	n/a	6.22184875	AtrioventricularNode	6.28	-0.26	n/a	-1.60
ABCB1	P-glycoprotein 1	n/a	6.22184875	BDCA4+_DentriticCells	6.65	-0.25	n/a	-1.54
ABCB1	P-glycoprotein 1	n/a	6.22184875	Bonemarrow	7.53	-0.23	n/a	-1.43
ABCB1	P-glycoprotein 1	n/a	6.22184875	BronchialEpithelialCells	6.28	-0.26	n/a	-1.60
ABCB1	P-glycoprotein 1	n/a	6.22184875	CardiacMyocytes	12.55	-0.12	n/a	-0.75
ABCB1	P-glycoprotein 1	n/a	6.22184875	Caudatenucleus	7.80	-0.22	n/a	-1.39
ABCB1	P-glycoprotein 1	n/a	6.22184875	CD105+_Endothelial	6.40	-0.25	n/a	-1.58
ABCB1	P-glycoprotein 1	n/a	6.22184875	CD14+_Monocytes	6.48	-0.25	n/a	-1.57
ABCB1	P-glycoprotein 1	n/a	6.22184875	CD19+_BCells(neg._sel.)	78.78	1.32	n/a	8.23
ABCB1	P-glycoprotein 1	n/a	6.22184875	CD33+_Myeloid	7.28	-0.23	n/a	-1.46
ABCB1	P-glycoprotein 1	n/a	6.22184875	CD34+	8.30	-0.21	n/a	-1.32
ABCB1	P-glycoprotein 1	n/a	6.22184875	CD4+_Tcells	27.45	0.20	n/a	1.27
ABCB1	P-glycoprotein 1	n/a	6.22184875	CD56+_NKCells	82.75	1.41	n/a	8.77
ABCB1	P-glycoprotein 1	n/a	6.22184875	CD71+_EarlyErythroid	6.20	-0.26	n/a	-1.61
ABCB1	P-glycoprotein 1	n/a	6.22184875	CD8+_Tcells	69.60	1.12	n/a	6.98
ABCB1	P-glycoprotein 1	n/a	6.22184875	Cerebellum	5.55	-0.27	n/a	-1.69
ABCB1	P-glycoprotein 1	n/a	6.22184875	CerebellumPeduncles	8.35	-0.21	n/a	-1.31
ABCB1	P-glycoprotein 1	n/a	6.22184875	CiliaryGanglion	6.65	-0.25	n/a	-1.54
ABCB1	P-glycoprotein 1	n/a	6.22184875	CingulateCortex	9.80	-0.18	n/a	-1.12
ABCB1	P-glycoprotein 1	n/a	6.22184875	Colon	8.13	-0.22	n/a	-1.35
ABCB1	P-glycoprotein 1	n/a	6.22184875	DorsalRootGanglion	7.13	-0.24	n/a	-1.48
ABCB1	P-glycoprotein 1	n/a	6.22184875	Fetalbrain	6.10	-0.26	n/a	-1.62
ABCB1	P-glycoprotein 1	n/a	6.22184875	Fetalliver	7.63	-0.23	n/a	-1.41
ABCB1	P-glycoprotein 1	n/a	6.22184875	Fetallung	5.18	-0.28	n/a	-1.74
ABCB1	P-glycoprotein 1	n/a	6.22184875	FetalThyroid	5.98	-0.26	n/a	-1.64
ABCB1	P-glycoprotein 1	n/a	6.22184875	GlobusPallidus	6.53	-0.25	n/a	-1.56
ABCB1	P-glycoprotein 1	n/a	6.22184875	Heart	11.95	-0.13	n/a	-0.83
ABCB1	P-glycoprotein 1	n/a	6.22184875	Hypothalamus	11.43	-0.14	n/a	-0.90
ABCB1	P-glycoprotein 1	n/a	6.22184875	Kidney	8.95	-0.20	n/a	-1.23
ABCB1	P-glycoprotein 1	n/a	6.22184875	Liver	44.98	0.59	n/a	3.65
ABCB1	P-glycoprotein 1	n/a	6.22184875	Lung	7.40	-0.23	n/a	-1.44
ABCB1	P-glycoprotein 1	n/a	6.22184875	Lymphnode	6.43	-0.25	n/a	-1.58
ABCB1	P-glycoprotein 1	n/a	6.22184875	MedullaOblongata	7.75	-0.22	n/a	-1.40
ABCB1	P-glycoprotein 1	n/a	6.22184875	OccipitalLobe	7.83	-0.22	n/a	-1.39
ABCB1	P-glycoprotein 1	n/a	6.22184875	OlfactoryBulb	11.08	-0.15	n/a	-0.95
ABCB1	P-glycoprotein 1	n/a	6.22184875	Ovary	4.53	-0.29	n/a	-1.83
ABCB1	P-glycoprotein 1	n/a	6.22184875	Pancreas	5.70	-0.27	n/a	-1.67
ABCB1	P-glycoprotein 1	n/a	6.22184875	PancreaticIslet	7.80	-0.22	n/a	-1.39
ABCB1	P-glycoprotein 1	n/a	6.22184875	ParietalLobe	7.78	-0.22	n/a	-1.39
ABCB1	P-glycoprotein 1	n/a	6.22184875	Pineal_day	9.02	-0.20	n/a	-1.22
ABCB1	P-glycoprotein 1	n/a	6.22184875	Pineal_night	10.25	-0.17	n/a	-1.06
ABCB1	P-glycoprotein 1	n/a	6.22184875	Pituitary	7.93	-0.22	n/a	-1.37
ABCB1	P-glycoprotein 1	n/a	6.22184875	Placenta	7.35	-0.23	n/a	-1.45
ABCB1	P-glycoprotein 1	n/a	6.22184875	Pons	6.00	-0.26	n/a	-1.63
ABCB1	P-glycoprotein 1	n/a	6.22184875	PrefrontalCortex	13.68	-0.10	n/a	-0.59
ABCB1	P-glycoprotein 1	n/a	6.22184875	Prostate	7.25	-0.24	n/a	-1.46
ABCB1	P-glycoprotein 1	n/a	6.22184875	Retina	8.90	-0.20	n/a	-1.24
ABCB1	P-glycoprotein 1	n/a	6.22184875	Salivarygland	5.75	-0.27	n/a	-1.67

S2 Table. Target-tissue analysis for clozapine and chlorpromazine

Gene Symbol	Gene Name	Affinity (-log10(Ki))		Tissue	Expression		Combined Score (-log10(Ki)*Z)	
		clozapine	chlorpromazine		Raw-numbers	Z-score	clozapine	chlorpromazine
ABCB1	P-glycoprotein 1	n/a	6.22184875	SkeletalMuscle	10.78	-0.16	n/a	-0.99
ABCB1	P-glycoprotein 1	n/a	6.22184875	Skin	6.58	-0.25	n/a	-1.56
ABCB1	P-glycoprotein 1	n/a	6.22184875	Small_intestine	22.03	0.09	n/a	0.54
ABCB1	P-glycoprotein 1	n/a	6.22184875	SmoothMuscle	7.18	-0.24	n/a	-1.47
ABCB1	P-glycoprotein 1	n/a	6.22184875	Spinalcord	8.85	-0.20	n/a	-1.25
ABCB1	P-glycoprotein 1	n/a	6.22184875	SubthalamicNucleus	6.73	-0.25	n/a	-1.53
ABCB1	P-glycoprotein 1	n/a	6.22184875	SuperiorCervicalGanglion	8.83	-0.20	n/a	-1.25
ABCB1	P-glycoprotein 1	n/a	6.22184875	TemporalLobe	6.20	-0.26	n/a	-1.61
ABCB1	P-glycoprotein 1	n/a	6.22184875	Testis	5.68	-0.27	n/a	-1.68
ABCB1	P-glycoprotein 1	n/a	6.22184875	TestisGermCell	5.08	-0.28	n/a	-1.76
ABCB1	P-glycoprotein 1	n/a	6.22184875	TestisIntersitial	5.38	-0.28	n/a	-1.72
ABCB1	P-glycoprotein 1	n/a	6.22184875	TestisLeydigCell	7.65	-0.23	n/a	-1.41
ABCB1	P-glycoprotein 1	n/a	6.22184875	TestisSeminiferousTubule	5.75	-0.27	n/a	-1.67
ABCB1	P-glycoprotein 1	n/a	6.22184875	Thalamus	9.85	-0.18	n/a	-1.11
ABCB1	P-glycoprotein 1	n/a	6.22184875	Thymus	4.73	-0.29	n/a	-1.81
ABCB1	P-glycoprotein 1	n/a	6.22184875	Thyroid	8.13	-0.22	n/a	-1.35
ABCB1	P-glycoprotein 1	n/a	6.22184875	Tongue	7.55	-0.23	n/a	-1.42
ABCB1	P-glycoprotein 1	n/a	6.22184875	Tonsil	7.05	-0.24	n/a	-1.49
ABCB1	P-glycoprotein 1	n/a	6.22184875	Trachea	5.58	-0.27	n/a	-1.69
ABCB1	P-glycoprotein 1	n/a	6.22184875	TrigeminalGanglion	8.38	-0.21	n/a	-1.31
ABCB1	P-glycoprotein 1	n/a	6.22184875	Uterus	15.60	-0.05	n/a	-0.33
ABCB1	P-glycoprotein 1	n/a	6.22184875	UterusCorpus	10.63	-0.16	n/a	-1.01
ABCB1	P-glycoprotein 1	n/a	6.22184875	WholeBlood	7.28	-0.23	n/a	-1.46
ABCB1	P-glycoprotein 1	n/a	6.22184875	Wholebrain	5.30	-0.28	n/a	-1.73
ADRA1A	Alpha-1a adrenergic receptor	7.657577319	n/a	Adipocyte	8.10	-0.24	-1.85	n/a
ADRA1A	Alpha-1a adrenergic receptor	7.657577319	n/a	AdrenalCortex	9.65	0.69	5.29	n/a
ADRA1A	Alpha-1a adrenergic receptor	7.657577319	n/a	Adrenalgland	8.15	-0.21	-1.62	n/a
ADRA1A	Alpha-1a adrenergic receptor	7.657577319	n/a	Amygdala	8.23	-0.17	-1.27	n/a
ADRA1A	Alpha-1a adrenergic receptor	7.657577319	n/a	Appendix	9.55	0.63	4.83	n/a
ADRA1A	Alpha-1a adrenergic receptor	7.657577319	n/a	AtrioventricularNode	9.10	0.36	2.76	n/a
ADRA1A	Alpha-1a adrenergic receptor	7.657577319	n/a	BDCA4+_DentriticCells	8.15	-0.21	-1.62	n/a
ADRA1A	Alpha-1a adrenergic receptor	7.657577319	n/a	Bonemarrow	9.65	0.69	5.29	n/a
ADRA1A	Alpha-1a adrenergic receptor	7.657577319	n/a	BronchialEpithelialCells	7.60	-0.54	-4.15	n/a
ADRA1A	Alpha-1a adrenergic receptor	7.657577319	n/a	CardiacMyocytes	14.53	3.62	27.76	n/a
ADRA1A	Alpha-1a adrenergic receptor	7.657577319	n/a	Caudatenucleus	7.38	-0.68	-5.19	n/a
ADRA1A	Alpha-1a adrenergic receptor	7.657577319	n/a	CD105+_Endothelial	8.20	-0.18	-1.39	n/a
ADRA1A	Alpha-1a adrenergic receptor	7.657577319	n/a	CD14+_Monocytes	8.73	0.13	1.03	n/a
ADRA1A	Alpha-1a adrenergic receptor	7.657577319	n/a	CD19+_BCells(neg._sel.)	8.45	-0.03	-0.24	n/a
ADRA1A	Alpha-1a adrenergic receptor	7.657577319	n/a	CD33+_Myeloid	10.08	0.95	7.25	n/a
ADRA1A	Alpha-1a adrenergic receptor	7.657577319	n/a	CD34+	9.93	0.86	6.56	n/a
ADRA1A	Alpha-1a adrenergic receptor	7.657577319	n/a	CD4+_Tcells	8.40	-0.06	-0.47	n/a
ADRA1A	Alpha-1a adrenergic receptor	7.657577319	n/a	CD56+_NKCells	8.68	0.10	0.80	n/a
ADRA1A	Alpha-1a adrenergic receptor	7.657577319	n/a	CD71+_EarlyErythroid	7.83	-0.41	-3.12	n/a
ADRA1A	Alpha-1a adrenergic receptor	7.657577319	n/a	CD8+_Tcells	7.48	-0.62	-4.73	n/a
ADRA1A	Alpha-1a adrenergic receptor	7.657577319	n/a	Cerebellum	6.70	-1.08	-8.30	n/a
ADRA1A	Alpha-1a adrenergic receptor	7.657577319	n/a	CerebellumPeduncles	10.38	1.13	8.63	n/a
ADRA1A	Alpha-1a adrenergic receptor	7.657577319	n/a	CiliaryGanglion	7.73	-0.47	-3.58	n/a
ADRA1A	Alpha-1a adrenergic receptor	7.657577319	n/a	CingulateCortex	8.65	0.09	0.69	n/a
ADRA1A	Alpha-1a adrenergic receptor	7.657577319	n/a	Colon	8.25	-0.15	-1.16	n/a
ADRA1A	Alpha-1a adrenergic receptor	7.657577319	n/a	DorsalRootGanglion	7.90	-0.36	-2.77	n/a
ADRA1A	Alpha-1a adrenergic receptor	7.657577319	n/a	Fetalbrain	7.88	-0.38	-2.89	n/a
ADRA1A	Alpha-1a adrenergic receptor	7.657577319	n/a	Fetalliver	7.25	-0.75	-5.77	n/a
ADRA1A	Alpha-1a adrenergic receptor	7.657577319	n/a	Fetallung	6.18	-1.40	-10.72	n/a

S2 Table. Target-tissue analysis for clozapine and chlorpromazine

Gene Symbol	Gene Name	Affinity (-log10(Ki))		Tissue	Expression		Combined Score (-log10(Ki)*Z)	
		clozapine	chlorpromazine		Raw-numbers	Z-score	clozapine	chlorpromazine
ADRA1A	Alpha-1a adrenergic receptor	7.657577319	n/a	FetalThyroid	8.18	-0.20	-1.50	n/a
ADRA1A	Alpha-1a adrenergic receptor	7.657577319	n/a	GlobusPallidus	6.98	-0.92	-7.03	n/a
ADRA1A	Alpha-1a adrenergic receptor	7.657577319	n/a	Heart	13.35	2.92	22.34	n/a
ADRA1A	Alpha-1a adrenergic receptor	7.657577319	n/a	Hypothalamus	8.53	0.01	0.11	n/a
ADRA1A	Alpha-1a adrenergic receptor	7.657577319	n/a	Kidney	8.73	0.13	1.03	n/a
ADRA1A	Alpha-1a adrenergic receptor	7.657577319	n/a	Liver	13.00	2.71	20.73	n/a
ADRA1A	Alpha-1a adrenergic receptor	7.657577319	n/a	Lung	8.88	0.22	1.72	n/a
ADRA1A	Alpha-1a adrenergic receptor	7.657577319	n/a	Lymphnode	6.88	-0.98	-7.49	n/a
ADRA1A	Alpha-1a adrenergic receptor	7.657577319	n/a	MedullaOblongata	7.48	-0.62	-4.73	n/a
ADRA1A	Alpha-1a adrenergic receptor	7.657577319	n/a	OccipitalLobe	7.38	-0.68	-5.19	n/a
ADRA1A	Alpha-1a adrenergic receptor	7.657577319	n/a	OlfactoryBulb	6.38	-1.28	-9.80	n/a
ADRA1A	Alpha-1a adrenergic receptor	7.657577319	n/a	Ovary	5.98	-1.52	-11.64	n/a
ADRA1A	Alpha-1a adrenergic receptor	7.657577319	n/a	Pancreas	6.65	-1.11	-8.53	n/a
ADRA1A	Alpha-1a adrenergic receptor	7.657577319	n/a	PancreaticIslet	8.93	0.25	1.95	n/a
ADRA1A	Alpha-1a adrenergic receptor	7.657577319	n/a	ParietalLobe	9.05	0.33	2.53	n/a
ADRA1A	Alpha-1a adrenergic receptor	7.657577319	n/a	Pineal_day	10.15	0.99	7.60	n/a
ADRA1A	Alpha-1a adrenergic receptor	7.657577319	n/a	Pineal_night	9.75	0.75	5.75	n/a
ADRA1A	Alpha-1a adrenergic receptor	7.657577319	n/a	Pituitary	9.48	0.59	4.49	n/a
ADRA1A	Alpha-1a adrenergic receptor	7.657577319	n/a	Placenta	7.93	-0.35	-2.66	n/a
ADRA1A	Alpha-1a adrenergic receptor	7.657577319	n/a	Pons	10.45	1.17	8.98	n/a
ADRA1A	Alpha-1a adrenergic receptor	7.657577319	n/a	PrefrontalCortex	9.80	0.78	5.98	n/a
ADRA1A	Alpha-1a adrenergic receptor	7.657577319	n/a	Prostate	9.20	0.42	3.22	n/a
ADRA1A	Alpha-1a adrenergic receptor	7.657577319	n/a	Retina	9.90	0.84	6.44	n/a
ADRA1A	Alpha-1a adrenergic receptor	7.657577319	n/a	Salivarygland	6.80	-1.02	-7.84	n/a
ADRA1A	Alpha-1a adrenergic receptor	7.657577319	n/a	SkeletalMuscle	12.15	2.20	16.81	n/a
ADRA1A	Alpha-1a adrenergic receptor	7.657577319	n/a	Skin	7.40	-0.66	-5.07	n/a
ADRA1A	Alpha-1a adrenergic receptor	7.657577319	n/a	Small_intestine	7.88	-0.38	-2.89	n/a
ADRA1A	Alpha-1a adrenergic receptor	7.657577319	n/a	SmoothMuscle	9.08	0.35	2.64	n/a
ADRA1A	Alpha-1a adrenergic receptor	7.657577319	n/a	Spinalcord	8.80	0.18	1.38	n/a
ADRA1A	Alpha-1a adrenergic receptor	7.657577319	n/a	SubthalamicNucleus	8.05	-0.27	-2.08	n/a
ADRA1A	Alpha-1a adrenergic receptor	7.657577319	n/a	SuperiorCervicalGanglion	10.75	1.35	10.36	n/a
ADRA1A	Alpha-1a adrenergic receptor	7.657577319	n/a	TemporalLobe	7.80	-0.42	-3.23	n/a
ADRA1A	Alpha-1a adrenergic receptor	7.657577319	n/a	Testis	6.98	-0.92	-7.03	n/a
ADRA1A	Alpha-1a adrenergic receptor	7.657577319	n/a	TestisGermCell	6.35	-1.29	-9.91	n/a
ADRA1A	Alpha-1a adrenergic receptor	7.657577319	n/a	TestisIntersitial	6.98	-0.92	-7.03	n/a
ADRA1A	Alpha-1a adrenergic receptor	7.657577319	n/a	TestisLeydigCell	8.35	-0.09	-0.70	n/a
ADRA1A	Alpha-1a adrenergic receptor	7.657577319	n/a	TestisSeminiferousTubule	7.00	-0.90	-6.92	n/a
ADRA1A	Alpha-1a adrenergic receptor	7.657577319	n/a	Thalamus	8.48	-0.02	-0.12	n/a
ADRA1A	Alpha-1a adrenergic receptor	7.657577319	n/a	Thymus	5.83	-1.61	-12.33	n/a
ADRA1A	Alpha-1a adrenergic receptor	7.657577319	n/a	Thyroid	9.83	0.80	6.10	n/a
ADRA1A	Alpha-1a adrenergic receptor	7.657577319	n/a	Tongue	8.85	0.21	1.61	n/a
ADRA1A	Alpha-1a adrenergic receptor	7.657577319	n/a	Tonsil	8.08	-0.26	-1.96	n/a
ADRA1A	Alpha-1a adrenergic receptor	7.657577319	n/a	Trachea	6.85	-0.99	-7.61	n/a
ADRA1A	Alpha-1a adrenergic receptor	7.657577319	n/a	TrigeminalGanglion	11.43	1.76	13.47	n/a
ADRA1A	Alpha-1a adrenergic receptor	7.657577319	n/a	Uterus	6.45	-1.23	-9.45	n/a
ADRA1A	Alpha-1a adrenergic receptor	7.657577319	n/a	UterusCorpus	7.98	-0.32	-2.43	n/a
ADRA1A	Alpha-1a adrenergic receptor	7.657577319	n/a	WholeBlood	8.63	0.07	0.57	n/a
ADRA1A	Alpha-1a adrenergic receptor	7.657577319	n/a	Wholebrain	6.18	-1.40	-10.72	n/a
ADRA1B	Alpha-1b adrenergic receptor	8.444905551	8.304518324	Adipocyte	2.85	0.00	0.03	0.03
ADRA1B	Alpha-1b adrenergic receptor	8.444905551	8.304518324	AdrenalCortex	2.95	0.22	1.85	1.82
ADRA1B	Alpha-1b adrenergic receptor	8.444905551	8.304518324	Adrenalgland	2.40	-0.96	-8.12	-7.98
ADRA1B	Alpha-1b adrenergic receptor	8.444905551	8.304518324	Amygdala	3.05	0.43	3.66	3.60
ADRA1B	Alpha-1b adrenergic receptor	8.444905551	8.304518324	Appendix	2.85	0.00	0.03	0.03

S2 Table. Target-tissue analysis for clozapine and chlorpromazine

Gene Symbol	Gene Name	Affinity (-log10(Ki))		Tissue	Expression		Combined Score (-log10(Ki)*Z)	
		clozapine	chlorpromazine		Raw-numbers	Z-score	clozapine	chlorpromazine
ADRA1B	Alpha-1b adrenergic receptor	8.444905551	8.304518324	AtrioventricularNode	2.15	-1.50	-12.65	-12.44
ADRA1B	Alpha-1b adrenergic receptor	8.444905551	8.304518324	BDCA4+_DentriticCells	3.20	0.76	6.38	6.27
ADRA1B	Alpha-1b adrenergic receptor	8.444905551	8.304518324	Bonemarrow	2.80	-0.10	-0.87	-0.86
ADRA1B	Alpha-1b adrenergic receptor	8.444905551	8.304518324	BronchialEpithelialCells	2.85	0.00	0.03	0.03
ADRA1B	Alpha-1b adrenergic receptor	8.444905551	8.304518324	CardiacMyocytes	3.55	1.51	12.72	12.51
ADRA1B	Alpha-1b adrenergic receptor	8.444905551	8.304518324	Caudatenucleus	2.60	-0.53	-4.50	-4.42
ADRA1B	Alpha-1b adrenergic receptor	8.444905551	8.304518324	CD105+_Endothelial	2.90	0.11	0.94	0.92
ADRA1B	Alpha-1b adrenergic receptor	8.444905551	8.304518324	CD14+_Monocytes	3.10	0.54	4.56	4.49
ADRA1B	Alpha-1b adrenergic receptor	8.444905551	8.304518324	CD19+_BCells(neg._sel.)	3.10	0.54	4.56	4.49
ADRA1B	Alpha-1b adrenergic receptor	8.444905551	8.304518324	CD33+_Myeloid	3.70	1.83	15.44	15.18
ADRA1B	Alpha-1b adrenergic receptor	8.444905551	8.304518324	CD34+	3.65	1.72	14.53	14.29
ADRA1B	Alpha-1b adrenergic receptor	8.444905551	8.304518324	CD4+_Tcells	3.15	0.65	5.47	5.38
ADRA1B	Alpha-1b adrenergic receptor	8.444905551	8.304518324	CD56+_NKCells	3.50	1.40	11.81	11.62
ADRA1B	Alpha-1b adrenergic receptor	8.444905551	8.304518324	CD71+_EarlyErythroid	2.70	-0.32	-2.68	-2.64
ADRA1B	Alpha-1b adrenergic receptor	8.444905551	8.304518324	CD8+_Tcells	2.80	-0.10	-0.87	-0.86
ADRA1B	Alpha-1b adrenergic receptor	8.444905551	8.304518324	Cerebellum	2.35	-1.07	-9.03	-8.88
ADRA1B	Alpha-1b adrenergic receptor	8.444905551	8.304518324	CerebellumPeduncles	3.25	0.86	7.28	7.16
ADRA1B	Alpha-1b adrenergic receptor	8.444905551	8.304518324	CiliaryGanglion	2.00	-1.82	-15.37	-15.11
ADRA1B	Alpha-1b adrenergic receptor	8.444905551	8.304518324	CingulateCortex	2.95	0.22	1.85	1.82
ADRA1B	Alpha-1b adrenergic receptor	8.444905551	8.304518324	Colon	2.90	0.11	0.94	0.92
ADRA1B	Alpha-1b adrenergic receptor	8.444905551	8.304518324	DorsalRootGanglion	2.15	-1.50	-12.65	-12.44
ADRA1B	Alpha-1b adrenergic receptor	8.444905551	8.304518324	Fetalbrain	2.90	0.11	0.94	0.92
ADRA1B	Alpha-1b adrenergic receptor	8.444905551	8.304518324	Fetalliver	2.45	-0.85	-7.21	-7.09
ADRA1B	Alpha-1b adrenergic receptor	8.444905551	8.304518324	Fetallung	2.40	-0.96	-8.12	-7.98
ADRA1B	Alpha-1b adrenergic receptor	8.444905551	8.304518324	FetalThyroid	2.80	-0.10	-0.87	-0.86
ADRA1B	Alpha-1b adrenergic receptor	8.444905551	8.304518324	GlobusPallidus	2.10	-1.61	-13.56	-13.33
ADRA1B	Alpha-1b adrenergic receptor	8.444905551	8.304518324	Heart	3.65	1.72	14.53	14.29
ADRA1B	Alpha-1b adrenergic receptor	8.444905551	8.304518324	Hypothalamus	3.20	0.76	6.38	6.27
ADRA1B	Alpha-1b adrenergic receptor	8.444905551	8.304518324	Kidney	2.30	-1.18	-9.93	-9.77
ADRA1B	Alpha-1b adrenergic receptor	8.444905551	8.304518324	Liver	3.75	1.94	16.34	16.07
ADRA1B	Alpha-1b adrenergic receptor	8.444905551	8.304518324	Lung	3.20	0.76	6.38	6.27
ADRA1B	Alpha-1b adrenergic receptor	8.444905551	8.304518324	Lymphnode	2.50	-0.75	-6.31	-6.20
ADRA1B	Alpha-1b adrenergic receptor	8.444905551	8.304518324	MedullaOblongata	2.65	-0.43	-3.59	-3.53
ADRA1B	Alpha-1b adrenergic receptor	8.444905551	8.304518324	OccipitalLobe	2.55	-0.64	-5.40	-5.31
ADRA1B	Alpha-1b adrenergic receptor	8.444905551	8.304518324	OlfactoryBulb	2.25	-1.28	-10.84	-10.66
ADRA1B	Alpha-1b adrenergic receptor	8.444905551	8.304518324	Ovary	1.90	-2.03	-17.18	-16.89
ADRA1B	Alpha-1b adrenergic receptor	8.444905551	8.304518324	Pancreas	2.25	-1.28	-10.84	-10.66
ADRA1B	Alpha-1b adrenergic receptor	8.444905551	8.304518324	PancreaticIslet	3.05	0.43	3.66	3.60
ADRA1B	Alpha-1b adrenergic receptor	8.444905551	8.304518324	ParietalLobe	3.00	0.33	2.75	2.71
ADRA1B	Alpha-1b adrenergic receptor	8.444905551	8.304518324	Pineal_day	3.62	1.66	13.99	13.75
ADRA1B	Alpha-1b adrenergic receptor	8.444905551	8.304518324	Pineal_night	3.56	1.53	12.90	12.69
ADRA1B	Alpha-1b adrenergic receptor	8.444905551	8.304518324	Pituitary	3.35	1.08	9.09	8.94
ADRA1B	Alpha-1b adrenergic receptor	8.444905551	8.304518324	Placenta	3.05	0.43	3.66	3.60
ADRA1B	Alpha-1b adrenergic receptor	8.444905551	8.304518324	Pons	2.75	-0.21	-1.78	-1.75
ADRA1B	Alpha-1b adrenergic receptor	8.444905551	8.304518324	PrefrontalCortex	3.65	1.72	14.53	14.29
ADRA1B	Alpha-1b adrenergic receptor	8.444905551	8.304518324	Prostate	3.40	1.18	10.00	9.83
ADRA1B	Alpha-1b adrenergic receptor	8.444905551	8.304518324	Retina	3.53	1.45	12.27	12.06
ADRA1B	Alpha-1b adrenergic receptor	8.444905551	8.304518324	Salivarygland	2.30	-1.18	-9.93	-9.77
ADRA1B	Alpha-1b adrenergic receptor	8.444905551	8.304518324	SkeletalMuscle	3.25	0.86	7.28	7.16
ADRA1B	Alpha-1b adrenergic receptor	8.444905551	8.304518324	Skin	2.15	-1.50	-12.65	-12.44
ADRA1B	Alpha-1b adrenergic receptor	8.444905551	8.304518324	Small_intestine	2.80	-0.10	-0.87	-0.86
ADRA1B	Alpha-1b adrenergic receptor	8.444905551	8.304518324	SmoothMuscle	3.25	0.86	7.28	7.16
ADRA1B	Alpha-1b adrenergic receptor	8.444905551	8.304518324	Spinalcord	3.05	0.43	3.66	3.60

S2 Table. Target-tissue analysis for clozapine and chlorpromazine

Gene Symbol	Gene Name	Affinity (-log10(Ki))		Tissue	Expression		Combined Score (-log10(Ki)*Z)	
		clozapine	chlorpromazine		Raw-numbers	Z-score	clozapine	chlorpromazine
ADRA1B	Alpha-1b adrenergic receptor	8.444905551	8.304518324	SubthalamicNucleus	2.60	-0.53	-4.50	-4.42
ADRA1B	Alpha-1b adrenergic receptor	8.444905551	8.304518324	SuperiorCervicalGanglion	3.20	0.76	6.38	6.27
ADRA1B	Alpha-1b adrenergic receptor	8.444905551	8.304518324	TemporalLobe	2.60	-0.53	-4.50	-4.42
ADRA1B	Alpha-1b adrenergic receptor	8.444905551	8.304518324	Testis	2.50	-0.75	-6.31	-6.20
ADRA1B	Alpha-1b adrenergic receptor	8.444905551	8.304518324	TestisGermCell	2.45	-0.85	-7.21	-7.09
ADRA1B	Alpha-1b adrenergic receptor	8.444905551	8.304518324	TestisIntersitial	2.45	-0.85	-7.21	-7.09
ADRA1B	Alpha-1b adrenergic receptor	8.444905551	8.304518324	TestisLeydigCell	2.80	-0.10	-0.87	-0.86
ADRA1B	Alpha-1b adrenergic receptor	8.444905551	8.304518324	TestisSeminiferousTubule	2.45	-0.85	-7.21	-7.09
ADRA1B	Alpha-1b adrenergic receptor	8.444905551	8.304518324	Thalamus	2.90	0.11	0.94	0.92
ADRA1B	Alpha-1b adrenergic receptor	8.444905551	8.304518324	Thymus	2.25	-1.28	-10.84	-10.66
ADRA1B	Alpha-1b adrenergic receptor	8.444905551	8.304518324	Thyroid	3.65	1.72	14.53	14.29
ADRA1B	Alpha-1b adrenergic receptor	8.444905551	8.304518324	Tongue	2.75	-0.21	-1.78	-1.75
ADRA1B	Alpha-1b adrenergic receptor	8.444905551	8.304518324	Tonsil	2.80	-0.10	-0.87	-0.86
ADRA1B	Alpha-1b adrenergic receptor	8.444905551	8.304518324	Trachea	2.40	-0.96	-8.12	-7.98
ADRA1B	Alpha-1b adrenergic receptor	8.444905551	8.304518324	TrigeminalGanglion	2.80	-0.10	-0.87	-0.86
ADRA1B	Alpha-1b adrenergic receptor	8.444905551	8.304518324	Uterus	2.40	-0.96	-8.12	-7.98
ADRA1B	Alpha-1b adrenergic receptor	8.444905551	8.304518324	UterusCorpus	2.55	-0.64	-5.40	-5.31
ADRA1B	Alpha-1b adrenergic receptor	8.444905551	8.304518324	WholeBlood	3.20	0.76	6.38	6.27
ADRA1B	Alpha-1b adrenergic receptor	8.444905551	8.304518324	Wholebrain	2.50	-0.75	-6.31	-6.20
ADRA1D	Alpha-1d adrenergic receptor	7.769551079	8.707743929	Adipocyte	6.60	0.01	0.10	0.11
ADRA1D	Alpha-1d adrenergic receptor	7.769551079	8.707743929	AdrenalCortex	8.10	1.05	8.13	9.11
ADRA1D	Alpha-1d adrenergic receptor	7.769551079	8.707743929	Adrenalgland	6.43	-0.11	-0.83	-0.93
ADRA1D	Alpha-1d adrenergic receptor	7.769551079	8.707743929	Amygdala	5.90	-0.47	-3.64	-4.08
ADRA1D	Alpha-1d adrenergic receptor	7.769551079	8.707743929	Appendix	6.68	0.06	0.50	0.56
ADRA1D	Alpha-1d adrenergic receptor	7.769551079	8.707743929	AtrioventricularNode	5.38	-0.83	-6.45	-7.23
ADRA1D	Alpha-1d adrenergic receptor	7.769551079	8.707743929	BDCA4+_DentriticCells	6.60	0.01	0.10	0.11
ADRA1D	Alpha-1d adrenergic receptor	7.769551079	8.707743929	Bonemarrow	6.73	0.10	0.77	0.86
ADRA1D	Alpha-1d adrenergic receptor	7.769551079	8.707743929	BronchialEpithelialCells	6.00	-0.40	-3.11	-3.48
ADRA1D	Alpha-1d adrenergic receptor	7.769551079	8.707743929	CardiacMyocytes	8.45	1.29	10.00	11.21
ADRA1D	Alpha-1d adrenergic receptor	7.769551079	8.707743929	Caudatenucleus	6.38	-0.14	-1.10	-1.23
ADRA1D	Alpha-1d adrenergic receptor	7.769551079	8.707743929	CD105+_Endothelial	5.90	-0.47	-3.64	-4.08
ADRA1D	Alpha-1d adrenergic receptor	7.769551079	8.707743929	CD14+_Monocytes	6.08	-0.35	-2.71	-3.03
ADRA1D	Alpha-1d adrenergic receptor	7.769551079	8.707743929	CD19+_BCells(neg._sel.)	6.10	-0.33	-2.57	-2.88
ADRA1D	Alpha-1d adrenergic receptor	7.769551079	8.707743929	CD33+_Myeloid	7.15	0.39	3.05	3.41
ADRA1D	Alpha-1d adrenergic receptor	7.769551079	8.707743929	CD34+	7.00	0.29	2.24	2.51
ADRA1D	Alpha-1d adrenergic receptor	7.769551079	8.707743929	CD4+_Tcells	6.00	-0.40	-3.11	-3.48
ADRA1D	Alpha-1d adrenergic receptor	7.769551079	8.707743929	CD56+_NKCells	6.53	-0.04	-0.30	-0.33
ADRA1D	Alpha-1d adrenergic receptor	7.769551079	8.707743929	CD71+_EarlyErythroid	5.58	-0.69	-5.38	-6.03
ADRA1D	Alpha-1d adrenergic receptor	7.769551079	8.707743929	CD8+_Tcells	5.13	-1.00	-7.79	-8.73
ADRA1D	Alpha-1d adrenergic receptor	7.769551079	8.707743929	Cerebellum	5.30	-0.88	-6.86	-7.68
ADRA1D	Alpha-1d adrenergic receptor	7.769551079	8.707743929	CerebellumPeduncles	8.18	1.10	8.53	9.56
ADRA1D	Alpha-1d adrenergic receptor	7.769551079	8.707743929	CiliaryGanglion	5.28	-0.90	-6.99	-7.83
ADRA1D	Alpha-1d adrenergic receptor	7.769551079	8.707743929	CingulateCortex	6.43	-0.11	-0.83	-0.93
ADRA1D	Alpha-1d adrenergic receptor	7.769551079	8.707743929	Colon	5.80	-0.54	-4.18	-4.68
ADRA1D	Alpha-1d adrenergic receptor	7.769551079	8.707743929	DorsalRootGanglion	6.05	-0.37	-2.84	-3.18
ADRA1D	Alpha-1d adrenergic receptor	7.769551079	8.707743929	Fetalbrain	6.33	-0.18	-1.37	-1.53
ADRA1D	Alpha-1d adrenergic receptor	7.769551079	8.707743929	Fetalliver	5.35	-0.85	-6.59	-7.38
ADRA1D	Alpha-1d adrenergic receptor	7.769551079	8.707743929	Fetallung	4.95	-1.12	-8.73	-9.78
ADRA1D	Alpha-1d adrenergic receptor	7.769551079	8.707743929	FetalThyroid	6.03	-0.38	-2.97	-3.33
ADRA1D	Alpha-1d adrenergic receptor	7.769551079	8.707743929	GlobusPallidus	5.68	-0.62	-4.85	-5.43
ADRA1D	Alpha-1d adrenergic receptor	7.769551079	8.707743929	Heart	8.50	1.32	10.27	11.51
ADRA1D	Alpha-1d adrenergic receptor	7.769551079	8.707743929	Hypothalamus	6.53	-0.04	-0.30	-0.33
ADRA1D	Alpha-1d adrenergic receptor	7.769551079	8.707743929	Kidney	6.88	0.20	1.57	1.76

S2 Table. Target-tissue analysis for clozapine and chlorpromazine

Gene Symbol	Gene Name	Affinity (-log10(Ki))		Tissue	Expression		Combined Score (-log10(Ki)*Z)	
		clozapine	chlorpromazine		Raw-numbers	Z-score	clozapine	chlorpromazine
ADRA1D	Alpha-1d adrenergic receptor	7.769551079	8.707743929	Liver	8.55	1.36	10.54	11.81
ADRA1D	Alpha-1d adrenergic receptor	7.769551079	8.707743929	Lung	6.70	0.08	0.64	0.71
ADRA1D	Alpha-1d adrenergic receptor	7.769551079	8.707743929	Lymphnode	5.35	-0.85	-6.59	-7.38
ADRA1D	Alpha-1d adrenergic receptor	7.769551079	8.707743929	MedullaOblongata	6.13	-0.31	-2.44	-2.73
ADRA1D	Alpha-1d adrenergic receptor	7.769551079	8.707743929	OccipitalLobe	6.18	-0.28	-2.17	-2.43
ADRA1D	Alpha-1d adrenergic receptor	7.769551079	8.707743929	OlfactoryBulb	4.95	-1.12	-8.73	-9.78
ADRA1D	Alpha-1d adrenergic receptor	7.769551079	8.707743929	Ovary	4.98	-1.11	-8.59	-9.63
ADRA1D	Alpha-1d adrenergic receptor	7.769551079	8.707743929	Pancreas	5.28	-0.90	-6.99	-7.83
ADRA1D	Alpha-1d adrenergic receptor	7.769551079	8.707743929	PancreaticIslet	6.83	0.17	1.31	1.46
ADRA1D	Alpha-1d adrenergic receptor	7.769551079	8.707743929	ParietalLobe	8.88	1.58	12.28	13.76
ADRA1D	Alpha-1d adrenergic receptor	7.769551079	8.707743929	Pineal_day	7.28	0.48	3.74	4.19
ADRA1D	Alpha-1d adrenergic receptor	7.769551079	8.707743929	Pineal_night	7.12	0.37	2.89	3.23
ADRA1D	Alpha-1d adrenergic receptor	7.769551079	8.707743929	Pituitary	7.43	0.58	4.52	5.06
ADRA1D	Alpha-1d adrenergic receptor	7.769551079	8.707743929	Placenta	6.13	-0.31	-2.44	-2.73
ADRA1D	Alpha-1d adrenergic receptor	7.769551079	8.707743929	Pons	9.63	2.10	16.29	18.26
ADRA1D	Alpha-1d adrenergic receptor	7.769551079	8.707743929	PrefrontalCortex	7.23	0.44	3.45	3.86
ADRA1D	Alpha-1d adrenergic receptor	7.769551079	8.707743929	Prostate	6.78	0.13	1.04	1.16
ADRA1D	Alpha-1d adrenergic receptor	7.769551079	8.707743929	Retina	7.65	0.74	5.72	6.41
ADRA1D	Alpha-1d adrenergic receptor	7.769551079	8.707743929	Salivarygland	5.43	-0.80	-6.19	-6.93
ADRA1D	Alpha-1d adrenergic receptor	7.769551079	8.707743929	SkeletalMuscle	15.40	6.08	47.20	52.90
ADRA1D	Alpha-1d adrenergic receptor	7.769551079	8.707743929	Skin	6.38	-0.14	-1.10	-1.23
ADRA1D	Alpha-1d adrenergic receptor	7.769551079	8.707743929	Small_intestine	5.48	-0.76	-5.92	-6.63
ADRA1D	Alpha-1d adrenergic receptor	7.769551079	8.707743929	SmoothMuscle	6.95	0.25	1.98	2.21
ADRA1D	Alpha-1d adrenergic receptor	7.769551079	8.707743929	Spinalcord	7.55	0.67	5.19	5.81
ADRA1D	Alpha-1d adrenergic receptor	7.769551079	8.707743929	SubthalamicNucleus	5.98	-0.42	-3.24	-3.63
ADRA1D	Alpha-1d adrenergic receptor	7.769551079	8.707743929	SuperiorCervicalGanglion	8.08	1.03	8.00	8.96
ADRA1D	Alpha-1d adrenergic receptor	7.769551079	8.707743929	TemporalLobe	6.53	-0.04	-0.30	-0.33
ADRA1D	Alpha-1d adrenergic receptor	7.769551079	8.707743929	Testis	5.23	-0.93	-7.26	-8.13
ADRA1D	Alpha-1d adrenergic receptor	7.769551079	8.707743929	TestisGermCell	6.00	-0.40	-3.11	-3.48
ADRA1D	Alpha-1d adrenergic receptor	7.769551079	8.707743929	TestisIntersitial	5.35	-0.85	-6.59	-7.38
ADRA1D	Alpha-1d adrenergic receptor	7.769551079	8.707743929	TestisLeydigCell	7.08	0.34	2.64	2.96
ADRA1D	Alpha-1d adrenergic receptor	7.769551079	8.707743929	TestisSeminiferousTubule	6.58	0.00	-0.03	-0.04
ADRA1D	Alpha-1d adrenergic receptor	7.769551079	8.707743929	Thalamus	6.93	0.24	1.84	2.06
ADRA1D	Alpha-1d adrenergic receptor	7.769551079	8.707743929	Thymus	4.80	-1.23	-9.53	-10.68
ADRA1D	Alpha-1d adrenergic receptor	7.769551079	8.707743929	Thyroid	6.98	0.27	2.11	2.36
ADRA1D	Alpha-1d adrenergic receptor	7.769551079	8.707743929	Tongue	7.60	0.70	5.45	6.11
ADRA1D	Alpha-1d adrenergic receptor	7.769551079	8.707743929	Tonsil	7.05	0.32	2.51	2.81
ADRA1D	Alpha-1d adrenergic receptor	7.769551079	8.707743929	Trachea	5.45	-0.78	-6.05	-6.78
ADRA1D	Alpha-1d adrenergic receptor	7.769551079	8.707743929	TrigeminalGanglion	6.55	-0.02	-0.17	-0.19
ADRA1D	Alpha-1d adrenergic receptor	7.769551079	8.707743929	Uterus	4.75	-1.26	-9.80	-10.98
ADRA1D	Alpha-1d adrenergic receptor	7.769551079	8.707743929	UterusCorpus	7.88	0.89	6.93	7.76
ADRA1D	Alpha-1d adrenergic receptor	7.769551079	8.707743929	WholeBlood	6.48	-0.07	-0.57	-0.63
ADRA1D	Alpha-1d adrenergic receptor	7.769551079	8.707743929	Wholebrain	5.35	-0.85	-6.59	-7.38
ADRA2A	Alpha-2a adrenergic receptor	7.619788758	6.879426069	Adipocyte	13.50	1.81	13.80	12.46
ADRA2A	Alpha-2a adrenergic receptor	7.619788758	6.879426069	AdrenalCortex	7.65	-0.21	-1.60	-1.44
ADRA2A	Alpha-2a adrenergic receptor	7.619788758	6.879426069	Adrenalgland	6.40	-0.64	-4.89	-4.41
ADRA2A	Alpha-2a adrenergic receptor	7.619788758	6.879426069	Amygdala	8.50	0.08	0.64	0.58
ADRA2A	Alpha-2a adrenergic receptor	7.619788758	6.879426069	Appendix	8.35	0.03	0.25	0.22
ADRA2A	Alpha-2a adrenergic receptor	7.619788758	6.879426069	AtrioventricularNode	6.40	-0.64	-4.89	-4.41
ADRA2A	Alpha-2a adrenergic receptor	7.619788758	6.879426069	BDCA4+_DentriticCells	6.95	-0.45	-3.44	-3.10
ADRA2A	Alpha-2a adrenergic receptor	7.619788758	6.879426069	Bonemarrow	8.05	-0.07	-0.54	-0.49
ADRA2A	Alpha-2a adrenergic receptor	7.619788758	6.879426069	BronchialEpithelialCells	6.80	-0.50	-3.83	-3.46
ADRA2A	Alpha-2a adrenergic receptor	7.619788758	6.879426069	CardiacMyocytes	12.40	1.43	10.91	9.85

S2 Table. Target-tissue analysis for clozapine and chlorpromazine

Gene Symbol	Gene Name	Affinity (-log10(Ki))		Tissue	Expression		Combined Score (-log10(Ki)*Z)	
		clozapine	chlorpromazine		Raw-numbers	Z-score	clozapine	chlorpromazine
ADRA2A	Alpha-2a adrenergic receptor	7.619788758	6.879426069	Caudatenucleus	6.45	-0.62	-4.76	-4.29
ADRA2A	Alpha-2a adrenergic receptor	7.619788758	6.879426069	CD105+_Endothelial	6.90	-0.47	-3.57	-3.22
ADRA2A	Alpha-2a adrenergic receptor	7.619788758	6.879426069	CD14+_Monocytes	7.60	-0.23	-1.73	-1.56
ADRA2A	Alpha-2a adrenergic receptor	7.619788758	6.879426069	CD19+_BCells(neg._sel.)	7.20	-0.36	-2.78	-2.51
ADRA2A	Alpha-2a adrenergic receptor	7.619788758	6.879426069	CD33+_Myeloid	8.50	0.08	0.64	0.58
ADRA2A	Alpha-2a adrenergic receptor	7.619788758	6.879426069	CD34+	10.55	0.79	6.04	5.45
ADRA2A	Alpha-2a adrenergic receptor	7.619788758	6.879426069	CD4+_Tcells	7.05	-0.42	-3.18	-2.87
ADRA2A	Alpha-2a adrenergic receptor	7.619788758	6.879426069	CD56+_NKCells	7.70	-0.19	-1.46	-1.32
ADRA2A	Alpha-2a adrenergic receptor	7.619788758	6.879426069	CD71+_EarlyErythroid	6.55	-0.59	-4.49	-4.06
ADRA2A	Alpha-2a adrenergic receptor	7.619788758	6.879426069	CD8+_Tcells	6.25	-0.69	-5.28	-4.77
ADRA2A	Alpha-2a adrenergic receptor	7.619788758	6.879426069	Cerebellum	6.25	-0.69	-5.28	-4.77
ADRA2A	Alpha-2a adrenergic receptor	7.619788758	6.879426069	CerebellumPeduncles	8.55	0.10	0.77	0.70
ADRA2A	Alpha-2a adrenergic receptor	7.619788758	6.879426069	CiliaryGanglion	6.20	-0.71	-5.41	-4.89
ADRA2A	Alpha-2a adrenergic receptor	7.619788758	6.879426069	CingulateCortex	7.35	-0.31	-2.39	-2.15
ADRA2A	Alpha-2a adrenergic receptor	7.619788758	6.879426069	Colon	7.05	-0.42	-3.18	-2.87
ADRA2A	Alpha-2a adrenergic receptor	7.619788758	6.879426069	DorsalRootGanglion	6.65	-0.55	-4.23	-3.82
ADRA2A	Alpha-2a adrenergic receptor	7.619788758	6.879426069	Fetalbrain	21.15	4.45	33.94	30.64
ADRA2A	Alpha-2a adrenergic receptor	7.619788758	6.879426069	Fetalliver	6.45	-0.62	-4.76	-4.29
ADRA2A	Alpha-2a adrenergic receptor	7.619788758	6.879426069	Fetallung	6.10	-0.74	-5.68	-5.12
ADRA2A	Alpha-2a adrenergic receptor	7.619788758	6.879426069	FetalThyroid	7.20	-0.36	-2.78	-2.51
ADRA2A	Alpha-2a adrenergic receptor	7.619788758	6.879426069	GlobusPallidus	5.35	-1.00	-7.65	-6.91
ADRA2A	Alpha-2a adrenergic receptor	7.619788758	6.879426069	Heart	10.25	0.69	5.25	4.74
ADRA2A	Alpha-2a adrenergic receptor	7.619788758	6.879426069	Hypothalamus	8.15	-0.04	-0.28	-0.25
ADRA2A	Alpha-2a adrenergic receptor	7.619788758	6.879426069	Kidney	6.25	-0.69	-5.28	-4.77
ADRA2A	Alpha-2a adrenergic receptor	7.619788758	6.879426069	Liver	11.45	1.10	8.41	7.59
ADRA2A	Alpha-2a adrenergic receptor	7.619788758	6.879426069	Lung	9.25	0.34	2.62	2.36
ADRA2A	Alpha-2a adrenergic receptor	7.619788758	6.879426069	Lymphnode	20.80	4.33	33.02	29.81
ADRA2A	Alpha-2a adrenergic receptor	7.619788758	6.879426069	MedullaOblongata	6.50	-0.61	-4.62	-4.17
ADRA2A	Alpha-2a adrenergic receptor	7.619788758	6.879426069	OccipitalLobe	6.60	-0.57	-4.36	-3.94
ADRA2A	Alpha-2a adrenergic receptor	7.619788758	6.879426069	OlfactoryBulb	5.65	-0.90	-6.86	-6.19
ADRA2A	Alpha-2a adrenergic receptor	7.619788758	6.879426069	Ovary	5.30	-1.02	-7.78	-7.03
ADRA2A	Alpha-2a adrenergic receptor	7.619788758	6.879426069	Pancreas	7.55	-0.24	-1.86	-1.68
ADRA2A	Alpha-2a adrenergic receptor	7.619788758	6.879426069	PancreaticIslet	17.00	3.02	23.02	20.78
ADRA2A	Alpha-2a adrenergic receptor	7.619788758	6.879426069	ParietalLobe	7.85	-0.14	-1.07	-0.97
ADRA2A	Alpha-2a adrenergic receptor	7.619788758	6.879426069	Pineal_day	8.74	0.17	1.27	1.15
ADRA2A	Alpha-2a adrenergic receptor	7.619788758	6.879426069	Pineal_night	8.56	0.10	0.80	0.72
ADRA2A	Alpha-2a adrenergic receptor	7.619788758	6.879426069	Pituitary	8.60	0.12	0.90	0.82
ADRA2A	Alpha-2a adrenergic receptor	7.619788758	6.879426069	Placenta	7.20	-0.36	-2.78	-2.51
ADRA2A	Alpha-2a adrenergic receptor	7.619788758	6.879426069	Pons	7.00	-0.43	-3.31	-2.99
ADRA2A	Alpha-2a adrenergic receptor	7.619788758	6.879426069	PrefrontalCortex	8.70	0.15	1.17	1.05
ADRA2A	Alpha-2a adrenergic receptor	7.619788758	6.879426069	Prostate	8.60	0.12	0.90	0.82
ADRA2A	Alpha-2a adrenergic receptor	7.619788758	6.879426069	Retina	9.70	0.50	3.80	3.43
ADRA2A	Alpha-2a adrenergic receptor	7.619788758	6.879426069	Salivarygland	6.00	-0.78	-5.94	-5.36
ADRA2A	Alpha-2a adrenergic receptor	7.619788758	6.879426069	SkeletalMuscle	8.55	0.10	0.77	0.70
ADRA2A	Alpha-2a adrenergic receptor	7.619788758	6.879426069	Skin	6.90	-0.47	-3.57	-3.22
ADRA2A	Alpha-2a adrenergic receptor	7.619788758	6.879426069	Small_intestine	6.90	-0.47	-3.57	-3.22
ADRA2A	Alpha-2a adrenergic receptor	7.619788758	6.879426069	SmoothMuscle	8.05	-0.07	-0.54	-0.49
ADRA2A	Alpha-2a adrenergic receptor	7.619788758	6.879426069	Spinalcord	7.65	-0.21	-1.60	-1.44
ADRA2A	Alpha-2a adrenergic receptor	7.619788758	6.879426069	SubthalamicNucleus	6.60	-0.57	-4.36	-3.94
ADRA2A	Alpha-2a adrenergic receptor	7.619788758	6.879426069	SuperiorCervicalGanglion	10.95	0.93	7.09	6.40
ADRA2A	Alpha-2a adrenergic receptor	7.619788758	6.879426069	TemporalLobe	6.80	-0.50	-3.83	-3.46
ADRA2A	Alpha-2a adrenergic receptor	7.619788758	6.879426069	Testis	8.85	0.21	1.56	1.41
ADRA2A	Alpha-2a adrenergic receptor	7.619788758	6.879426069	TestisGermCell	5.60	-0.92	-6.99	-6.31

S2 Table. Target-tissue analysis for clozapine and chlorpromazine

Gene Symbol	Gene Name	Affinity (-log10(Ki))		Tissue	Expression		Combined Score (-log10(Ki)*Z)	
		clozapine	chlorpromazine		Raw-numbers	Z-score	clozapine	chlorpromazine
ADRA2A	Alpha-2a adrenergic receptor	7.619788758	6.879426069	TestisIntersitial	6.05	-0.76	-5.81	-5.24
ADRA2A	Alpha-2a adrenergic receptor	7.619788758	6.879426069	TestisLeydigCell	7.25	-0.35	-2.65	-2.39
ADRA2A	Alpha-2a adrenergic receptor	7.619788758	6.879426069	TestisSeminiferousTubule	6.15	-0.73	-5.54	-5.01
ADRA2A	Alpha-2a adrenergic receptor	7.619788758	6.879426069	Thalamus	7.35	-0.31	-2.39	-2.15
ADRA2A	Alpha-2a adrenergic receptor	7.619788758	6.879426069	Thymus	5.40	-0.99	-7.52	-6.79
ADRA2A	Alpha-2a adrenergic receptor	7.619788758	6.879426069	Thyroid	9.35	0.38	2.88	2.60
ADRA2A	Alpha-2a adrenergic receptor	7.619788758	6.879426069	Tongue	7.60	-0.23	-1.73	-1.56
ADRA2A	Alpha-2a adrenergic receptor	7.619788758	6.879426069	Tonsil	10.35	0.72	5.51	4.98
ADRA2A	Alpha-2a adrenergic receptor	7.619788758	6.879426069	Trachea	9.15	0.31	2.35	2.12
ADRA2A	Alpha-2a adrenergic receptor	7.619788758	6.879426069	TrigeminalGanglion	8.70	0.15	1.17	1.05
ADRA2A	Alpha-2a adrenergic receptor	7.619788758	6.879426069	Uterus	11.60	1.16	8.80	7.95
ADRA2A	Alpha-2a adrenergic receptor	7.619788758	6.879426069	UterusCorpus	6.50	-0.61	-4.62	-4.17
ADRA2A	Alpha-2a adrenergic receptor	7.619788758	6.879426069	WholeBlood	7.80	-0.16	-1.20	-1.08
ADRA2A	Alpha-2a adrenergic receptor	7.619788758	6.879426069	Wholebrain	11.90	1.26	9.59	8.66
ADRA2B	Alpha-2b adrenergic receptor	7.958607315	7.920818754	Adipocyte	3.95	-0.05	-0.41	-0.41
ADRA2B	Alpha-2b adrenergic receptor	7.958607315	7.920818754	AdrenalCortex	4.15	0.25	2.00	1.99
ADRA2B	Alpha-2b adrenergic receptor	7.958607315	7.920818754	Adrenalgland	3.40	-0.88	-7.04	-7.01
ADRA2B	Alpha-2b adrenergic receptor	7.958607315	7.920818754	Amygdala	4.20	0.33	2.60	2.59
ADRA2B	Alpha-2b adrenergic receptor	7.958607315	7.920818754	Appendix	4.00	0.02	0.19	0.19
ADRA2B	Alpha-2b adrenergic receptor	7.958607315	7.920818754	AtrioventricularNode	3.10	-1.34	-10.65	-10.60
ADRA2B	Alpha-2b adrenergic receptor	7.958607315	7.920818754	BDCA4+_DentriticCells	4.45	0.71	5.62	5.59
ADRA2B	Alpha-2b adrenergic receptor	7.958607315	7.920818754	Bonemarrow	3.95	-0.05	-0.41	-0.41
ADRA2B	Alpha-2b adrenergic receptor	7.958607315	7.920818754	BronchialEpithelialCells	3.95	-0.05	-0.41	-0.41
ADRA2B	Alpha-2b adrenergic receptor	7.958607315	7.920818754	CardiacMyocytes	5.35	2.07	16.46	16.39
ADRA2B	Alpha-2b adrenergic receptor	7.958607315	7.920818754	Caudatenucleus	3.60	-0.58	-4.63	-4.61
ADRA2B	Alpha-2b adrenergic receptor	7.958607315	7.920818754	CD105+_Endothelial	4.05	0.10	0.80	0.79
ADRA2B	Alpha-2b adrenergic receptor	7.958607315	7.920818754	CD14+_Monocytes	4.30	0.48	3.81	3.79
ADRA2B	Alpha-2b adrenergic receptor	7.958607315	7.920818754	CD19+_BCells(neg._sel.)	4.25	0.40	3.21	3.19
ADRA2B	Alpha-2b adrenergic receptor	7.958607315	7.920818754	CD33+_Myeloid	5.20	1.84	14.66	14.59
ADRA2B	Alpha-2b adrenergic receptor	7.958607315	7.920818754	CD34+	5.10	1.69	13.45	13.39
ADRA2B	Alpha-2b adrenergic receptor	7.958607315	7.920818754	CD4+_Tcells	4.35	0.55	4.41	4.39
ADRA2B	Alpha-2b adrenergic receptor	7.958607315	7.920818754	CD56+_NKCells	4.75	1.16	9.23	9.19
ADRA2B	Alpha-2b adrenergic receptor	7.958607315	7.920818754	CD71+_EarlyErythroid	3.80	-0.28	-2.22	-2.21
ADRA2B	Alpha-2b adrenergic receptor	7.958607315	7.920818754	CD8+_Tcells	3.90	-0.13	-1.01	-1.01
ADRA2B	Alpha-2b adrenergic receptor	7.958607315	7.920818754	Cerebellum	3.20	-1.19	-9.45	-9.40
ADRA2B	Alpha-2b adrenergic receptor	7.958607315	7.920818754	CerebellumPeduncles	4.55	0.86	6.82	6.79
ADRA2B	Alpha-2b adrenergic receptor	7.958607315	7.920818754	CiliaryGanglion	2.85	-1.72	-13.67	-13.60
ADRA2B	Alpha-2b adrenergic receptor	7.958607315	7.920818754	CingulateCortex	4.10	0.18	1.40	1.39
ADRA2B	Alpha-2b adrenergic receptor	7.958607315	7.920818754	Colon	4.05	0.10	0.80	0.79
ADRA2B	Alpha-2b adrenergic receptor	7.958607315	7.920818754	DorsalRootGanglion	3.10	-1.34	-10.65	-10.60
ADRA2B	Alpha-2b adrenergic receptor	7.958607315	7.920818754	Fetalbrain	3.95	-0.05	-0.41	-0.41
ADRA2B	Alpha-2b adrenergic receptor	7.958607315	7.920818754	Fetalliver	3.45	-0.81	-6.44	-6.41
ADRA2B	Alpha-2b adrenergic receptor	7.958607315	7.920818754	Fetallung	3.30	-1.04	-8.24	-8.20
ADRA2B	Alpha-2b adrenergic receptor	7.958607315	7.920818754	FetalThyroid	3.90	-0.13	-1.01	-1.01
ADRA2B	Alpha-2b adrenergic receptor	7.958607315	7.920818754	GlobusPallidus	2.95	-1.57	-12.46	-12.40
ADRA2B	Alpha-2b adrenergic receptor	7.958607315	7.920818754	Heart	5.10	1.69	13.45	13.39
ADRA2B	Alpha-2b adrenergic receptor	7.958607315	7.920818754	Hypothalamus	4.35	0.55	4.41	4.39
ADRA2B	Alpha-2b adrenergic receptor	7.958607315	7.920818754	Kidney	3.25	-1.11	-8.85	-8.80
ADRA2B	Alpha-2b adrenergic receptor	7.958607315	7.920818754	Liver	5.25	1.92	15.26	15.19
ADRA2B	Alpha-2b adrenergic receptor	7.958607315	7.920818754	Lung	4.40	0.63	5.01	4.99
ADRA2B	Alpha-2b adrenergic receptor	7.958607315	7.920818754	Lymphnode	3.50	-0.73	-5.83	-5.81
ADRA2B	Alpha-2b adrenergic receptor	7.958607315	7.920818754	MedullaOblongata	3.65	-0.51	-4.03	-4.01
ADRA2B	Alpha-2b adrenergic receptor	7.958607315	7.920818754	OccipitalLobe	3.60	-0.58	-4.63	-4.61

S2 Table. Target-tissue analysis for clozapine and chlorpromazine

Gene Symbol	Gene Name	Affinity (-log10(Ki))		Tissue	Expression		Combined Score (-log10(Ki)*Z)	
		clozapine	chlorpromazine		Raw-numbers	Z-score	clozapine	chlorpromazine
ADRA2B	Alpha-2b adrenergic receptor	7.958607315	7.920818754	OlfactoryBulb	3.15	-1.26	-10.05	-10.00
ADRA2B	Alpha-2b adrenergic receptor	7.958607315	7.920818754	Ovary	2.60	-2.10	-16.68	-16.60
ADRA2B	Alpha-2b adrenergic receptor	7.958607315	7.920818754	Pancreas	3.20	-1.19	-9.45	-9.40
ADRA2B	Alpha-2b adrenergic receptor	7.958607315	7.920818754	PancreaticIslet	4.25	0.40	3.21	3.19
ADRA2B	Alpha-2b adrenergic receptor	7.958607315	7.920818754	ParietalLobe	4.20	0.33	2.60	2.59
ADRA2B	Alpha-2b adrenergic receptor	7.958607315	7.920818754	Pineal_day	5.00	1.54	12.24	12.19
ADRA2B	Alpha-2b adrenergic receptor	7.958607315	7.920818754	Pineal_night	4.92	1.42	11.28	11.23
ADRA2B	Alpha-2b adrenergic receptor	7.958607315	7.920818754	Pituitary	4.65	1.01	8.03	7.99
ADRA2B	Alpha-2b adrenergic receptor	7.958607315	7.920818754	Placenta	4.10	0.18	1.40	1.39
ADRA2B	Alpha-2b adrenergic receptor	7.958607315	7.920818754	Pons	3.80	-0.28	-2.22	-2.21
ADRA2B	Alpha-2b adrenergic receptor	7.958607315	7.920818754	PrefrontalCortex	5.00	1.54	12.24	12.19
ADRA2B	Alpha-2b adrenergic receptor	7.958607315	7.920818754	Prostate	4.65	1.01	8.03	7.99
ADRA2B	Alpha-2b adrenergic receptor	7.958607315	7.920818754	Retina	4.85	1.31	10.44	10.39
ADRA2B	Alpha-2b adrenergic receptor	7.958607315	7.920818754	Salivarygland	3.25	-1.11	-8.85	-8.80
ADRA2B	Alpha-2b adrenergic receptor	7.958607315	7.920818754	SkeletalMuscle	4.70	1.08	8.63	8.59
ADRA2B	Alpha-2b adrenergic receptor	7.958607315	7.920818754	Skin	3.05	-1.41	-11.26	-11.20
ADRA2B	Alpha-2b adrenergic receptor	7.958607315	7.920818754	Small_intestine	3.85	-0.20	-1.62	-1.61
ADRA2B	Alpha-2b adrenergic receptor	7.958607315	7.920818754	SmoothMuscle	4.55	0.86	6.82	6.79
ADRA2B	Alpha-2b adrenergic receptor	7.958607315	7.920818754	Spinalcord	4.30	0.48	3.81	3.79
ADRA2B	Alpha-2b adrenergic receptor	7.958607315	7.920818754	SubthalamicNucleus	3.65	-0.51	-4.03	-4.01
ADRA2B	Alpha-2b adrenergic receptor	7.958607315	7.920818754	SuperiorCervicalGanglion	4.70	1.08	8.63	8.59
ADRA2B	Alpha-2b adrenergic receptor	7.958607315	7.920818754	TemporalLobe	3.65	-0.51	-4.03	-4.01
ADRA2B	Alpha-2b adrenergic receptor	7.958607315	7.920818754	Testis	3.40	-0.88	-7.04	-7.01
ADRA2B	Alpha-2b adrenergic receptor	7.958607315	7.920818754	TestisGermCell	3.35	-0.96	-7.64	-7.61
ADRA2B	Alpha-2b adrenergic receptor	7.958607315	7.920818754	TestisIntersitial	3.40	-0.88	-7.04	-7.01
ADRA2B	Alpha-2b adrenergic receptor	7.958607315	7.920818754	TestisLeydigCell	4.00	0.02	0.19	0.19
ADRA2B	Alpha-2b adrenergic receptor	7.958607315	7.920818754	TestisSeminiferousTubule	3.40	-0.88	-7.04	-7.01
ADRA2B	Alpha-2b adrenergic receptor	7.958607315	7.920818754	Thalamus	4.05	0.10	0.80	0.79
ADRA2B	Alpha-2b adrenergic receptor	7.958607315	7.920818754	Thymus	3.10	-1.34	-10.65	-10.60
ADRA2B	Alpha-2b adrenergic receptor	7.958607315	7.920818754	Thyroid	5.00	1.54	12.24	12.19
ADRA2B	Alpha-2b adrenergic receptor	7.958607315	7.920818754	Tongue	3.95	-0.05	-0.41	-0.41
ADRA2B	Alpha-2b adrenergic receptor	7.958607315	7.920818754	Tonsil	3.90	-0.13	-1.01	-1.01
ADRA2B	Alpha-2b adrenergic receptor	7.958607315	7.920818754	Trachea	3.30	-1.04	-8.24	-8.20
ADRA2B	Alpha-2b adrenergic receptor	7.958607315	7.920818754	TrigeminalGanglion	5.00	1.54	12.24	12.19
ADRA2B	Alpha-2b adrenergic receptor	7.958607315	7.920818754	Uterus	3.30	-1.04	-8.24	-8.20
ADRA2B	Alpha-2b adrenergic receptor	7.958607315	7.920818754	UterusCorpus	3.55	-0.66	-5.23	-5.21
ADRA2B	Alpha-2b adrenergic receptor	7.958607315	7.920818754	WholeBlood	4.35	0.55	4.41	4.39
ADRA2B	Alpha-2b adrenergic receptor	7.958607315	7.920818754	Wholebrain	3.35	-0.96	-7.64	-7.61
ADRA2C	Alpha-2c adrenergic receptor	8.943095149	7.26760624	Adipocyte	28.40	-0.23	-2.03	-1.65
ADRA2C	Alpha-2c adrenergic receptor	8.943095149	7.26760624	AdrenalCortex	34.65	0.49	4.36	3.55
ADRA2C	Alpha-2c adrenergic receptor	8.943095149	7.26760624	Adrenalgland	24.80	-0.64	-5.71	-4.64
ADRA2C	Alpha-2c adrenergic receptor	8.943095149	7.26760624	Amygdala	30.30	-0.01	-0.08	-0.07
ADRA2C	Alpha-2c adrenergic receptor	8.943095149	7.26760624	Appendix	31.35	0.11	0.99	0.80
ADRA2C	Alpha-2c adrenergic receptor	8.943095149	7.26760624	AtrioventricularNode	27.80	-0.30	-2.64	-2.15
ADRA2C	Alpha-2c adrenergic receptor	8.943095149	7.26760624	BDCA4+_DentriticCells	17.60	-1.46	-13.07	-10.62
ADRA2C	Alpha-2c adrenergic receptor	8.943095149	7.26760624	Bonemarrow	32.45	0.24	2.11	1.72
ADRA2C	Alpha-2c adrenergic receptor	8.943095149	7.26760624	BronchialEpithelialCells	25.65	-0.54	-4.84	-3.93
ADRA2C	Alpha-2c adrenergic receptor	8.943095149	7.26760624	CardiacMyocytes	50.55	2.31	20.62	16.75
ADRA2C	Alpha-2c adrenergic receptor	8.943095149	7.26760624	Caudatenucleus	40.15	1.12	9.98	8.11
ADRA2C	Alpha-2c adrenergic receptor	8.943095149	7.26760624	CD105+_Endothelial	27.10	-0.38	-3.36	-2.73
ADRA2C	Alpha-2c adrenergic receptor	8.943095149	7.26760624	CD14+_Monocytes	28.80	-0.18	-1.62	-1.31
ADRA2C	Alpha-2c adrenergic receptor	8.943095149	7.26760624	CD19+_BCells(neg._sel.)	27.85	-0.29	-2.59	-2.10
ADRA2C	Alpha-2c adrenergic receptor	8.943095149	7.26760624	CD33+_Myeloid	35.20	0.55	4.92	4.00

S2 Table. Target-tissue analysis for clozapine and chlorpromazine

Gene Symbol	Gene Name	Affinity (-log10(Ki))		Tissue	Expression		Combined Score (-log10(Ki)*Z)	
		clozapine	chlorpromazine		Raw-numbers	Z-score	clozapine	chlorpromazine
ADRA2C	Alpha-2c adrenergic receptor	8.943095149	7.26760624	CD34+	27.55	-0.32	-2.90	-2.35
ADRA2C	Alpha-2c adrenergic receptor	8.943095149	7.26760624	CD4+_Tcells	30.35	0.00	-0.03	-0.03
ADRA2C	Alpha-2c adrenergic receptor	8.943095149	7.26760624	CD56+_NKCells	23.10	-0.83	-7.44	-6.05
ADRA2C	Alpha-2c adrenergic receptor	8.943095149	7.26760624	CD71+_EarlyErythroid	26.90	-0.40	-3.56	-2.89
ADRA2C	Alpha-2c adrenergic receptor	8.943095149	7.26760624	CD8+_Tcells	24.25	-0.70	-6.27	-5.09
ADRA2C	Alpha-2c adrenergic receptor	8.943095149	7.26760624	Cerebellum	23.05	-0.84	-7.50	-6.09
ADRA2C	Alpha-2c adrenergic receptor	8.943095149	7.26760624	CerebellumPeduncles	40.40	1.15	10.24	8.32
ADRA2C	Alpha-2c adrenergic receptor	8.943095149	7.26760624	CiliaryGanglion	24.00	-0.73	-6.52	-5.30
ADRA2C	Alpha-2c adrenergic receptor	8.943095149	7.26760624	CingulateCortex	35.10	0.54	4.82	3.92
ADRA2C	Alpha-2c adrenergic receptor	8.943095149	7.26760624	Colon	28.60	-0.20	-1.82	-1.48
ADRA2C	Alpha-2c adrenergic receptor	8.943095149	7.26760624	DorsalRootGanglion	22.45	-0.91	-8.11	-6.59
ADRA2C	Alpha-2c adrenergic receptor	8.943095149	7.26760624	Fetalbrain	27.50	-0.33	-2.95	-2.39
ADRA2C	Alpha-2c adrenergic receptor	8.943095149	7.26760624	Fetalliver	24.70	-0.65	-5.81	-4.72
ADRA2C	Alpha-2c adrenergic receptor	8.943095149	7.26760624	Fetallung	16.55	-1.58	-14.14	-11.49
ADRA2C	Alpha-2c adrenergic receptor	8.943095149	7.26760624	FetalThyroid	27.10	-0.38	-3.36	-2.73
ADRA2C	Alpha-2c adrenergic receptor	8.943095149	7.26760624	GlobusPallidus	34.45	0.46	4.16	3.38
ADRA2C	Alpha-2c adrenergic receptor	8.943095149	7.26760624	Heart	58.75	3.24	29.00	23.57
ADRA2C	Alpha-2c adrenergic receptor	8.943095149	7.26760624	Hypothalamus	27.55	-0.32	-2.90	-2.35
ADRA2C	Alpha-2c adrenergic receptor	8.943095149	7.26760624	Kidney	31.25	0.10	0.89	0.72
ADRA2C	Alpha-2c adrenergic receptor	8.943095149	7.26760624	Liver	38.15	0.89	7.94	6.45
ADRA2C	Alpha-2c adrenergic receptor	8.943095149	7.26760624	Lung	31.75	0.16	1.40	1.14
ADRA2C	Alpha-2c adrenergic receptor	8.943095149	7.26760624	Lymphnode	23.40	-0.80	-7.14	-5.80
ADRA2C	Alpha-2c adrenergic receptor	8.943095149	7.26760624	MedullaOblongata	26.30	-0.47	-4.17	-3.39
ADRA2C	Alpha-2c adrenergic receptor	8.943095149	7.26760624	OccipitalLobe	26.80	-0.41	-3.66	-2.98
ADRA2C	Alpha-2c adrenergic receptor	8.943095149	7.26760624	OlfactoryBulb	22.25	-0.93	-8.31	-6.76
ADRA2C	Alpha-2c adrenergic receptor	8.943095149	7.26760624	Ovary	20.45	-1.14	-10.15	-8.25
ADRA2C	Alpha-2c adrenergic receptor	8.943095149	7.26760624	Pancreas	24.80	-0.64	-5.71	-4.64
ADRA2C	Alpha-2c adrenergic receptor	8.943095149	7.26760624	PancreaticIslet	30.65	0.03	0.27	0.22
ADRA2C	Alpha-2c adrenergic receptor	8.943095149	7.26760624	ParietalLobe	29.95	-0.05	-0.44	-0.36
ADRA2C	Alpha-2c adrenergic receptor	8.943095149	7.26760624	Pineal_day	28.84	-0.18	-1.58	-1.28
ADRA2C	Alpha-2c adrenergic receptor	8.943095149	7.26760624	Pineal_night	31.02	0.07	0.65	0.53
ADRA2C	Alpha-2c adrenergic receptor	8.943095149	7.26760624	Pituitary	33.50	0.36	3.19	2.59
ADRA2C	Alpha-2c adrenergic receptor	8.943095149	7.26760624	Placenta	29.85	-0.06	-0.54	-0.44
ADRA2C	Alpha-2c adrenergic receptor	8.943095149	7.26760624	Pons	39.40	1.03	9.22	7.49
ADRA2C	Alpha-2c adrenergic receptor	8.943095149	7.26760624	PrefrontalCortex	34.15	0.43	3.85	3.13
ADRA2C	Alpha-2c adrenergic receptor	8.943095149	7.26760624	Prostate	33.75	0.38	3.44	2.80
ADRA2C	Alpha-2c adrenergic receptor	8.943095149	7.26760624	Retina	34.78	0.50	4.49	3.65
ADRA2C	Alpha-2c adrenergic receptor	8.943095149	7.26760624	Salivarygland	22.85	-0.86	-7.70	-6.26
ADRA2C	Alpha-2c adrenergic receptor	8.943095149	7.26760624	SkeletalMuscle	65.55	4.02	35.95	29.21
ADRA2C	Alpha-2c adrenergic receptor	8.943095149	7.26760624	Skin	30.85	0.05	0.48	0.39
ADRA2C	Alpha-2c adrenergic receptor	8.943095149	7.26760624	Small_intestine	27.80	-0.30	-2.64	-2.15
ADRA2C	Alpha-2c adrenergic receptor	8.943095149	7.26760624	SmoothMuscle	26.75	-0.42	-3.71	-3.02
ADRA2C	Alpha-2c adrenergic receptor	8.943095149	7.26760624	Spinalcord	29.25	-0.13	-1.16	-0.94
ADRA2C	Alpha-2c adrenergic receptor	8.943095149	7.26760624	SubthalamicNucleus	26.05	-0.50	-4.43	-3.60
ADRA2C	Alpha-2c adrenergic receptor	8.943095149	7.26760624	SuperiorCervicalGanglion	42.90	1.43	12.80	10.40
ADRA2C	Alpha-2c adrenergic receptor	8.943095149	7.26760624	TemporalLobe	28.40	-0.23	-2.03	-1.65
ADRA2C	Alpha-2c adrenergic receptor	8.943095149	7.26760624	Testis	24.70	-0.65	-5.81	-4.72
ADRA2C	Alpha-2c adrenergic receptor	8.943095149	7.26760624	TestisGermCell	23.85	-0.75	-6.68	-5.43
ADRA2C	Alpha-2c adrenergic receptor	8.943095149	7.26760624	TestisIntersitial	23.65	-0.77	-6.88	-5.59
ADRA2C	Alpha-2c adrenergic receptor	8.943095149	7.26760624	TestisLeydigCell	31.05	0.08	0.68	0.55
ADRA2C	Alpha-2c adrenergic receptor	8.943095149	7.26760624	TestisSeminiferousTubule	24.05	-0.72	-6.47	-5.26
ADRA2C	Alpha-2c adrenergic receptor	8.943095149	7.26760624	Thalamus	33.45	0.35	3.14	2.55
ADRA2C	Alpha-2c adrenergic receptor	8.943095149	7.26760624	Thymus	19.80	-1.21	-10.82	-8.79

S2 Table. Target-tissue analysis for clozapine and chlorpromazine

Gene Symbol	Gene Name	Affinity (-log10(Ki))		Tissue	Expression		Combined Score (-log10(Ki)*Z)	
		clozapine	chlorpromazine		Raw-numbers	Z-score	clozapine	chlorpromazine
ADRA2C	Alpha-2c adrenergic receptor	8.943095149	7.26760624	Thyroid	28.90	-0.17	-1.52	-1.23
ADRA2C	Alpha-2c adrenergic receptor	8.943095149	7.26760624	Tongue	40.20	1.12	10.04	8.16
ADRA2C	Alpha-2c adrenergic receptor	8.943095149	7.26760624	Tonsil	27.20	-0.36	-3.25	-2.64
ADRA2C	Alpha-2c adrenergic receptor	8.943095149	7.26760624	Trachea	24.80	-0.64	-5.71	-4.64
ADRA2C	Alpha-2c adrenergic receptor	8.943095149	7.26760624	TrigeminalGanglion	39.45	1.04	9.27	7.53
ADRA2C	Alpha-2c adrenergic receptor	8.943095149	7.26760624	Uterus	56.20	2.95	26.39	21.45
ADRA2C	Alpha-2c adrenergic receptor	8.943095149	7.26760624	UterusCorpus	44.80	1.65	14.74	11.98
ADRA2C	Alpha-2c adrenergic receptor	8.943095149	7.26760624	WholeBlood	18.40	-1.37	-12.25	-9.95
ADRA2C	Alpha-2c adrenergic receptor	8.943095149	7.26760624	Wholebrain	22.50	-0.90	-8.06	-6.55
ADRB1	Beta-1 adrenergic receptor	4.585026652	n/a	Adipocyte	9.45	0.15	0.71	n/a
ADRB1	Beta-1 adrenergic receptor	4.585026652	n/a	AdrenalCortex	11.95	0.80	3.65	n/a
ADRB1	Beta-1 adrenergic receptor	4.585026652	n/a	Adrenalgland	7.00	-0.47	-2.17	n/a
ADRB1	Beta-1 adrenergic receptor	4.585026652	n/a	Amygdala	7.95	-0.23	-1.06	n/a
ADRB1	Beta-1 adrenergic receptor	4.585026652	n/a	Appendix	9.80	0.24	1.12	n/a
ADRB1	Beta-1 adrenergic receptor	4.585026652	n/a	AtrioventricularNode	8.55	-0.08	-0.35	n/a
ADRB1	Beta-1 adrenergic receptor	4.585026652	n/a	BDCA4+_DentriticCells	7.65	-0.31	-1.41	n/a
ADRB1	Beta-1 adrenergic receptor	4.585026652	n/a	Bonemarrow	8.35	-0.13	-0.59	n/a
ADRB1	Beta-1 adrenergic receptor	4.585026652	n/a	BronchialEpithelialCells	7.70	-0.29	-1.35	n/a
ADRB1	Beta-1 adrenergic receptor	4.585026652	n/a	CardiacMyocytes	11.70	0.73	3.35	n/a
ADRB1	Beta-1 adrenergic receptor	4.585026652	n/a	Caudatenucleus	7.05	-0.46	-2.11	n/a
ADRB1	Beta-1 adrenergic receptor	4.585026652	n/a	CD105+_Endothelial	7.55	-0.33	-1.53	n/a
ADRB1	Beta-1 adrenergic receptor	4.585026652	n/a	CD14+_Monocytes	8.20	-0.17	-0.76	n/a
ADRB1	Beta-1 adrenergic receptor	4.585026652	n/a	CD19+_BCells(neg._sel.)	8.10	-0.19	-0.88	n/a
ADRB1	Beta-1 adrenergic receptor	4.585026652	n/a	CD33+_Myeloid	9.20	0.09	0.41	n/a
ADRB1	Beta-1 adrenergic receptor	4.585026652	n/a	CD34+	9.60	0.19	0.88	n/a
ADRB1	Beta-1 adrenergic receptor	4.585026652	n/a	CD4+_Tcells	8.20	-0.17	-0.76	n/a
ADRB1	Beta-1 adrenergic receptor	4.585026652	n/a	CD56+_NKCells	8.55	-0.08	-0.35	n/a
ADRB1	Beta-1 adrenergic receptor	4.585026652	n/a	CD71+_EarlyErythroid	7.50	-0.35	-1.59	n/a
ADRB1	Beta-1 adrenergic receptor	4.585026652	n/a	CD8+_Tcells	7.20	-0.42	-1.94	n/a
ADRB1	Beta-1 adrenergic receptor	4.585026652	n/a	Cerebellum	6.55	-0.59	-2.70	n/a
ADRB1	Beta-1 adrenergic receptor	4.585026652	n/a	CerebellumPeduncles	9.75	0.23	1.06	n/a
ADRB1	Beta-1 adrenergic receptor	4.585026652	n/a	CiliaryGanglion	6.80	-0.53	-2.41	n/a
ADRB1	Beta-1 adrenergic receptor	4.585026652	n/a	CingulateCortex	9.40	0.14	0.65	n/a
ADRB1	Beta-1 adrenergic receptor	4.585026652	n/a	Colon	7.50	-0.35	-1.59	n/a
ADRB1	Beta-1 adrenergic receptor	4.585026652	n/a	DorsalRootGanglion	7.10	-0.45	-2.06	n/a
ADRB1	Beta-1 adrenergic receptor	4.585026652	n/a	Fetalbrain	7.65	-0.31	-1.41	n/a
ADRB1	Beta-1 adrenergic receptor	4.585026652	n/a	Fetalliver	6.85	-0.51	-2.35	n/a
ADRB1	Beta-1 adrenergic receptor	4.585026652	n/a	Fetallung	5.45	-0.87	-4.00	n/a
ADRB1	Beta-1 adrenergic receptor	4.585026652	n/a	FetalThyroid	7.75	-0.28	-1.29	n/a
ADRB1	Beta-1 adrenergic receptor	4.585026652	n/a	GlobusPallidus	7.25	-0.41	-1.88	n/a
ADRB1	Beta-1 adrenergic receptor	4.585026652	n/a	Heart	12.30	0.89	4.06	n/a
ADRB1	Beta-1 adrenergic receptor	4.585026652	n/a	Hypothalamus	8.40	-0.11	-0.53	n/a
ADRB1	Beta-1 adrenergic receptor	4.585026652	n/a	Kidney	7.55	-0.33	-1.53	n/a
ADRB1	Beta-1 adrenergic receptor	4.585026652	n/a	Liver	13.15	1.10	5.06	n/a
ADRB1	Beta-1 adrenergic receptor	4.585026652	n/a	Lung	12.00	0.81	3.71	n/a
ADRB1	Beta-1 adrenergic receptor	4.585026652	n/a	Lymphnode	6.40	-0.63	-2.88	n/a
ADRB1	Beta-1 adrenergic receptor	4.585026652	n/a	MedullaOblongata	7.20	-0.42	-1.94	n/a
ADRB1	Beta-1 adrenergic receptor	4.585026652	n/a	OccipitalLobe	7.05	-0.46	-2.11	n/a
ADRB1	Beta-1 adrenergic receptor	4.585026652	n/a	OlfactoryBulb	6.10	-0.70	-3.23	n/a
ADRB1	Beta-1 adrenergic receptor	4.585026652	n/a	Ovary	5.65	-0.82	-3.76	n/a
ADRB1	Beta-1 adrenergic receptor	4.585026652	n/a	Pancreas	6.55	-0.59	-2.70	n/a
ADRB1	Beta-1 adrenergic receptor	4.585026652	n/a	PancreaticIslet	8.50	-0.09	-0.41	n/a
ADRB1	Beta-1 adrenergic receptor	4.585026652	n/a	ParietalLobe	10.55	0.44	2.00	n/a

S2 Table. Target-tissue analysis for clozapine and chlorpromazine

Gene Symbol	Gene Name	Affinity (-log10(Ki))		Tissue	Expression		Combined Score (-log10(Ki)*Z)	
		clozapine	chlorpromazine		Raw-numbers	Z-score	clozapine	chlorpromazine
ADRB1	Beta-1 adrenergic receptor	4.585026652	n/a	Pineal_day	24.52	4.02	18.44	n/a
ADRB1	Beta-1 adrenergic receptor	4.585026652	n/a	Pineal_night	34.22	6.51	29.85	n/a
ADRB1	Beta-1 adrenergic receptor	4.585026652	n/a	Pituitary	9.45	0.15	0.71	n/a
ADRB1	Beta-1 adrenergic receptor	4.585026652	n/a	Placenta	7.95	-0.23	-1.06	n/a
ADRB1	Beta-1 adrenergic receptor	4.585026652	n/a	Pons	8.75	-0.03	-0.12	n/a
ADRB1	Beta-1 adrenergic receptor	4.585026652	n/a	PrefrontalCortex	9.15	0.08	0.36	n/a
ADRB1	Beta-1 adrenergic receptor	4.585026652	n/a	Prostate	9.05	0.05	0.24	n/a
ADRB1	Beta-1 adrenergic receptor	4.585026652	n/a	Retina	9.40	0.14	0.65	n/a
ADRB1	Beta-1 adrenergic receptor	4.585026652	n/a	Salivarygland	6.90	-0.50	-2.29	n/a
ADRB1	Beta-1 adrenergic receptor	4.585026652	n/a	SkeletalMuscle	14.25	1.39	6.35	n/a
ADRB1	Beta-1 adrenergic receptor	4.585026652	n/a	Skin	6.75	-0.54	-2.47	n/a
ADRB1	Beta-1 adrenergic receptor	4.585026652	n/a	Small_intestine	7.20	-0.42	-1.94	n/a
ADRB1	Beta-1 adrenergic receptor	4.585026652	n/a	SmoothMuscle	8.75	-0.03	-0.12	n/a
ADRB1	Beta-1 adrenergic receptor	4.585026652	n/a	Spinalcord	8.40	-0.11	-0.53	n/a
ADRB1	Beta-1 adrenergic receptor	4.585026652	n/a	SubthalamicNucleus	9.20	0.09	0.41	n/a
ADRB1	Beta-1 adrenergic receptor	4.585026652	n/a	SuperiorCervicalGanglion	12.45	0.92	4.24	n/a
ADRB1	Beta-1 adrenergic receptor	4.585026652	n/a	TemporalLobe	7.45	-0.36	-1.64	n/a
ADRB1	Beta-1 adrenergic receptor	4.585026652	n/a	Testis	6.70	-0.55	-2.53	n/a
ADRB1	Beta-1 adrenergic receptor	4.585026652	n/a	TestisGermCell	6.35	-0.64	-2.94	n/a
ADRB1	Beta-1 adrenergic receptor	4.585026652	n/a	TestisIntersitial	6.70	-0.55	-2.53	n/a
ADRB1	Beta-1 adrenergic receptor	4.585026652	n/a	TestisLeydigCell	8.35	-0.13	-0.59	n/a
ADRB1	Beta-1 adrenergic receptor	4.585026652	n/a	TestisSeminiferousTubule	6.75	-0.54	-2.47	n/a
ADRB1	Beta-1 adrenergic receptor	4.585026652	n/a	Thalamus	8.25	-0.15	-0.70	n/a
ADRB1	Beta-1 adrenergic receptor	4.585026652	n/a	Thymus	5.80	-0.78	-3.59	n/a
ADRB1	Beta-1 adrenergic receptor	4.585026652	n/a	Thyroid	8.75	-0.03	-0.12	n/a
ADRB1	Beta-1 adrenergic receptor	4.585026652	n/a	Tongue	9.95	0.28	1.30	n/a
ADRB1	Beta-1 adrenergic receptor	4.585026652	n/a	Tonsil	7.70	-0.29	-1.35	n/a
ADRB1	Beta-1 adrenergic receptor	4.585026652	n/a	Trachea	6.60	-0.58	-2.64	n/a
ADRB1	Beta-1 adrenergic receptor	4.585026652	n/a	TrigeminalGanglion	10.05	0.31	1.41	n/a
ADRB1	Beta-1 adrenergic receptor	4.585026652	n/a	Uterus	6.00	-0.73	-3.35	n/a
ADRB1	Beta-1 adrenergic receptor	4.585026652	n/a	UterusCorpus	9.95	0.28	1.30	n/a
ADRB1	Beta-1 adrenergic receptor	4.585026652	n/a	WholeBlood	8.35	-0.13	-0.59	n/a
ADRB1	Beta-1 adrenergic receptor	4.585026652	n/a	Wholebrain	6.50	-0.60	-2.76	n/a
AOX1	Aldehyde oxidase	n/a	5.638272164	Adipocyte	313.93	5.03	n/a	28.35
AOX1	Aldehyde oxidase	n/a	5.638272164	AdrenalCortex	355.33	5.74	n/a	32.34
AOX1	Aldehyde oxidase	n/a	5.638272164	Adrenalgland	231.38	3.62	n/a	20.39
AOX1	Aldehyde oxidase	n/a	5.638272164	Amygdala	6.25	-0.23	n/a	-1.32
AOX1	Aldehyde oxidase	n/a	5.638272164	Appendix	7.73	-0.21	n/a	-1.18
AOX1	Aldehyde oxidase	n/a	5.638272164	AtrioventricularNode	5.40	-0.25	n/a	-1.40
AOX1	Aldehyde oxidase	n/a	5.638272164	BDCA4+_DentriticCells	6.18	-0.24	n/a	-1.33
AOX1	Aldehyde oxidase	n/a	5.638272164	Bonemarrow	7.10	-0.22	n/a	-1.24
AOX1	Aldehyde oxidase	n/a	5.638272164	BronchialEpithelialCells	6.38	-0.23	n/a	-1.31
AOX1	Aldehyde oxidase	n/a	5.638272164	CardiacMyocytes	7.45	-0.21	n/a	-1.21
AOX1	Aldehyde oxidase	n/a	5.638272164	Caudatenucleus	5.43	-0.25	n/a	-1.40
AOX1	Aldehyde oxidase	n/a	5.638272164	CD105+_Endothelial	5.73	-0.24	n/a	-1.37
AOX1	Aldehyde oxidase	n/a	5.638272164	CD14+_Monocytes	6.08	-0.24	n/a	-1.34
AOX1	Aldehyde oxidase	n/a	5.638272164	CD19+_BCells(neg._sel.)	5.78	-0.24	n/a	-1.37
AOX1	Aldehyde oxidase	n/a	5.638272164	CD33+_Myeloid	7.48	-0.21	n/a	-1.20
AOX1	Aldehyde oxidase	n/a	5.638272164	CD34+	7.08	-0.22	n/a	-1.24
AOX1	Aldehyde oxidase	n/a	5.638272164	CD4+_Tcells	6.13	-0.24	n/a	-1.33
AOX1	Aldehyde oxidase	n/a	5.638272164	CD56+_NKCells	6.55	-0.23	n/a	-1.29
AOX1	Aldehyde oxidase	n/a	5.638272164	CD71+_EarlyErythroid	5.63	-0.25	n/a	-1.38
AOX1	Aldehyde oxidase	n/a	5.638272164	CD8+_Tcells	5.45	-0.25	n/a	-1.40

S2 Table. Target-tissue analysis for clozapine and chlorpromazine

Gene Symbol	Gene Name	Affinity (-log10(Ki))		Tissue	Expression		Combined Score (-log10(Ki)*Z)	
		clozapine	chlorpromazine		Raw-numbers	Z-score	clozapine	chlorpromazine
AOX1	Aldehyde oxidase	n/a	5.638272164	Cerebellum	5.15	-0.25	n/a	-1.43
AOX1	Aldehyde oxidase	n/a	5.638272164	CerebellumPeduncles	7.43	-0.21	n/a	-1.21
AOX1	Aldehyde oxidase	n/a	5.638272164	CiliaryGanglion	6.83	-0.22	n/a	-1.27
AOX1	Aldehyde oxidase	n/a	5.638272164	CingulateCortex	6.80	-0.23	n/a	-1.27
AOX1	Aldehyde oxidase	n/a	5.638272164	Colon	5.80	-0.24	n/a	-1.37
AOX1	Aldehyde oxidase	n/a	5.638272164	DorsalRootGanglion	4.80	-0.26	n/a	-1.46
AOX1	Aldehyde oxidase	n/a	5.638272164	Fetalbrain	6.10	-0.24	n/a	-1.34
AOX1	Aldehyde oxidase	n/a	5.638272164	Fetalliver	5.90	-0.24	n/a	-1.36
AOX1	Aldehyde oxidase	n/a	5.638272164	Fetallung	4.80	-0.26	n/a	-1.46
AOX1	Aldehyde oxidase	n/a	5.638272164	FetalThyroid	6.13	-0.24	n/a	-1.33
AOX1	Aldehyde oxidase	n/a	5.638272164	GlobusPallidus	5.73	-0.24	n/a	-1.37
AOX1	Aldehyde oxidase	n/a	5.638272164	Heart	7.70	-0.21	n/a	-1.18
AOX1	Aldehyde oxidase	n/a	5.638272164	Hypothalamus	6.38	-0.23	n/a	-1.31
AOX1	Aldehyde oxidase	n/a	5.638272164	Kidney	66.23	0.79	n/a	4.46
AOX1	Aldehyde oxidase	n/a	5.638272164	Liver	28.08	0.14	n/a	0.78
AOX1	Aldehyde oxidase	n/a	5.638272164	Lung	6.50	-0.23	n/a	-1.30
AOX1	Aldehyde oxidase	n/a	5.638272164	Lymphnode	5.28	-0.25	n/a	-1.42
AOX1	Aldehyde oxidase	n/a	5.638272164	MedullaOblongata	7.40	-0.21	n/a	-1.21
AOX1	Aldehyde oxidase	n/a	5.638272164	OccipitalLobe	5.48	-0.25	n/a	-1.40
AOX1	Aldehyde oxidase	n/a	5.638272164	OlfactoryBulb	4.73	-0.26	n/a	-1.47
AOX1	Aldehyde oxidase	n/a	5.638272164	Ovary	9.28	-0.18	n/a	-1.03
AOX1	Aldehyde oxidase	n/a	5.638272164	Pancreas	13.70	-0.11	n/a	-0.60
AOX1	Aldehyde oxidase	n/a	5.638272164	PancreaticIslet	58.75	0.66	n/a	3.74
AOX1	Aldehyde oxidase	n/a	5.638272164	ParietalLobe	6.43	-0.23	n/a	-1.31
AOX1	Aldehyde oxidase	n/a	5.638272164	Pineal_day	7.74	-0.21	n/a	-1.18
AOX1	Aldehyde oxidase	n/a	5.638272164	Pineal_night	8.17	-0.20	n/a	-1.14
AOX1	Aldehyde oxidase	n/a	5.638272164	Pituitary	9.90	-0.17	n/a	-0.97
AOX1	Aldehyde oxidase	n/a	5.638272164	Placenta	5.85	-0.24	n/a	-1.36
AOX1	Aldehyde oxidase	n/a	5.638272164	Pons	9.20	-0.18	n/a	-1.04
AOX1	Aldehyde oxidase	n/a	5.638272164	PrefrontalCortex	7.25	-0.22	n/a	-1.23
AOX1	Aldehyde oxidase	n/a	5.638272164	Prostate	8.55	-0.20	n/a	-1.10
AOX1	Aldehyde oxidase	n/a	5.638272164	Retina	9.51	-0.18	n/a	-1.01
AOX1	Aldehyde oxidase	n/a	5.638272164	Salivarygland	6.98	-0.22	n/a	-1.25
AOX1	Aldehyde oxidase	n/a	5.638272164	SkeletalMuscle	8.35	-0.20	n/a	-1.12
AOX1	Aldehyde oxidase	n/a	5.638272164	Skin	5.65	-0.24	n/a	-1.38
AOX1	Aldehyde oxidase	n/a	5.638272164	Small_intestine	5.28	-0.25	n/a	-1.42
AOX1	Aldehyde oxidase	n/a	5.638272164	SmoothMuscle	7.00	-0.22	n/a	-1.25
AOX1	Aldehyde oxidase	n/a	5.638272164	Spinalcord	6.45	-0.23	n/a	-1.30
AOX1	Aldehyde oxidase	n/a	5.638272164	SubthalamicNucleus	5.73	-0.24	n/a	-1.37
AOX1	Aldehyde oxidase	n/a	5.638272164	SuperiorCervicalGanglion	14.35	-0.10	n/a	-0.54
AOX1	Aldehyde oxidase	n/a	5.638272164	TemporalLobe	6.38	-0.23	n/a	-1.31
AOX1	Aldehyde oxidase	n/a	5.638272164	Testis	6.18	-0.24	n/a	-1.33
AOX1	Aldehyde oxidase	n/a	5.638272164	TestisGermCell	9.65	-0.18	n/a	-0.99
AOX1	Aldehyde oxidase	n/a	5.638272164	TestisIntersitial	5.13	-0.25	n/a	-1.43
AOX1	Aldehyde oxidase	n/a	5.638272164	TestisLeydigCell	6.58	-0.23	n/a	-1.29
AOX1	Aldehyde oxidase	n/a	5.638272164	TestisSeminiferousTubule	8.83	-0.19	n/a	-1.07
AOX1	Aldehyde oxidase	n/a	5.638272164	Thalamus	6.15	-0.24	n/a	-1.33
AOX1	Aldehyde oxidase	n/a	5.638272164	Thymus	4.73	-0.26	n/a	-1.47
AOX1	Aldehyde oxidase	n/a	5.638272164	Thyroid	11.08	-0.15	n/a	-0.86
AOX1	Aldehyde oxidase	n/a	5.638272164	Tongue	6.33	-0.23	n/a	-1.32
AOX1	Aldehyde oxidase	n/a	5.638272164	Tonsil	5.88	-0.24	n/a	-1.36
AOX1	Aldehyde oxidase	n/a	5.638272164	Trachea	5.70	-0.24	n/a	-1.38
AOX1	Aldehyde oxidase	n/a	5.638272164	TrigeminalGanglion	7.48	-0.21	n/a	-1.20

S2 Table. Target-tissue analysis for clozapine and chlorpromazine

Gene Symbol	Gene Name	Affinity (-log10(Ki))		Tissue	Expression		Combined Score (-log10(Ki)*Z)	
		clozapine	chlorpromazine		Raw-numbers	Z-score	clozapine	chlorpromazine
AOX1	Aldehyde oxidase	n/a	5.638272164	Uterus	4.78	-0.26	n/a	-1.46
AOX1	Aldehyde oxidase	n/a	5.638272164	UterusCorpus	5.53	-0.25	n/a	-1.39
AOX1	Aldehyde oxidase	n/a	5.638272164	WholeBlood	6.30	-0.23	n/a	-1.32
AOX1	Aldehyde oxidase	n/a	5.638272164	Wholebrain	4.73	-0.26	n/a	-1.47
CALM1	Calmodulin	n/a	4.71489297	Adipocyte	178.55	-0.64	n/a	-3.00
CALM1	Calmodulin	n/a	4.71489297	AdrenalCortex	77.35	-0.72	n/a	-3.41
CALM1	Calmodulin	n/a	4.71489297	Adrenalgland	110.50	-0.69	n/a	-3.27
CALM1	Calmodulin	n/a	4.71489297	Amygdala	4496.55	3.07	n/a	14.46
CALM1	Calmodulin	n/a	4.71489297	Appendix	156.35	-0.65	n/a	-3.09
CALM1	Calmodulin	n/a	4.71489297	AtrioventricularNode	51.25	-0.75	n/a	-3.51
CALM1	Calmodulin	n/a	4.71489297	BDCA4+_DentriticCells	322.15	-0.51	n/a	-2.42
CALM1	Calmodulin	n/a	4.71489297	Bonemarrow	134.70	-0.67	n/a	-3.18
CALM1	Calmodulin	n/a	4.71489297	BronchialEpithelialCells	201.90	-0.62	n/a	-2.90
CALM1	Calmodulin	n/a	4.71489297	CardiacMyocytes	332.05	-0.50	n/a	-2.38
CALM1	Calmodulin	n/a	4.71489297	Caudatenucleus	1692.95	0.66	n/a	3.13
CALM1	Calmodulin	n/a	4.71489297	CD105+_Endothelial	405.75	-0.44	n/a	-2.08
CALM1	Calmodulin	n/a	4.71489297	CD14+_Monocytes	541.50	-0.32	n/a	-1.53
CALM1	Calmodulin	n/a	4.71489297	CD19+_BCells(neg._sel.)	480.10	-0.38	n/a	-1.78
CALM1	Calmodulin	n/a	4.71489297	CD33+_Myeloid	571.35	-0.30	n/a	-1.41
CALM1	Calmodulin	n/a	4.71489297	CD34+	562.90	-0.31	n/a	-1.44
CALM1	Calmodulin	n/a	4.71489297	CD4+_Tcells	895.60	-0.02	n/a	-0.10
CALM1	Calmodulin	n/a	4.71489297	CD56+_NKCells	1354.10	0.37	n/a	1.76
CALM1	Calmodulin	n/a	4.71489297	CD71+_EarlyErythroid	113.65	-0.69	n/a	-3.26
CALM1	Calmodulin	n/a	4.71489297	CD8+_Tcells	937.45	0.01	n/a	0.07
CALM1	Calmodulin	n/a	4.71489297	Cerebellum	1675.90	0.65	n/a	3.06
CALM1	Calmodulin	n/a	4.71489297	CerebellumPeduncles	1816.70	0.77	n/a	3.63
CALM1	Calmodulin	n/a	4.71489297	CiliaryGanglion	100.20	-0.70	n/a	-3.31
CALM1	Calmodulin	n/a	4.71489297	CingulateCortex	2530.65	1.38	n/a	6.51
CALM1	Calmodulin	n/a	4.71489297	Colon	555.20	-0.31	n/a	-1.48
CALM1	Calmodulin	n/a	4.71489297	DorsalRootGanglion	225.10	-0.60	n/a	-2.81
CALM1	Calmodulin	n/a	4.71489297	Fetalbrain	2437.15	1.30	n/a	6.13
CALM1	Calmodulin	n/a	4.71489297	Fetalliver	122.65	-0.68	n/a	-3.22
CALM1	Calmodulin	n/a	4.71489297	Fetallung	249.80	-0.57	n/a	-2.71
CALM1	Calmodulin	n/a	4.71489297	FetalThyroid	367.05	-0.47	n/a	-2.24
CALM1	Calmodulin	n/a	4.71489297	GlobusPallidus	1582.05	0.57	n/a	2.68
CALM1	Calmodulin	n/a	4.71489297	Heart	216.90	-0.60	n/a	-2.84
CALM1	Calmodulin	n/a	4.71489297	Hypothalamus	1475.10	0.48	n/a	2.24
CALM1	Calmodulin	n/a	4.71489297	Kidney	266.05	-0.56	n/a	-2.64
CALM1	Calmodulin	n/a	4.71489297	Liver	118.80	-0.69	n/a	-3.24
CALM1	Calmodulin	n/a	4.71489297	Lung	149.85	-0.66	n/a	-3.11
CALM1	Calmodulin	n/a	4.71489297	Lymphnode	180.80	-0.63	n/a	-2.99
CALM1	Calmodulin	n/a	4.71489297	MedullaOblongata	2930.20	1.72	n/a	8.13
CALM1	Calmodulin	n/a	4.71489297	OccipitalLobe	4406.45	2.99	n/a	14.10
CALM1	Calmodulin	n/a	4.71489297	OlfactoryBulb	637.70	-0.24	n/a	-1.14
CALM1	Calmodulin	n/a	4.71489297	Ovary	69.30	-0.73	n/a	-3.44
CALM1	Calmodulin	n/a	4.71489297	Pancreas	86.45	-0.71	n/a	-3.37
CALM1	Calmodulin	n/a	4.71489297	PancreaticIslet	429.45	-0.42	n/a	-1.98
CALM1	Calmodulin	n/a	4.71489297	ParietalLobe	3833.35	2.50	n/a	11.78
CALM1	Calmodulin	n/a	4.71489297	Pineal_day	2533.14	1.38	n/a	6.52
CALM1	Calmodulin	n/a	4.71489297	Pineal_night	2230.40	1.12	n/a	5.30
CALM1	Calmodulin	n/a	4.71489297	Pituitary	899.35	-0.02	n/a	-0.08
CALM1	Calmodulin	n/a	4.71489297	Placenta	467.80	-0.39	n/a	-1.83
CALM1	Calmodulin	n/a	4.71489297	Pons	1797.00	0.75	n/a	3.55

S2 Table. Target-tissue analysis for clozapine and chlorpromazine

Gene Symbol	Gene Name	Affinity (-log10(Ki))		Tissue	Expression		Combined Score (-log10(Ki)*Z)	
		clozapine	chlorpromazine		Raw-numbers	Z-score	clozapine	chlorpromazine
CALM1	Calmodulin	n/a	4.71489297	PrefrontalCortex	4954.55	3.46	n/a	16.31
CALM1	Calmodulin	n/a	4.71489297	Prostate	186.10	-0.63	n/a	-2.97
CALM1	Calmodulin	n/a	4.71489297	Retina	1185.93	0.23	n/a	1.08
CALM1	Calmodulin	n/a	4.71489297	Salivarygland	139.30	-0.67	n/a	-3.16
CALM1	Calmodulin	n/a	4.71489297	SkeletalMuscle	124.55	-0.68	n/a	-3.22
CALM1	Calmodulin	n/a	4.71489297	Skin	49.05	-0.75	n/a	-3.52
CALM1	Calmodulin	n/a	4.71489297	Small_intestine	555.45	-0.31	n/a	-1.47
CALM1	Calmodulin	n/a	4.71489297	SmoothMuscle	461.20	-0.39	n/a	-1.86
CALM1	Calmodulin	n/a	4.71489297	Spinalcord	952.80	0.03	n/a	0.13
CALM1	Calmodulin	n/a	4.71489297	SubthalamicNucleus	3056.65	1.83	n/a	8.64
CALM1	Calmodulin	n/a	4.71489297	SuperiorCervicalGanglion	88.30	-0.71	n/a	-3.36
CALM1	Calmodulin	n/a	4.71489297	TemporalLobe	2391.80	1.26	n/a	5.95
CALM1	Calmodulin	n/a	4.71489297	Testis	422.05	-0.43	n/a	-2.01
CALM1	Calmodulin	n/a	4.71489297	TestisGermCell	595.85	-0.28	n/a	-1.31
CALM1	Calmodulin	n/a	4.71489297	TestisIntersitial	234.15	-0.59	n/a	-2.77
CALM1	Calmodulin	n/a	4.71489297	TestisLeydigCell	221.45	-0.60	n/a	-2.82
CALM1	Calmodulin	n/a	4.71489297	TestisSeminiferousTubule	267.05	-0.56	n/a	-2.64
CALM1	Calmodulin	n/a	4.71489297	Thalamus	1659.00	0.63	n/a	2.99
CALM1	Calmodulin	n/a	4.71489297	Thymus	216.25	-0.60	n/a	-2.85
CALM1	Calmodulin	n/a	4.71489297	Thyroid	341.35	-0.50	n/a	-2.34
CALM1	Calmodulin	n/a	4.71489297	Tongue	153.90	-0.66	n/a	-3.10
CALM1	Calmodulin	n/a	4.71489297	Tonsil	116.85	-0.69	n/a	-3.25
CALM1	Calmodulin	n/a	4.71489297	Trachea	350.30	-0.49	n/a	-2.30
CALM1	Calmodulin	n/a	4.71489297	TrigeminalGanglion	61.40	-0.74	n/a	-3.47
CALM1	Calmodulin	n/a	4.71489297	Uterus	188.45	-0.63	n/a	-2.96
CALM1	Calmodulin	n/a	4.71489297	UterusCorpus	62.50	-0.74	n/a	-3.47
CALM1	Calmodulin	n/a	4.71489297	WholeBlood	519.10	-0.34	n/a	-1.62
CALM1	Calmodulin	n/a	4.71489297	Wholebrain	3275.40	2.02	n/a	9.52
CHRM1	Muscarinic acetylcholine receptor M1	9.008773924	7.698970004	Amygdala	11.92	0.31	2.81	2.40
CHRM1	Muscarinic acetylcholine receptor M1	9.008773924	7.698970004	Amygdala central nucleus	9.87	-0.96	-8.62	-7.36
CHRM1	Muscarinic acetylcholine receptor M1	9.008773924	7.698970004	Bone marrow	11.98	0.35	3.12	2.67
CHRM1	Muscarinic acetylcholine receptor M1	9.008773924	7.698970004	Cerebellum	10.66	-0.47	-4.21	-3.60
CHRM1	Muscarinic acetylcholine receptor M1	9.008773924	7.698970004	Cerebral cortex	11.71	0.18	1.63	1.39
CHRM1	Muscarinic acetylcholine receptor M1	9.008773924	7.698970004	Cornea	11.54	0.07	0.66	0.57
CHRM1	Muscarinic acetylcholine receptor M1	9.008773924	7.698970004	Dorsal raphe	10.74	-0.42	-3.77	-3.22
CHRM1	Muscarinic acetylcholine receptor M1	9.008773924	7.698970004	Dorsal root ganglion fisher	11.38	-0.02	-0.18	-0.15
CHRM1	Muscarinic acetylcholine receptor M1	9.008773924	7.698970004	Dorsal striatum	13.10	1.04	9.38	8.01
CHRM1	Muscarinic acetylcholine receptor M1	9.008773924	7.698970004	Endothelial cells	10.62	-0.50	-4.46	-3.81
CHRM1	Muscarinic acetylcholine receptor M1	9.008773924	7.698970004	Frontal cortex	11.35	-0.04	-0.40	-0.34
CHRM1	Muscarinic acetylcholine receptor M1	9.008773924	7.698970004	Heart	11.00	-0.26	-2.34	-2.00
CHRM1	Muscarinic acetylcholine receptor M1	9.008773924	7.698970004	Hippocampus	12.93	0.94	8.46	7.23
CHRM1	Muscarinic acetylcholine receptor M1	9.008773924	7.698970004	Hypothalamus	10.45	-0.60	-5.41	-4.63
CHRM1	Muscarinic acetylcholine receptor M1	9.008773924	7.698970004	Kidney	10.17	-0.77	-6.95	-5.94
CHRM1	Muscarinic acetylcholine receptor M1	9.008773924	7.698970004	Large intestine	9.24	-1.35	-12.16	-10.39
CHRM1	Muscarinic acetylcholine receptor M1	9.008773924	7.698970004	Locus coeruleus	11.62	0.12	1.12	0.96
CHRM1	Muscarinic acetylcholine receptor M1	9.008773924	7.698970004	Nucleus accumbens whole	11.50	0.05	0.49	0.42
CHRM1	Muscarinic acetylcholine receptor M1	9.008773924	7.698970004	Nucleus accumbens_core	11.76	0.21	1.90	1.62
CHRM1	Muscarinic acetylcholine receptor M1	9.008773924	7.698970004	Nucleus accumbens_shell	12.09	0.42	3.76	3.21
CHRM1	Muscarinic acetylcholine receptor M1	9.008773924	7.698970004	Pineal	10.28	-0.70	-6.31	-5.39
CHRM1	Muscarinic acetylcholine receptor M1	9.008773924	7.698970004	Pituitary	9.82	-0.99	-8.90	-7.60
CHRM1	Muscarinic acetylcholine receptor M1	9.008773924	7.698970004	Prefrontal cortex	18.05	4.11	37.00	31.62
CHRM1	Muscarinic acetylcholine receptor M1	9.008773924	7.698970004	Primary cortical neurons	13.37	1.21	10.92	9.33
CHRM1	Muscarinic acetylcholine receptor M1	9.008773924	7.698970004	Skeletal muscle	11.43	0.01	0.05	0.05

S2 Table. Target-tissue analysis for clozapine and chlorpromazine

Gene Symbol	Gene Name	Affinity (-log10(Ki))		Tissue	Expression		Combined Score (-log10(Ki)*Z)	
		clozapine	chlorpromazine		Raw-numbers	Z-score	clozapine	chlorpromazine
CHRM1	Muscarinic acetylcholine receptor M1	9.008773924	7.698970004	Small intestine	9.63	-1.10	-9.95	-8.51
CHRM1	Muscarinic acetylcholine receptor M1	9.008773924	7.698970004	Spleen	10.47	-0.59	-5.30	-4.53
CHRM1	Muscarinic acetylcholine receptor M1	9.008773924	7.698970004	Thymus	10.17	-0.77	-6.94	-5.93
CHRM1	Muscarinic acetylcholine receptor M1	9.008773924	7.698970004	Ventral striatum	12.08	0.41	3.69	3.15
CHRM1	Muscarinic acetylcholine receptor M1	9.008773924	7.698970004	Ventral tegmental area	11.58	0.10	0.92	0.79
CHRM2	Muscarinic acetylcholine receptor M2	6.772113295	6.634512015	Adipocyte	2.90	-0.21	-1.45	-1.42
CHRM2	Muscarinic acetylcholine receptor M2	6.772113295	6.634512015	AdrenalCortex	3.00	-0.09	-0.61	-0.60
CHRM2	Muscarinic acetylcholine receptor M2	6.772113295	6.634512015	Adrenalgland	2.45	-0.77	-5.24	-5.13
CHRM2	Muscarinic acetylcholine receptor M2	6.772113295	6.634512015	Amygdala	3.10	0.03	0.23	0.23
CHRM2	Muscarinic acetylcholine receptor M2	6.772113295	6.634512015	Appendix	3.05	-0.03	-0.19	-0.18
CHRM2	Muscarinic acetylcholine receptor M2	6.772113295	6.634512015	AtrioventricularNode	2.25	-1.02	-6.93	-6.78
CHRM2	Muscarinic acetylcholine receptor M2	6.772113295	6.634512015	BDCA4+_DentriticCells	3.30	0.28	1.92	1.88
CHRM2	Muscarinic acetylcholine receptor M2	6.772113295	6.634512015	Bonemarrow	2.90	-0.21	-1.45	-1.42
CHRM2	Muscarinic acetylcholine receptor M2	6.772113295	6.634512015	BronchialEpithelialCells	2.90	-0.21	-1.45	-1.42
CHRM2	Muscarinic acetylcholine receptor M2	6.772113295	6.634512015	CardiacMyocytes	3.65	0.72	4.87	4.77
CHRM2	Muscarinic acetylcholine receptor M2	6.772113295	6.634512015	Caudatenucleus	2.60	-0.59	-3.98	-3.90
CHRM2	Muscarinic acetylcholine receptor M2	6.772113295	6.634512015	CD105+_Endothelial	3.00	-0.09	-0.61	-0.60
CHRM2	Muscarinic acetylcholine receptor M2	6.772113295	6.634512015	CD14+_Monocytes	3.10	0.03	0.23	0.23
CHRM2	Muscarinic acetylcholine receptor M2	6.772113295	6.634512015	CD19+_BCells(neg._sel.)	3.15	0.10	0.66	0.64
CHRM2	Muscarinic acetylcholine receptor M2	6.772113295	6.634512015	CD33+_Myeloid	3.80	0.91	6.13	6.01
CHRM2	Muscarinic acetylcholine receptor M2	6.772113295	6.634512015	CD34+	3.75	0.84	5.71	5.59
CHRM2	Muscarinic acetylcholine receptor M2	6.772113295	6.634512015	CD4+_Tcells	3.25	0.22	1.50	1.47
CHRM2	Muscarinic acetylcholine receptor M2	6.772113295	6.634512015	CD56+_NKCells	3.55	0.59	4.03	3.94
CHRM2	Muscarinic acetylcholine receptor M2	6.772113295	6.634512015	CD71+_EarlyErythroid	2.70	-0.46	-3.13	-3.07
CHRM2	Muscarinic acetylcholine receptor M2	6.772113295	6.634512015	CD8+_Tcells	2.90	-0.21	-1.45	-1.42
CHRM2	Muscarinic acetylcholine receptor M2	6.772113295	6.634512015	Cerebellum	2.60	-0.59	-3.98	-3.90
CHRM2	Muscarinic acetylcholine receptor M2	6.772113295	6.634512015	CerebellumPeduncles	3.30	0.28	1.92	1.88
CHRM2	Muscarinic acetylcholine receptor M2	6.772113295	6.634512015	CiliaryGanglion	2.90	-0.21	-1.45	-1.42
CHRM2	Muscarinic acetylcholine receptor M2	6.772113295	6.634512015	CingulateCortex	3.00	-0.09	-0.61	-0.60
CHRM2	Muscarinic acetylcholine receptor M2	6.772113295	6.634512015	Colon	2.95	-0.15	-1.03	-1.01
CHRM2	Muscarinic acetylcholine receptor M2	6.772113295	6.634512015	DorsalRootGanglion	3.65	0.72	4.87	4.77
CHRM2	Muscarinic acetylcholine receptor M2	6.772113295	6.634512015	Fetalbrain	2.95	-0.15	-1.03	-1.01
CHRM2	Muscarinic acetylcholine receptor M2	6.772113295	6.634512015	Fetalliver	2.55	-0.65	-4.40	-4.31
CHRM2	Muscarinic acetylcholine receptor M2	6.772113295	6.634512015	Fetallung	2.45	-0.77	-5.24	-5.13
CHRM2	Muscarinic acetylcholine receptor M2	6.772113295	6.634512015	FetalThyroid	2.85	-0.28	-1.87	-1.83
CHRM2	Muscarinic acetylcholine receptor M2	6.772113295	6.634512015	GlobusPallidus	2.50	-0.71	-4.82	-4.72
CHRM2	Muscarinic acetylcholine receptor M2	6.772113295	6.634512015	Heart	3.75	0.84	5.71	5.59
CHRM2	Muscarinic acetylcholine receptor M2	6.772113295	6.634512015	Hypothalamus	3.20	0.16	1.08	1.06
CHRM2	Muscarinic acetylcholine receptor M2	6.772113295	6.634512015	Kidney	2.35	-0.90	-6.08	-5.96
CHRM2	Muscarinic acetylcholine receptor M2	6.772113295	6.634512015	Liver	3.80	0.91	6.13	6.01
CHRM2	Muscarinic acetylcholine receptor M2	6.772113295	6.634512015	Lung	3.25	0.22	1.50	1.47
CHRM2	Muscarinic acetylcholine receptor M2	6.772113295	6.634512015	Lymphnode	2.55	-0.65	-4.40	-4.31
CHRM2	Muscarinic acetylcholine receptor M2	6.772113295	6.634512015	MedullaOblongata	2.70	-0.46	-3.13	-3.07
CHRM2	Muscarinic acetylcholine receptor M2	6.772113295	6.634512015	OccipitalLobe	2.65	-0.53	-3.56	-3.48
CHRM2	Muscarinic acetylcholine receptor M2	6.772113295	6.634512015	OlfactoryBulb	2.35	-0.90	-6.08	-5.96
CHRM2	Muscarinic acetylcholine receptor M2	6.772113295	6.634512015	Ovary	2.10	-1.21	-8.19	-8.02
CHRM2	Muscarinic acetylcholine receptor M2	6.772113295	6.634512015	Pancreas	2.35	-0.90	-6.08	-5.96
CHRM2	Muscarinic acetylcholine receptor M2	6.772113295	6.634512015	PancreaticIslet	3.15	0.10	0.66	0.64
CHRM2	Muscarinic acetylcholine receptor M2	6.772113295	6.634512015	ParietalLobe	3.40	0.41	2.76	2.71
CHRM2	Muscarinic acetylcholine receptor M2	6.772113295	6.634512015	Pineal_day	4.84	2.20	14.89	14.59
CHRM2	Muscarinic acetylcholine receptor M2	6.772113295	6.634512015	Pineal_night	3.64	0.71	4.78	4.69
CHRM2	Muscarinic acetylcholine receptor M2	6.772113295	6.634512015	Pituitary	3.45	0.47	3.18	3.12
CHRM2	Muscarinic acetylcholine receptor M2	6.772113295	6.634512015	Placenta	3.05	-0.03	-0.19	-0.18

S2 Table. Target-tissue analysis for clozapine and chlorpromazine

Gene Symbol	Gene Name	Affinity (-log10(Ki))		Tissue	Expression		Combined Score (-log10(Ki)*Z)	
		clozapine	chlorpromazine		Raw-numbers	Z-score	clozapine	chlorpromazine
CHRM2	Muscarinic acetylcholine receptor M2	6.772113295	6.634512015	Pons	2.75	-0.40	-2.71	-2.66
CHRM2	Muscarinic acetylcholine receptor M2	6.772113295	6.634512015	PrefrontalCortex	3.70	0.78	5.29	5.18
CHRM2	Muscarinic acetylcholine receptor M2	6.772113295	6.634512015	Prostate	3.50	0.53	3.60	3.53
CHRM2	Muscarinic acetylcholine receptor M2	6.772113295	6.634512015	Retina	3.58	0.63	4.24	4.15
CHRM2	Muscarinic acetylcholine receptor M2	6.772113295	6.634512015	Salivarygland	2.30	-0.96	-6.50	-6.37
CHRM2	Muscarinic acetylcholine receptor M2	6.772113295	6.634512015	SkeletalMuscle	7.90	6.00	40.67	39.84
CHRM2	Muscarinic acetylcholine receptor M2	6.772113295	6.634512015	Skin	2.25	-1.02	-6.93	-6.78
CHRM2	Muscarinic acetylcholine receptor M2	6.772113295	6.634512015	Small_intestine	2.85	-0.28	-1.87	-1.83
CHRM2	Muscarinic acetylcholine receptor M2	6.772113295	6.634512015	SmoothMuscle	3.30	0.28	1.92	1.88
CHRM2	Muscarinic acetylcholine receptor M2	6.772113295	6.634512015	Spinalcord	3.15	0.10	0.66	0.64
CHRM2	Muscarinic acetylcholine receptor M2	6.772113295	6.634512015	SubthalamicNucleus	2.65	-0.53	-3.56	-3.48
CHRM2	Muscarinic acetylcholine receptor M2	6.772113295	6.634512015	SuperiorCervicalGanglion	5.70	3.27	22.14	21.69
CHRM2	Muscarinic acetylcholine receptor M2	6.772113295	6.634512015	TemporalLobe	2.90	-0.21	-1.45	-1.42
CHRM2	Muscarinic acetylcholine receptor M2	6.772113295	6.634512015	Testis	2.50	-0.71	-4.82	-4.72
CHRM2	Muscarinic acetylcholine receptor M2	6.772113295	6.634512015	TestisGermCell	2.50	-0.71	-4.82	-4.72
CHRM2	Muscarinic acetylcholine receptor M2	6.772113295	6.634512015	TestisIntersitial	2.45	-0.77	-5.24	-5.13
CHRM2	Muscarinic acetylcholine receptor M2	6.772113295	6.634512015	TestisLeydigCell	2.90	-0.21	-1.45	-1.42
CHRM2	Muscarinic acetylcholine receptor M2	6.772113295	6.634512015	TestisSeminiferousTubule	2.50	-0.71	-4.82	-4.72
CHRM2	Muscarinic acetylcholine receptor M2	6.772113295	6.634512015	Thalamus	3.00	-0.09	-0.61	-0.60
CHRM2	Muscarinic acetylcholine receptor M2	6.772113295	6.634512015	Thymus	2.35	-0.90	-6.08	-5.96
CHRM2	Muscarinic acetylcholine receptor M2	6.772113295	6.634512015	Thyroid	3.70	0.78	5.29	5.18
CHRM2	Muscarinic acetylcholine receptor M2	6.772113295	6.634512015	Tongue	2.90	-0.21	-1.45	-1.42
CHRM2	Muscarinic acetylcholine receptor M2	6.772113295	6.634512015	Tonsil	2.85	-0.28	-1.87	-1.83
CHRM2	Muscarinic acetylcholine receptor M2	6.772113295	6.634512015	Trachea	2.45	-0.77	-5.24	-5.13
CHRM2	Muscarinic acetylcholine receptor M2	6.772113295	6.634512015	TrigeminalGanglion	2.95	-0.15	-1.03	-1.01
CHRM2	Muscarinic acetylcholine receptor M2	6.772113295	6.634512015	Uterus	2.40	-0.84	-5.66	-5.55
CHRM2	Muscarinic acetylcholine receptor M2	6.772113295	6.634512015	UterusCorpus	3.25	0.22	1.50	1.47
CHRM2	Muscarinic acetylcholine receptor M2	6.772113295	6.634512015	WholeBlood	3.25	0.22	1.50	1.47
CHRM2	Muscarinic acetylcholine receptor M2	6.772113295	6.634512015	Wholebrain	2.50	-0.71	-4.82	-4.72
CHRM3	Muscarinic acetylcholine receptor M3	7.769551079	7.356547324	Adipocyte	3.65	-0.17	-1.32	-1.25
CHRM3	Muscarinic acetylcholine receptor M3	7.769551079	7.356547324	AdrenalCortex	3.85	-0.15	-1.20	-1.13
CHRM3	Muscarinic acetylcholine receptor M3	7.769551079	7.356547324	Adrenalgland	3.10	-0.21	-1.67	-1.58
CHRM3	Muscarinic acetylcholine receptor M3	7.769551079	7.356547324	Amygdala	19.10	1.08	8.36	7.92
CHRM3	Muscarinic acetylcholine receptor M3	7.769551079	7.356547324	Appendix	4.25	-0.12	-0.95	-0.90
CHRM3	Muscarinic acetylcholine receptor M3	7.769551079	7.356547324	AtrioventricularNode	2.85	-0.23	-1.83	-1.73
CHRM3	Muscarinic acetylcholine receptor M3	7.769551079	7.356547324	BDCA4+_DentriticCells	4.05	-0.14	-1.07	-1.02
CHRM3	Muscarinic acetylcholine receptor M3	7.769551079	7.356547324	Bonemarrow	3.65	-0.17	-1.32	-1.25
CHRM3	Muscarinic acetylcholine receptor M3	7.769551079	7.356547324	BronchialEpithelialCells	3.60	-0.17	-1.36	-1.28
CHRM3	Muscarinic acetylcholine receptor M3	7.769551079	7.356547324	CardiacMyocytes	5.00	-0.06	-0.48	-0.45
CHRM3	Muscarinic acetylcholine receptor M3	7.769551079	7.356547324	Caudatenucleus	3.30	-0.20	-1.54	-1.46
CHRM3	Muscarinic acetylcholine receptor M3	7.769551079	7.356547324	CD105+_Endothelial	3.80	-0.16	-1.23	-1.16
CHRM3	Muscarinic acetylcholine receptor M3	7.769551079	7.356547324	CD14+_Monocytes	3.90	-0.15	-1.17	-1.11
CHRM3	Muscarinic acetylcholine receptor M3	7.769551079	7.356547324	CD19+_BCells(neg._sel.)	4.00	-0.14	-1.10	-1.05
CHRM3	Muscarinic acetylcholine receptor M3	7.769551079	7.356547324	CD33+_Myeloid	4.75	-0.08	-0.63	-0.60
CHRM3	Muscarinic acetylcholine receptor M3	7.769551079	7.356547324	CD34+	4.75	-0.08	-0.63	-0.60
CHRM3	Muscarinic acetylcholine receptor M3	7.769551079	7.356547324	CD4+_Tcells	4.00	-0.14	-1.10	-1.05
CHRM3	Muscarinic acetylcholine receptor M3	7.769551079	7.356547324	CD56+_NKCells	4.45	-0.11	-0.82	-0.78
CHRM3	Muscarinic acetylcholine receptor M3	7.769551079	7.356547324	CD71+_EarlyErythroid	3.45	-0.19	-1.45	-1.37
CHRM3	Muscarinic acetylcholine receptor M3	7.769551079	7.356547324	CD8+_Tcells	3.60	-0.17	-1.36	-1.28
CHRM3	Muscarinic acetylcholine receptor M3	7.769551079	7.356547324	Cerebellum	2.95	-0.23	-1.76	-1.67
CHRM3	Muscarinic acetylcholine receptor M3	7.769551079	7.356547324	CerebellumPeduncles	4.20	-0.13	-0.98	-0.93
CHRM3	Muscarinic acetylcholine receptor M3	7.769551079	7.356547324	CiliaryGanglion	2.65	-0.25	-1.95	-1.85
CHRM3	Muscarinic acetylcholine receptor M3	7.769551079	7.356547324	CingulateCortex	4.00	-0.14	-1.10	-1.05

Gene Symbol	Gene Name	Affinity (-log10(Ki))		Tissue	Expression		Combined Score (-log10(Ki)*Z)	
		clozapine	chlorpromazine		Raw-numbers	Z-score	clozapine	chlorpromazine
CHRM3	Muscarinic acetylcholine receptor M3	7.769551079	7.356547324	Colon	4.40	-0.11	-0.85	-0.81
CHRM3	Muscarinic acetylcholine receptor M3	7.769551079	7.356547324	DorsalRootGanglion	2.85	-0.23	-1.83	-1.73
CHRM3	Muscarinic acetylcholine receptor M3	7.769551079	7.356547324	Fetalbrain	11.70	0.48	3.72	3.52
CHRM3	Muscarinic acetylcholine receptor M3	7.769551079	7.356547324	Fetalliver	3.20	-0.21	-1.61	-1.52
CHRM3	Muscarinic acetylcholine receptor M3	7.769551079	7.356547324	Fetallung	3.10	-0.21	-1.67	-1.58
CHRM3	Muscarinic acetylcholine receptor M3	7.769551079	7.356547324	FetalThyroid	3.60	-0.17	-1.36	-1.28
CHRM3	Muscarinic acetylcholine receptor M3	7.769551079	7.356547324	GlobusPallidus	2.80	-0.24	-1.86	-1.76
CHRM3	Muscarinic acetylcholine receptor M3	7.769551079	7.356547324	Heart	4.90	-0.07	-0.54	-0.51
CHRM3	Muscarinic acetylcholine receptor M3	7.769551079	7.356547324	Hypothalamus	4.15	-0.13	-1.01	-0.96
CHRM3	Muscarinic acetylcholine receptor M3	7.769551079	7.356547324	Kidney	3.00	-0.22	-1.73	-1.64
CHRM3	Muscarinic acetylcholine receptor M3	7.769551079	7.356547324	Liver	4.90	-0.07	-0.54	-0.51
CHRM3	Muscarinic acetylcholine receptor M3	7.769551079	7.356547324	Lung	4.00	-0.14	-1.10	-1.05
CHRM3	Muscarinic acetylcholine receptor M3	7.769551079	7.356547324	Lymphnode	3.15	-0.21	-1.64	-1.55
CHRM3	Muscarinic acetylcholine receptor M3	7.769551079	7.356547324	MedullaOblongata	5.55	-0.02	-0.13	-0.13
CHRM3	Muscarinic acetylcholine receptor M3	7.769551079	7.356547324	OccipitalLobe	8.70	0.24	1.84	1.74
CHRM3	Muscarinic acetylcholine receptor M3	7.769551079	7.356547324	OlfactoryBulb	2.90	-0.23	-1.79	-1.70
CHRM3	Muscarinic acetylcholine receptor M3	7.769551079	7.356547324	Ovary	2.50	-0.26	-2.04	-1.94
CHRM3	Muscarinic acetylcholine receptor M3	7.769551079	7.356547324	Pancreas	4.00	-0.14	-1.10	-1.05
CHRM3	Muscarinic acetylcholine receptor M3	7.769551079	7.356547324	PancreaticIslet	3.95	-0.15	-1.14	-1.08
CHRM3	Muscarinic acetylcholine receptor M3	7.769551079	7.356547324	ParietalLobe	4.20	-0.13	-0.98	-0.93
CHRM3	Muscarinic acetylcholine receptor M3	7.769551079	7.356547324	Pineal_day	4.62	-0.09	-0.72	-0.68
CHRM3	Muscarinic acetylcholine receptor M3	7.769551079	7.356547324	Pineal_night	4.50	-0.10	-0.79	-0.75
CHRM3	Muscarinic acetylcholine receptor M3	7.769551079	7.356547324	Pituitary	4.25	-0.12	-0.95	-0.90
CHRM3	Muscarinic acetylcholine receptor M3	7.769551079	7.356547324	Placenta	3.85	-0.15	-1.20	-1.13
CHRM3	Muscarinic acetylcholine receptor M3	7.769551079	7.356547324	Pons	3.55	-0.18	-1.39	-1.31
CHRM3	Muscarinic acetylcholine receptor M3	7.769551079	7.356547324	PrefrontalCortex	110.70	8.47	65.78	62.28
CHRM3	Muscarinic acetylcholine receptor M3	7.769551079	7.356547324	Prostate	4.40	-0.11	-0.85	-0.81
CHRM3	Muscarinic acetylcholine receptor M3	7.769551079	7.356547324	Retina	8.53	0.22	1.73	1.64
CHRM3	Muscarinic acetylcholine receptor M3	7.769551079	7.356547324	Salivarygland	2.90	-0.23	-1.79	-1.70
CHRM3	Muscarinic acetylcholine receptor M3	7.769551079	7.356547324	SkeletalMuscle	4.10	-0.13	-1.04	-0.99
CHRM3	Muscarinic acetylcholine receptor M3	7.769551079	7.356547324	Skin	3.50	-0.18	-1.42	-1.34
CHRM3	Muscarinic acetylcholine receptor M3	7.769551079	7.356547324	Small_intestine	3.85	-0.15	-1.20	-1.13
CHRM3	Muscarinic acetylcholine receptor M3	7.769551079	7.356547324	SmoothMuscle	4.20	-0.13	-0.98	-0.93
CHRM3	Muscarinic acetylcholine receptor M3	7.769551079	7.356547324	Spinalcord	3.90	-0.15	-1.17	-1.11
CHRM3	Muscarinic acetylcholine receptor M3	7.769551079	7.356547324	SubthalamicNucleus	5.65	-0.01	-0.07	-0.07
CHRM3	Muscarinic acetylcholine receptor M3	7.769551079	7.356547324	SuperiorCervicalGanglion	4.20	-0.13	-0.98	-0.93
CHRM3	Muscarinic acetylcholine receptor M3	7.769551079	7.356547324	TemporalLobe	3.75	-0.16	-1.26	-1.19
CHRM3	Muscarinic acetylcholine receptor M3	7.769551079	7.356547324	Testis	3.15	-0.21	-1.64	-1.55
CHRM3	Muscarinic acetylcholine receptor M3	7.769551079	7.356547324	TestisGermCell	3.05	-0.22	-1.70	-1.61
CHRM3	Muscarinic acetylcholine receptor M3	7.769551079	7.356547324	TestisIntersitial	3.10	-0.21	-1.67	-1.58
CHRM3	Muscarinic acetylcholine receptor M3	7.769551079	7.356547324	TestisLeydigCell	3.65	-0.17	-1.32	-1.25
CHRM3	Muscarinic acetylcholine receptor M3	7.769551079	7.356547324	TestisSeminiferousTubule	3.15	-0.21	-1.64	-1.55
CHRM3	Muscarinic acetylcholine receptor M3	7.769551079	7.356547324	Thalamus	3.75	-0.16	-1.26	-1.19
CHRM3	Muscarinic acetylcholine receptor M3	7.769551079	7.356547324	Thymus	2.90	-0.23	-1.79	-1.70
CHRM3	Muscarinic acetylcholine receptor M3	7.769551079	7.356547324	Thyroid	4.65	-0.09	-0.70	-0.66
CHRM3	Muscarinic acetylcholine receptor M3	7.769551079	7.356547324	Tongue	3.60	-0.17	-1.36	-1.28
CHRM3	Muscarinic acetylcholine receptor M3	7.769551079	7.356547324	Tonsil	3.60	-0.17	-1.36	-1.28
CHRM3	Muscarinic acetylcholine receptor M3	7.769551079	7.356547324	Trachea	3.20	-0.21	-1.61	-1.52
CHRM3	Muscarinic acetylcholine receptor M3	7.769551079	7.356547324	TrigeminalGanglion	3.70	-0.17	-1.29	-1.22
CHRM3	Muscarinic acetylcholine receptor M3	7.769551079	7.356547324	Uterus	3.05	-0.22	-1.70	-1.61
CHRM3	Muscarinic acetylcholine receptor M3	7.769551079	7.356547324	UterusCorpus	3.30	-0.20	-1.54	-1.46
CHRM3	Muscarinic acetylcholine receptor M3	7.769551079	7.356547324	WholeBlood	4.10	-0.13	-1.04	-0.99
CHRM3	Muscarinic acetylcholine receptor M3	7.769551079	7.356547324	Wholebrain	16.85	0.89	6.95	6.58

S2 Table. Target-tissue analysis for clozapine and chlorpromazine

Gene Symbol	Gene Name	Affinity (-log10(Ki))		Tissue	Expression		Combined Score (-log10(Ki)*Z)	
		clozapine	chlorpromazine		Raw-numbers	Z-score	clozapine	chlorpromazine
CHRM4	Muscarinic acetylcholine receptor M4	8.200659451	7.677780705	Adipocyte	4.25	-0.03	-0.22	-0.21
CHRM4	Muscarinic acetylcholine receptor M4	8.200659451	7.677780705	AdrenalCortex	4.45	0.26	2.15	2.01
CHRM4	Muscarinic acetylcholine receptor M4	8.200659451	7.677780705	Adrenalgland	3.60	-0.97	-7.93	-7.42
CHRM4	Muscarinic acetylcholine receptor M4	8.200659451	7.677780705	Amygdala	4.50	0.33	2.74	2.57
CHRM4	Muscarinic acetylcholine receptor M4	8.200659451	7.677780705	Appendix	4.30	0.05	0.37	0.35
CHRM4	Muscarinic acetylcholine receptor M4	8.200659451	7.677780705	AtrioventricularNode	3.25	-1.47	-12.08	-11.31
CHRM4	Muscarinic acetylcholine receptor M4	8.200659451	7.677780705	BDCA4+_DentriticCells	4.65	0.55	4.52	4.23
CHRM4	Muscarinic acetylcholine receptor M4	8.200659451	7.677780705	Bonemarrow	4.25	-0.03	-0.22	-0.21
CHRM4	Muscarinic acetylcholine receptor M4	8.200659451	7.677780705	BronchialEpithelialCells	4.25	-0.03	-0.22	-0.21
CHRM4	Muscarinic acetylcholine receptor M4	8.200659451	7.677780705	CardiacMyocytes	5.45	1.71	14.01	13.12
CHRM4	Muscarinic acetylcholine receptor M4	8.200659451	7.677780705	Caudatenucleus	3.85	-0.61	-4.96	-4.65
CHRM4	Muscarinic acetylcholine receptor M4	8.200659451	7.677780705	CD105+_Endothelial	4.35	0.12	0.96	0.90
CHRM4	Muscarinic acetylcholine receptor M4	8.200659451	7.677780705	CD14+_Monocytes	4.55	0.41	3.34	3.12
CHRM4	Muscarinic acetylcholine receptor M4	8.200659451	7.677780705	CD19+_BCells(neg._sel.)	4.65	0.55	4.52	4.23
CHRM4	Muscarinic acetylcholine receptor M4	8.200659451	7.677780705	CD33+_Myeloid	5.50	1.78	14.60	13.67
CHRM4	Muscarinic acetylcholine receptor M4	8.200659451	7.677780705	CD34+	5.50	1.78	14.60	13.67
CHRM4	Muscarinic acetylcholine receptor M4	8.200659451	7.677780705	CD4+_Tcells	4.70	0.62	5.12	4.79
CHRM4	Muscarinic acetylcholine receptor M4	8.200659451	7.677780705	CD56+_NKCells	5.20	1.35	11.04	10.34
CHRM4	Muscarinic acetylcholine receptor M4	8.200659451	7.677780705	CD71+_EarlyErythroid	4.00	-0.39	-3.19	-2.98
CHRM4	Muscarinic acetylcholine receptor M4	8.200659451	7.677780705	CD8+_Tcells	4.15	-0.17	-1.41	-1.32
CHRM4	Muscarinic acetylcholine receptor M4	8.200659451	7.677780705	Cerebellum	3.45	-1.18	-9.71	-9.09
CHRM4	Muscarinic acetylcholine receptor M4	8.200659451	7.677780705	CerebellumPeduncles	4.90	0.91	7.49	7.01
CHRM4	Muscarinic acetylcholine receptor M4	8.200659451	7.677780705	CiliaryGanglion	3.00	-1.83	-15.04	-14.08
CHRM4	Muscarinic acetylcholine receptor M4	8.200659451	7.677780705	CingulateCortex	4.40	0.19	1.56	1.46
CHRM4	Muscarinic acetylcholine receptor M4	8.200659451	7.677780705	Colon	4.30	0.05	0.37	0.35
CHRM4	Muscarinic acetylcholine receptor M4	8.200659451	7.677780705	DorsalRootGanglion	3.25	-1.47	-12.08	-11.31
CHRM4	Muscarinic acetylcholine receptor M4	8.200659451	7.677780705	Fetalbrain	4.30	0.05	0.37	0.35
CHRM4	Muscarinic acetylcholine receptor M4	8.200659451	7.677780705	Fetalliver	3.75	-0.75	-6.15	-5.76
CHRM4	Muscarinic acetylcholine receptor M4	8.200659451	7.677780705	Fetallung	3.65	-0.89	-7.34	-6.87
CHRM4	Muscarinic acetylcholine receptor M4	8.200659451	7.677780705	FetalThyroid	4.20	-0.10	-0.81	-0.76
CHRM4	Muscarinic acetylcholine receptor M4	8.200659451	7.677780705	GlobusPallidus	3.15	-1.62	-13.27	-12.42
CHRM4	Muscarinic acetylcholine receptor M4	8.200659451	7.677780705	Heart	5.50	1.78	14.60	13.67
CHRM4	Muscarinic acetylcholine receptor M4	8.200659451	7.677780705	Hypothalamus	4.65	0.55	4.52	4.23
CHRM4	Muscarinic acetylcholine receptor M4	8.200659451	7.677780705	Kidney	3.45	-1.18	-9.71	-9.09
CHRM4	Muscarinic acetylcholine receptor M4	8.200659451	7.677780705	Liver	5.95	2.43	19.94	18.67
CHRM4	Muscarinic acetylcholine receptor M4	8.200659451	7.677780705	Lung	4.75	0.70	5.71	5.34
CHRM4	Muscarinic acetylcholine receptor M4	8.200659451	7.677780705	Lymphnode	3.70	-0.82	-6.74	-6.31
CHRM4	Muscarinic acetylcholine receptor M4	8.200659451	7.677780705	MedullaOblongata	3.95	-0.46	-3.78	-3.54
CHRM4	Muscarinic acetylcholine receptor M4	8.200659451	7.677780705	OccipitalLobe	3.85	-0.61	-4.96	-4.65
CHRM4	Muscarinic acetylcholine receptor M4	8.200659451	7.677780705	OlfactoryBulb	3.40	-1.26	-10.30	-9.64
CHRM4	Muscarinic acetylcholine receptor M4	8.200659451	7.677780705	Ovary	2.85	-2.05	-16.82	-15.75
CHRM4	Muscarinic acetylcholine receptor M4	8.200659451	7.677780705	Pancreas	3.50	-1.11	-9.11	-8.53
CHRM4	Muscarinic acetylcholine receptor M4	8.200659451	7.677780705	PancreaticIslet	4.60	0.48	3.93	3.68
CHRM4	Muscarinic acetylcholine receptor M4	8.200659451	7.677780705	ParietalLobe	4.50	0.33	2.74	2.57
CHRM4	Muscarinic acetylcholine receptor M4	8.200659451	7.677780705	Pineal_day	5.34	1.55	12.70	11.89
CHRM4	Muscarinic acetylcholine receptor M4	8.200659451	7.677780705	Pineal_night	5.22	1.38	11.28	10.56
CHRM4	Muscarinic acetylcholine receptor M4	8.200659451	7.677780705	Pituitary	5.00	1.06	8.67	8.12
CHRM4	Muscarinic acetylcholine receptor M4	8.200659451	7.677780705	Placenta	4.45	0.26	2.15	2.01
CHRM4	Muscarinic acetylcholine receptor M4	8.200659451	7.677780705	Pons	4.10	-0.24	-2.00	-1.87
CHRM4	Muscarinic acetylcholine receptor M4	8.200659451	7.677780705	PrefrontalCortex	5.35	1.56	12.82	12.01
CHRM4	Muscarinic acetylcholine receptor M4	8.200659451	7.677780705	Prostate	5.05	1.13	9.27	8.68
CHRM4	Muscarinic acetylcholine receptor M4	8.200659451	7.677780705	Retina	5.18	1.31	10.75	10.06
CHRM4	Muscarinic acetylcholine receptor M4	8.200659451	7.677780705	Salivarygland	3.40	-1.26	-10.30	-9.64

S2 Table. Target-tissue analysis for clozapine and chlorpromazine

Gene Symbol	Gene Name	Affinity (-log10(Ki))		Tissue	Expression		Combined Score (-log10(Ki)*Z)	
		clozapine	chlorpromazine		Raw-numbers	Z-score	clozapine	chlorpromazine
CHRM4	Muscarinic acetylcholine receptor M4	8.200659451	7.677780705	SkeletalMuscle	4.95	0.99	8.08	7.56
CHRM4	Muscarinic acetylcholine receptor M4	8.200659451	7.677780705	Skin	3.40	-1.26	-10.30	-9.64
CHRM4	Muscarinic acetylcholine receptor M4	8.200659451	7.677780705	Small_intestine	4.20	-0.10	-0.81	-0.76
CHRM4	Muscarinic acetylcholine receptor M4	8.200659451	7.677780705	SmoothMuscle	4.90	0.91	7.49	7.01
CHRM4	Muscarinic acetylcholine receptor M4	8.200659451	7.677780705	Spinalcord	4.60	0.48	3.93	3.68
CHRM4	Muscarinic acetylcholine receptor M4	8.200659451	7.677780705	SubthalamicNucleus	3.90	-0.53	-4.37	-4.09
CHRM4	Muscarinic acetylcholine receptor M4	8.200659451	7.677780705	SuperiorCervicalGanglion	4.85	0.84	6.89	6.45
CHRM4	Muscarinic acetylcholine receptor M4	8.200659451	7.677780705	TemporalLobe	3.95	-0.46	-3.78	-3.54
CHRM4	Muscarinic acetylcholine receptor M4	8.200659451	7.677780705	Testis	3.75	-0.75	-6.15	-5.76
CHRM4	Muscarinic acetylcholine receptor M4	8.200659451	7.677780705	TestisGermCell	3.65	-0.89	-7.34	-6.87
CHRM4	Muscarinic acetylcholine receptor M4	8.200659451	7.677780705	TestisIntersitial	3.65	-0.89	-7.34	-6.87
CHRM4	Muscarinic acetylcholine receptor M4	8.200659451	7.677780705	TestisLeydigCell	4.25	-0.03	-0.22	-0.21
CHRM4	Muscarinic acetylcholine receptor M4	8.200659451	7.677780705	TestisSeminiferousTubule	3.70	-0.82	-6.74	-6.31
CHRM4	Muscarinic acetylcholine receptor M4	8.200659451	7.677780705	Thalamus	4.35	0.12	0.96	0.90
CHRM4	Muscarinic acetylcholine receptor M4	8.200659451	7.677780705	Thymus	3.40	-1.26	-10.30	-9.64
CHRM4	Muscarinic acetylcholine receptor M4	8.200659451	7.677780705	Thyroid	5.45	1.71	14.01	13.12
CHRM4	Muscarinic acetylcholine receptor M4	8.200659451	7.677780705	Tongue	4.25	-0.03	-0.22	-0.21
CHRM4	Muscarinic acetylcholine receptor M4	8.200659451	7.677780705	Tonsil	4.20	-0.10	-0.81	-0.76
CHRM4	Muscarinic acetylcholine receptor M4	8.200659451	7.677780705	Trachea	3.60	-0.97	-7.93	-7.42
CHRM4	Muscarinic acetylcholine receptor M4	8.200659451	7.677780705	TrigeminalGanglion	4.25	-0.03	-0.22	-0.21
CHRM4	Muscarinic acetylcholine receptor M4	8.200659451	7.677780705	Uterus	3.50	-1.11	-9.11	-8.53
CHRM4	Muscarinic acetylcholine receptor M4	8.200659451	7.677780705	UterusCorpus	4.00	-0.39	-3.19	-2.98
CHRM4	Muscarinic acetylcholine receptor M4	8.200659451	7.677780705	WholeBlood	4.75	0.70	5.71	5.34
CHRM4	Muscarinic acetylcholine receptor M4	8.200659451	7.677780705	Wholebrain	3.70	-0.82	-6.74	-6.31
CHRM5	Muscarinic acetylcholine receptor M5	8.020907099	7.744727495	Adipocyte	15.00	-0.19	-1.49	-1.44
CHRM5	Muscarinic acetylcholine receptor M5	8.020907099	7.744727495	AdrenalCortex	16.45	-0.08	-0.61	-0.59
CHRM5	Muscarinic acetylcholine receptor M5	8.020907099	7.744727495	Adrenalgland	14.55	-0.22	-1.76	-1.70
CHRM5	Muscarinic acetylcholine receptor M5	8.020907099	7.744727495	Amygdala	14.95	-0.19	-1.52	-1.47
CHRM5	Muscarinic acetylcholine receptor M5	8.020907099	7.744727495	Appendix	20.25	0.21	1.71	1.65
CHRM5	Muscarinic acetylcholine receptor M5	8.020907099	7.744727495	AtrioventricularNode	24.25	0.52	4.15	4.00
CHRM5	Muscarinic acetylcholine receptor M5	8.020907099	7.744727495	BDCA4+_DentriticCells	9.80	-0.58	-4.66	-4.50
CHRM5	Muscarinic acetylcholine receptor M5	8.020907099	7.744727495	Bonemarrow	24.25	0.52	4.15	4.00
CHRM5	Muscarinic acetylcholine receptor M5	8.020907099	7.744727495	BronchialEpithelialCells	13.10	-0.33	-2.65	-2.56
CHRM5	Muscarinic acetylcholine receptor M5	8.020907099	7.744727495	CardiacMyocytes	18.60	0.09	0.70	0.68
CHRM5	Muscarinic acetylcholine receptor M5	8.020907099	7.744727495	Caudatenucleus	13.45	-0.30	-2.43	-2.35
CHRM5	Muscarinic acetylcholine receptor M5	8.020907099	7.744727495	CD105+_Endothelial	8.60	-0.67	-5.39	-5.20
CHRM5	Muscarinic acetylcholine receptor M5	8.020907099	7.744727495	CD14+_Monocytes	8.45	-0.68	-5.48	-5.29
CHRM5	Muscarinic acetylcholine receptor M5	8.020907099	7.744727495	CD19+_BCells(neg._sel.)	8.35	-0.69	-5.54	-5.35
CHRM5	Muscarinic acetylcholine receptor M5	8.020907099	7.744727495	CD33+_Myeloid	11.60	-0.44	-3.56	-3.44
CHRM5	Muscarinic acetylcholine receptor M5	8.020907099	7.744727495	CD34+	16.70	-0.06	-0.45	-0.44
CHRM5	Muscarinic acetylcholine receptor M5	8.020907099	7.744727495	CD4+_Tcells	8.05	-0.71	-5.72	-5.53
CHRM5	Muscarinic acetylcholine receptor M5	8.020907099	7.744727495	CD56+_NKCells	10.65	-0.52	-4.14	-4.00
CHRM5	Muscarinic acetylcholine receptor M5	8.020907099	7.744727495	CD71+_EarlyErythroid	13.75	-0.28	-2.25	-2.17
CHRM5	Muscarinic acetylcholine receptor M5	8.020907099	7.744727495	CD8+_Tcells	9.30	-0.62	-4.96	-4.79
CHRM5	Muscarinic acetylcholine receptor M5	8.020907099	7.744727495	Cerebellum	27.65	0.78	6.22	6.00
CHRM5	Muscarinic acetylcholine receptor M5	8.020907099	7.744727495	CerebellumPeduncles	19.25	0.14	1.10	1.06
CHRM5	Muscarinic acetylcholine receptor M5	8.020907099	7.744727495	CiliaryGanglion	21.90	0.34	2.71	2.62
CHRM5	Muscarinic acetylcholine receptor M5	8.020907099	7.744727495	CingulateCortex	20.10	0.20	1.62	1.56
CHRM5	Muscarinic acetylcholine receptor M5	8.020907099	7.744727495	Colon	12.10	-0.41	-3.26	-3.14
CHRM5	Muscarinic acetylcholine receptor M5	8.020907099	7.744727495	DorsalRootGanglion	12.50	-0.38	-3.01	-2.91
CHRM5	Muscarinic acetylcholine receptor M5	8.020907099	7.744727495	Fetalbrain	15.00	-0.19	-1.49	-1.44
CHRM5	Muscarinic acetylcholine receptor M5	8.020907099	7.744727495	Fetalliver	12.95	-0.34	-2.74	-2.64
CHRM5	Muscarinic acetylcholine receptor M5	8.020907099	7.744727495	Fetallung	11.80	-0.43	-3.44	-3.32

S2 Table. Target-tissue analysis for clozapine and chlorpromazine

Gene Symbol	Gene Name	Affinity (-log10(Ki))		Tissue	Expression		Combined Score (-log10(Ki)*Z)	
		clozapine	chlorpromazine		Raw-numbers	Z-score	clozapine	chlorpromazine
CHRM5	Muscarinic acetylcholine receptor M5	8.020907099	7.744727495	FetalThyroid	18.35	0.07	0.55	0.53
CHRM5	Muscarinic acetylcholine receptor M5	8.020907099	7.744727495	GlobusPallidus	25.05	0.58	4.63	4.47
CHRM5	Muscarinic acetylcholine receptor M5	8.020907099	7.744727495	Heart	23.10	0.43	3.44	3.33
CHRM5	Muscarinic acetylcholine receptor M5	8.020907099	7.744727495	Hypothalamus	17.00	-0.03	-0.27	-0.26
CHRM5	Muscarinic acetylcholine receptor M5	8.020907099	7.744727495	Kidney	15.90	-0.12	-0.94	-0.91
CHRM5	Muscarinic acetylcholine receptor M5	8.020907099	7.744727495	Liver	23.35	0.45	3.60	3.47
CHRM5	Muscarinic acetylcholine receptor M5	8.020907099	7.744727495	Lung	18.10	0.05	0.40	0.39
CHRM5	Muscarinic acetylcholine receptor M5	8.020907099	7.744727495	Lymphnode	12.70	-0.36	-2.89	-2.79
CHRM5	Muscarinic acetylcholine receptor M5	8.020907099	7.744727495	MedullaOblongata	17.50	0.00	0.03	0.03
CHRM5	Muscarinic acetylcholine receptor M5	8.020907099	7.744727495	OccipitalLobe	10.90	-0.50	-3.99	-3.85
CHRM5	Muscarinic acetylcholine receptor M5	8.020907099	7.744727495	OlfactoryBulb	13.30	-0.31	-2.52	-2.44
CHRM5	Muscarinic acetylcholine receptor M5	8.020907099	7.744727495	Ovary	11.65	-0.44	-3.53	-3.41
CHRM5	Muscarinic acetylcholine receptor M5	8.020907099	7.744727495	Pancreas	11.85	-0.42	-3.41	-3.29
CHRM5	Muscarinic acetylcholine receptor M5	8.020907099	7.744727495	PancreaticIslet	16.20	-0.09	-0.76	-0.73
CHRM5	Muscarinic acetylcholine receptor M5	8.020907099	7.744727495	ParietalLobe	24.90	0.57	4.54	4.38
CHRM5	Muscarinic acetylcholine receptor M5	8.020907099	7.744727495	Pineal_day	16.36	-0.08	-0.66	-0.64
CHRM5	Muscarinic acetylcholine receptor M5	8.020907099	7.744727495	Pineal_night	16.96	-0.04	-0.30	-0.29
CHRM5	Muscarinic acetylcholine receptor M5	8.020907099	7.744727495	Pituitary	22.75	0.40	3.23	3.12
CHRM5	Muscarinic acetylcholine receptor M5	8.020907099	7.744727495	Placenta	13.15	-0.33	-2.62	-2.53
CHRM5	Muscarinic acetylcholine receptor M5	8.020907099	7.744727495	Pons	31.30	1.05	8.44	8.15
CHRM5	Muscarinic acetylcholine receptor M5	8.020907099	7.744727495	PrefrontalCortex	18.40	0.07	0.58	0.56
CHRM5	Muscarinic acetylcholine receptor M5	8.020907099	7.744727495	Prostate	16.35	-0.08	-0.67	-0.64
CHRM5	Muscarinic acetylcholine receptor M5	8.020907099	7.744727495	Retina	13.75	-0.28	-2.25	-2.17
CHRM5	Muscarinic acetylcholine receptor M5	8.020907099	7.744727495	Salivarygland	12.55	-0.37	-2.98	-2.88
CHRM5	Muscarinic acetylcholine receptor M5	8.020907099	7.744727495	SkeletalMuscle	122.80	8.00	64.18	61.97
CHRM5	Muscarinic acetylcholine receptor M5	8.020907099	7.744727495	Skin	11.75	-0.43	-3.47	-3.35
CHRM5	Muscarinic acetylcholine receptor M5	8.020907099	7.744727495	Small_intestine	13.05	-0.33	-2.68	-2.59
CHRM5	Muscarinic acetylcholine receptor M5	8.020907099	7.744727495	SmoothMuscle	15.30	-0.16	-1.31	-1.26
CHRM5	Muscarinic acetylcholine receptor M5	8.020907099	7.744727495	Spinalcord	16.00	-0.11	-0.88	-0.85
CHRM5	Muscarinic acetylcholine receptor M5	8.020907099	7.744727495	SubthalamicNucleus	19.85	0.18	1.46	1.41
CHRM5	Muscarinic acetylcholine receptor M5	8.020907099	7.744727495	SuperiorCervicalGanglion	26.35	0.68	5.42	5.24
CHRM5	Muscarinic acetylcholine receptor M5	8.020907099	7.744727495	TemporalLobe	13.80	-0.28	-2.22	-2.14
CHRM5	Muscarinic acetylcholine receptor M5	8.020907099	7.744727495	Testis	21.35	0.30	2.38	2.30
CHRM5	Muscarinic acetylcholine receptor M5	8.020907099	7.744727495	TestisGermCell	11.70	-0.44	-3.50	-3.38
CHRM5	Muscarinic acetylcholine receptor M5	8.020907099	7.744727495	TestisIntersitial	12.40	-0.38	-3.07	-2.97
CHRM5	Muscarinic acetylcholine receptor M5	8.020907099	7.744727495	TestisLeydigCell	17.50	0.00	0.03	0.03
CHRM5	Muscarinic acetylcholine receptor M5	8.020907099	7.744727495	TestisSeminiferousTubule	16.60	-0.06	-0.51	-0.50
CHRM5	Muscarinic acetylcholine receptor M5	8.020907099	7.744727495	Thalamus	14.20	-0.25	-1.98	-1.91
CHRM5	Muscarinic acetylcholine receptor M5	8.020907099	7.744727495	Thymus	10.30	-0.54	-4.35	-4.20
CHRM5	Muscarinic acetylcholine receptor M5	8.020907099	7.744727495	Thyroid	16.80	-0.05	-0.39	-0.38
CHRM5	Muscarinic acetylcholine receptor M5	8.020907099	7.744727495	Tongue	16.80	-0.05	-0.39	-0.38
CHRM5	Muscarinic acetylcholine receptor M5	8.020907099	7.744727495	Tonsil	14.05	-0.26	-2.07	-2.00
CHRM5	Muscarinic acetylcholine receptor M5	8.020907099	7.744727495	Trachea	14.35	-0.24	-1.89	-1.82
CHRM5	Muscarinic acetylcholine receptor M5	8.020907099	7.744727495	TrigeminalGanglion	15.65	-0.14	-1.09	-1.06
CHRM5	Muscarinic acetylcholine receptor M5	8.020907099	7.744727495	Uterus	13.20	-0.32	-2.59	-2.50
CHRM5	Muscarinic acetylcholine receptor M5	8.020907099	7.744727495	UterusCorpus	28.65	0.85	6.83	6.59
CHRM5	Muscarinic acetylcholine receptor M5	8.020907099	7.744727495	WholeBlood	15.35	-0.16	-1.28	-1.23
CHRM5	Muscarinic acetylcholine receptor M5	8.020907099	7.744727495	Wholebrain	10.70	-0.51	-4.11	-3.97
CYP2D6	Cytochrome P450 2D6	n/a	5.15490196	Adipocyte	5.50	-0.20	n/a	-1.01
CYP2D6	Cytochrome P450 2D6	n/a	5.15490196	AdrenalCortex	8.00	2.10	n/a	10.83
CYP2D6	Cytochrome P450 2D6	n/a	5.15490196	Adrenalgland	4.65	-0.98	n/a	-5.03
CYP2D6	Cytochrome P450 2D6	n/a	5.15490196	Amygdala	5.55	-0.15	n/a	-0.77
CYP2D6	Cytochrome P450 2D6	n/a	5.15490196	Appendix	5.75	0.03	n/a	0.18

S2 Table. Target-tissue analysis for clozapine and chlorpromazine

Gene Symbol	Gene Name	Affinity (-log10(Ki))		Tissue	Expression		Combined Score (-log10(Ki)*Z)	
		clozapine	chlorpromazine		Raw-numbers	Z-score	clozapine	chlorpromazine
CYP2D6	Cytochrome P450 2D6	n/a	5.15490196	AtrioventricularNode	5.80	0.08	n/a	0.42
CYP2D6	Cytochrome P450 2D6	n/a	5.15490196	BDCA4+_DentriticCells	6.15	0.40	n/a	2.07
CYP2D6	Cytochrome P450 2D6	n/a	5.15490196	Bonemarrow	5.45	-0.24	n/a	-1.24
CYP2D6	Cytochrome P450 2D6	n/a	5.15490196	BronchialEpithelialCells	5.55	-0.15	n/a	-0.77
CYP2D6	Cytochrome P450 2D6	n/a	5.15490196	CardiacMyocytes	7.40	1.55	n/a	7.99
CYP2D6	Cytochrome P450 2D6	n/a	5.15490196	Caudatenucleus	5.05	-0.61	n/a	-3.14
CYP2D6	Cytochrome P450 2D6	n/a	5.15490196	CD105+_Endothelial	6.30	0.54	n/a	2.78
CYP2D6	Cytochrome P450 2D6	n/a	5.15490196	CD14+_Monocytes	6.00	0.26	n/a	1.36
CYP2D6	Cytochrome P450 2D6	n/a	5.15490196	CD19+_BCells(neg._sel.)	6.20	0.45	n/a	2.31
CYP2D6	Cytochrome P450 2D6	n/a	5.15490196	CD33+_Myeloid	7.25	1.41	n/a	7.28
CYP2D6	Cytochrome P450 2D6	n/a	5.15490196	CD34+	7.05	1.23	n/a	6.33
CYP2D6	Cytochrome P450 2D6	n/a	5.15490196	CD4+_Tcells	6.20	0.45	n/a	2.31
CYP2D6	Cytochrome P450 2D6	n/a	5.15490196	CD56+_NKCells	6.65	0.86	n/a	4.44
CYP2D6	Cytochrome P450 2D6	n/a	5.15490196	CD71+_EarlyErythroid	6.85	1.05	n/a	5.39
CYP2D6	Cytochrome P450 2D6	n/a	5.15490196	CD8+_Tcells	5.55	-0.15	n/a	-0.77
CYP2D6	Cytochrome P450 2D6	n/a	5.15490196	Cerebellum	4.30	-1.30	n/a	-6.69
CYP2D6	Cytochrome P450 2D6	n/a	5.15490196	CerebellumPeduncles	5.95	0.22	n/a	1.13
CYP2D6	Cytochrome P450 2D6	n/a	5.15490196	CiliaryGanglion	3.95	-1.62	n/a	-8.35
CYP2D6	Cytochrome P450 2D6	n/a	5.15490196	CingulateCortex	5.70	-0.01	n/a	-0.06
CYP2D6	Cytochrome P450 2D6	n/a	5.15490196	Colon	5.65	-0.06	n/a	-0.30
CYP2D6	Cytochrome P450 2D6	n/a	5.15490196	DorsalRootGanglion	4.15	-1.44	n/a	-7.40
CYP2D6	Cytochrome P450 2D6	n/a	5.15490196	Fetalbrain	5.40	-0.29	n/a	-1.48
CYP2D6	Cytochrome P450 2D6	n/a	5.15490196	Fetalliver	6.55	0.77	n/a	3.97
CYP2D6	Cytochrome P450 2D6	n/a	5.15490196	Fetallung	4.50	-1.11	n/a	-5.74
CYP2D6	Cytochrome P450 2D6	n/a	5.15490196	FetalThyroid	5.40	-0.29	n/a	-1.48
CYP2D6	Cytochrome P450 2D6	n/a	5.15490196	GlobusPallidus	4.00	-1.57	n/a	-8.11
CYP2D6	Cytochrome P450 2D6	n/a	5.15490196	Heart	7.25	1.41	n/a	7.28
CYP2D6	Cytochrome P450 2D6	n/a	5.15490196	Hypothalamus	5.85	0.13	n/a	0.65
CYP2D6	Cytochrome P450 2D6	n/a	5.15490196	Kidney	4.50	-1.11	n/a	-5.74
CYP2D6	Cytochrome P450 2D6	n/a	5.15490196	Liver	7.35	1.50	n/a	7.76
CYP2D6	Cytochrome P450 2D6	n/a	5.15490196	Lung	6.20	0.45	n/a	2.31
CYP2D6	Cytochrome P450 2D6	n/a	5.15490196	Lymphnode	4.85	-0.79	n/a	-4.08
CYP2D6	Cytochrome P450 2D6	n/a	5.15490196	MedullaOblongata	5.05	-0.61	n/a	-3.14
CYP2D6	Cytochrome P450 2D6	n/a	5.15490196	OccipitalLobe	5.05	-0.61	n/a	-3.14
CYP2D6	Cytochrome P450 2D6	n/a	5.15490196	OlfactoryBulb	4.40	-1.21	n/a	-6.21
CYP2D6	Cytochrome P450 2D6	n/a	5.15490196	Ovary	3.70	-1.85	n/a	-9.53
CYP2D6	Cytochrome P450 2D6	n/a	5.15490196	Pancreas	4.45	-1.16	n/a	-5.98
CYP2D6	Cytochrome P450 2D6	n/a	5.15490196	PancreaticIslet	5.95	0.22	n/a	1.13
CYP2D6	Cytochrome P450 2D6	n/a	5.15490196	ParietalLobe	5.85	0.13	n/a	0.65
CYP2D6	Cytochrome P450 2D6	n/a	5.15490196	Pineal_day	6.92	1.11	n/a	5.72
CYP2D6	Cytochrome P450 2D6	n/a	5.15490196	Pineal_night	7.48	1.62	n/a	8.37
CYP2D6	Cytochrome P450 2D6	n/a	5.15490196	Pituitary	6.50	0.72	n/a	3.73
CYP2D6	Cytochrome P450 2D6	n/a	5.15490196	Placenta	5.85	0.13	n/a	0.65
CYP2D6	Cytochrome P450 2D6	n/a	5.15490196	Pons	5.30	-0.38	n/a	-1.95
CYP2D6	Cytochrome P450 2D6	n/a	5.15490196	PrefrontalCortex	7.05	1.23	n/a	6.33
CYP2D6	Cytochrome P450 2D6	n/a	5.15490196	Prostate	6.45	0.68	n/a	3.49
CYP2D6	Cytochrome P450 2D6	n/a	5.15490196	Retina	7.80	1.92	n/a	9.89
CYP2D6	Cytochrome P450 2D6	n/a	5.15490196	Salivarygland	5.75	0.03	n/a	0.18
CYP2D6	Cytochrome P450 2D6	n/a	5.15490196	SkeletalMuscle	7.95	2.06	n/a	10.60
CYP2D6	Cytochrome P450 2D6	n/a	5.15490196	Skin	5.45	-0.24	n/a	-1.24
CYP2D6	Cytochrome P450 2D6	n/a	5.15490196	Small_intestine	5.45	-0.24	n/a	-1.24
CYP2D6	Cytochrome P450 2D6	n/a	5.15490196	SmoothMuscle	6.35	0.59	n/a	3.02
CYP2D6	Cytochrome P450 2D6	n/a	5.15490196	Spinalcord	5.95	0.22	n/a	1.13

S2 Table. Target-tissue analysis for clozapine and chlorpromazine

Gene Symbol	Gene Name	Affinity (-log10(Ki))		Tissue	Expression		Combined Score (-log10(Ki)*Z)	
		clozapine	chlorpromazine		Raw-numbers	Z-score	clozapine	chlorpromazine
CYP2D6	Cytochrome P450 2D6	n/a	5.15490196	SubthalamicNucleus	5.00	-0.65	n/a	-3.37
CYP2D6	Cytochrome P450 2D6	n/a	5.15490196	SuperiorCervicalGanglion	9.05	3.07	n/a	15.81
CYP2D6	Cytochrome P450 2D6	n/a	5.15490196	TemporalLobe	5.10	-0.56	n/a	-2.90
CYP2D6	Cytochrome P450 2D6	n/a	5.15490196	Testis	4.25	-1.34	n/a	-6.93
CYP2D6	Cytochrome P450 2D6	n/a	5.15490196	TestisGermCell	4.70	-0.93	n/a	-4.79
CYP2D6	Cytochrome P450 2D6	n/a	5.15490196	TestisIntersitial	4.65	-0.98	n/a	-5.03
CYP2D6	Cytochrome P450 2D6	n/a	5.15490196	TestisLeydigCell	5.50	-0.20	n/a	-1.01
CYP2D6	Cytochrome P450 2D6	n/a	5.15490196	TestisSeminiferousTubule	4.75	-0.88	n/a	-4.56
CYP2D6	Cytochrome P450 2D6	n/a	5.15490196	Thalamus	6.50	0.72	n/a	3.73
CYP2D6	Cytochrome P450 2D6	n/a	5.15490196	Thymus	3.80	-1.76	n/a	-9.06
CYP2D6	Cytochrome P450 2D6	n/a	5.15490196	Thyroid	6.00	0.26	n/a	1.36
CYP2D6	Cytochrome P450 2D6	n/a	5.15490196	Tongue	6.05	0.31	n/a	1.60
CYP2D6	Cytochrome P450 2D6	n/a	5.15490196	Tonsil	5.40	-0.29	n/a	-1.48
CYP2D6	Cytochrome P450 2D6	n/a	5.15490196	Trachea	4.65	-0.98	n/a	-5.03
CYP2D6	Cytochrome P450 2D6	n/a	5.15490196	TrigeminalGanglion	5.35	-0.33	n/a	-1.72
CYP2D6	Cytochrome P450 2D6	n/a	5.15490196	Uterus	4.60	-1.02	n/a	-5.27
CYP2D6	Cytochrome P450 2D6	n/a	5.15490196	UterusCorpus	5.35	-0.33	n/a	-1.72
CYP2D6	Cytochrome P450 2D6	n/a	5.15490196	WholeBlood	5.65	-0.06	n/a	-0.30
CYP2D6	Cytochrome P450 2D6	n/a	5.15490196	Wholebrain	4.35	-1.25	n/a	-6.45
DRD1	Dopamine D1 receptor	7.669992299	7.017728767	Adipocyte	6.80	-0.32	-2.47	-2.26
DRD1	Dopamine D1 receptor	7.669992299	7.017728767	AdrenalCortex	7.80	0.29	2.23	2.04
DRD1	Dopamine D1 receptor	7.669992299	7.017728767	Adrenalgland	6.30	-0.63	-4.82	-4.41
DRD1	Dopamine D1 receptor	7.669992299	7.017728767	Amygdala	7.25	-0.05	-0.35	-0.32
DRD1	Dopamine D1 receptor	7.669992299	7.017728767	Appendix	8.30	0.60	4.58	4.19
DRD1	Dopamine D1 receptor	7.669992299	7.017728767	AtrioventricularNode	9.20	1.15	8.81	8.06
DRD1	Dopamine D1 receptor	7.669992299	7.017728767	BDCA4+_DentriticCells	6.95	-0.23	-1.76	-1.61
DRD1	Dopamine D1 receptor	7.669992299	7.017728767	Bonemarrow	7.75	0.26	1.99	1.82
DRD1	Dopamine D1 receptor	7.669992299	7.017728767	BronchialEpithelialCells	6.50	-0.51	-3.88	-3.55
DRD1	Dopamine D1 receptor	7.669992299	7.017728767	CardiacMyocytes	9.95	1.61	12.33	11.28
DRD1	Dopamine D1 receptor	7.669992299	7.017728767	Caudatenucleus	14.00	4.09	31.36	28.69
DRD1	Dopamine D1 receptor	7.669992299	7.017728767	CD105+_Endothelial	6.95	-0.23	-1.76	-1.61
DRD1	Dopamine D1 receptor	7.669992299	7.017728767	CD14+_Monocytes	7.50	0.11	0.82	0.75
DRD1	Dopamine D1 receptor	7.669992299	7.017728767	CD19+_BCells(neg._sel.)	7.15	-0.11	-0.82	-0.75
DRD1	Dopamine D1 receptor	7.669992299	7.017728767	CD33+_Myeloid	8.40	0.66	5.05	4.62
DRD1	Dopamine D1 receptor	7.669992299	7.017728767	CD34+	8.35	0.63	4.81	4.40
DRD1	Dopamine D1 receptor	7.669992299	7.017728767	CD4+_Tcells	7.25	-0.05	-0.35	-0.32
DRD1	Dopamine D1 receptor	7.669992299	7.017728767	CD56+_NKCells	7.50	0.11	0.82	0.75
DRD1	Dopamine D1 receptor	7.669992299	7.017728767	CD71+_EarlyErythroid	6.65	-0.41	-3.17	-2.90
DRD1	Dopamine D1 receptor	7.669992299	7.017728767	CD8+_Tcells	6.35	-0.60	-4.58	-4.19
DRD1	Dopamine D1 receptor	7.669992299	7.017728767	Cerebellum	5.65	-1.03	-7.87	-7.20
DRD1	Dopamine D1 receptor	7.669992299	7.017728767	CerebellumPeduncles	8.80	0.90	6.93	6.34
DRD1	Dopamine D1 receptor	7.669992299	7.017728767	CiliaryGanglion	6.65	-0.41	-3.17	-2.90
DRD1	Dopamine D1 receptor	7.669992299	7.017728767	CingulateCortex	7.40	0.05	0.35	0.32
DRD1	Dopamine D1 receptor	7.669992299	7.017728767	Colon	6.85	-0.29	-2.23	-2.04
DRD1	Dopamine D1 receptor	7.669992299	7.017728767	DorsalRootGanglion	6.55	-0.48	-3.64	-3.33
DRD1	Dopamine D1 receptor	7.669992299	7.017728767	Fetalbrain	6.90	-0.26	-2.00	-1.83
DRD1	Dopamine D1 receptor	7.669992299	7.017728767	Fetalliver	6.10	-0.75	-5.76	-5.27
DRD1	Dopamine D1 receptor	7.669992299	7.017728767	Fetallung	5.50	-1.12	-8.58	-7.85
DRD1	Dopamine D1 receptor	7.669992299	7.017728767	FetalThyroid	6.75	-0.35	-2.70	-2.47
DRD1	Dopamine D1 receptor	7.669992299	7.017728767	GlobusPallidus	5.60	-1.06	-8.11	-7.42
DRD1	Dopamine D1 receptor	7.669992299	7.017728767	Heart	12.00	2.86	21.96	20.09
DRD1	Dopamine D1 receptor	7.669992299	7.017728767	Hypothalamus	7.35	0.01	0.12	0.11
DRD1	Dopamine D1 receptor	7.669992299	7.017728767	Kidney	6.10	-0.75	-5.76	-5.27

S2 Table. Target-tissue analysis for clozapine and chlorpromazine

Gene Symbol	Gene Name	Affinity (-log10(Ki))		Tissue	Expression		Combined Score (-log10(Ki)*Z)	
		clozapine	chlorpromazine		Raw-numbers	Z-score	clozapine	chlorpromazine
DRD1	Dopamine D1 receptor	7.669992299	7.017728767	Liver	9.60	1.39	10.69	9.78
DRD1	Dopamine D1 receptor	7.669992299	7.017728767	Lung	7.50	0.11	0.82	0.75
DRD1	Dopamine D1 receptor	7.669992299	7.017728767	Lymphnode	5.85	-0.90	-6.93	-6.34
DRD1	Dopamine D1 receptor	7.669992299	7.017728767	MedullaOblongata	6.35	-0.60	-4.58	-4.19
DRD1	Dopamine D1 receptor	7.669992299	7.017728767	OccipitalLobe	6.80	-0.32	-2.47	-2.26
DRD1	Dopamine D1 receptor	7.669992299	7.017728767	OlfactoryBulb	5.45	-1.15	-8.81	-8.06
DRD1	Dopamine D1 receptor	7.669992299	7.017728767	Ovary	5.10	-1.36	-10.46	-9.57
DRD1	Dopamine D1 receptor	7.669992299	7.017728767	Pancreas	5.70	-1.00	-7.64	-6.99
DRD1	Dopamine D1 receptor	7.669992299	7.017728767	PancreaticIslet	7.50	0.11	0.82	0.75
DRD1	Dopamine D1 receptor	7.669992299	7.017728767	ParietalLobe	7.70	0.23	1.76	1.61
DRD1	Dopamine D1 receptor	7.669992299	7.017728767	Pineal_day	8.58	0.77	5.89	5.39
DRD1	Dopamine D1 receptor	7.669992299	7.017728767	Pineal_night	8.36	0.63	4.86	4.45
DRD1	Dopamine D1 receptor	7.669992299	7.017728767	Pituitary	8.20	0.54	4.11	3.76
DRD1	Dopamine D1 receptor	7.669992299	7.017728767	Placenta	7.00	-0.20	-1.53	-1.40
DRD1	Dopamine D1 receptor	7.669992299	7.017728767	Pons	13.60	3.84	29.48	26.97
DRD1	Dopamine D1 receptor	7.669992299	7.017728767	PrefrontalCortex	9.20	1.15	8.81	8.06
DRD1	Dopamine D1 receptor	7.669992299	7.017728767	Prostate	7.85	0.32	2.46	2.25
DRD1	Dopamine D1 receptor	7.669992299	7.017728767	Retina	8.38	0.64	4.93	4.51
DRD1	Dopamine D1 receptor	7.669992299	7.017728767	Salivarygland	6.20	-0.69	-5.29	-4.84
DRD1	Dopamine D1 receptor	7.669992299	7.017728767	SkeletalMuscle	8.85	0.93	7.16	6.55
DRD1	Dopamine D1 receptor	7.669992299	7.017728767	Skin	6.75	-0.35	-2.70	-2.47
DRD1	Dopamine D1 receptor	7.669992299	7.017728767	Small_intestine	6.45	-0.54	-4.11	-3.76
DRD1	Dopamine D1 receptor	7.669992299	7.017728767	SmoothMuscle	7.80	0.29	2.23	2.04
DRD1	Dopamine D1 receptor	7.669992299	7.017728767	Spinalcord	7.45	0.08	0.58	0.54
DRD1	Dopamine D1 receptor	7.669992299	7.017728767	SubthalamicNucleus	6.80	-0.32	-2.47	-2.26
DRD1	Dopamine D1 receptor	7.669992299	7.017728767	SuperiorCervicalGanglion	9.35	1.24	9.51	8.70
DRD1	Dopamine D1 receptor	7.669992299	7.017728767	TemporalLobe	6.55	-0.48	-3.64	-3.33
DRD1	Dopamine D1 receptor	7.669992299	7.017728767	Testis	5.85	-0.90	-6.93	-6.34
DRD1	Dopamine D1 receptor	7.669992299	7.017728767	TestisGermCell	5.50	-1.12	-8.58	-7.85
DRD1	Dopamine D1 receptor	7.669992299	7.017728767	TestisIntersitial	5.80	-0.93	-7.17	-6.56
DRD1	Dopamine D1 receptor	7.669992299	7.017728767	TestisLeydigCell	7.05	-0.17	-1.29	-1.18
DRD1	Dopamine D1 receptor	7.669992299	7.017728767	TestisSeminiferousTubule	6.00	-0.81	-6.23	-5.70
DRD1	Dopamine D1 receptor	7.669992299	7.017728767	Thalamus	7.30	-0.02	-0.12	-0.11
DRD1	Dopamine D1 receptor	7.669992299	7.017728767	Thymus	5.10	-1.36	-10.46	-9.57
DRD1	Dopamine D1 receptor	7.669992299	7.017728767	Thyroid	8.15	0.51	3.87	3.54
DRD1	Dopamine D1 receptor	7.669992299	7.017728767	Tongue	7.35	0.01	0.12	0.11
DRD1	Dopamine D1 receptor	7.669992299	7.017728767	Tonsil	6.95	-0.23	-1.76	-1.61
DRD1	Dopamine D1 receptor	7.669992299	7.017728767	Trachea	5.85	-0.90	-6.93	-6.34
DRD1	Dopamine D1 receptor	7.669992299	7.017728767	TrigeminalGanglion	8.05	0.44	3.40	3.11
DRD1	Dopamine D1 receptor	7.669992299	7.017728767	Uterus	5.40	-1.18	-9.05	-8.28
DRD1	Dopamine D1 receptor	7.669992299	7.017728767	UterusCorpus	6.50	-0.51	-3.88	-3.55
DRD1	Dopamine D1 receptor	7.669992299	7.017728767	WholeBlood	7.35	0.01	0.12	0.11
DRD1	Dopamine D1 receptor	7.669992299	7.017728767	Wholebrain	5.85	-0.90	-6.93	-6.34
DRD2	Dopamine D2 receptor	7.552841969	8.522878745	Adipocyte	4.45	-0.05	-0.35	-0.40
DRD2	Dopamine D2 receptor	7.552841969	8.522878745	AdrenalCortex	4.63	0.19	1.41	1.60
DRD2	Dopamine D2 receptor	7.552841969	8.522878745	Adrenalgland	3.65	-1.11	-8.42	-9.50
DRD2	Dopamine D2 receptor	7.552841969	8.522878745	Amygdala	4.55	0.09	0.66	0.74
DRD2	Dopamine D2 receptor	7.552841969	8.522878745	Appendix	5.13	0.85	6.46	7.29
DRD2	Dopamine D2 receptor	7.552841969	8.522878745	AtrioventricularNode	3.45	-1.38	-10.44	-11.78
DRD2	Dopamine D2 receptor	7.552841969	8.522878745	BDCA4+_DentriticCells	4.68	0.25	1.92	2.16
DRD2	Dopamine D2 receptor	7.552841969	8.522878745	Bonemarrow	4.30	-0.25	-1.86	-2.10
DRD2	Dopamine D2 receptor	7.552841969	8.522878745	BronchialEpithelialCells	4.55	0.09	0.66	0.74
DRD2	Dopamine D2 receptor	7.552841969	8.522878745	CardiacMyocytes	5.55	1.42	10.74	12.12

S2 Table. Target-tissue analysis for clozapine and chlorpromazine

Gene Symbol	Gene Name	Affinity (-log10(Ki))		Tissue	Expression		Combined Score (-log10(Ki)*Z)	
		clozapine	chlorpromazine		Raw-numbers	Z-score	clozapine	chlorpromazine
DRD2	Dopamine D2 receptor	7.552841969	8.522878745	Caudatenucleus	5.75	1.69	12.76	14.40
DRD2	Dopamine D2 receptor	7.552841969	8.522878745	CD105+_Endothelial	4.70	0.29	2.17	2.45
DRD2	Dopamine D2 receptor	7.552841969	8.522878745	CD14+_Monocytes	4.58	0.12	0.91	1.03
DRD2	Dopamine D2 receptor	7.552841969	8.522878745	CD19+_BCells(neg._sel.)	4.48	-0.01	-0.10	-0.11
DRD2	Dopamine D2 receptor	7.552841969	8.522878745	CD33+_Myeloid	5.60	1.49	11.25	12.69
DRD2	Dopamine D2 receptor	7.552841969	8.522878745	CD34+	5.48	1.32	9.99	11.27
DRD2	Dopamine D2 receptor	7.552841969	8.522878745	CD4+_Tcells	4.83	0.45	3.43	3.87
DRD2	Dopamine D2 receptor	7.552841969	8.522878745	CD56+_NKCells	5.03	0.72	5.45	6.15
DRD2	Dopamine D2 receptor	7.552841969	8.522878745	CD71+_EarlyErythroid	4.08	-0.55	-4.13	-4.67
DRD2	Dopamine D2 receptor	7.552841969	8.522878745	CD8+_Tcells	4.23	-0.35	-2.62	-2.96
DRD2	Dopamine D2 receptor	7.552841969	8.522878745	Cerebellum	3.53	-1.28	-9.68	-10.93
DRD2	Dopamine D2 receptor	7.552841969	8.522878745	CerebellumPeduncles	4.95	0.62	4.69	5.29
DRD2	Dopamine D2 receptor	7.552841969	8.522878745	CiliaryGanglion	3.13	-1.82	-13.72	-15.48
DRD2	Dopamine D2 receptor	7.552841969	8.522878745	CingulateCortex	4.88	0.52	3.94	4.44
DRD2	Dopamine D2 receptor	7.552841969	8.522878745	Colon	4.23	-0.35	-2.62	-2.96
DRD2	Dopamine D2 receptor	7.552841969	8.522878745	DorsalRootGanglion	3.55	-1.25	-9.43	-10.64
DRD2	Dopamine D2 receptor	7.552841969	8.522878745	Fetalbrain	4.40	-0.11	-0.86	-0.97
DRD2	Dopamine D2 receptor	7.552841969	8.522878745	Fetalliver	3.83	-0.88	-6.66	-7.51
DRD2	Dopamine D2 receptor	7.552841969	8.522878745	Fetallung	3.68	-1.08	-8.17	-9.22
DRD2	Dopamine D2 receptor	7.552841969	8.522878745	FetalThyroid	4.70	0.29	2.17	2.45
DRD2	Dopamine D2 receptor	7.552841969	8.522878745	GlobusPallidus	3.18	-1.75	-13.21	-14.91
DRD2	Dopamine D2 receptor	7.552841969	8.522878745	Heart	5.78	1.72	13.01	14.69
DRD2	Dopamine D2 receptor	7.552841969	8.522878745	Hypothalamus	4.80	0.42	3.18	3.59
DRD2	Dopamine D2 receptor	7.552841969	8.522878745	Kidney	3.53	-1.28	-9.68	-10.93
DRD2	Dopamine D2 receptor	7.552841969	8.522878745	Liver	5.95	1.96	14.78	16.68
DRD2	Dopamine D2 receptor	7.552841969	8.522878745	Lung	5.35	1.16	8.73	9.85
DRD2	Dopamine D2 receptor	7.552841969	8.522878745	Lymphnode	3.78	-0.95	-7.16	-8.08
DRD2	Dopamine D2 receptor	7.552841969	8.522878745	MedullaOblongata	4.85	0.49	3.68	4.16
DRD2	Dopamine D2 receptor	7.552841969	8.522878745	OccipitalLobe	3.93	-0.75	-5.65	-6.37
DRD2	Dopamine D2 receptor	7.552841969	8.522878745	OlfactoryBulb	3.45	-1.38	-10.44	-11.78
DRD2	Dopamine D2 receptor	7.552841969	8.522878745	Ovary	3.30	-1.58	-11.95	-13.49
DRD2	Dopamine D2 receptor	7.552841969	8.522878745	Pancreas	3.55	-1.25	-9.43	-10.64
DRD2	Dopamine D2 receptor	7.552841969	8.522878745	PancreaticIslet	4.68	0.25	1.92	2.16
DRD2	Dopamine D2 receptor	7.552841969	8.522878745	ParietalLobe	4.60	0.15	1.16	1.31
DRD2	Dopamine D2 receptor	7.552841969	8.522878745	Pineal_day	5.38	1.20	9.03	10.19
DRD2	Dopamine D2 receptor	7.552841969	8.522878745	Pineal_night	5.19	0.94	7.11	8.03
DRD2	Dopamine D2 receptor	7.552841969	8.522878745	Pituitary	6.23	2.32	17.55	19.81
DRD2	Dopamine D2 receptor	7.552841969	8.522878745	Placenta	4.50	0.02	0.15	0.17
DRD2	Dopamine D2 receptor	7.552841969	8.522878745	Pons	5.13	0.85	6.46	7.29
DRD2	Dopamine D2 receptor	7.552841969	8.522878745	PrefrontalCortex	5.45	1.29	9.74	10.99
DRD2	Dopamine D2 receptor	7.552841969	8.522878745	Prostate	5.30	1.09	8.22	9.28
DRD2	Dopamine D2 receptor	7.552841969	8.522878745	Retina	5.56	1.44	10.87	12.27
DRD2	Dopamine D2 receptor	7.552841969	8.522878745	Salivarygland	3.53	-1.28	-9.68	-10.93
DRD2	Dopamine D2 receptor	7.552841969	8.522878745	SkeletalMuscle	5.08	0.79	5.95	6.72
DRD2	Dopamine D2 receptor	7.552841969	8.522878745	Skin	3.40	-1.45	-10.94	-12.35
DRD2	Dopamine D2 receptor	7.552841969	8.522878745	Small_intestine	4.08	-0.55	-4.13	-4.67
DRD2	Dopamine D2 receptor	7.552841969	8.522878745	SmoothMuscle	5.55	1.42	10.74	12.12
DRD2	Dopamine D2 receptor	7.552841969	8.522878745	Spinalcord	4.68	0.25	1.92	2.16
DRD2	Dopamine D2 receptor	7.552841969	8.522878745	SubthalamicNucleus	4.53	0.05	0.41	0.46
DRD2	Dopamine D2 receptor	7.552841969	8.522878745	SuperiorCervicalGanglion	5.28	1.06	7.97	8.99
DRD2	Dopamine D2 receptor	7.552841969	8.522878745	TemporalLobe	4.00	-0.65	-4.89	-5.52
DRD2	Dopamine D2 receptor	7.552841969	8.522878745	Testis	3.88	-0.81	-6.15	-6.94
DRD2	Dopamine D2 receptor	7.552841969	8.522878745	TestisGermCell	4.03	-0.61	-4.64	-5.23

S2 Table. Target-tissue analysis for clozapine and chlorpromazine

Gene Symbol	Gene Name	Affinity (-log10(Ki))		Tissue	Expression		Combined Score (-log10(Ki)*Z)	
		clozapine	chlorpromazine		Raw-numbers	Z-score	clozapine	chlorpromazine
DRD2	Dopamine D2 receptor	7.552841969	8.522878745	TestisIntersitial	3.93	-0.75	-5.65	-6.37
DRD2	Dopamine D2 receptor	7.552841969	8.522878745	TestisLeydigCell	4.80	0.42	3.18	3.59
DRD2	Dopamine D2 receptor	7.552841969	8.522878745	TestisSeminiferousTubule	3.95	-0.71	-5.39	-6.09
DRD2	Dopamine D2 receptor	7.552841969	8.522878745	Thalamus	4.63	0.19	1.41	1.60
DRD2	Dopamine D2 receptor	7.552841969	8.522878745	Thymus	3.63	-1.15	-8.67	-9.79
DRD2	Dopamine D2 receptor	7.552841969	8.522878745	Thyroid	5.50	1.36	10.24	11.56
DRD2	Dopamine D2 receptor	7.552841969	8.522878745	Tongue	4.50	0.02	0.15	0.17
DRD2	Dopamine D2 receptor	7.552841969	8.522878745	Tonsil	4.33	-0.21	-1.61	-1.82
DRD2	Dopamine D2 receptor	7.552841969	8.522878745	Trachea	3.60	-1.18	-8.93	-10.07
DRD2	Dopamine D2 receptor	7.552841969	8.522878745	TrigeminalGanglion	4.50	0.02	0.15	0.17
DRD2	Dopamine D2 receptor	7.552841969	8.522878745	Uterus	3.60	-1.18	-8.93	-10.07
DRD2	Dopamine D2 receptor	7.552841969	8.522878745	UterusCorpus	3.95	-0.71	-5.39	-6.09
DRD2	Dopamine D2 receptor	7.552841969	8.522878745	WholeBlood	4.80	0.42	3.18	3.59
DRD2	Dopamine D2 receptor	7.552841969	8.522878745	Wholebrain	3.70	-1.05	-7.92	-8.93
DRD3	Dopamine D3 receptor	7.055517328	8.522878745	Adipocyte	5.55	-0.16	-1.12	-1.35
DRD3	Dopamine D3 receptor	7.055517328	8.522878745	AdrenalCortex	6.70	0.92	6.48	7.83
DRD3	Dopamine D3 receptor	7.055517328	8.522878745	Adrenalgland	5.00	-0.67	-4.75	-5.74
DRD3	Dopamine D3 receptor	7.055517328	8.522878745	Amygdala	5.55	-0.16	-1.12	-1.35
DRD3	Dopamine D3 receptor	7.055517328	8.522878745	Appendix	6.20	0.45	3.18	3.84
DRD3	Dopamine D3 receptor	7.055517328	8.522878745	AtrioventricularNode	5.95	0.22	1.52	1.84
DRD3	Dopamine D3 receptor	7.055517328	8.522878745	BDCA4+_DentriticCells	6.08	0.33	2.35	2.84
DRD3	Dopamine D3 receptor	7.055517328	8.522878745	Bonemarrow	6.33	0.57	4.00	4.83
DRD3	Dopamine D3 receptor	7.055517328	8.522878745	BronchialEpithelialCells	5.43	-0.28	-1.94	-2.35
DRD3	Dopamine D3 receptor	7.055517328	8.522878745	CardiacMyocytes	8.98	3.05	21.51	25.98
DRD3	Dopamine D3 receptor	7.055517328	8.522878745	Caudatenucleus	5.03	-0.65	-4.59	-5.54
DRD3	Dopamine D3 receptor	7.055517328	8.522878745	CD105+_Endothelial	5.60	-0.11	-0.79	-0.95
DRD3	Dopamine D3 receptor	7.055517328	8.522878745	CD14+_Monocytes	6.10	0.36	2.52	3.04
DRD3	Dopamine D3 receptor	7.055517328	8.522878745	CD19+_BCells(neg._sel.)	5.85	0.12	0.86	1.04
DRD3	Dopamine D3 receptor	7.055517328	8.522878745	CD33+_Myeloid	7.30	1.48	10.44	12.61
DRD3	Dopamine D3 receptor	7.055517328	8.522878745	CD34+	6.83	1.04	7.30	8.82
DRD3	Dopamine D3 receptor	7.055517328	8.522878745	CD4+_Tcells	6.13	0.38	2.68	3.24
DRD3	Dopamine D3 receptor	7.055517328	8.522878745	CD56+_NKCells	6.35	0.59	4.17	5.03
DRD3	Dopamine D3 receptor	7.055517328	8.522878745	CD71+_EarlyErythroid	5.48	-0.23	-1.61	-1.95
DRD3	Dopamine D3 receptor	7.055517328	8.522878745	CD8+_Tcells	5.15	-0.53	-3.76	-4.54
DRD3	Dopamine D3 receptor	7.055517328	8.522878745	Cerebellum	4.53	-1.12	-7.89	-9.53
DRD3	Dopamine D3 receptor	7.055517328	8.522878745	CerebellumPeduncles	7.00	1.20	8.46	10.22
DRD3	Dopamine D3 receptor	7.055517328	8.522878745	CiliaryGanglion	4.53	-1.12	-7.89	-9.53
DRD3	Dopamine D3 receptor	7.055517328	8.522878745	CingulateCortex	5.73	0.01	0.04	0.05
DRD3	Dopamine D3 receptor	7.055517328	8.522878745	Colon	5.68	-0.04	-0.29	-0.35
DRD3	Dopamine D3 receptor	7.055517328	8.522878745	DorsalRootGanglion	4.93	-0.74	-5.25	-6.34
DRD3	Dopamine D3 receptor	7.055517328	8.522878745	Fetalbrain	5.63	-0.09	-0.62	-0.75
DRD3	Dopamine D3 receptor	7.055517328	8.522878745	Fetalliver	4.98	-0.70	-4.92	-5.94
DRD3	Dopamine D3 receptor	7.055517328	8.522878745	Fetallung	4.33	-1.31	-9.21	-11.12
DRD3	Dopamine D3 receptor	7.055517328	8.522878745	FetalThyroid	5.50	-0.21	-1.45	-1.75
DRD3	Dopamine D3 receptor	7.055517328	8.522878745	GlobusPallidus	4.70	-0.95	-6.73	-8.13
DRD3	Dopamine D3 receptor	7.055517328	8.522878745	Heart	10.08	4.08	28.77	34.75
DRD3	Dopamine D3 receptor	7.055517328	8.522878745	Hypothalamus	5.88	0.15	1.03	1.24
DRD3	Dopamine D3 receptor	7.055517328	8.522878745	Kidney	5.05	-0.63	-4.42	-5.34
DRD3	Dopamine D3 receptor	7.055517328	8.522878745	Liver	8.60	2.70	19.03	22.99
DRD3	Dopamine D3 receptor	7.055517328	8.522878745	Lung	6.18	0.43	3.01	3.64
DRD3	Dopamine D3 receptor	7.055517328	8.522878745	Lymphnode	4.83	-0.84	-5.91	-7.13
DRD3	Dopamine D3 receptor	7.055517328	8.522878745	MedullaOblongata	4.95	-0.72	-5.08	-6.14
DRD3	Dopamine D3 receptor	7.055517328	8.522878745	OccipitalLobe	5.05	-0.63	-4.42	-5.34

S2 Table. Target-tissue analysis for clozapine and chlorpromazine

Gene Symbol	Gene Name	Affinity (-log10(Ki))		Tissue	Expression		Combined Score (-log10(Ki)*Z)	
		clozapine	chlorpromazine		Raw-numbers	Z-score	clozapine	chlorpromazine
DRD3	Dopamine D3 receptor	7.055517328	8.522878745	OlfactoryBulb	4.40	-1.23	-8.71	-10.53
DRD3	Dopamine D3 receptor	7.055517328	8.522878745	Ovary	3.83	-1.77	-12.51	-15.11
DRD3	Dopamine D3 receptor	7.055517328	8.522878745	Pancreas	4.53	-1.12	-7.89	-9.53
DRD3	Dopamine D3 receptor	7.055517328	8.522878745	PancreaticIslet	6.00	0.26	1.85	2.24
DRD3	Dopamine D3 receptor	7.055517328	8.522878745	ParietalLobe	6.18	0.43	3.01	3.64
DRD3	Dopamine D3 receptor	7.055517328	8.522878745	Pineal_day	6.94	1.14	8.06	9.74
DRD3	Dopamine D3 receptor	7.055517328	8.522878745	Pineal_night	6.70	0.92	6.48	7.83
DRD3	Dopamine D3 receptor	7.055517328	8.522878745	Pituitary	6.45	0.68	4.83	5.83
DRD3	Dopamine D3 receptor	7.055517328	8.522878745	Placenta	5.68	-0.04	-0.29	-0.35
DRD3	Dopamine D3 receptor	7.055517328	8.522878745	Pons	5.53	-0.18	-1.28	-1.55
DRD3	Dopamine D3 receptor	7.055517328	8.522878745	PrefrontalCortex	6.55	0.78	5.49	6.63
DRD3	Dopamine D3 receptor	7.055517328	8.522878745	Prostate	6.45	0.68	4.83	5.83
DRD3	Dopamine D3 receptor	7.055517328	8.522878745	Retina	6.61	0.84	5.90	7.13
DRD3	Dopamine D3 receptor	7.055517328	8.522878745	Salivarygland	4.53	-1.12	-7.89	-9.53
DRD3	Dopamine D3 receptor	7.055517328	8.522878745	SkeletalMuscle	6.58	0.80	5.65	6.83
DRD3	Dopamine D3 receptor	7.055517328	8.522878745	Skin	4.63	-1.02	-7.23	-8.73
DRD3	Dopamine D3 receptor	7.055517328	8.522878745	Small_intestine	5.43	-0.28	-1.94	-2.35
DRD3	Dopamine D3 receptor	7.055517328	8.522878745	SmoothMuscle	6.30	0.54	3.84	4.63
DRD3	Dopamine D3 receptor	7.055517328	8.522878745	Spinalcord	5.98	0.24	1.69	2.04
DRD3	Dopamine D3 receptor	7.055517328	8.522878745	SubthalamicNucleus	5.48	-0.23	-1.61	-1.95
DRD3	Dopamine D3 receptor	7.055517328	8.522878745	SuperiorCervicalGanglion	6.50	0.73	5.16	6.23
DRD3	Dopamine D3 receptor	7.055517328	8.522878745	TemporalLobe	5.20	-0.49	-3.43	-4.14
DRD3	Dopamine D3 receptor	7.055517328	8.522878745	Testis	4.83	-0.84	-5.91	-7.13
DRD3	Dopamine D3 receptor	7.055517328	8.522878745	TestisGermCell	4.45	-1.19	-8.38	-10.13
DRD3	Dopamine D3 receptor	7.055517328	8.522878745	TestisIntersitial	4.68	-0.98	-6.90	-8.33
DRD3	Dopamine D3 receptor	7.055517328	8.522878745	TestisLeydigCell	5.83	0.10	0.70	0.84
DRD3	Dopamine D3 receptor	7.055517328	8.522878745	TestisSeminiferousTubule	4.83	-0.84	-5.91	-7.13
DRD3	Dopamine D3 receptor	7.055517328	8.522878745	Thalamus	5.75	0.03	0.20	0.25
DRD3	Dopamine D3 receptor	7.055517328	8.522878745	Thymus	4.23	-1.40	-9.87	-11.92
DRD3	Dopamine D3 receptor	7.055517328	8.522878745	Thyroid	6.75	0.97	6.81	8.22
DRD3	Dopamine D3 receptor	7.055517328	8.522878745	Tongue	6.58	0.80	5.65	6.83
DRD3	Dopamine D3 receptor	7.055517328	8.522878745	Tonsil	5.50	-0.21	-1.45	-1.75
DRD3	Dopamine D3 receptor	7.055517328	8.522878745	Trachea	4.70	-0.95	-6.73	-8.13
DRD3	Dopamine D3 receptor	7.055517328	8.522878745	TrigeminalGanglion	5.93	0.19	1.36	1.64
DRD3	Dopamine D3 receptor	7.055517328	8.522878745	Uterus	4.55	-1.09	-7.72	-9.33
DRD3	Dopamine D3 receptor	7.055517328	8.522878745	UterusCorpus	5.08	-0.60	-4.25	-5.14
DRD3	Dopamine D3 receptor	7.055517328	8.522878745	WholeBlood	6.18	0.43	3.01	3.64
DRD3	Dopamine D3 receptor	7.055517328	8.522878745	Wholebrain	4.48	-1.16	-8.22	-9.93
DRD4	Dopamine D4 receptor	8.045757491	6.319664487	Adipocyte	2.80	-0.17	-1.38	-1.08
DRD4	Dopamine D4 receptor	8.045757491	6.319664487	AdrenalCortex	2.90	-0.15	-1.18	-0.93
DRD4	Dopamine D4 receptor	8.045757491	6.319664487	Adrenalgland	2.35	-0.28	-2.27	-1.79
DRD4	Dopamine D4 receptor	8.045757491	6.319664487	Amygdala	3.00	-0.12	-0.98	-0.77
DRD4	Dopamine D4 receptor	8.045757491	6.319664487	Appendix	2.80	-0.17	-1.38	-1.08
DRD4	Dopamine D4 receptor	8.045757491	6.319664487	AtrioventricularNode	2.70	-0.20	-1.58	-1.24
DRD4	Dopamine D4 receptor	8.045757491	6.319664487	BDCA4+_DentriticCells	5.50	0.50	3.99	3.14
DRD4	Dopamine D4 receptor	8.045757491	6.319664487	Bonemarrow	2.75	-0.18	-1.48	-1.16
DRD4	Dopamine D4 receptor	8.045757491	6.319664487	BronchialEpithelialCells	2.85	-0.16	-1.28	-1.00
DRD4	Dopamine D4 receptor	8.045757491	6.319664487	CardiacMyocytes	3.55	0.01	0.11	0.09
DRD4	Dopamine D4 receptor	8.045757491	6.319664487	Caudatenucleus	2.55	-0.23	-1.88	-1.47
DRD4	Dopamine D4 receptor	8.045757491	6.319664487	CD105+_Endothelial	2.90	-0.15	-1.18	-0.93
DRD4	Dopamine D4 receptor	8.045757491	6.319664487	CD14+_Monocytes	3.05	-0.11	-0.88	-0.69
DRD4	Dopamine D4 receptor	8.045757491	6.319664487	CD19+_BCells(neg._sel.)	3.05	-0.11	-0.88	-0.69
DRD4	Dopamine D4 receptor	8.045757491	6.319664487	CD33+_Myeloid	3.70	0.05	0.41	0.32

S2 Table. Target-tissue analysis for clozapine and chlorpromazine

Gene Symbol	Gene Name	Affinity (-log10(Ki))		Tissue	Expression		Combined Score (-log10(Ki)*Z)	
		clozapine	chlorpromazine		Raw-numbers	Z-score	clozapine	chlorpromazine
DRD4	Dopamine D4 receptor	8.045757491	6.319664487	CD34+	3.70	0.05	0.41	0.32
DRD4	Dopamine D4 receptor	8.045757491	6.319664487	CD4+_Tcells	3.15	-0.08	-0.68	-0.54
DRD4	Dopamine D4 receptor	8.045757491	6.319664487	CD56+_NKCells	3.45	-0.01	-0.09	-0.07
DRD4	Dopamine D4 receptor	8.045757491	6.319664487	CD71+_EarlyErythroid	2.65	-0.21	-1.68	-1.32
DRD4	Dopamine D4 receptor	8.045757491	6.319664487	CD8+_Tcells	2.80	-0.17	-1.38	-1.08
DRD4	Dopamine D4 receptor	8.045757491	6.319664487	Cerebellum	2.30	-0.29	-2.37	-1.86
DRD4	Dopamine D4 receptor	8.045757491	6.319664487	CerebellumPeduncles	3.15	-0.08	-0.68	-0.54
DRD4	Dopamine D4 receptor	8.045757491	6.319664487	CiliaryGanglion	1.95	-0.38	-3.07	-2.41
DRD4	Dopamine D4 receptor	8.045757491	6.319664487	CingulateCortex	2.90	-0.15	-1.18	-0.93
DRD4	Dopamine D4 receptor	8.045757491	6.319664487	Colon	2.85	-0.16	-1.28	-1.00
DRD4	Dopamine D4 receptor	8.045757491	6.319664487	DorsalRootGanglion	2.15	-0.33	-2.67	-2.10
DRD4	Dopamine D4 receptor	8.045757491	6.319664487	Fetalbrain	2.90	-0.15	-1.18	-0.93
DRD4	Dopamine D4 receptor	8.045757491	6.319664487	Fetalliver	2.40	-0.27	-2.17	-1.71
DRD4	Dopamine D4 receptor	8.045757491	6.319664487	Fetallung	2.40	-0.27	-2.17	-1.71
DRD4	Dopamine D4 receptor	8.045757491	6.319664487	FetalThyroid	2.75	-0.18	-1.48	-1.16
DRD4	Dopamine D4 receptor	8.045757491	6.319664487	GlobusPallidus	2.05	-0.36	-2.87	-2.25
DRD4	Dopamine D4 receptor	8.045757491	6.319664487	Heart	5.00	0.37	3.00	2.35
DRD4	Dopamine D4 receptor	8.045757491	6.319664487	Hypothalamus	3.10	-0.10	-0.78	-0.61
DRD4	Dopamine D4 receptor	8.045757491	6.319664487	Kidney	2.25	-0.31	-2.47	-1.94
DRD4	Dopamine D4 receptor	8.045757491	6.319664487	Liver	3.65	0.04	0.31	0.25
DRD4	Dopamine D4 receptor	8.045757491	6.319664487	Lung	3.20	-0.07	-0.58	-0.46
DRD4	Dopamine D4 receptor	8.045757491	6.319664487	Lymphnode	2.50	-0.25	-1.97	-1.55
DRD4	Dopamine D4 receptor	8.045757491	6.319664487	MedullaOblongata	2.60	-0.22	-1.78	-1.39
DRD4	Dopamine D4 receptor	8.045757491	6.319664487	OccipitalLobe	2.55	-0.23	-1.88	-1.47
DRD4	Dopamine D4 receptor	8.045757491	6.319664487	OlfactoryBulb	2.25	-0.31	-2.47	-1.94
DRD4	Dopamine D4 receptor	8.045757491	6.319664487	Ovary	1.85	-0.41	-3.27	-2.57
DRD4	Dopamine D4 receptor	8.045757491	6.319664487	Pancreas	2.25	-0.31	-2.47	-1.94
DRD4	Dopamine D4 receptor	8.045757491	6.319664487	PancreaticIslet	3.00	-0.12	-0.98	-0.77
DRD4	Dopamine D4 receptor	8.045757491	6.319664487	ParietalLobe	2.95	-0.13	-1.08	-0.85
DRD4	Dopamine D4 receptor	8.045757491	6.319664487	Pineal_day	31.08	6.82	54.87	43.10
DRD4	Dopamine D4 receptor	8.045757491	6.319664487	Pineal_night	24.12	5.10	41.03	32.23
DRD4	Dopamine D4 receptor	8.045757491	6.319664487	Pituitary	3.30	-0.05	-0.38	-0.30
DRD4	Dopamine D4 receptor	8.045757491	6.319664487	Placenta	2.95	-0.13	-1.08	-0.85
DRD4	Dopamine D4 receptor	8.045757491	6.319664487	Pons	2.65	-0.21	-1.68	-1.32
DRD4	Dopamine D4 receptor	8.045757491	6.319664487	PrefrontalCortex	3.60	0.03	0.21	0.17
DRD4	Dopamine D4 receptor	8.045757491	6.319664487	Prostate	3.35	-0.04	-0.28	-0.22
DRD4	Dopamine D4 receptor	8.045757491	6.319664487	Retina	3.50	0.00	0.01	0.01
DRD4	Dopamine D4 receptor	8.045757491	6.319664487	Salivarygland	2.20	-0.32	-2.57	-2.02
DRD4	Dopamine D4 receptor	8.045757491	6.319664487	SkeletalMuscle	3.25	-0.06	-0.48	-0.38
DRD4	Dopamine D4 receptor	8.045757491	6.319664487	Skin	2.15	-0.33	-2.67	-2.10
DRD4	Dopamine D4 receptor	8.045757491	6.319664487	Small_intestine	2.75	-0.18	-1.48	-1.16
DRD4	Dopamine D4 receptor	8.045757491	6.319664487	SmoothMuscle	3.25	-0.06	-0.48	-0.38
DRD4	Dopamine D4 receptor	8.045757491	6.319664487	Spinalcord	3.05	-0.11	-0.88	-0.69
DRD4	Dopamine D4 receptor	8.045757491	6.319664487	SubthalamicNucleus	2.55	-0.23	-1.88	-1.47
DRD4	Dopamine D4 receptor	8.045757491	6.319664487	SuperiorCervicalGanglion	3.15	-0.08	-0.68	-0.54
DRD4	Dopamine D4 receptor	8.045757491	6.319664487	TemporalLobe	2.55	-0.23	-1.88	-1.47
DRD4	Dopamine D4 receptor	8.045757491	6.319664487	Testis	2.45	-0.26	-2.07	-1.63
DRD4	Dopamine D4 receptor	8.045757491	6.319664487	TestisGermCell	2.40	-0.27	-2.17	-1.71
DRD4	Dopamine D4 receptor	8.045757491	6.319664487	TestisIntersitial	2.40	-0.27	-2.17	-1.71
DRD4	Dopamine D4 receptor	8.045757491	6.319664487	TestisLeydigCell	2.80	-0.17	-1.38	-1.08
DRD4	Dopamine D4 receptor	8.045757491	6.319664487	TestisSeminiferousTubule	2.45	-0.26	-2.07	-1.63
DRD4	Dopamine D4 receptor	8.045757491	6.319664487	Thalamus	2.90	-0.15	-1.18	-0.93
DRD4	Dopamine D4 receptor	8.045757491	6.319664487	Thymus	2.25	-0.31	-2.47	-1.94

S2 Table. Target-tissue analysis for clozapine and chlorpromazine

Gene Symbol	Gene Name	Affinity (-log10(Ki))		Tissue	Expression		Combined Score (-log10(Ki)*Z)	
		clozapine	chlorpromazine		Raw-numbers	Z-score	clozapine	chlorpromazine
DRD4	Dopamine D4 receptor	8.045757491	6.319664487	Thyroid	3.55	0.01	0.11	0.09
DRD4	Dopamine D4 receptor	8.045757491	6.319664487	Tongue	2.75	-0.18	-1.48	-1.16
DRD4	Dopamine D4 receptor	8.045757491	6.319664487	Tonsil	2.75	-0.18	-1.48	-1.16
DRD4	Dopamine D4 receptor	8.045757491	6.319664487	Trachea	2.40	-0.27	-2.17	-1.71
DRD4	Dopamine D4 receptor	8.045757491	6.319664487	TrigeminalGanglion	2.80	-0.17	-1.38	-1.08
DRD4	Dopamine D4 receptor	8.045757491	6.319664487	Uterus	2.35	-0.28	-2.27	-1.79
DRD4	Dopamine D4 receptor	8.045757491	6.319664487	UterusCorpus	2.50	-0.25	-1.97	-1.55
DRD4	Dopamine D4 receptor	8.045757491	6.319664487	WholeBlood	3.15	-0.08	-0.68	-0.54
DRD4	Dopamine D4 receptor	8.045757491	6.319664487	Wholebrain	2.45	-0.26	-2.07	-1.63
DRD5	Dopamine D5 receptor	6.70333481	6.764471553	Adipocyte	7.45	-0.17	-1.14	-1.15
DRD5	Dopamine D5 receptor	6.70333481	6.764471553	AdrenalCortex	6.65	-0.49	-3.29	-3.32
DRD5	Dopamine D5 receptor	6.70333481	6.764471553	Adrenalgland	5.90	-0.79	-5.31	-5.35
DRD5	Dopamine D5 receptor	6.70333481	6.764471553	Amygdala	7.35	-0.21	-1.41	-1.42
DRD5	Dopamine D5 receptor	6.70333481	6.764471553	Appendix	9.65	0.71	4.77	4.82
DRD5	Dopamine D5 receptor	6.70333481	6.764471553	AtrioventricularNode	5.20	-1.07	-7.19	-7.25
DRD5	Dopamine D5 receptor	6.70333481	6.764471553	BDCA4+_DentriticCells	6.75	-0.45	-3.02	-3.05
DRD5	Dopamine D5 receptor	6.70333481	6.764471553	Bonemarrow	8.40	0.21	1.41	1.43
DRD5	Dopamine D5 receptor	6.70333481	6.764471553	BronchialEpithelialCells	6.15	-0.69	-4.63	-4.68
DRD5	Dopamine D5 receptor	6.70333481	6.764471553	CardiacMyocytes	8.55	0.27	1.82	1.83
DRD5	Dopamine D5 receptor	6.70333481	6.764471553	Caudatenucleus	8.20	0.13	0.88	0.89
DRD5	Dopamine D5 receptor	6.70333481	6.764471553	CD105+_Endothelial	5.80	-0.83	-5.57	-5.62
DRD5	Dopamine D5 receptor	6.70333481	6.764471553	CD14+_Monocytes	5.75	-0.85	-5.71	-5.76
DRD5	Dopamine D5 receptor	6.70333481	6.764471553	CD19+_BCells(neg._sel.)	6.40	-0.59	-3.96	-4.00
DRD5	Dopamine D5 receptor	6.70333481	6.764471553	CD33+_Myeloid	7.95	0.03	0.21	0.21
DRD5	Dopamine D5 receptor	6.70333481	6.764471553	CD34+	8.15	0.11	0.74	0.75
DRD5	Dopamine D5 receptor	6.70333481	6.764471553	CD4+_Tcells	6.70	-0.47	-3.15	-3.18
DRD5	Dopamine D5 receptor	6.70333481	6.764471553	CD56+_NKCells	7.90	0.01	0.07	0.07
DRD5	Dopamine D5 receptor	6.70333481	6.764471553	CD71+_EarlyErythroid	7.25	-0.25	-1.68	-1.69
DRD5	Dopamine D5 receptor	6.70333481	6.764471553	CD8+_Tcells	4.80	-1.23	-8.26	-8.34
DRD5	Dopamine D5 receptor	6.70333481	6.764471553	Cerebellum	8.90	0.41	2.76	2.78
DRD5	Dopamine D5 receptor	6.70333481	6.764471553	CerebellumPeduncles	8.20	0.13	0.88	0.89
DRD5	Dopamine D5 receptor	6.70333481	6.764471553	CiliaryGanglion	6.20	-0.67	-4.50	-4.54
DRD5	Dopamine D5 receptor	6.70333481	6.764471553	CingulateCortex	10.35	0.99	6.66	6.72
DRD5	Dopamine D5 receptor	6.70333481	6.764471553	Colon	9.65	0.71	4.77	4.82
DRD5	Dopamine D5 receptor	6.70333481	6.764471553	DorsalRootGanglion	6.80	-0.43	-2.89	-2.91
DRD5	Dopamine D5 receptor	6.70333481	6.764471553	Fetalbrain	9.80	0.77	5.18	5.22
DRD5	Dopamine D5 receptor	6.70333481	6.764471553	Fetalliver	16.25	3.36	22.51	22.72
DRD5	Dopamine D5 receptor	6.70333481	6.764471553	Fetallung	5.30	-1.03	-6.92	-6.98
DRD5	Dopamine D5 receptor	6.70333481	6.764471553	FetalThyroid	7.55	-0.13	-0.87	-0.88
DRD5	Dopamine D5 receptor	6.70333481	6.764471553	GlobusPallidus	6.45	-0.57	-3.83	-3.86
DRD5	Dopamine D5 receptor	6.70333481	6.764471553	Heart	19.40	4.62	30.98	31.26
DRD5	Dopamine D5 receptor	6.70333481	6.764471553	Hypothalamus	7.50	-0.15	-1.00	-1.01
DRD5	Dopamine D5 receptor	6.70333481	6.764471553	Kidney	6.65	-0.49	-3.29	-3.32
DRD5	Dopamine D5 receptor	6.70333481	6.764471553	Liver	15.40	3.02	20.23	20.41
DRD5	Dopamine D5 receptor	6.70333481	6.764471553	Lung	6.90	-0.39	-2.62	-2.64
DRD5	Dopamine D5 receptor	6.70333481	6.764471553	Lymphnode	6.35	-0.61	-4.10	-4.13
DRD5	Dopamine D5 receptor	6.70333481	6.764471553	MedullaOblongata	7.15	-0.29	-1.95	-1.96
DRD5	Dopamine D5 receptor	6.70333481	6.764471553	OccipitalLobe	5.75	-0.85	-5.71	-5.76
DRD5	Dopamine D5 receptor	6.70333481	6.764471553	OlfactoryBulb	5.20	-1.07	-7.19	-7.25
DRD5	Dopamine D5 receptor	6.70333481	6.764471553	Ovary	7.90	0.01	0.07	0.07
DRD5	Dopamine D5 receptor	6.70333481	6.764471553	Pancreas	7.25	-0.25	-1.68	-1.69
DRD5	Dopamine D5 receptor	6.70333481	6.764471553	PancreaticIslet	7.40	-0.19	-1.27	-1.28
DRD5	Dopamine D5 receptor	6.70333481	6.764471553	ParietalLobe	8.40	0.21	1.41	1.43

S2 Table. Target-tissue analysis for clozapine and chlorpromazine

Gene Symbol	Gene Name	Affinity (-log10(Ki))		Tissue	Expression		Combined Score (-log10(Ki)*Z)	
		clozapine	chlorpromazine		Raw-numbers	Z-score	clozapine	chlorpromazine
DRD5	Dopamine D5 receptor	6.70333481	6.764471553	Pineal_day	7.72	-0.06	-0.41	-0.42
DRD5	Dopamine D5 receptor	6.70333481	6.764471553	Pineal_night	7.68	-0.08	-0.52	-0.53
DRD5	Dopamine D5 receptor	6.70333481	6.764471553	Pituitary	9.50	0.65	4.37	4.41
DRD5	Dopamine D5 receptor	6.70333481	6.764471553	Placenta	6.80	-0.43	-2.89	-2.91
DRD5	Dopamine D5 receptor	6.70333481	6.764471553	Pons	8.95	0.43	2.89	2.92
DRD5	Dopamine D5 receptor	6.70333481	6.764471553	PrefrontalCortex	8.75	0.35	2.36	2.38
DRD5	Dopamine D5 receptor	6.70333481	6.764471553	Prostate	7.30	-0.23	-1.54	-1.56
DRD5	Dopamine D5 receptor	6.70333481	6.764471553	Retina	9.28	0.56	3.77	3.80
DRD5	Dopamine D5 receptor	6.70333481	6.764471553	Salivarygland	5.50	-0.95	-6.38	-6.44
DRD5	Dopamine D5 receptor	6.70333481	6.764471553	SkeletalMuscle	9.10	0.49	3.30	3.33
DRD5	Dopamine D5 receptor	6.70333481	6.764471553	Skin	7.25	-0.25	-1.68	-1.69
DRD5	Dopamine D5 receptor	6.70333481	6.764471553	Small_intestine	10.20	0.93	6.25	6.31
DRD5	Dopamine D5 receptor	6.70333481	6.764471553	SmoothMuscle	6.85	-0.41	-2.75	-2.78
DRD5	Dopamine D5 receptor	6.70333481	6.764471553	Spinalcord	7.35	-0.21	-1.41	-1.42
DRD5	Dopamine D5 receptor	6.70333481	6.764471553	SubthalamicNucleus	6.05	-0.73	-4.90	-4.95
DRD5	Dopamine D5 receptor	6.70333481	6.764471553	SuperiorCervicalGanglion	14.05	2.48	16.60	16.75
DRD5	Dopamine D5 receptor	6.70333481	6.764471553	TemporalLobe	7.95	0.03	0.21	0.21
DRD5	Dopamine D5 receptor	6.70333481	6.764471553	Testis	9.40	0.61	4.10	4.14
DRD5	Dopamine D5 receptor	6.70333481	6.764471553	TestisGermCell	7.30	-0.23	-1.54	-1.56
DRD5	Dopamine D5 receptor	6.70333481	6.764471553	TestisIntersitial	5.60	-0.91	-6.11	-6.17
DRD5	Dopamine D5 receptor	6.70333481	6.764471553	TestisLeydigCell	7.60	-0.11	-0.74	-0.74
DRD5	Dopamine D5 receptor	6.70333481	6.764471553	TestisSeminiferousTubule	5.90	-0.79	-5.31	-5.35
DRD5	Dopamine D5 receptor	6.70333481	6.764471553	Thalamus	7.05	-0.33	-2.21	-2.23
DRD5	Dopamine D5 receptor	6.70333481	6.764471553	Thymus	4.90	-1.19	-7.99	-8.07
DRD5	Dopamine D5 receptor	6.70333481	6.764471553	Thyroid	8.75	0.35	2.36	2.38
DRD5	Dopamine D5 receptor	6.70333481	6.764471553	Tongue	7.20	-0.27	-1.81	-1.83
DRD5	Dopamine D5 receptor	6.70333481	6.764471553	Tonsil	6.55	-0.53	-3.56	-3.59
DRD5	Dopamine D5 receptor	6.70333481	6.764471553	Trachea	6.10	-0.71	-4.77	-4.81
DRD5	Dopamine D5 receptor	6.70333481	6.764471553	TrigeminalGanglion	11.10	1.29	8.67	8.75
DRD5	Dopamine D5 receptor	6.70333481	6.764471553	Uterus	5.70	-0.87	-5.84	-5.90
DRD5	Dopamine D5 receptor	6.70333481	6.764471553	UterusCorpus	11.70	1.53	10.28	10.38
DRD5	Dopamine D5 receptor	6.70333481	6.764471553	WholeBlood	7.50	-0.15	-1.00	-1.01
DRD5	Dopamine D5 receptor	6.70333481	6.764471553	Wholebrain	6.00	-0.75	-5.04	-5.08
HRH1	Histamine H1 receptor	9.243363892	8.707743929	Adipocyte	4.73	-0.33	-3.07	-2.89
HRH1	Histamine H1 receptor	9.243363892	8.707743929	AdrenalCortex	5.23	0.13	1.23	1.16
HRH1	Histamine H1 receptor	9.243363892	8.707743929	Adrenalgland	4.13	-0.89	-8.22	-7.74
HRH1	Histamine H1 receptor	9.243363892	8.707743929	Amygdala	5.45	0.34	3.16	2.98
HRH1	Histamine H1 receptor	9.243363892	8.707743929	Appendix	5.93	0.78	7.24	6.82
HRH1	Histamine H1 receptor	9.243363892	8.707743929	AtrioventricularNode	5.63	0.50	4.66	4.39
HRH1	Histamine H1 receptor	9.243363892	8.707743929	BDCA4+_DentriticCells	5.18	0.09	0.80	0.75
HRH1	Histamine H1 receptor	9.243363892	8.707743929	Bonemarrow	4.85	-0.22	-1.99	-1.88
HRH1	Histamine H1 receptor	9.243363892	8.707743929	BronchialEpithelialCells	4.68	-0.38	-3.50	-3.29
HRH1	Histamine H1 receptor	9.243363892	8.707743929	CardiacMyocytes	7.53	2.27	20.98	19.76
HRH1	Histamine H1 receptor	9.243363892	8.707743929	Caudatenucleus	4.28	-0.75	-6.93	-6.53
HRH1	Histamine H1 receptor	9.243363892	8.707743929	CD105+_Endothelial	4.73	-0.33	-3.07	-2.89
HRH1	Histamine H1 receptor	9.243363892	8.707743929	CD14+_Monocytes	5.10	0.02	0.15	0.14
HRH1	Histamine H1 receptor	9.243363892	8.707743929	CD19+_BCells(neg._sel.)	5.05	-0.03	-0.28	-0.26
HRH1	Histamine H1 receptor	9.243363892	8.707743929	CD33+_Myeloid	6.08	0.92	8.53	8.03
HRH1	Histamine H1 receptor	9.243363892	8.707743929	CD34+	6.08	0.92	8.53	8.03
HRH1	Histamine H1 receptor	9.243363892	8.707743929	CD4+_Tcells	5.18	0.09	0.80	0.75
HRH1	Histamine H1 receptor	9.243363892	8.707743929	CD56+_NKCells	5.55	0.43	4.02	3.79
HRH1	Histamine H1 receptor	9.243363892	8.707743929	CD71+_EarlyErythroid	4.53	-0.52	-4.78	-4.51
HRH1	Histamine H1 receptor	9.243363892	8.707743929	CD8+_Tcells	4.53	-0.52	-4.78	-4.51

S2 Table. Target-tissue analysis for clozapine and chlorpromazine

Gene Symbol	Gene Name	Affinity (-log10(Ki))		Tissue	Expression		Combined Score (-log10(Ki)*Z)	
		clozapine	chlorpromazine		Raw-numbers	Z-score	clozapine	chlorpromazine
HRH1	Histamine H1 receptor	9.243363892	8.707743929	Cerebellum	3.93	-1.08	-9.94	-9.36
HRH1	Histamine H1 receptor	9.243363892	8.707743929	CerebellumPeduncles	5.48	0.37	3.37	3.18
HRH1	Histamine H1 receptor	9.243363892	8.707743929	CiliaryGanglion	7.40	2.15	19.91	18.75
HRH1	Histamine H1 receptor	9.243363892	8.707743929	CingulateCortex	5.23	0.13	1.23	1.16
HRH1	Histamine H1 receptor	9.243363892	8.707743929	Colon	4.80	-0.26	-2.42	-2.28
HRH1	Histamine H1 receptor	9.243363892	8.707743929	DorsalRootGanglion	4.65	-0.40	-3.71	-3.50
HRH1	Histamine H1 receptor	9.243363892	8.707743929	Fetalbrain	4.70	-0.36	-3.28	-3.09
HRH1	Histamine H1 receptor	9.243363892	8.707743929	Fetalliver	4.23	-0.80	-7.36	-6.93
HRH1	Histamine H1 receptor	9.243363892	8.707743929	Fetallung	3.98	-1.03	-9.51	-8.96
HRH1	Histamine H1 receptor	9.243363892	8.707743929	FetalThyroid	4.68	-0.38	-3.50	-3.29
HRH1	Histamine H1 receptor	9.243363892	8.707743929	GlobusPallidus	3.98	-1.03	-9.51	-8.96
HRH1	Histamine H1 receptor	9.243363892	8.707743929	Heart	6.20	1.04	9.60	9.04
HRH1	Histamine H1 receptor	9.243363892	8.707743929	Hypothalamus	5.15	0.06	0.58	0.55
HRH1	Histamine H1 receptor	9.243363892	8.707743929	Kidney	4.05	-0.96	-8.86	-8.35
HRH1	Histamine H1 receptor	9.243363892	8.707743929	Liver	6.38	1.20	11.10	10.46
HRH1	Histamine H1 receptor	9.243363892	8.707743929	Lung	5.28	0.18	1.66	1.56
HRH1	Histamine H1 receptor	9.243363892	8.707743929	Lymphnode	4.08	-0.94	-8.65	-8.15
HRH1	Histamine H1 receptor	9.243363892	8.707743929	MedullaOblongata	5.43	0.32	2.94	2.77
HRH1	Histamine H1 receptor	9.243363892	8.707743929	OccipitalLobe	4.33	-0.70	-6.50	-6.13
HRH1	Histamine H1 receptor	9.243363892	8.707743929	OlfactoryBulb	3.80	-1.19	-11.01	-10.37
HRH1	Histamine H1 receptor	9.243363892	8.707743929	Ovary	3.40	-1.56	-14.45	-13.61
HRH1	Histamine H1 receptor	9.243363892	8.707743929	Pancreas	3.85	-1.14	-10.58	-9.97
HRH1	Histamine H1 receptor	9.243363892	8.707743929	PancreaticIslet	5.15	0.06	0.58	0.55
HRH1	Histamine H1 receptor	9.243363892	8.707743929	ParietalLobe	5.20	0.11	1.01	0.95
HRH1	Histamine H1 receptor	9.243363892	8.707743929	Pineal_day	5.92	0.78	7.20	6.78
HRH1	Histamine H1 receptor	9.243363892	8.707743929	Pineal_night	5.83	0.69	6.42	6.05
HRH1	Histamine H1 receptor	9.243363892	8.707743929	Pituitary	5.60	0.48	4.45	4.19
HRH1	Histamine H1 receptor	9.243363892	8.707743929	Placenta	5.03	-0.05	-0.49	-0.46
HRH1	Histamine H1 receptor	9.243363892	8.707743929	Pons	4.68	-0.38	-3.50	-3.29
HRH1	Histamine H1 receptor	9.243363892	8.707743929	PrefrontalCortex	6.50	1.32	12.18	11.47
HRH1	Histamine H1 receptor	9.243363892	8.707743929	Prostate	5.55	0.43	4.02	3.79
HRH1	Histamine H1 receptor	9.243363892	8.707743929	Retina	5.83	0.69	6.38	6.01
HRH1	Histamine H1 receptor	9.243363892	8.707743929	Salivarygland	3.93	-1.08	-9.94	-9.36
HRH1	Histamine H1 receptor	9.243363892	8.707743929	SkeletalMuscle	6.78	1.57	14.54	13.70
HRH1	Histamine H1 receptor	9.243363892	8.707743929	Skin	4.80	-0.26	-2.42	-2.28
HRH1	Histamine H1 receptor	9.243363892	8.707743929	Small_intestine	4.63	-0.42	-3.93	-3.70
HRH1	Histamine H1 receptor	9.243363892	8.707743929	SmoothMuscle	7.78	2.50	23.13	21.79
HRH1	Histamine H1 receptor	9.243363892	8.707743929	Spinalcord	5.13	0.04	0.37	0.35
HRH1	Histamine H1 receptor	9.243363892	8.707743929	SubthalamicNucleus	4.60	-0.45	-4.14	-3.90
HRH1	Histamine H1 receptor	9.243363892	8.707743929	SuperiorCervicalGanglion	10.20	4.76	43.95	41.41
HRH1	Histamine H1 receptor	9.243363892	8.707743929	TemporalLobe	4.45	-0.59	-5.43	-5.11
HRH1	Histamine H1 receptor	9.243363892	8.707743929	Testis	4.08	-0.94	-8.65	-8.15
HRH1	Histamine H1 receptor	9.243363892	8.707743929	TestisGermCell	3.90	-1.10	-10.15	-9.56
HRH1	Histamine H1 receptor	9.243363892	8.707743929	TestisIntersitial	4.13	-0.89	-8.22	-7.74
HRH1	Histamine H1 receptor	9.243363892	8.707743929	TestisLeydigCell	4.93	-0.15	-1.35	-1.27
HRH1	Histamine H1 receptor	9.243363892	8.707743929	TestisSeminiferousTubule	4.13	-0.89	-8.22	-7.74
HRH1	Histamine H1 receptor	9.243363892	8.707743929	Thalamus	4.90	-0.17	-1.56	-1.47
HRH1	Histamine H1 receptor	9.243363892	8.707743929	Thymus	3.78	-1.21	-11.23	-10.58
HRH1	Histamine H1 receptor	9.243363892	8.707743929	Thyroid	5.95	0.81	7.45	7.02
HRH1	Histamine H1 receptor	9.243363892	8.707743929	Tongue	5.45	0.34	3.16	2.98
HRH1	Histamine H1 receptor	9.243363892	8.707743929	Tonsil	4.65	-0.40	-3.71	-3.50
HRH1	Histamine H1 receptor	9.243363892	8.707743929	Trachea	4.03	-0.98	-9.08	-8.55
HRH1	Histamine H1 receptor	9.243363892	8.707743929	TrigeminalGanglion	5.35	0.25	2.30	2.17

S2 Table. Target-tissue analysis for clozapine and chlorpromazine

Gene Symbol	Gene Name	Affinity (-log10(Ki))		Tissue	Expression		Combined Score (-log10(Ki)*Z)	
		clozapine	chlorpromazine		Raw-numbers	Z-score	clozapine	chlorpromazine
HRH1	Histamine H1 receptor	9.243363892	8.707743929	Uterus	4.23	-0.80	-7.36	-6.93
HRH1	Histamine H1 receptor	9.243363892	8.707743929	UterusCorpus	5.75	0.62	5.74	5.40
HRH1	Histamine H1 receptor	9.243363892	8.707743929	WholeBlood	5.20	0.11	1.01	0.95
HRH1	Histamine H1 receptor	9.243363892	8.707743929	Wholebrain	4.03	-0.98	-9.08	-8.55
HRH2	Histamine H2 receptor	5.449771647	5.588043762	Adipocyte	4.70	-0.27	-1.49	-1.53
HRH2	Histamine H2 receptor	5.449771647	5.588043762	AdrenalCortex	5.00	-0.06	-0.33	-0.33
HRH2	Histamine H2 receptor	5.449771647	5.588043762	Adrenalgland	4.10	-0.70	-3.82	-3.91
HRH2	Histamine H2 receptor	5.449771647	5.588043762	Amygdala	5.25	0.12	0.64	0.66
HRH2	Histamine H2 receptor	5.449771647	5.588043762	Appendix	4.85	-0.17	-0.91	-0.93
HRH2	Histamine H2 receptor	5.449771647	5.588043762	AtrioventricularNode	4.65	-0.31	-1.68	-1.73
HRH2	Histamine H2 receptor	5.449771647	5.588043762	BDCA4+_DentriticCells	5.65	0.40	2.20	2.25
HRH2	Histamine H2 receptor	5.449771647	5.588043762	Bonemarrow	4.85	-0.17	-0.91	-0.93
HRH2	Histamine H2 receptor	5.449771647	5.588043762	BronchialEpithelialCells	4.90	-0.13	-0.71	-0.73
HRH2	Histamine H2 receptor	5.449771647	5.588043762	CardiacMyocytes	12.10	4.99	27.22	27.91
HRH2	Histamine H2 receptor	5.449771647	5.588043762	Caudatenucleus	4.40	-0.49	-2.65	-2.72
HRH2	Histamine H2 receptor	5.449771647	5.588043762	CD105+_Endothelial	5.85	0.55	2.97	3.05
HRH2	Histamine H2 receptor	5.449771647	5.588043762	CD14+_Monocytes	5.45	0.26	1.42	1.46
HRH2	Histamine H2 receptor	5.449771647	5.588043762	CD19+_BCells(neg._sel.)	5.30	0.15	0.84	0.86
HRH2	Histamine H2 receptor	5.449771647	5.588043762	CD33+_Myeloid	6.80	1.22	6.66	6.83
HRH2	Histamine H2 receptor	5.449771647	5.588043762	CD34+	6.10	0.72	3.94	4.04
HRH2	Histamine H2 receptor	5.449771647	5.588043762	CD4+_Tcells	6.25	0.83	4.52	4.64
HRH2	Histamine H2 receptor	5.449771647	5.588043762	CD56+_NKCells	6.00	0.65	3.55	3.64
HRH2	Histamine H2 receptor	5.449771647	5.588043762	CD71+_EarlyErythroid	4.55	-0.38	-2.07	-2.12
HRH2	Histamine H2 receptor	5.449771647	5.588043762	CD8+_Tcells	4.85	-0.17	-0.91	-0.93
HRH2	Histamine H2 receptor	5.449771647	5.588043762	Cerebellum	4.05	-0.74	-4.01	-4.11
HRH2	Histamine H2 receptor	5.449771647	5.588043762	CerebellumPeduncles	5.50	0.30	1.61	1.65
HRH2	Histamine H2 receptor	5.449771647	5.588043762	CiliaryGanglion	3.45	-1.16	-6.34	-6.50
HRH2	Histamine H2 receptor	5.449771647	5.588043762	CingulateCortex	4.95	-0.10	-0.52	-0.53
HRH2	Histamine H2 receptor	5.449771647	5.588043762	Colon	4.95	-0.10	-0.52	-0.53
HRH2	Histamine H2 receptor	5.449771647	5.588043762	DorsalRootGanglion	3.65	-1.02	-5.56	-5.70
HRH2	Histamine H2 receptor	5.449771647	5.588043762	Fetalbrain	4.75	-0.24	-1.30	-1.33
HRH2	Histamine H2 receptor	5.449771647	5.588043762	Fetalliver	4.55	-0.38	-2.07	-2.12
HRH2	Histamine H2 receptor	5.449771647	5.588043762	Fetallung	4.00	-0.77	-4.20	-4.31
HRH2	Histamine H2 receptor	5.449771647	5.588043762	FetalThyroid	4.65	-0.31	-1.68	-1.73
HRH2	Histamine H2 receptor	5.449771647	5.588043762	GlobusPallidus	3.50	-1.13	-6.14	-6.30
HRH2	Histamine H2 receptor	5.449771647	5.588043762	Heart	11.90	4.85	26.44	27.11
HRH2	Histamine H2 receptor	5.449771647	5.588043762	Hypothalamus	5.20	0.08	0.45	0.46
HRH2	Histamine H2 receptor	5.449771647	5.588043762	Kidney	3.95	-0.81	-4.40	-4.51
HRH2	Histamine H2 receptor	5.449771647	5.588043762	Liver	6.45	0.97	5.30	5.43
HRH2	Histamine H2 receptor	5.449771647	5.588043762	Lung	5.50	0.30	1.61	1.65
HRH2	Histamine H2 receptor	5.449771647	5.588043762	Lymphnode	4.30	-0.56	-3.04	-3.12
HRH2	Histamine H2 receptor	5.449771647	5.588043762	MedullaOblongata	4.55	-0.38	-2.07	-2.12
HRH2	Histamine H2 receptor	5.449771647	5.588043762	OccipitalLobe	4.45	-0.45	-2.46	-2.52
HRH2	Histamine H2 receptor	5.449771647	5.588043762	OlfactoryBulb	3.70	-0.99	-5.37	-5.50
HRH2	Histamine H2 receptor	5.449771647	5.588043762	Ovary	4.85	-0.17	-0.91	-0.93
HRH2	Histamine H2 receptor	5.449771647	5.588043762	Pancreas	3.90	-0.84	-4.59	-4.71
HRH2	Histamine H2 receptor	5.449771647	5.588043762	PancreaticIslet	5.20	0.08	0.45	0.46
HRH2	Histamine H2 receptor	5.449771647	5.588043762	ParietalLobe	5.05	-0.02	-0.13	-0.14
HRH2	Histamine H2 receptor	5.449771647	5.588043762	Pineal_day	7.26	1.55	8.44	8.66
HRH2	Histamine H2 receptor	5.449771647	5.588043762	Pineal_night	6.68	1.14	6.19	6.35
HRH2	Histamine H2 receptor	5.449771647	5.588043762	Pituitary	5.75	0.47	2.58	2.65
HRH2	Histamine H2 receptor	5.449771647	5.588043762	Placenta	4.95	-0.10	-0.52	-0.53
HRH2	Histamine H2 receptor	5.449771647	5.588043762	Pons	4.60	-0.34	-1.88	-1.93

S2 Table. Target-tissue analysis for clozapine and chlorpromazine

Gene Symbol	Gene Name	Affinity (-log10(Ki))		Tissue	Expression		Combined Score (-log10(Ki)*Z)	
		clozapine	chlorpromazine		Raw-numbers	Z-score	clozapine	chlorpromazine
HRH2	Histamine H2 receptor	5.449771647	5.588043762	PrefrontalCortex	5.95	0.62	3.36	3.44
HRH2	Histamine H2 receptor	5.449771647	5.588043762	Prostate	5.85	0.55	2.97	3.05
HRH2	Histamine H2 receptor	5.449771647	5.588043762	Retina	5.98	0.63	3.46	3.54
HRH2	Histamine H2 receptor	5.449771647	5.588043762	Salivarygland	3.90	-0.84	-4.59	-4.71
HRH2	Histamine H2 receptor	5.449771647	5.588043762	SkeletalMuscle	5.85	0.55	2.97	3.05
HRH2	Histamine H2 receptor	5.449771647	5.588043762	Skin	3.65	-1.02	-5.56	-5.70
HRH2	Histamine H2 receptor	5.449771647	5.588043762	Small_intestine	4.80	-0.20	-1.10	-1.13
HRH2	Histamine H2 receptor	5.449771647	5.588043762	SmoothMuscle	5.25	0.12	0.64	0.66
HRH2	Histamine H2 receptor	5.449771647	5.588043762	Spinalcord	5.15	0.05	0.26	0.26
HRH2	Histamine H2 receptor	5.449771647	5.588043762	SubthalamicNucleus	4.35	-0.52	-2.85	-2.92
HRH2	Histamine H2 receptor	5.449771647	5.588043762	SuperiorCervicalGanglion	5.60	0.37	2.00	2.05
HRH2	Histamine H2 receptor	5.449771647	5.588043762	TemporalLobe	4.35	-0.52	-2.85	-2.92
HRH2	Histamine H2 receptor	5.449771647	5.588043762	Testis	4.25	-0.59	-3.24	-3.32
HRH2	Histamine H2 receptor	5.449771647	5.588043762	TestisGermCell	3.80	-0.91	-4.98	-5.11
HRH2	Histamine H2 receptor	5.449771647	5.588043762	TestisIntersitial	3.95	-0.81	-4.40	-4.51
HRH2	Histamine H2 receptor	5.449771647	5.588043762	TestisLeydigCell	4.80	-0.20	-1.10	-1.13
HRH2	Histamine H2 receptor	5.449771647	5.588043762	TestisSeminiferousTubule	4.20	-0.63	-3.43	-3.52
HRH2	Histamine H2 receptor	5.449771647	5.588043762	Thalamus	5.00	-0.06	-0.33	-0.33
HRH2	Histamine H2 receptor	5.449771647	5.588043762	Thymus	3.75	-0.95	-5.17	-5.31
HRH2	Histamine H2 receptor	5.449771647	5.588043762	Thyroid	6.25	0.83	4.52	4.64
HRH2	Histamine H2 receptor	5.449771647	5.588043762	Tongue	4.70	-0.27	-1.49	-1.53
HRH2	Histamine H2 receptor	5.449771647	5.588043762	Tonsil	5.05	-0.02	-0.13	-0.14
HRH2	Histamine H2 receptor	5.449771647	5.588043762	Trachea	4.35	-0.52	-2.85	-2.92
HRH2	Histamine H2 receptor	5.449771647	5.588043762	TrigeminalGanglion	4.75	-0.24	-1.30	-1.33
HRH2	Histamine H2 receptor	5.449771647	5.588043762	Uterus	4.10	-0.70	-3.82	-3.91
HRH2	Histamine H2 receptor	5.449771647	5.588043762	UterusCorpus	4.30	-0.56	-3.04	-3.12
HRH2	Histamine H2 receptor	5.449771647	5.588043762	WholeBlood	5.45	0.26	1.42	1.46
HRH2	Histamine H2 receptor	5.449771647	5.588043762	Wholebrain	4.25	-0.59	-3.24	-3.32
HRH3	Histamine H3 receptor	6.199970641	n/a	Adipocyte	5.10	-0.38	-2.38	n/a
HRH3	Histamine H3 receptor	6.199970641	n/a	AdrenalCortex	6.05	0.38	2.35	n/a
HRH3	Histamine H3 receptor	6.199970641	n/a	Adrenalgland	4.48	-0.88	-5.49	n/a
HRH3	Histamine H3 receptor	6.199970641	n/a	Amygdala	7.15	1.26	7.82	n/a
HRH3	Histamine H3 receptor	6.199970641	n/a	Appendix	5.33	-0.20	-1.26	n/a
HRH3	Histamine H3 receptor	6.199970641	n/a	AtrioventricularNode	5.70	0.10	0.61	n/a
HRH3	Histamine H3 receptor	6.199970641	n/a	BDCA4+_DentriticCells	5.38	-0.16	-1.01	n/a
HRH3	Histamine H3 receptor	6.199970641	n/a	Bonemarrow	5.35	-0.18	-1.13	n/a
HRH3	Histamine H3 receptor	6.199970641	n/a	BronchialEpithelialCells	4.75	-0.66	-4.12	n/a
HRH3	Histamine H3 receptor	6.199970641	n/a	CardiacMyocytes	7.68	1.68	10.43	n/a
HRH3	Histamine H3 receptor	6.199970641	n/a	Caudatenucleus	8.90	2.66	16.52	n/a
HRH3	Histamine H3 receptor	6.199970641	n/a	CD105+_Endothelial	5.23	-0.28	-1.76	n/a
HRH3	Histamine H3 receptor	6.199970641	n/a	CD14+_Monocytes	5.98	0.32	1.97	n/a
HRH3	Histamine H3 receptor	6.199970641	n/a	CD19+_BCells(neg._sel.)	5.33	-0.20	-1.26	n/a
HRH3	Histamine H3 receptor	6.199970641	n/a	CD33+_Myeloid	6.45	0.70	4.34	n/a
HRH3	Histamine H3 receptor	6.199970641	n/a	CD34+	6.55	0.78	4.83	n/a
HRH3	Histamine H3 receptor	6.199970641	n/a	CD4+_Tcells	5.53	-0.04	-0.26	n/a
HRH3	Histamine H3 receptor	6.199970641	n/a	CD56+_NKCells	6.03	0.36	2.22	n/a
HRH3	Histamine H3 receptor	6.199970641	n/a	CD71+_EarlyErythroid	5.08	-0.40	-2.50	n/a
HRH3	Histamine H3 receptor	6.199970641	n/a	CD8+_Tcells	4.90	-0.54	-3.37	n/a
HRH3	Histamine H3 receptor	6.199970641	n/a	Cerebellum	5.45	-0.10	-0.64	n/a
HRH3	Histamine H3 receptor	6.199970641	n/a	CerebellumPeduncles	7.43	1.48	9.18	n/a
HRH3	Histamine H3 receptor	6.199970641	n/a	CiliaryGanglion	4.03	-1.25	-7.72	n/a
HRH3	Histamine H3 receptor	6.199970641	n/a	CingulateCortex	6.25	0.54	3.34	n/a
HRH3	Histamine H3 receptor	6.199970641	n/a	Colon	5.25	-0.26	-1.63	n/a

S2 Table. Target-tissue analysis for clozapine and chlorpromazine

Gene Symbol	Gene Name	Affinity (-log10(Ki))		Tissue	Expression		Combined Score (-log10(Ki)*Z)	
		clozapine	chlorpromazine		Raw-numbers	Z-score	clozapine	chlorpromazine
HRH3	Histamine H3 receptor	6.199970641	n/a	DorsalRootGanglion	4.08	-1.21	-7.48	n/a
HRH3	Histamine H3 receptor	6.199970641	n/a	Fetalbrain	4.93	-0.52	-3.25	n/a
HRH3	Histamine H3 receptor	6.199970641	n/a	Fetalliver	4.65	-0.74	-4.62	n/a
HRH3	Histamine H3 receptor	6.199970641	n/a	Fetallung	3.98	-1.29	-7.97	n/a
HRH3	Histamine H3 receptor	6.199970641	n/a	FetalThyroid	5.80	0.18	1.10	n/a
HRH3	Histamine H3 receptor	6.199970641	n/a	GlobusPallidus	5.13	-0.36	-2.25	n/a
HRH3	Histamine H3 receptor	6.199970641	n/a	Heart	6.93	1.08	6.70	n/a
HRH3	Histamine H3 receptor	6.199970641	n/a	Hypothalamus	5.78	0.16	0.98	n/a
HRH3	Histamine H3 receptor	6.199970641	n/a	Kidney	4.33	-1.01	-6.23	n/a
HRH3	Histamine H3 receptor	6.199970641	n/a	Liver	7.60	1.62	10.05	n/a
HRH3	Histamine H3 receptor	6.199970641	n/a	Lung	5.60	0.02	0.11	n/a
HRH3	Histamine H3 receptor	6.199970641	n/a	Lymphnode	4.33	-1.01	-6.23	n/a
HRH3	Histamine H3 receptor	6.199970641	n/a	MedullaOblongata	4.90	-0.54	-3.37	n/a
HRH3	Histamine H3 receptor	6.199970641	n/a	OccipitalLobe	4.70	-0.70	-4.37	n/a
HRH3	Histamine H3 receptor	6.199970641	n/a	OlfactoryBulb	3.98	-1.29	-7.97	n/a
HRH3	Histamine H3 receptor	6.199970641	n/a	Ovary	3.55	-1.63	-10.09	n/a
HRH3	Histamine H3 receptor	6.199970641	n/a	Pancreas	4.25	-1.07	-6.60	n/a
HRH3	Histamine H3 receptor	6.199970641	n/a	PancreaticIslet	5.85	0.22	1.35	n/a
HRH3	Histamine H3 receptor	6.199970641	n/a	ParietalLobe	5.90	0.26	1.60	n/a
HRH3	Histamine H3 receptor	6.199970641	n/a	Pineal_day	6.43	0.68	4.24	n/a
HRH3	Histamine H3 receptor	6.199970641	n/a	Pineal_night	6.40	0.66	4.09	n/a
HRH3	Histamine H3 receptor	6.199970641	n/a	Pituitary	5.95	0.30	1.85	n/a
HRH3	Histamine H3 receptor	6.199970641	n/a	Placenta	4.83	-0.60	-3.75	n/a
HRH3	Histamine H3 receptor	6.199970641	n/a	Pons	8.50	2.34	14.53	n/a
HRH3	Histamine H3 receptor	6.199970641	n/a	PrefrontalCortex	6.30	0.58	3.59	n/a
HRH3	Histamine H3 receptor	6.199970641	n/a	Prostate	5.98	0.32	1.97	n/a
HRH3	Histamine H3 receptor	6.199970641	n/a	Retina	6.26	0.55	3.40	n/a
HRH3	Histamine H3 receptor	6.199970641	n/a	Salivarygland	4.43	-0.92	-5.73	n/a
HRH3	Histamine H3 receptor	6.199970641	n/a	SkeletalMuscle	9.13	2.84	17.64	n/a
HRH3	Histamine H3 receptor	6.199970641	n/a	Skin	6.30	0.58	3.59	n/a
HRH3	Histamine H3 receptor	6.199970641	n/a	Small_intestine	4.95	-0.50	-3.12	n/a
HRH3	Histamine H3 receptor	6.199970641	n/a	SmoothMuscle	5.88	0.24	1.48	n/a
HRH3	Histamine H3 receptor	6.199970641	n/a	Spinalcord	5.45	-0.10	-0.64	n/a
HRH3	Histamine H3 receptor	6.199970641	n/a	SubthalamicNucleus	5.13	-0.36	-2.25	n/a
HRH3	Histamine H3 receptor	6.199970641	n/a	SuperiorCervicalGanglion	6.20	0.50	3.09	n/a
HRH3	Histamine H3 receptor	6.199970641	n/a	TemporalLobe	7.98	1.92	11.92	n/a
HRH3	Histamine H3 receptor	6.199970641	n/a	Testis	4.30	-1.03	-6.36	n/a
HRH3	Histamine H3 receptor	6.199970641	n/a	TestisGermCell	4.08	-1.21	-7.48	n/a
HRH3	Histamine H3 receptor	6.199970641	n/a	TestisIntersitial	4.28	-1.05	-6.48	n/a
HRH3	Histamine H3 receptor	6.199970641	n/a	TestisLeydigCell	5.18	-0.32	-2.00	n/a
HRH3	Histamine H3 receptor	6.199970641	n/a	TestisSeminiferousTubule	4.40	-0.94	-5.86	n/a
HRH3	Histamine H3 receptor	6.199970641	n/a	Thalamus	9.23	2.93	18.14	n/a
HRH3	Histamine H3 receptor	6.199970641	n/a	Thymus	3.95	-1.31	-8.10	n/a
HRH3	Histamine H3 receptor	6.199970641	n/a	Thyroid	6.30	0.58	3.59	n/a
HRH3	Histamine H3 receptor	6.199970641	n/a	Tongue	5.23	-0.28	-1.76	n/a
HRH3	Histamine H3 receptor	6.199970641	n/a	Tonsil	5.03	-0.44	-2.75	n/a
HRH3	Histamine H3 receptor	6.199970641	n/a	Trachea	4.18	-1.13	-6.98	n/a
HRH3	Histamine H3 receptor	6.199970641	n/a	TrigeminalGanglion	6.43	0.68	4.21	n/a
HRH3	Histamine H3 receptor	6.199970641	n/a	Uterus	4.18	-1.13	-6.98	n/a
HRH3	Histamine H3 receptor	6.199970641	n/a	UterusCorpus	4.70	-0.70	-4.37	n/a
HRH3	Histamine H3 receptor	6.199970641	n/a	WholeBlood	5.63	0.04	0.23	n/a
HRH3	Histamine H3 receptor	6.199970641	n/a	Wholebrain	4.88	-0.56	-3.50	n/a
HRH4	Histamine H4 receptor	6.699991797	n/a	Adipocyte	4.03	-0.10	-0.65	n/a

S2 Table. Target-tissue analysis for clozapine and chlorpromazine

Gene Symbol	Gene Name	Affinity (-log10(Ki))		Tissue	Expression		Combined Score (-log10(Ki)*Z)	
		clozapine	chlorpromazine		Raw-numbers	Z-score	clozapine	chlorpromazine
HRH4	Histamine H4 receptor	6.699991797	n/a	AdrenalCortex	4.35	0.40	2.67	n/a
HRH4	Histamine H4 receptor	6.699991797	n/a	Adrenalgland	3.40	-1.05	-7.04	n/a
HRH4	Histamine H4 receptor	6.699991797	n/a	Amygdala	4.28	0.28	1.90	n/a
HRH4	Histamine H4 receptor	6.699991797	n/a	Appendix	4.25	0.25	1.65	n/a
HRH4	Histamine H4 receptor	6.699991797	n/a	AtrioventricularNode	3.53	-0.86	-5.76	n/a
HRH4	Histamine H4 receptor	6.699991797	n/a	BDCA4+_DentriticCells	4.45	0.55	3.69	n/a
HRH4	Histamine H4 receptor	6.699991797	n/a	Bonemarrow	4.10	0.02	0.11	n/a
HRH4	Histamine H4 receptor	6.699991797	n/a	BronchialEpithelialCells	4.00	-0.14	-0.91	n/a
HRH4	Histamine H4 receptor	6.699991797	n/a	CardiacMyocytes	5.48	2.11	14.16	n/a
HRH4	Histamine H4 receptor	6.699991797	n/a	Caudatenucleus	3.63	-0.71	-4.74	n/a
HRH4	Histamine H4 receptor	6.699991797	n/a	CD105+_Endothelial	4.13	0.05	0.37	n/a
HRH4	Histamine H4 receptor	6.699991797	n/a	CD14+_Monocytes	4.35	0.40	2.67	n/a
HRH4	Histamine H4 receptor	6.699991797	n/a	CD19+_BCells(neg._sel.)	4.38	0.44	2.92	n/a
HRH4	Histamine H4 receptor	6.699991797	n/a	CD33+_Myeloid	5.28	1.81	12.12	n/a
HRH4	Histamine H4 receptor	6.699991797	n/a	CD34+	5.20	1.69	11.35	n/a
HRH4	Histamine H4 receptor	6.699991797	n/a	CD4+_Tcells	4.45	0.55	3.69	n/a
HRH4	Histamine H4 receptor	6.699991797	n/a	CD56+_NKCells	4.88	1.20	8.03	n/a
HRH4	Histamine H4 receptor	6.699991797	n/a	CD71+_EarlyErythroid	3.85	-0.36	-2.44	n/a
HRH4	Histamine H4 receptor	6.699991797	n/a	CD8+_Tcells	3.93	-0.25	-1.68	n/a
HRH4	Histamine H4 receptor	6.699991797	n/a	Cerebellum	3.28	-1.24	-8.32	n/a
HRH4	Histamine H4 receptor	6.699991797	n/a	CerebellumPeduncles	4.58	0.74	4.97	n/a
HRH4	Histamine H4 receptor	6.699991797	n/a	CiliaryGanglion	3.23	-1.32	-8.83	n/a
HRH4	Histamine H4 receptor	6.699991797	n/a	CingulateCortex	4.18	0.13	0.88	n/a
HRH4	Histamine H4 receptor	6.699991797	n/a	Colon	4.10	0.02	0.11	n/a
HRH4	Histamine H4 receptor	6.699991797	n/a	DorsalRootGanglion	3.25	-1.28	-8.57	n/a
HRH4	Histamine H4 receptor	6.699991797	n/a	Fetalbrain	4.08	-0.02	-0.14	n/a
HRH4	Histamine H4 receptor	6.699991797	n/a	Fetalliver	3.53	-0.86	-5.76	n/a
HRH4	Histamine H4 receptor	6.699991797	n/a	Fetallung	3.35	-1.13	-7.55	n/a
HRH4	Histamine H4 receptor	6.699991797	n/a	FetalThyroid	3.98	-0.17	-1.16	n/a
HRH4	Histamine H4 receptor	6.699991797	n/a	GlobusPallidus	3.03	-1.62	-10.87	n/a
HRH4	Histamine H4 receptor	6.699991797	n/a	Heart	5.48	2.11	14.16	n/a
HRH4	Histamine H4 receptor	6.699991797	n/a	Hypothalamus	4.45	0.55	3.69	n/a
HRH4	Histamine H4 receptor	6.699991797	n/a	Kidney	3.28	-1.24	-8.32	n/a
HRH4	Histamine H4 receptor	6.699991797	n/a	Liver	5.53	2.19	14.67	n/a
HRH4	Histamine H4 receptor	6.699991797	n/a	Lung	4.48	0.59	3.94	n/a
HRH4	Histamine H4 receptor	6.699991797	n/a	Lymphnode	3.48	-0.94	-6.27	n/a
HRH4	Histamine H4 receptor	6.699991797	n/a	MedullaOblongata	3.73	-0.56	-3.72	n/a
HRH4	Histamine H4 receptor	6.699991797	n/a	OccipitalLobe	3.65	-0.67	-4.49	n/a
HRH4	Histamine H4 receptor	6.699991797	n/a	OlfactoryBulb	3.20	-1.36	-9.08	n/a
HRH4	Histamine H4 receptor	6.699991797	n/a	Ovary	2.75	-2.04	-13.68	n/a
HRH4	Histamine H4 receptor	6.699991797	n/a	Pancreas	3.30	-1.20	-8.06	n/a
HRH4	Histamine H4 receptor	6.699991797	n/a	PancreaticIslet	4.38	0.44	2.92	n/a
HRH4	Histamine H4 receptor	6.699991797	n/a	ParietalLobe	4.35	0.40	2.67	n/a
HRH4	Histamine H4 receptor	6.699991797	n/a	Pineal_day	5.11	1.56	10.43	n/a
HRH4	Histamine H4 receptor	6.699991797	n/a	Pineal_night	4.98	1.36	9.10	n/a
HRH4	Histamine H4 receptor	6.699991797	n/a	Pituitary	4.75	1.01	6.75	n/a
HRH4	Histamine H4 receptor	6.699991797	n/a	Placenta	4.23	0.21	1.39	n/a
HRH4	Histamine H4 receptor	6.699991797	n/a	Pons	3.88	-0.33	-2.19	n/a
HRH4	Histamine H4 receptor	6.699991797	n/a	PrefrontalCortex	4.98	1.35	9.05	n/a
HRH4	Histamine H4 receptor	6.699991797	n/a	Prostate	4.80	1.08	7.27	n/a
HRH4	Histamine H4 receptor	6.699991797	n/a	Retina	4.91	1.26	8.41	n/a
HRH4	Histamine H4 receptor	6.699991797	n/a	Salivarygland	3.63	-0.71	-4.74	n/a
HRH4	Histamine H4 receptor	6.699991797	n/a	SkeletalMuscle	4.83	1.12	7.52	n/a

S2 Table. Target-tissue analysis for clozapine and chlorpromazine

Gene Symbol	Gene Name	Affinity (-log10(Ki))		Tissue	Expression		Combined Score (-log10(Ki)*Z)	
		clozapine	chlorpromazine		Raw-numbers	Z-score	clozapine	chlorpromazine
HRH4	Histamine H4 receptor	6.699991797	n/a	Skin	3.40	-1.05	-7.04	n/a
HRH4	Histamine H4 receptor	6.699991797	n/a	Small_intestine	3.90	-0.29	-1.93	n/a
HRH4	Histamine H4 receptor	6.699991797	n/a	SmoothMuscle	4.60	0.78	5.22	n/a
HRH4	Histamine H4 receptor	6.699991797	n/a	Spinalcord	4.35	0.40	2.67	n/a
HRH4	Histamine H4 receptor	6.699991797	n/a	SubthalamicNucleus	3.78	-0.48	-3.21	n/a
HRH4	Histamine H4 receptor	6.699991797	n/a	SuperiorCervicalGanglion	5.08	1.50	10.07	n/a
HRH4	Histamine H4 receptor	6.699991797	n/a	TemporalLobe	3.75	-0.52	-3.46	n/a
HRH4	Histamine H4 receptor	6.699991797	n/a	Testis	3.50	-0.90	-6.02	n/a
HRH4	Histamine H4 receptor	6.699991797	n/a	TestisGermCell	3.43	-1.01	-6.78	n/a
HRH4	Histamine H4 receptor	6.699991797	n/a	TestisIntersitial	3.43	-1.01	-6.78	n/a
HRH4	Histamine H4 receptor	6.699991797	n/a	TestisLeydigCell	4.03	-0.10	-0.65	n/a
HRH4	Histamine H4 receptor	6.699991797	n/a	TestisSeminiferousTubule	3.50	-0.90	-6.02	n/a
HRH4	Histamine H4 receptor	6.699991797	n/a	Thalamus	4.15	0.09	0.62	n/a
HRH4	Histamine H4 receptor	6.699991797	n/a	Thymus	3.23	-1.32	-8.83	n/a
HRH4	Histamine H4 receptor	6.699991797	n/a	Thyroid	5.15	1.62	10.84	n/a
HRH4	Histamine H4 receptor	6.699991797	n/a	Tongue	4.33	0.36	2.41	n/a
HRH4	Histamine H4 receptor	6.699991797	n/a	Tonsil	3.93	-0.25	-1.68	n/a
HRH4	Histamine H4 receptor	6.699991797	n/a	Trachea	3.40	-1.05	-7.04	n/a
HRH4	Histamine H4 receptor	6.699991797	n/a	TrigeminalGanglion	4.43	0.51	3.43	n/a
HRH4	Histamine H4 receptor	6.699991797	n/a	Uterus	3.33	-1.17	-7.81	n/a
HRH4	Histamine H4 receptor	6.699991797	n/a	UterusCorpus	3.75	-0.52	-3.46	n/a
HRH4	Histamine H4 receptor	6.699991797	n/a	WholeBlood	4.48	0.59	3.94	n/a
HRH4	Histamine H4 receptor	6.699991797	n/a	Wholebrain	3.43	-1.01	-6.78	n/a
HTR1A	Serotonin 1a (5-HT1a) receptor	6.995678626	6.171984936	Adipocyte	3.70	-0.09	-0.60	-0.53
HTR1A	Serotonin 1a (5-HT1a) receptor	6.995678626	6.171984936	AdrenalCortex	3.75	-0.04	-0.27	-0.24
HTR1A	Serotonin 1a (5-HT1a) receptor	6.995678626	6.171984936	Adrenalgland	3.10	-0.65	-4.52	-3.98
HTR1A	Serotonin 1a (5-HT1a) receptor	6.995678626	6.171984936	Amygdala	3.90	0.10	0.70	0.62
HTR1A	Serotonin 1a (5-HT1a) receptor	6.995678626	6.171984936	Appendix	3.70	-0.09	-0.60	-0.53
HTR1A	Serotonin 1a (5-HT1a) receptor	6.995678626	6.171984936	AtrioventricularNode	2.80	-0.93	-6.47	-5.71
HTR1A	Serotonin 1a (5-HT1a) receptor	6.995678626	6.171984936	BDCA4+_DentriticCells	4.15	0.33	2.34	2.06
HTR1A	Serotonin 1a (5-HT1a) receptor	6.995678626	6.171984936	Bonemarrow	3.70	-0.09	-0.60	-0.53
HTR1A	Serotonin 1a (5-HT1a) receptor	6.995678626	6.171984936	BronchialEpithelialCells	3.70	-0.09	-0.60	-0.53
HTR1A	Serotonin 1a (5-HT1a) receptor	6.995678626	6.171984936	CardiacMyocytes	4.75	0.89	6.25	5.51
HTR1A	Serotonin 1a (5-HT1a) receptor	6.995678626	6.171984936	Caudatenucleus	3.35	-0.41	-2.88	-2.54
HTR1A	Serotonin 1a (5-HT1a) receptor	6.995678626	6.171984936	CD105+_Endothelial	3.80	0.01	0.05	0.05
HTR1A	Serotonin 1a (5-HT1a) receptor	6.995678626	6.171984936	CD14+_Monocytes	4.00	0.19	1.36	1.20
HTR1A	Serotonin 1a (5-HT1a) receptor	6.995678626	6.171984936	CD19+_BCells(neg._sel.)	4.00	0.19	1.36	1.20
HTR1A	Serotonin 1a (5-HT1a) receptor	6.995678626	6.171984936	CD33+_Myeloid	4.85	0.99	6.90	6.09
HTR1A	Serotonin 1a (5-HT1a) receptor	6.995678626	6.171984936	CD34+	4.80	0.94	6.58	5.80
HTR1A	Serotonin 1a (5-HT1a) receptor	6.995678626	6.171984936	CD4+_Tcells	4.10	0.29	2.01	1.77
HTR1A	Serotonin 1a (5-HT1a) receptor	6.995678626	6.171984936	CD56+_NKCells	4.55	0.71	4.95	4.36
HTR1A	Serotonin 1a (5-HT1a) receptor	6.995678626	6.171984936	CD71+_EarlyErythroid	3.45	-0.32	-2.23	-1.97
HTR1A	Serotonin 1a (5-HT1a) receptor	6.995678626	6.171984936	CD8+_Tcells	3.60	-0.18	-1.25	-1.11
HTR1A	Serotonin 1a (5-HT1a) receptor	6.995678626	6.171984936	Cerebellum	3.00	-0.74	-5.17	-4.56
HTR1A	Serotonin 1a (5-HT1a) receptor	6.995678626	6.171984936	CerebellumPeduncles	4.20	0.38	2.66	2.35
HTR1A	Serotonin 1a (5-HT1a) receptor	6.995678626	6.171984936	CiliaryGanglion	2.55	-1.16	-8.10	-7.15
HTR1A	Serotonin 1a (5-HT1a) receptor	6.995678626	6.171984936	CingulateCortex	3.90	0.10	0.70	0.62
HTR1A	Serotonin 1a (5-HT1a) receptor	6.995678626	6.171984936	Colon	3.75	-0.04	-0.27	-0.24
HTR1A	Serotonin 1a (5-HT1a) receptor	6.995678626	6.171984936	DorsalRootGanglion	2.80	-0.93	-6.47	-5.71
HTR1A	Serotonin 1a (5-HT1a) receptor	6.995678626	6.171984936	Fetalbrain	3.75	-0.04	-0.27	-0.24
HTR1A	Serotonin 1a (5-HT1a) receptor	6.995678626	6.171984936	Fetalliver	3.30	-0.46	-3.21	-2.83
HTR1A	Serotonin 1a (5-HT1a) receptor	6.995678626	6.171984936	Fetallung	3.15	-0.60	-4.19	-3.70
HTR1A	Serotonin 1a (5-HT1a) receptor	6.995678626	6.171984936	FetalThyroid	3.60	-0.18	-1.25	-1.11

S2 Table. Target-tissue analysis for clozapine and chlorpromazine

Gene Symbol	Gene Name	Affinity (-log10(Ki))		Tissue	Expression		Combined Score (-log10(Ki)*Z)	
		clozapine	chlorpromazine		Raw-numbers	Z-score	clozapine	chlorpromazine
HTR1A	Serotonin 1a (5-HT1a) receptor	6.995678626	6.171984936	GlobusPallidus	2.70	-1.02	-7.12	-6.29
HTR1A	Serotonin 1a (5-HT1a) receptor	6.995678626	6.171984936	Heart	5.65	1.73	12.12	10.70
HTR1A	Serotonin 1a (5-HT1a) receptor	6.995678626	6.171984936	Hypothalamus	4.10	0.29	2.01	1.77
HTR1A	Serotonin 1a (5-HT1a) receptor	6.995678626	6.171984936	Kidney	3.05	-0.69	-4.84	-4.27
HTR1A	Serotonin 1a (5-HT1a) receptor	6.995678626	6.171984936	Liver	11.35	7.05	49.31	43.51
HTR1A	Serotonin 1a (5-HT1a) receptor	6.995678626	6.171984936	Lung	4.15	0.33	2.34	2.06
HTR1A	Serotonin 1a (5-HT1a) receptor	6.995678626	6.171984936	Lymphnode	3.25	-0.51	-3.54	-3.12
HTR1A	Serotonin 1a (5-HT1a) receptor	6.995678626	6.171984936	MedullaOblongata	3.40	-0.37	-2.56	-2.26
HTR1A	Serotonin 1a (5-HT1a) receptor	6.995678626	6.171984936	OccipitalLobe	3.35	-0.41	-2.88	-2.54
HTR1A	Serotonin 1a (5-HT1a) receptor	6.995678626	6.171984936	OlfactoryBulb	2.95	-0.79	-5.49	-4.85
HTR1A	Serotonin 1a (5-HT1a) receptor	6.995678626	6.171984936	Ovary	2.45	-1.25	-8.76	-7.73
HTR1A	Serotonin 1a (5-HT1a) receptor	6.995678626	6.171984936	Pancreas	2.95	-0.79	-5.49	-4.85
HTR1A	Serotonin 1a (5-HT1a) receptor	6.995678626	6.171984936	PancreaticIslet	4.00	0.19	1.36	1.20
HTR1A	Serotonin 1a (5-HT1a) receptor	6.995678626	6.171984936	ParietalLobe	3.90	0.10	0.70	0.62
HTR1A	Serotonin 1a (5-HT1a) receptor	6.995678626	6.171984936	Pineal_day	4.68	0.83	5.79	5.11
HTR1A	Serotonin 1a (5-HT1a) receptor	6.995678626	6.171984936	Pineal_night	4.58	0.73	5.14	4.54
HTR1A	Serotonin 1a (5-HT1a) receptor	6.995678626	6.171984936	Pituitary	4.35	0.52	3.64	3.21
HTR1A	Serotonin 1a (5-HT1a) receptor	6.995678626	6.171984936	Placenta	3.80	0.01	0.05	0.05
HTR1A	Serotonin 1a (5-HT1a) receptor	6.995678626	6.171984936	Pons	3.55	-0.23	-1.58	-1.39
HTR1A	Serotonin 1a (5-HT1a) receptor	6.995678626	6.171984936	PrefrontalCortex	4.75	0.89	6.25	5.51
HTR1A	Serotonin 1a (5-HT1a) receptor	6.995678626	6.171984936	Prostate	4.35	0.52	3.64	3.21
HTR1A	Serotonin 1a (5-HT1a) receptor	6.995678626	6.171984936	Retina	4.58	0.73	5.11	4.51
HTR1A	Serotonin 1a (5-HT1a) receptor	6.995678626	6.171984936	Salivarygland	3.00	-0.74	-5.17	-4.56
HTR1A	Serotonin 1a (5-HT1a) receptor	6.995678626	6.171984936	SkeletalMuscle	4.30	0.47	3.31	2.92
HTR1A	Serotonin 1a (5-HT1a) receptor	6.995678626	6.171984936	Skin	2.75	-0.97	-6.80	-6.00
HTR1A	Serotonin 1a (5-HT1a) receptor	6.995678626	6.171984936	Small_intestine	3.60	-0.18	-1.25	-1.11
HTR1A	Serotonin 1a (5-HT1a) receptor	6.995678626	6.171984936	SmoothMuscle	4.25	0.43	2.99	2.64
HTR1A	Serotonin 1a (5-HT1a) receptor	6.995678626	6.171984936	Spinalcord	3.95	0.15	1.03	0.91
HTR1A	Serotonin 1a (5-HT1a) receptor	6.995678626	6.171984936	SubthalamicNucleus	3.35	-0.41	-2.88	-2.54
HTR1A	Serotonin 1a (5-HT1a) receptor	6.995678626	6.171984936	SuperiorCervicalGanglion	4.10	0.29	2.01	1.77
HTR1A	Serotonin 1a (5-HT1a) receptor	6.995678626	6.171984936	TemporalLobe	3.55	-0.23	-1.58	-1.39
HTR1A	Serotonin 1a (5-HT1a) receptor	6.995678626	6.171984936	Testis	3.25	-0.51	-3.54	-3.12
HTR1A	Serotonin 1a (5-HT1a) receptor	6.995678626	6.171984936	TestisGermCell	3.10	-0.65	-4.52	-3.98
HTR1A	Serotonin 1a (5-HT1a) receptor	6.995678626	6.171984936	TestisIntersitial	3.10	-0.65	-4.52	-3.98
HTR1A	Serotonin 1a (5-HT1a) receptor	6.995678626	6.171984936	TestisLeydigCell	3.65	-0.13	-0.93	-0.82
HTR1A	Serotonin 1a (5-HT1a) receptor	6.995678626	6.171984936	TestisSeminiferousTubule	3.20	-0.55	-3.86	-3.41
HTR1A	Serotonin 1a (5-HT1a) receptor	6.995678626	6.171984936	Thalamus	3.80	0.01	0.05	0.05
HTR1A	Serotonin 1a (5-HT1a) receptor	6.995678626	6.171984936	Thymus	2.95	-0.79	-5.49	-4.85
HTR1A	Serotonin 1a (5-HT1a) receptor	6.995678626	6.171984936	Thyroid	4.70	0.85	5.92	5.23
HTR1A	Serotonin 1a (5-HT1a) receptor	6.995678626	6.171984936	Tongue	3.65	-0.13	-0.93	-0.82
HTR1A	Serotonin 1a (5-HT1a) receptor	6.995678626	6.171984936	Tonsil	3.65	-0.13	-0.93	-0.82
HTR1A	Serotonin 1a (5-HT1a) receptor	6.995678626	6.171984936	Trachea	3.10	-0.65	-4.52	-3.98
HTR1A	Serotonin 1a (5-HT1a) receptor	6.995678626	6.171984936	TrigeminalGanglion	3.65	-0.13	-0.93	-0.82
HTR1A	Serotonin 1a (5-HT1a) receptor	6.995678626	6.171984936	Uterus	3.05	-0.69	-4.84	-4.27
HTR1A	Serotonin 1a (5-HT1a) receptor	6.995678626	6.171984936	UterusCorpus	3.30	-0.46	-3.21	-2.83
HTR1A	Serotonin 1a (5-HT1a) receptor	6.995678626	6.171984936	WholeBlood	4.10	0.29	2.01	1.77
HTR1A	Serotonin 1a (5-HT1a) receptor	6.995678626	6.171984936	Wholebrain	3.25	-0.51	-3.54	-3.12
HTR1B	Serotonin 1b (5-HT1b) receptor	6.018181393	5.735890844	Adipocyte	7.80	-0.15	-0.91	-0.86
HTR1B	Serotonin 1b (5-HT1b) receptor	6.018181393	5.735890844	AdrenalCortex	8.25	0.09	0.55	0.53
HTR1B	Serotonin 1b (5-HT1b) receptor	6.018181393	5.735890844	Adrenalgland	6.75	-0.72	-4.31	-4.11
HTR1B	Serotonin 1b (5-HT1b) receptor	6.018181393	5.735890844	Amygdala	8.30	0.12	0.72	0.68
HTR1B	Serotonin 1b (5-HT1b) receptor	6.018181393	5.735890844	Appendix	7.90	-0.10	-0.58	-0.55
HTR1B	Serotonin 1b (5-HT1b) receptor	6.018181393	5.735890844	AtrioventricularNode	6.15	-1.04	-6.26	-5.97

S2 Table. Target-tissue analysis for clozapine and chlorpromazine

Gene Symbol	Gene Name	Affinity (-log10(Ki))		Tissue	Expression		Combined Score (-log10(Ki)*Z)	
		clozapine	chlorpromazine		Raw-numbers	Z-score	clozapine	chlorpromazine
HTR1B	Serotonin 1b (5-HT1b) receptor	6.018181393	5.735890844	BDCA4+_DentriticCells	8.15	0.04	0.23	0.22
HTR1B	Serotonin 1b (5-HT1b) receptor	6.018181393	5.735890844	Bonemarrow	9.65	0.85	5.10	4.86
HTR1B	Serotonin 1b (5-HT1b) receptor	6.018181393	5.735890844	BronchialEpithelialCells	7.40	-0.37	-2.20	-2.10
HTR1B	Serotonin 1b (5-HT1b) receptor	6.018181393	5.735890844	CardiacMyocytes	9.95	1.01	6.07	5.79
HTR1B	Serotonin 1b (5-HT1b) receptor	6.018181393	5.735890844	Caudatenucleus	7.10	-0.53	-3.18	-3.03
HTR1B	Serotonin 1b (5-HT1b) receptor	6.018181393	5.735890844	CD105+_Endothelial	7.80	-0.15	-0.91	-0.86
HTR1B	Serotonin 1b (5-HT1b) receptor	6.018181393	5.735890844	CD14+_Monocytes	8.30	0.12	0.72	0.68
HTR1B	Serotonin 1b (5-HT1b) receptor	6.018181393	5.735890844	CD19+_BCells(neg._sel.)	8.20	0.07	0.39	0.37
HTR1B	Serotonin 1b (5-HT1b) receptor	6.018181393	5.735890844	CD33+_Myeloid	9.45	0.74	4.45	4.24
HTR1B	Serotonin 1b (5-HT1b) receptor	6.018181393	5.735890844	CD34+	9.75	0.90	5.42	5.17
HTR1B	Serotonin 1b (5-HT1b) receptor	6.018181393	5.735890844	CD4+_Tcells	8.35	0.15	0.88	0.84
HTR1B	Serotonin 1b (5-HT1b) receptor	6.018181393	5.735890844	CD56+_NKCells	9.05	0.52	3.15	3.00
HTR1B	Serotonin 1b (5-HT1b) receptor	6.018181393	5.735890844	CD71+_EarlyErythroid	7.35	-0.39	-2.37	-2.25
HTR1B	Serotonin 1b (5-HT1b) receptor	6.018181393	5.735890844	CD8+_Tcells	7.05	-0.55	-3.34	-3.18
HTR1B	Serotonin 1b (5-HT1b) receptor	6.018181393	5.735890844	Cerebellum	6.40	-0.91	-5.45	-5.19
HTR1B	Serotonin 1b (5-HT1b) receptor	6.018181393	5.735890844	CerebellumPeduncles	9.05	0.52	3.15	3.00
HTR1B	Serotonin 1b (5-HT1b) receptor	6.018181393	5.735890844	CiliaryGanglion	5.65	-1.31	-7.88	-7.51
HTR1B	Serotonin 1b (5-HT1b) receptor	6.018181393	5.735890844	CingulateCortex	8.10	0.01	0.07	0.06
HTR1B	Serotonin 1b (5-HT1b) receptor	6.018181393	5.735890844	Colon	7.90	-0.10	-0.58	-0.55
HTR1B	Serotonin 1b (5-HT1b) receptor	6.018181393	5.735890844	DorsalRootGanglion	6.05	-1.09	-6.58	-6.28
HTR1B	Serotonin 1b (5-HT1b) receptor	6.018181393	5.735890844	Fetalbrain	7.80	-0.15	-0.91	-0.86
HTR1B	Serotonin 1b (5-HT1b) receptor	6.018181393	5.735890844	Fetalliver	8.90	0.44	2.66	2.54
HTR1B	Serotonin 1b (5-HT1b) receptor	6.018181393	5.735890844	Fetallung	6.45	-0.88	-5.29	-5.04
HTR1B	Serotonin 1b (5-HT1b) receptor	6.018181393	5.735890844	FetalThyroid	7.65	-0.23	-1.39	-1.33
HTR1B	Serotonin 1b (5-HT1b) receptor	6.018181393	5.735890844	GlobusPallidus	5.75	-1.26	-7.56	-7.20
HTR1B	Serotonin 1b (5-HT1b) receptor	6.018181393	5.735890844	Heart	17.35	5.00	30.08	28.67
HTR1B	Serotonin 1b (5-HT1b) receptor	6.018181393	5.735890844	Hypothalamus	8.55	0.25	1.53	1.46
HTR1B	Serotonin 1b (5-HT1b) receptor	6.018181393	5.735890844	Kidney	6.55	-0.82	-4.96	-4.73
HTR1B	Serotonin 1b (5-HT1b) receptor	6.018181393	5.735890844	Liver	14.90	3.68	22.13	21.09
HTR1B	Serotonin 1b (5-HT1b) receptor	6.018181393	5.735890844	Lung	8.80	0.39	2.34	2.23
HTR1B	Serotonin 1b (5-HT1b) receptor	6.018181393	5.735890844	Lymphnode	6.85	-0.66	-3.99	-3.80
HTR1B	Serotonin 1b (5-HT1b) receptor	6.018181393	5.735890844	MedullaOblongata	7.25	-0.45	-2.69	-2.56
HTR1B	Serotonin 1b (5-HT1b) receptor	6.018181393	5.735890844	OccipitalLobe	7.15	-0.50	-3.01	-2.87
HTR1B	Serotonin 1b (5-HT1b) receptor	6.018181393	5.735890844	OlfactoryBulb	6.05	-1.09	-6.58	-6.28
HTR1B	Serotonin 1b (5-HT1b) receptor	6.018181393	5.735890844	Ovary	5.30	-1.50	-9.02	-8.59
HTR1B	Serotonin 1b (5-HT1b) receptor	6.018181393	5.735890844	Pancreas	6.45	-0.88	-5.29	-5.04
HTR1B	Serotonin 1b (5-HT1b) receptor	6.018181393	5.735890844	PancreaticIslet	8.45	0.20	1.20	1.15
HTR1B	Serotonin 1b (5-HT1b) receptor	6.018181393	5.735890844	ParietalLobe	8.30	0.12	0.72	0.68
HTR1B	Serotonin 1b (5-HT1b) receptor	6.018181393	5.735890844	Pineal_day	9.50	0.77	4.61	4.39
HTR1B	Serotonin 1b (5-HT1b) receptor	6.018181393	5.735890844	Pineal_night	9.60	0.82	4.93	4.70
HTR1B	Serotonin 1b (5-HT1b) receptor	6.018181393	5.735890844	Pituitary	9.25	0.63	3.80	3.62
HTR1B	Serotonin 1b (5-HT1b) receptor	6.018181393	5.735890844	Placenta	8.25	0.09	0.55	0.53
HTR1B	Serotonin 1b (5-HT1b) receptor	6.018181393	5.735890844	Pons	7.65	-0.23	-1.39	-1.33
HTR1B	Serotonin 1b (5-HT1b) receptor	6.018181393	5.735890844	PrefrontalCortex	9.90	0.98	5.91	5.63
HTR1B	Serotonin 1b (5-HT1b) receptor	6.018181393	5.735890844	Prostate	11.20	1.68	10.13	9.65
HTR1B	Serotonin 1b (5-HT1b) receptor	6.018181393	5.735890844	Retina	9.55	0.79	4.77	4.55
HTR1B	Serotonin 1b (5-HT1b) receptor	6.018181393	5.735890844	Salivarygland	6.30	-0.96	-5.77	-5.50
HTR1B	Serotonin 1b (5-HT1b) receptor	6.018181393	5.735890844	SkeletalMuscle	11.60	1.90	11.42	10.89
HTR1B	Serotonin 1b (5-HT1b) receptor	6.018181393	5.735890844	Skin	6.10	-1.07	-6.42	-6.12
HTR1B	Serotonin 1b (5-HT1b) receptor	6.018181393	5.735890844	Small_intestine	7.65	-0.23	-1.39	-1.33
HTR1B	Serotonin 1b (5-HT1b) receptor	6.018181393	5.735890844	SmoothMuscle	9.05	0.52	3.15	3.00
HTR1B	Serotonin 1b (5-HT1b) receptor	6.018181393	5.735890844	Spinalcord	8.45	0.20	1.20	1.15
HTR1B	Serotonin 1b (5-HT1b) receptor	6.018181393	5.735890844	SubthalamicNucleus	7.25	-0.45	-2.69	-2.56

S2 Table. Target-tissue analysis for clozapine and chlorpromazine

Gene Symbol	Gene Name	Affinity (-log10(Ki))		Tissue	Expression		Combined Score (-log10(Ki)*Z)	
		clozapine	chlorpromazine		Raw-numbers	Z-score	clozapine	chlorpromazine
HTR1B	Serotonin 1b (5-HT1b) receptor	6.018181393	5.735890844	SuperiorCervicalGanglion	9.25	0.63	3.80	3.62
HTR1B	Serotonin 1b (5-HT1b) receptor	6.018181393	5.735890844	TemporalLobe	7.20	-0.47	-2.85	-2.72
HTR1B	Serotonin 1b (5-HT1b) receptor	6.018181393	5.735890844	Testis	7.25	-0.45	-2.69	-2.56
HTR1B	Serotonin 1b (5-HT1b) receptor	6.018181393	5.735890844	TestisGermCell	6.65	-0.77	-4.64	-4.42
HTR1B	Serotonin 1b (5-HT1b) receptor	6.018181393	5.735890844	TestisIntersitial	6.65	-0.77	-4.64	-4.42
HTR1B	Serotonin 1b (5-HT1b) receptor	6.018181393	5.735890844	TestisLeydigCell	7.75	-0.18	-1.07	-1.02
HTR1B	Serotonin 1b (5-HT1b) receptor	6.018181393	5.735890844	TestisSeminiferousTubule	6.80	-0.69	-4.15	-3.96
HTR1B	Serotonin 1b (5-HT1b) receptor	6.018181393	5.735890844	Thalamus	8.00	-0.04	-0.26	-0.24
HTR1B	Serotonin 1b (5-HT1b) receptor	6.018181393	5.735890844	Thymus	6.20	-1.01	-6.10	-5.81
HTR1B	Serotonin 1b (5-HT1b) receptor	6.018181393	5.735890844	Thyroid	9.85	0.95	5.75	5.48
HTR1B	Serotonin 1b (5-HT1b) receptor	6.018181393	5.735890844	Tongue	7.90	-0.10	-0.58	-0.55
HTR1B	Serotonin 1b (5-HT1b) receptor	6.018181393	5.735890844	Tonsil	7.70	-0.20	-1.23	-1.17
HTR1B	Serotonin 1b (5-HT1b) receptor	6.018181393	5.735890844	Trachea	6.65	-0.77	-4.64	-4.42
HTR1B	Serotonin 1b (5-HT1b) receptor	6.018181393	5.735890844	TrigeminalGanglion	8.25	0.09	0.55	0.53
HTR1B	Serotonin 1b (5-HT1b) receptor	6.018181393	5.735890844	Uterus	6.50	-0.85	-5.12	-4.88
HTR1B	Serotonin 1b (5-HT1b) receptor	6.018181393	5.735890844	UterusCorpus	8.45	0.20	1.20	1.15
HTR1B	Serotonin 1b (5-HT1b) receptor	6.018181393	5.735890844	WholeBlood	8.65	0.31	1.85	1.77
HTR1B	Serotonin 1b (5-HT1b) receptor	6.018181393	5.735890844	Wholebrain	6.75	-0.72	-4.31	-4.11
HTR2A	Serotonin 2a (5-HT2a) receptor	8.920818754	8.958607315	Adipocyte	4.28	-0.29	-2.59	-2.60
HTR2A	Serotonin 2a (5-HT2a) receptor	8.920818754	8.958607315	AdrenalCortex	4.63	-0.17	-1.50	-1.51
HTR2A	Serotonin 2a (5-HT2a) receptor	8.920818754	8.958607315	Adrenalgland	3.68	-0.50	-4.47	-4.49
HTR2A	Serotonin 2a (5-HT2a) receptor	8.920818754	8.958607315	Amygdala	4.58	-0.19	-1.66	-1.66
HTR2A	Serotonin 2a (5-HT2a) receptor	8.920818754	8.958607315	Appendix	4.55	-0.19	-1.73	-1.74
HTR2A	Serotonin 2a (5-HT2a) receptor	8.920818754	8.958607315	AtrioventricularNode	4.05	-0.37	-3.30	-3.31
HTR2A	Serotonin 2a (5-HT2a) receptor	8.920818754	8.958607315	BDCA4+_DentriticCells	4.68	-0.15	-1.34	-1.35
HTR2A	Serotonin 2a (5-HT2a) receptor	8.920818754	8.958607315	Bonemarrow	4.30	-0.28	-2.52	-2.53
HTR2A	Serotonin 2a (5-HT2a) receptor	8.920818754	8.958607315	BronchialEpithelialCells	4.25	-0.30	-2.67	-2.68
HTR2A	Serotonin 2a (5-HT2a) receptor	8.920818754	8.958607315	CardiacMyocytes	5.65	0.19	1.70	1.71
HTR2A	Serotonin 2a (5-HT2a) receptor	8.920818754	8.958607315	Caudatenucleus	3.88	-0.43	-3.84	-3.86
HTR2A	Serotonin 2a (5-HT2a) receptor	8.920818754	8.958607315	CD105+_Endothelial	4.40	-0.25	-2.20	-2.21
HTR2A	Serotonin 2a (5-HT2a) receptor	8.920818754	8.958607315	CD14+_Monocytes	4.60	-0.18	-1.58	-1.58
HTR2A	Serotonin 2a (5-HT2a) receptor	8.920818754	8.958607315	CD19+_BCells(neg._sel.)	4.60	-0.18	-1.58	-1.58
HTR2A	Serotonin 2a (5-HT2a) receptor	8.920818754	8.958607315	CD33+_Myeloid	5.58	0.16	1.47	1.48
HTR2A	Serotonin 2a (5-HT2a) receptor	8.920818754	8.958607315	CD34+	5.53	0.15	1.31	1.32
HTR2A	Serotonin 2a (5-HT2a) receptor	8.920818754	8.958607315	CD4+_Tcells	4.68	-0.15	-1.34	-1.35
HTR2A	Serotonin 2a (5-HT2a) receptor	8.920818754	8.958607315	CD56+_NKCells	5.10	0.00	-0.01	-0.01
HTR2A	Serotonin 2a (5-HT2a) receptor	8.920818754	8.958607315	CD71+_EarlyErythroid	4.05	-0.37	-3.30	-3.31
HTR2A	Serotonin 2a (5-HT2a) receptor	8.920818754	8.958607315	CD8+_Tcells	4.15	-0.33	-2.98	-3.00
HTR2A	Serotonin 2a (5-HT2a) receptor	8.920818754	8.958607315	Cerebellum	3.50	-0.56	-5.02	-5.04
HTR2A	Serotonin 2a (5-HT2a) receptor	8.920818754	8.958607315	CerebellumPeduncles	4.93	-0.06	-0.56	-0.56
HTR2A	Serotonin 2a (5-HT2a) receptor	8.920818754	8.958607315	CiliaryGanglion	3.20	-0.67	-5.95	-5.98
HTR2A	Serotonin 2a (5-HT2a) receptor	8.920818754	8.958607315	CingulateCortex	9.53	1.55	13.81	13.87
HTR2A	Serotonin 2a (5-HT2a) receptor	8.920818754	8.958607315	Colon	4.33	-0.27	-2.44	-2.45
HTR2A	Serotonin 2a (5-HT2a) receptor	8.920818754	8.958607315	DorsalRootGanglion	3.95	-0.40	-3.61	-3.62
HTR2A	Serotonin 2a (5-HT2a) receptor	8.920818754	8.958607315	Fetalbrain	4.38	-0.26	-2.28	-2.29
HTR2A	Serotonin 2a (5-HT2a) receptor	8.920818754	8.958607315	Fetalliver	3.73	-0.48	-4.31	-4.33
HTR2A	Serotonin 2a (5-HT2a) receptor	8.920818754	8.958607315	Fetallung	3.60	-0.53	-4.70	-4.72
HTR2A	Serotonin 2a (5-HT2a) receptor	8.920818754	8.958607315	FetalThyroid	4.23	-0.31	-2.75	-2.76
HTR2A	Serotonin 2a (5-HT2a) receptor	8.920818754	8.958607315	GlobusPallidus	5.98	0.30	2.72	2.73
HTR2A	Serotonin 2a (5-HT2a) receptor	8.920818754	8.958607315	Heart	5.60	0.17	1.55	1.55
HTR2A	Serotonin 2a (5-HT2a) receptor	8.920818754	8.958607315	Hypothalamus	4.80	-0.11	-0.95	-0.96
HTR2A	Serotonin 2a (5-HT2a) receptor	8.920818754	8.958607315	Kidney	3.53	-0.55	-4.94	-4.96
HTR2A	Serotonin 2a (5-HT2a) receptor	8.920818754	8.958607315	Liver	5.78	0.23	2.09	2.10

S2 Table. Target-tissue analysis for clozapine and chlorpromazine

Gene Symbol	Gene Name	Affinity (-log10(Ki))		Tissue	Expression		Combined Score (-log10(Ki)*Z)	
		clozapine	chlorpromazine		Raw-numbers	Z-score	clozapine	chlorpromazine
HTR2A	Serotonin 2a (5-HT2a) receptor	8.920818754	8.958607315	Lung	4.78	-0.12	-1.03	-1.04
HTR2A	Serotonin 2a (5-HT2a) receptor	8.920818754	8.958607315	Lymphnode	3.75	-0.47	-4.23	-4.25
HTR2A	Serotonin 2a (5-HT2a) receptor	8.920818754	8.958607315	MedullaOblongata	7.05	0.68	6.08	6.11
HTR2A	Serotonin 2a (5-HT2a) receptor	8.920818754	8.958607315	OccipitalLobe	13.58	2.97	26.47	26.58
HTR2A	Serotonin 2a (5-HT2a) receptor	8.920818754	8.958607315	OlfactoryBulb	3.43	-0.59	-5.25	-5.27
HTR2A	Serotonin 2a (5-HT2a) receptor	8.920818754	8.958607315	Ovary	3.10	-0.70	-6.27	-6.29
HTR2A	Serotonin 2a (5-HT2a) receptor	8.920818754	8.958607315	Pancreas	3.48	-0.57	-5.09	-5.12
HTR2A	Serotonin 2a (5-HT2a) receptor	8.920818754	8.958607315	PancreaticIslet	4.63	-0.17	-1.50	-1.51
HTR2A	Serotonin 2a (5-HT2a) receptor	8.920818754	8.958607315	ParietalLobe	9.10	1.40	12.49	12.54
HTR2A	Serotonin 2a (5-HT2a) receptor	8.920818754	8.958607315	Pineal_day	5.40	0.10	0.92	0.93
HTR2A	Serotonin 2a (5-HT2a) receptor	8.920818754	8.958607315	Pineal_night	5.28	0.06	0.55	0.55
HTR2A	Serotonin 2a (5-HT2a) receptor	8.920818754	8.958607315	Pituitary	5.10	0.00	-0.01	-0.01
HTR2A	Serotonin 2a (5-HT2a) receptor	8.920818754	8.958607315	Placenta	4.48	-0.22	-1.97	-1.98
HTR2A	Serotonin 2a (5-HT2a) receptor	8.920818754	8.958607315	Pons	5.83	0.25	2.25	2.26
HTR2A	Serotonin 2a (5-HT2a) receptor	8.920818754	8.958607315	PrefrontalCortex	24.53	6.80	60.69	60.95
HTR2A	Serotonin 2a (5-HT2a) receptor	8.920818754	8.958607315	Prostate	5.10	0.00	-0.01	-0.01
HTR2A	Serotonin 2a (5-HT2a) receptor	8.920818754	8.958607315	Retina	5.24	0.05	0.41	0.42
HTR2A	Serotonin 2a (5-HT2a) receptor	8.920818754	8.958607315	Salivarygland	3.45	-0.58	-5.17	-5.19
HTR2A	Serotonin 2a (5-HT2a) receptor	8.920818754	8.958607315	SkeletalMuscle	5.05	-0.02	-0.17	-0.17
HTR2A	Serotonin 2a (5-HT2a) receptor	8.920818754	8.958607315	Skin	3.65	-0.51	-4.55	-4.57
HTR2A	Serotonin 2a (5-HT2a) receptor	8.920818754	8.958607315	Small_intestine	4.18	-0.33	-2.91	-2.92
HTR2A	Serotonin 2a (5-HT2a) receptor	8.920818754	8.958607315	SmoothMuscle	4.88	-0.08	-0.72	-0.72
HTR2A	Serotonin 2a (5-HT2a) receptor	8.920818754	8.958607315	Spinalcord	4.63	-0.17	-1.50	-1.51
HTR2A	Serotonin 2a (5-HT2a) receptor	8.920818754	8.958607315	SubthalamicNucleus	12.23	2.49	22.25	22.35
HTR2A	Serotonin 2a (5-HT2a) receptor	8.920818754	8.958607315	SuperiorCervicalGanglion	5.53	0.15	1.31	1.32
HTR2A	Serotonin 2a (5-HT2a) receptor	8.920818754	8.958607315	TemporalLobe	8.38	1.15	10.22	10.26
HTR2A	Serotonin 2a (5-HT2a) receptor	8.920818754	8.958607315	Testis	3.73	-0.48	-4.31	-4.33
HTR2A	Serotonin 2a (5-HT2a) receptor	8.920818754	8.958607315	TestisGermCell	3.68	-0.50	-4.47	-4.49
HTR2A	Serotonin 2a (5-HT2a) receptor	8.920818754	8.958607315	TestisIntersitial	3.63	-0.52	-4.62	-4.64
HTR2A	Serotonin 2a (5-HT2a) receptor	8.920818754	8.958607315	TestisLeydigCell	4.40	-0.25	-2.20	-2.21
HTR2A	Serotonin 2a (5-HT2a) receptor	8.920818754	8.958607315	TestisSeminiferousTubule	3.73	-0.48	-4.31	-4.33
HTR2A	Serotonin 2a (5-HT2a) receptor	8.920818754	8.958607315	Thalamus	4.40	-0.25	-2.20	-2.21
HTR2A	Serotonin 2a (5-HT2a) receptor	8.920818754	8.958607315	Thymus	3.40	-0.60	-5.33	-5.35
HTR2A	Serotonin 2a (5-HT2a) receptor	8.920818754	8.958607315	Thyroid	5.38	0.09	0.84	0.85
HTR2A	Serotonin 2a (5-HT2a) receptor	8.920818754	8.958607315	Tongue	4.38	-0.26	-2.28	-2.29
HTR2A	Serotonin 2a (5-HT2a) receptor	8.920818754	8.958607315	Tonsil	4.23	-0.31	-2.75	-2.76
HTR2A	Serotonin 2a (5-HT2a) receptor	8.920818754	8.958607315	Trachea	3.63	-0.52	-4.62	-4.64
HTR2A	Serotonin 2a (5-HT2a) receptor	8.920818754	8.958607315	TrigeminalGanglion	4.53	-0.20	-1.81	-1.82
HTR2A	Serotonin 2a (5-HT2a) receptor	8.920818754	8.958607315	Uterus	3.55	-0.54	-4.86	-4.88
HTR2A	Serotonin 2a (5-HT2a) receptor	8.920818754	8.958607315	UterusCorpus	3.90	-0.42	-3.77	-3.78
HTR2A	Serotonin 2a (5-HT2a) receptor	8.920818754	8.958607315	WholeBlood	4.70	-0.14	-1.27	-1.27
HTR2A	Serotonin 2a (5-HT2a) receptor	8.920818754	8.958607315	Wholebrain	3.88	-0.43	-3.84	-3.86
HTR2B	Serotonin 2b (5-HT2b) receptor	8.143875556	7.283996656	Adipocyte	5.00	-0.21	-1.74	-1.56
HTR2B	Serotonin 2b (5-HT2b) receptor	8.143875556	7.283996656	AdrenalCortex	5.85	0.48	3.93	3.52
HTR2B	Serotonin 2b (5-HT2b) receptor	8.143875556	7.283996656	Adrenalgland	4.35	-0.75	-6.08	-5.44
HTR2B	Serotonin 2b (5-HT2b) receptor	8.143875556	7.283996656	Amygdala	5.20	-0.05	-0.41	-0.36
HTR2B	Serotonin 2b (5-HT2b) receptor	8.143875556	7.283996656	Appendix	8.10	2.33	18.94	16.94
HTR2B	Serotonin 2b (5-HT2b) receptor	8.143875556	7.283996656	AtrioventricularNode	4.20	-0.87	-7.08	-6.33
HTR2B	Serotonin 2b (5-HT2b) receptor	8.143875556	7.283996656	BDCA4+_DentriticCells	5.25	-0.01	-0.07	-0.07
HTR2B	Serotonin 2b (5-HT2b) receptor	8.143875556	7.283996656	Bonemarrow	5.20	-0.05	-0.41	-0.36
HTR2B	Serotonin 2b (5-HT2b) receptor	8.143875556	7.283996656	BronchialEpithelialCells	4.95	-0.25	-2.08	-1.86
HTR2B	Serotonin 2b (5-HT2b) receptor	8.143875556	7.283996656	CardiacMyocytes	7.05	1.47	11.94	10.68
HTR2B	Serotonin 2b (5-HT2b) receptor	8.143875556	7.283996656	Caudatenucleus	4.55	-0.58	-4.74	-4.24

S2 Table. Target-tissue analysis for clozapine and chlorpromazine

Gene Symbol	Gene Name	Affinity (-log10(Ki))		Tissue	Expression		Combined Score (-log10(Ki)*Z)	
		clozapine	chlorpromazine		Raw-numbers	Z-score	clozapine	chlorpromazine
HTR2B	Serotonin 2b (5-HT2b) receptor	8.143875556	7.283996656	CD105+_Endothelial	5.10	-0.13	-1.07	-0.96
HTR2B	Serotonin 2b (5-HT2b) receptor	8.143875556	7.283996656	CD14+_Monocytes	5.35	0.07	0.59	0.53
HTR2B	Serotonin 2b (5-HT2b) receptor	8.143875556	7.283996656	CD19+_BCells(neg._sel.)	5.30	0.03	0.26	0.23
HTR2B	Serotonin 2b (5-HT2b) receptor	8.143875556	7.283996656	CD33+_Myeloid	6.25	0.81	6.60	5.90
HTR2B	Serotonin 2b (5-HT2b) receptor	8.143875556	7.283996656	CD34+	6.20	0.77	6.27	5.60
HTR2B	Serotonin 2b (5-HT2b) receptor	8.143875556	7.283996656	CD4+_Tcells	5.35	0.07	0.59	0.53
HTR2B	Serotonin 2b (5-HT2b) receptor	8.143875556	7.283996656	CD56+_NKCells	5.75	0.40	3.26	2.92
HTR2B	Serotonin 2b (5-HT2b) receptor	8.143875556	7.283996656	CD71+_EarlyErythroid	4.80	-0.38	-3.08	-2.75
HTR2B	Serotonin 2b (5-HT2b) receptor	8.143875556	7.283996656	CD8+_Tcells	4.65	-0.50	-4.08	-3.65
HTR2B	Serotonin 2b (5-HT2b) receptor	8.143875556	7.283996656	Cerebellum	4.15	-0.91	-7.41	-6.63
HTR2B	Serotonin 2b (5-HT2b) receptor	8.143875556	7.283996656	CerebellumPeduncles	5.85	0.48	3.93	3.52
HTR2B	Serotonin 2b (5-HT2b) receptor	8.143875556	7.283996656	CiliaryGanglion	3.80	-1.20	-9.75	-8.72
HTR2B	Serotonin 2b (5-HT2b) receptor	8.143875556	7.283996656	CingulateCortex	5.25	-0.01	-0.07	-0.07
HTR2B	Serotonin 2b (5-HT2b) receptor	8.143875556	7.283996656	Colon	5.05	-0.17	-1.41	-1.26
HTR2B	Serotonin 2b (5-HT2b) receptor	8.143875556	7.283996656	DorsalRootGanglion	4.05	-0.99	-8.08	-7.23
HTR2B	Serotonin 2b (5-HT2b) receptor	8.143875556	7.283996656	Fetalbrain	4.95	-0.25	-2.08	-1.86
HTR2B	Serotonin 2b (5-HT2b) receptor	8.143875556	7.283996656	Fetalliver	4.50	-0.62	-5.08	-4.54
HTR2B	Serotonin 2b (5-HT2b) receptor	8.143875556	7.283996656	Fetallung	4.10	-0.95	-7.75	-6.93
HTR2B	Serotonin 2b (5-HT2b) receptor	8.143875556	7.283996656	FetalThyroid	4.90	-0.30	-2.41	-2.15
HTR2B	Serotonin 2b (5-HT2b) receptor	8.143875556	7.283996656	GlobusPallidus	3.80	-1.20	-9.75	-8.72
HTR2B	Serotonin 2b (5-HT2b) receptor	8.143875556	7.283996656	Heart	6.95	1.38	11.27	10.08
HTR2B	Serotonin 2b (5-HT2b) receptor	8.143875556	7.283996656	Hypothalamus	5.40	0.11	0.93	0.83
HTR2B	Serotonin 2b (5-HT2b) receptor	8.143875556	7.283996656	Kidney	4.25	-0.83	-6.75	-6.03
HTR2B	Serotonin 2b (5-HT2b) receptor	8.143875556	7.283996656	Liver	7.20	1.59	12.94	11.57
HTR2B	Serotonin 2b (5-HT2b) receptor	8.143875556	7.283996656	Lung	5.55	0.24	1.93	1.72
HTR2B	Serotonin 2b (5-HT2b) receptor	8.143875556	7.283996656	Lymphnode	4.30	-0.79	-6.41	-5.74
HTR2B	Serotonin 2b (5-HT2b) receptor	8.143875556	7.283996656	MedullaOblongata	4.60	-0.54	-4.41	-3.94
HTR2B	Serotonin 2b (5-HT2b) receptor	8.143875556	7.283996656	OccipitalLobe	4.50	-0.62	-5.08	-4.54
HTR2B	Serotonin 2b (5-HT2b) receptor	8.143875556	7.283996656	OlfactoryBulb	3.95	-1.07	-8.75	-7.82
HTR2B	Serotonin 2b (5-HT2b) receptor	8.143875556	7.283996656	Ovary	3.55	-1.40	-11.42	-10.21
HTR2B	Serotonin 2b (5-HT2b) receptor	8.143875556	7.283996656	Pancreas	4.10	-0.95	-7.75	-6.93
HTR2B	Serotonin 2b (5-HT2b) receptor	8.143875556	7.283996656	PancreaticIslet	5.40	0.11	0.93	0.83
HTR2B	Serotonin 2b (5-HT2b) receptor	8.143875556	7.283996656	ParietalLobe	5.50	0.20	1.59	1.43
HTR2B	Serotonin 2b (5-HT2b) receptor	8.143875556	7.283996656	Pineal_day	6.22	0.79	6.40	5.72
HTR2B	Serotonin 2b (5-HT2b) receptor	8.143875556	7.283996656	Pineal_night	6.10	0.69	5.60	5.01
HTR2B	Serotonin 2b (5-HT2b) receptor	8.143875556	7.283996656	Pituitary	5.90	0.52	4.26	3.81
HTR2B	Serotonin 2b (5-HT2b) receptor	8.143875556	7.283996656	Placenta	5.10	-0.13	-1.07	-0.96
HTR2B	Serotonin 2b (5-HT2b) receptor	8.143875556	7.283996656	Pons	4.85	-0.34	-2.74	-2.45
HTR2B	Serotonin 2b (5-HT2b) receptor	8.143875556	7.283996656	PrefrontalCortex	6.20	0.77	6.27	5.60
HTR2B	Serotonin 2b (5-HT2b) receptor	8.143875556	7.283996656	Prostate	5.85	0.48	3.93	3.52
HTR2B	Serotonin 2b (5-HT2b) receptor	8.143875556	7.283996656	Retina	6.08	0.67	5.43	4.86
HTR2B	Serotonin 2b (5-HT2b) receptor	8.143875556	7.283996656	Salivarygland	4.20	-0.87	-7.08	-6.33
HTR2B	Serotonin 2b (5-HT2b) receptor	8.143875556	7.283996656	SkeletalMuscle	6.30	0.85	6.93	6.20
HTR2B	Serotonin 2b (5-HT2b) receptor	8.143875556	7.283996656	Skin	4.60	-0.54	-4.41	-3.94
HTR2B	Serotonin 2b (5-HT2b) receptor	8.143875556	7.283996656	Small_intestine	4.90	-0.30	-2.41	-2.15
HTR2B	Serotonin 2b (5-HT2b) receptor	8.143875556	7.283996656	SmoothMuscle	5.70	0.36	2.93	2.62
HTR2B	Serotonin 2b (5-HT2b) receptor	8.143875556	7.283996656	Spinalcord	5.40	0.11	0.93	0.83
HTR2B	Serotonin 2b (5-HT2b) receptor	8.143875556	7.283996656	SubthalamicNucleus	4.70	-0.46	-3.74	-3.35
HTR2B	Serotonin 2b (5-HT2b) receptor	8.143875556	7.283996656	SuperiorCervicalGanglion	6.20	0.77	6.27	5.60
HTR2B	Serotonin 2b (5-HT2b) receptor	8.143875556	7.283996656	TemporalLobe	4.75	-0.42	-3.41	-3.05
HTR2B	Serotonin 2b (5-HT2b) receptor	8.143875556	7.283996656	Testis	4.30	-0.79	-6.41	-5.74
HTR2B	Serotonin 2b (5-HT2b) receptor	8.143875556	7.283996656	TestisGermCell	4.15	-0.91	-7.41	-6.63
HTR2B	Serotonin 2b (5-HT2b) receptor	8.143875556	7.283996656	TestisIntersitial	4.30	-0.79	-6.41	-5.74

S2 Table. Target-tissue analysis for clozapine and chlorpromazine

Gene Symbol	Gene Name	Affinity (-log10(Ki))		Tissue	Expression		Combined Score (-log10(Ki)*Z)	
		clozapine	chlorpromazine		Raw-numbers	Z-score	clozapine	chlorpromazine
HTR2B	Serotonin 2b (5-HT2b) receptor	8.143875556	7.283996656	TestisLeydigCell	5.15	-0.09	-0.74	-0.66
HTR2B	Serotonin 2b (5-HT2b) receptor	8.143875556	7.283996656	TestisSeminiferousTubule	4.35	-0.75	-6.08	-5.44
HTR2B	Serotonin 2b (5-HT2b) receptor	8.143875556	7.283996656	Thalamus	5.20	-0.05	-0.41	-0.36
HTR2B	Serotonin 2b (5-HT2b) receptor	8.143875556	7.283996656	Thymus	3.85	-1.16	-9.41	-8.42
HTR2B	Serotonin 2b (5-HT2b) receptor	8.143875556	7.283996656	Thyroid	6.20	0.77	6.27	5.60
HTR2B	Serotonin 2b (5-HT2b) receptor	8.143875556	7.283996656	Tongue	5.20	-0.05	-0.41	-0.36
HTR2B	Serotonin 2b (5-HT2b) receptor	8.143875556	7.283996656	Tonsil	4.90	-0.30	-2.41	-2.15
HTR2B	Serotonin 2b (5-HT2b) receptor	8.143875556	7.283996656	Trachea	4.25	-0.83	-6.75	-6.03
HTR2B	Serotonin 2b (5-HT2b) receptor	8.143875556	7.283996656	TrigeminalGanglion	5.70	0.36	2.93	2.62
HTR2B	Serotonin 2b (5-HT2b) receptor	8.143875556	7.283996656	Uterus	10.10	3.96	32.29	28.88
HTR2B	Serotonin 2b (5-HT2b) receptor	8.143875556	7.283996656	UterusCorpus	10.70	4.46	36.29	32.46
HTR2B	Serotonin 2b (5-HT2b) receptor	8.143875556	7.283996656	WholeBlood	5.45	0.15	1.26	1.13
HTR2B	Serotonin 2b (5-HT2b) receptor	8.143875556	7.283996656	Wholebrain	4.15	-0.91	-7.41	-6.63
HTR2C	Serotonin 2c (5-HT2c) receptor	8.500312917	8.562249437	Adipocyte	5.70	-0.22	-1.91	-1.92
HTR2C	Serotonin 2c (5-HT2c) receptor	8.500312917	8.562249437	AdrenalCortex	6.88	-0.15	-1.31	-1.32
HTR2C	Serotonin 2c (5-HT2c) receptor	8.500312917	8.562249437	Adrenalgland	5.15	-0.26	-2.19	-2.21
HTR2C	Serotonin 2c (5-HT2c) receptor	8.500312917	8.562249437	Amygdala	33.83	1.46	12.37	12.46
HTR2C	Serotonin 2c (5-HT2c) receptor	8.500312917	8.562249437	Appendix	7.53	-0.12	-0.98	-0.99
HTR2C	Serotonin 2c (5-HT2c) receptor	8.500312917	8.562249437	AtrioventricularNode	5.78	-0.22	-1.87	-1.89
HTR2C	Serotonin 2c (5-HT2c) receptor	8.500312917	8.562249437	BDCA4+_DentriticCells	6.10	-0.20	-1.71	-1.72
HTR2C	Serotonin 2c (5-HT2c) receptor	8.500312917	8.562249437	Bonemarrow	6.20	-0.19	-1.66	-1.67
HTR2C	Serotonin 2c (5-HT2c) receptor	8.500312917	8.562249437	BronchialEpithelialCells	5.68	-0.23	-1.92	-1.94
HTR2C	Serotonin 2c (5-HT2c) receptor	8.500312917	8.562249437	CardiacMyocytes	8.88	-0.04	-0.30	-0.30
HTR2C	Serotonin 2c (5-HT2c) receptor	8.500312917	8.562249437	Caudatenucleus	126.93	7.02	59.66	60.09
HTR2C	Serotonin 2c (5-HT2c) receptor	8.500312917	8.562249437	CD105+_Endothelial	5.83	-0.22	-1.85	-1.86
HTR2C	Serotonin 2c (5-HT2c) receptor	8.500312917	8.562249437	CD14+_Monocytes	6.20	-0.19	-1.66	-1.67
HTR2C	Serotonin 2c (5-HT2c) receptor	8.500312917	8.562249437	CD19+_BCells(neg._sel.)	6.10	-0.20	-1.71	-1.72
HTR2C	Serotonin 2c (5-HT2c) receptor	8.500312917	8.562249437	CD33+_Myeloid	7.25	-0.13	-1.12	-1.13
HTR2C	Serotonin 2c (5-HT2c) receptor	8.500312917	8.562249437	CD34+	7.13	-0.14	-1.19	-1.20
HTR2C	Serotonin 2c (5-HT2c) receptor	8.500312917	8.562249437	CD4+_Tcells	6.13	-0.20	-1.69	-1.71
HTR2C	Serotonin 2c (5-HT2c) receptor	8.500312917	8.562249437	CD56+_NKCells	6.80	-0.16	-1.35	-1.36
HTR2C	Serotonin 2c (5-HT2c) receptor	8.500312917	8.562249437	CD71+_EarlyErythroid	5.53	-0.24	-2.00	-2.01
HTR2C	Serotonin 2c (5-HT2c) receptor	8.500312917	8.562249437	CD8+_Tcells	5.35	-0.25	-2.09	-2.10
HTR2C	Serotonin 2c (5-HT2c) receptor	8.500312917	8.562249437	Cerebellum	4.80	-0.28	-2.37	-2.39
HTR2C	Serotonin 2c (5-HT2c) receptor	8.500312917	8.562249437	CerebellumPeduncles	6.95	-0.15	-1.28	-1.29
HTR2C	Serotonin 2c (5-HT2c) receptor	8.500312917	8.562249437	CiliaryGanglion	4.78	-0.28	-2.38	-2.40
HTR2C	Serotonin 2c (5-HT2c) receptor	8.500312917	8.562249437	CingulateCortex	6.13	-0.20	-1.69	-1.71
HTR2C	Serotonin 2c (5-HT2c) receptor	8.500312917	8.562249437	Colon	5.90	-0.21	-1.81	-1.82
HTR2C	Serotonin 2c (5-HT2c) receptor	8.500312917	8.562249437	DorsalRootGanglion	5.50	-0.24	-2.01	-2.03
HTR2C	Serotonin 2c (5-HT2c) receptor	8.500312917	8.562249437	Fetalbrain	5.63	-0.23	-1.95	-1.96
HTR2C	Serotonin 2c (5-HT2c) receptor	8.500312917	8.562249437	Fetalliver	5.20	-0.25	-2.16	-2.18
HTR2C	Serotonin 2c (5-HT2c) receptor	8.500312917	8.562249437	Fetallung	4.70	-0.28	-2.42	-2.44
HTR2C	Serotonin 2c (5-HT2c) receptor	8.500312917	8.562249437	FetalThyroid	5.75	-0.22	-1.89	-1.90
HTR2C	Serotonin 2c (5-HT2c) receptor	8.500312917	8.562249437	GlobusPallidus	4.58	-0.29	-2.48	-2.50
HTR2C	Serotonin 2c (5-HT2c) receptor	8.500312917	8.562249437	Heart	8.78	-0.04	-0.35	-0.35
HTR2C	Serotonin 2c (5-HT2c) receptor	8.500312917	8.562249437	Hypothalamus	61.90	3.13	26.63	26.83
HTR2C	Serotonin 2c (5-HT2c) receptor	8.500312917	8.562249437	Kidney	5.40	-0.24	-2.06	-2.08
HTR2C	Serotonin 2c (5-HT2c) receptor	8.500312917	8.562249437	Liver	8.48	-0.06	-0.50	-0.51
HTR2C	Serotonin 2c (5-HT2c) receptor	8.500312917	8.562249437	Lung	6.38	-0.18	-1.57	-1.58
HTR2C	Serotonin 2c (5-HT2c) receptor	8.500312917	8.562249437	Lymphnode	4.83	-0.28	-2.36	-2.37
HTR2C	Serotonin 2c (5-HT2c) receptor	8.500312917	8.562249437	MedullaOblongata	8.18	-0.08	-0.65	-0.66
HTR2C	Serotonin 2c (5-HT2c) receptor	8.500312917	8.562249437	OccipitalLobe	5.18	-0.26	-2.18	-2.19
HTR2C	Serotonin 2c (5-HT2c) receptor	8.500312917	8.562249437	OlfactoryBulb	4.60	-0.29	-2.47	-2.49

S2 Table. Target-tissue analysis for clozapine and chlorpromazine

Gene Symbol	Gene Name	Affinity (-log10(Ki))		Tissue	Expression		Combined Score (-log10(Ki)*Z)	
		clozapine	chlorpromazine		Raw-numbers	Z-score	clozapine	chlorpromazine
HTR2C	Serotonin 2c (5-HT2c) receptor	8.500312917	8.562249437	Ovary	4.33	-0.31	-2.61	-2.63
HTR2C	Serotonin 2c (5-HT2c) receptor	8.500312917	8.562249437	Pancreas	4.95	-0.27	-2.29	-2.31
HTR2C	Serotonin 2c (5-HT2c) receptor	8.500312917	8.562249437	PancreaticIslet	6.30	-0.19	-1.61	-1.62
HTR2C	Serotonin 2c (5-HT2c) receptor	8.500312917	8.562249437	ParietalLobe	6.45	-0.18	-1.53	-1.54
HTR2C	Serotonin 2c (5-HT2c) receptor	8.500312917	8.562249437	Pineal_day	7.23	-0.13	-1.13	-1.14
HTR2C	Serotonin 2c (5-HT2c) receptor	8.500312917	8.562249437	Pineal_night	66.05	3.38	28.74	28.95
HTR2C	Serotonin 2c (5-HT2c) receptor	8.500312917	8.562249437	Pituitary	6.80	-0.16	-1.35	-1.36
HTR2C	Serotonin 2c (5-HT2c) receptor	8.500312917	8.562249437	Placenta	5.83	-0.22	-1.85	-1.86
HTR2C	Serotonin 2c (5-HT2c) receptor	8.500312917	8.562249437	Pons	5.80	-0.22	-1.86	-1.87
HTR2C	Serotonin 2c (5-HT2c) receptor	8.500312917	8.562249437	PrefrontalCortex	7.08	-0.14	-1.21	-1.22
HTR2C	Serotonin 2c (5-HT2c) receptor	8.500312917	8.562249437	Prostate	6.78	-0.16	-1.36	-1.37
HTR2C	Serotonin 2c (5-HT2c) receptor	8.500312917	8.562249437	Retina	7.09	-0.14	-1.21	-1.21
HTR2C	Serotonin 2c (5-HT2c) receptor	8.500312917	8.562249437	Salivarygland	4.85	-0.28	-2.34	-2.36
HTR2C	Serotonin 2c (5-HT2c) receptor	8.500312917	8.562249437	SkeletalMuscle	8.03	-0.09	-0.73	-0.74
HTR2C	Serotonin 2c (5-HT2c) receptor	8.500312917	8.562249437	Skin	5.40	-0.24	-2.06	-2.08
HTR2C	Serotonin 2c (5-HT2c) receptor	8.500312917	8.562249437	Small_intestine	5.65	-0.23	-1.94	-1.95
HTR2C	Serotonin 2c (5-HT2c) receptor	8.500312917	8.562249437	SmoothMuscle	6.58	-0.17	-1.47	-1.48
HTR2C	Serotonin 2c (5-HT2c) receptor	8.500312917	8.562249437	Spinalcord	6.23	-0.19	-1.64	-1.66
HTR2C	Serotonin 2c (5-HT2c) receptor	8.500312917	8.562249437	SubthalamicNucleus	5.53	-0.24	-2.00	-2.01
HTR2C	Serotonin 2c (5-HT2c) receptor	8.500312917	8.562249437	SuperiorCervicalGanglion	8.50	-0.06	-0.49	-0.49
HTR2C	Serotonin 2c (5-HT2c) receptor	8.500312917	8.562249437	TemporalLobe	5.48	-0.24	-2.03	-2.04
HTR2C	Serotonin 2c (5-HT2c) receptor	8.500312917	8.562249437	Testis	5.00	-0.27	-2.27	-2.28
HTR2C	Serotonin 2c (5-HT2c) receptor	8.500312917	8.562249437	TestisGermCell	4.68	-0.29	-2.43	-2.45
HTR2C	Serotonin 2c (5-HT2c) receptor	8.500312917	8.562249437	TestisIntersitial	4.98	-0.27	-2.28	-2.30
HTR2C	Serotonin 2c (5-HT2c) receptor	8.500312917	8.562249437	TestisLeydigCell	6.00	-0.21	-1.76	-1.77
HTR2C	Serotonin 2c (5-HT2c) receptor	8.500312917	8.562249437	TestisSeminiferousTubule	5.00	-0.27	-2.27	-2.28
HTR2C	Serotonin 2c (5-HT2c) receptor	8.500312917	8.562249437	Thalamus	6.30	-0.19	-1.61	-1.62
HTR2C	Serotonin 2c (5-HT2c) receptor	8.500312917	8.562249437	Thymus	4.45	-0.30	-2.55	-2.56
HTR2C	Serotonin 2c (5-HT2c) receptor	8.500312917	8.562249437	Thyroid	7.13	-0.14	-1.19	-1.20
HTR2C	Serotonin 2c (5-HT2c) receptor	8.500312917	8.562249437	Tongue	6.35	-0.19	-1.58	-1.59
HTR2C	Serotonin 2c (5-HT2c) receptor	8.500312917	8.562249437	Tonsil	5.75	-0.22	-1.89	-1.90
HTR2C	Serotonin 2c (5-HT2c) receptor	8.500312917	8.562249437	Trachea	4.83	-0.28	-2.36	-2.37
HTR2C	Serotonin 2c (5-HT2c) receptor	8.500312917	8.562249437	TrigeminalGanglion	6.95	-0.15	-1.28	-1.29
HTR2C	Serotonin 2c (5-HT2c) receptor	8.500312917	8.562249437	Uterus	4.68	-0.29	-2.43	-2.45
HTR2C	Serotonin 2c (5-HT2c) receptor	8.500312917	8.562249437	UterusCorpus	5.83	-0.22	-1.85	-1.86
HTR2C	Serotonin 2c (5-HT2c) receptor	8.500312917	8.562249437	WholeBlood	6.30	-0.19	-1.61	-1.62
HTR2C	Serotonin 2c (5-HT2c) receptor	8.500312917	8.562249437	Wholebrain	4.98	-0.27	-2.28	-2.30
HTR3A	Serotonin 3a (5-HT3a) receptor	7.494850022	n/a	Adipocyte	4.28	-0.29	-2.19	n/a
HTR3A	Serotonin 3a (5-HT3a) receptor	7.494850022	n/a	AdrenalCortex	4.90	0.18	1.37	n/a
HTR3A	Serotonin 3a (5-HT3a) receptor	7.494850022	n/a	Adrenalgland	3.63	-0.78	-5.88	n/a
HTR3A	Serotonin 3a (5-HT3a) receptor	7.494850022	n/a	Amygdala	4.50	-0.12	-0.91	n/a
HTR3A	Serotonin 3a (5-HT3a) receptor	7.494850022	n/a	Appendix	7.23	1.94	14.57	n/a
HTR3A	Serotonin 3a (5-HT3a) receptor	7.494850022	n/a	AtrioventricularNode	3.95	-0.54	-4.03	n/a
HTR3A	Serotonin 3a (5-HT3a) receptor	7.494850022	n/a	BDCA4+_DentriticCells	6.28	1.22	9.18	n/a
HTR3A	Serotonin 3a (5-HT3a) receptor	7.494850022	n/a	Bonemarrow	4.55	-0.08	-0.62	n/a
HTR3A	Serotonin 3a (5-HT3a) receptor	7.494850022	n/a	BronchialEpithelialCells	4.23	-0.33	-2.47	n/a
HTR3A	Serotonin 3a (5-HT3a) receptor	7.494850022	n/a	CardiacMyocytes	5.75	0.83	6.19	n/a
HTR3A	Serotonin 3a (5-HT3a) receptor	7.494850022	n/a	Caudatenucleus	3.85	-0.61	-4.60	n/a
HTR3A	Serotonin 3a (5-HT3a) receptor	7.494850022	n/a	CD105+_Endothelial	4.33	-0.25	-1.90	n/a
HTR3A	Serotonin 3a (5-HT3a) receptor	7.494850022	n/a	CD14+_Monocytes	4.53	-0.10	-0.77	n/a
HTR3A	Serotonin 3a (5-HT3a) receptor	7.494850022	n/a	CD19+_BCells(neg._sel.)	4.63	-0.03	-0.20	n/a
HTR3A	Serotonin 3a (5-HT3a) receptor	7.494850022	n/a	CD33+_Myeloid	5.55	0.67	5.06	n/a
HTR3A	Serotonin 3a (5-HT3a) receptor	7.494850022	n/a	CD34+	5.38	0.54	4.06	n/a

S2 Table. Target-tissue analysis for clozapine and chlorpromazine

Gene Symbol	Gene Name	Affinity (-log10(Ki))		Tissue	Expression		Combined Score (-log10(Ki)*Z)	
		clozapine	chlorpromazine		Raw-numbers	Z-score	clozapine	chlorpromazine
HTR3A	Serotonin 3a (5-HT3a) receptor	7.494850022	n/a	CD4+_Tcells	4.65	-0.01	-0.05	n/a
HTR3A	Serotonin 3a (5-HT3a) receptor	7.494850022	n/a	CD56+_NKCells	5.08	0.31	2.36	n/a
HTR3A	Serotonin 3a (5-HT3a) receptor	7.494850022	n/a	CD71+_EarlyErythroid	4.05	-0.46	-3.46	n/a
HTR3A	Serotonin 3a (5-HT3a) receptor	7.494850022	n/a	CD8+_Tcells	4.08	-0.44	-3.32	n/a
HTR3A	Serotonin 3a (5-HT3a) receptor	7.494850022	n/a	Cerebellum	3.48	-0.90	-6.73	n/a
HTR3A	Serotonin 3a (5-HT3a) receptor	7.494850022	n/a	CerebellumPeduncles	4.93	0.20	1.51	n/a
HTR3A	Serotonin 3a (5-HT3a) receptor	7.494850022	n/a	CiliaryGanglion	7.33	2.02	15.14	n/a
HTR3A	Serotonin 3a (5-HT3a) receptor	7.494850022	n/a	CingulateCortex	4.58	-0.06	-0.48	n/a
HTR3A	Serotonin 3a (5-HT3a) receptor	7.494850022	n/a	Colon	4.30	-0.27	-2.04	n/a
HTR3A	Serotonin 3a (5-HT3a) receptor	7.494850022	n/a	DorsalRootGanglion	12.10	5.64	42.26	n/a
HTR3A	Serotonin 3a (5-HT3a) receptor	7.494850022	n/a	Fetalbrain	4.23	-0.33	-2.47	n/a
HTR3A	Serotonin 3a (5-HT3a) receptor	7.494850022	n/a	Fetalliver	3.75	-0.69	-5.17	n/a
HTR3A	Serotonin 3a (5-HT3a) receptor	7.494850022	n/a	Fetallung	3.58	-0.82	-6.16	n/a
HTR3A	Serotonin 3a (5-HT3a) receptor	7.494850022	n/a	FetalThyroid	4.30	-0.27	-2.04	n/a
HTR3A	Serotonin 3a (5-HT3a) receptor	7.494850022	n/a	GlobusPallidus	3.18	-1.13	-8.43	n/a
HTR3A	Serotonin 3a (5-HT3a) receptor	7.494850022	n/a	Heart	5.85	0.90	6.76	n/a
HTR3A	Serotonin 3a (5-HT3a) receptor	7.494850022	n/a	Hypothalamus	4.65	-0.01	-0.05	n/a
HTR3A	Serotonin 3a (5-HT3a) receptor	7.494850022	n/a	Kidney	3.55	-0.84	-6.30	n/a
HTR3A	Serotonin 3a (5-HT3a) receptor	7.494850022	n/a	Liver	5.78	0.85	6.34	n/a
HTR3A	Serotonin 3a (5-HT3a) receptor	7.494850022	n/a	Lung	4.75	0.07	0.51	n/a
HTR3A	Serotonin 3a (5-HT3a) receptor	7.494850022	n/a	Lymphnode	3.75	-0.69	-5.17	n/a
HTR3A	Serotonin 3a (5-HT3a) receptor	7.494850022	n/a	MedullaOblongata	4.18	-0.37	-2.75	n/a
HTR3A	Serotonin 3a (5-HT3a) receptor	7.494850022	n/a	OccipitalLobe	3.85	-0.61	-4.60	n/a
HTR3A	Serotonin 3a (5-HT3a) receptor	7.494850022	n/a	OlfactoryBulb	3.43	-0.94	-7.01	n/a
HTR3A	Serotonin 3a (5-HT3a) receptor	7.494850022	n/a	Ovary	3.28	-1.05	-7.87	n/a
HTR3A	Serotonin 3a (5-HT3a) receptor	7.494850022	n/a	Pancreas	3.45	-0.92	-6.87	n/a
HTR3A	Serotonin 3a (5-HT3a) receptor	7.494850022	n/a	PancreaticIslet	4.60	-0.05	-0.34	n/a
HTR3A	Serotonin 3a (5-HT3a) receptor	7.494850022	n/a	ParietalLobe	4.58	-0.06	-0.48	n/a
HTR3A	Serotonin 3a (5-HT3a) receptor	7.494850022	n/a	Pineal_day	5.40	0.56	4.21	n/a
HTR3A	Serotonin 3a (5-HT3a) receptor	7.494850022	n/a	Pineal_night	5.22	0.42	3.18	n/a
HTR3A	Serotonin 3a (5-HT3a) receptor	7.494850022	n/a	Pituitary	4.98	0.24	1.79	n/a
HTR3A	Serotonin 3a (5-HT3a) receptor	7.494850022	n/a	Placenta	4.35	-0.23	-1.76	n/a
HTR3A	Serotonin 3a (5-HT3a) receptor	7.494850022	n/a	Pons	4.15	-0.39	-2.90	n/a
HTR3A	Serotonin 3a (5-HT3a) receptor	7.494850022	n/a	PrefrontalCortex	5.33	0.50	3.78	n/a
HTR3A	Serotonin 3a (5-HT3a) receptor	7.494850022	n/a	Prostate	5.03	0.28	2.08	n/a
HTR3A	Serotonin 3a (5-HT3a) receptor	7.494850022	n/a	Retina	5.38	0.54	4.06	n/a
HTR3A	Serotonin 3a (5-HT3a) receptor	7.494850022	n/a	Salivarygland	5.00	0.26	1.93	n/a
HTR3A	Serotonin 3a (5-HT3a) receptor	7.494850022	n/a	SkeletalMuscle	5.40	0.56	4.21	n/a
HTR3A	Serotonin 3a (5-HT3a) receptor	7.494850022	n/a	Skin	3.65	-0.77	-5.74	n/a
HTR3A	Serotonin 3a (5-HT3a) receptor	7.494850022	n/a	Small_intestine	4.18	-0.37	-2.75	n/a
HTR3A	Serotonin 3a (5-HT3a) receptor	7.494850022	n/a	SmoothMuscle	5.35	0.52	3.92	n/a
HTR3A	Serotonin 3a (5-HT3a) receptor	7.494850022	n/a	Spinalcord	4.60	-0.05	-0.34	n/a
HTR3A	Serotonin 3a (5-HT3a) receptor	7.494850022	n/a	SubthalamicNucleus	3.98	-0.52	-3.89	n/a
HTR3A	Serotonin 3a (5-HT3a) receptor	7.494850022	n/a	SuperiorCervicalGanglion	9.25	3.48	26.07	n/a
HTR3A	Serotonin 3a (5-HT3a) receptor	7.494850022	n/a	TemporalLobe	3.98	-0.52	-3.89	n/a
HTR3A	Serotonin 3a (5-HT3a) receptor	7.494850022	n/a	Testis	3.83	-0.63	-4.74	n/a
HTR3A	Serotonin 3a (5-HT3a) receptor	7.494850022	n/a	TestisGermCell	3.55	-0.84	-6.30	n/a
HTR3A	Serotonin 3a (5-HT3a) receptor	7.494850022	n/a	TestisIntersitial	3.55	-0.84	-6.30	n/a
HTR3A	Serotonin 3a (5-HT3a) receptor	7.494850022	n/a	TestisLeydigCell	4.28	-0.29	-2.19	n/a
HTR3A	Serotonin 3a (5-HT3a) receptor	7.494850022	n/a	TestisSeminiferousTubule	3.70	-0.73	-5.45	n/a
HTR3A	Serotonin 3a (5-HT3a) receptor	7.494850022	n/a	Thalamus	4.43	-0.18	-1.33	n/a
HTR3A	Serotonin 3a (5-HT3a) receptor	7.494850022	n/a	Thymus	3.40	-0.95	-7.16	n/a
HTR3A	Serotonin 3a (5-HT3a) receptor	7.494850022	n/a	Thyroid	5.33	0.50	3.78	n/a

S2 Table. Target-tissue analysis for clozapine and chlorpromazine

Gene Symbol	Gene Name	Affinity (-log10(Ki))		Tissue	Expression		Combined Score (-log10(Ki)*Z)	
		clozapine	chlorpromazine		Raw-numbers	Z-score	clozapine	chlorpromazine
HTR3A	Serotonin 3a (5-HT3a) receptor	7.494850022	n/a	Tongue	4.40	-0.20	-1.48	n/a
HTR3A	Serotonin 3a (5-HT3a) receptor	7.494850022	n/a	Tonsil	5.45	0.60	4.49	n/a
HTR3A	Serotonin 3a (5-HT3a) receptor	7.494850022	n/a	Trachea	3.60	-0.80	-6.02	n/a
HTR3A	Serotonin 3a (5-HT3a) receptor	7.494850022	n/a	TrigeminalGanglion	5.20	0.41	3.07	n/a
HTR3A	Serotonin 3a (5-HT3a) receptor	7.494850022	n/a	Uterus	3.50	-0.88	-6.59	n/a
HTR3A	Serotonin 3a (5-HT3a) receptor	7.494850022	n/a	UterusCorpus	4.33	-0.25	-1.90	n/a
HTR3A	Serotonin 3a (5-HT3a) receptor	7.494850022	n/a	WholeBlood	4.70	0.03	0.23	n/a
HTR3A	Serotonin 3a (5-HT3a) receptor	7.494850022	n/a	Wholebrain	3.60	-0.80	-6.02	n/a
HTR5A	Serotonin 5a (5-HT5a) receptor	6	n/a	Adipocyte	5.90	-0.25	-1.52	n/a
HTR5A	Serotonin 5a (5-HT5a) receptor	6	n/a	AdrenalCortex	7.25	0.77	4.61	n/a
HTR5A	Serotonin 5a (5-HT5a) receptor	6	n/a	Adrenalgland	5.40	-0.63	-3.79	n/a
HTR5A	Serotonin 5a (5-HT5a) receptor	6	n/a	Amygdala	6.20	-0.03	-0.16	n/a
HTR5A	Serotonin 5a (5-HT5a) receptor	6	n/a	Appendix	7.35	0.84	5.06	n/a
HTR5A	Serotonin 5a (5-HT5a) receptor	6	n/a	AtrioventricularNode	4.55	-1.28	-7.66	n/a
HTR5A	Serotonin 5a (5-HT5a) receptor	6	n/a	BDCA4+_DentriticCells	6.00	-0.18	-1.07	n/a
HTR5A	Serotonin 5a (5-HT5a) receptor	6	n/a	Bonemarrow	6.35	0.09	0.52	n/a
HTR5A	Serotonin 5a (5-HT5a) receptor	6	n/a	BronchialEpithelialCells	5.85	-0.29	-1.75	n/a
HTR5A	Serotonin 5a (5-HT5a) receptor	6	n/a	CardiacMyocytes	8.05	1.37	8.24	n/a
HTR5A	Serotonin 5a (5-HT5a) receptor	6	n/a	Caudatenucleus	5.45	-0.59	-3.57	n/a
HTR5A	Serotonin 5a (5-HT5a) receptor	6	n/a	CD105+_Endothelial	5.95	-0.22	-1.30	n/a
HTR5A	Serotonin 5a (5-HT5a) receptor	6	n/a	CD14+_Monocytes	6.40	0.12	0.75	n/a
HTR5A	Serotonin 5a (5-HT5a) receptor	6	n/a	CD19+_BCells(neg._sel.)	6.35	0.09	0.52	n/a
HTR5A	Serotonin 5a (5-HT5a) receptor	6	n/a	CD33+_Myeloid	7.50	0.96	5.74	n/a
HTR5A	Serotonin 5a (5-HT5a) receptor	6	n/a	CD34+	7.50	0.96	5.74	n/a
HTR5A	Serotonin 5a (5-HT5a) receptor	6	n/a	CD4+_Tcells	6.45	0.16	0.97	n/a
HTR5A	Serotonin 5a (5-HT5a) receptor	6	n/a	CD56+_NKCells	7.05	0.62	3.70	n/a
HTR5A	Serotonin 5a (5-HT5a) receptor	6	n/a	CD71+_EarlyErythroid	5.70	-0.41	-2.43	n/a
HTR5A	Serotonin 5a (5-HT5a) receptor	6	n/a	CD8+_Tcells	5.60	-0.48	-2.89	n/a
HTR5A	Serotonin 5a (5-HT5a) receptor	6	n/a	Cerebellum	5.10	-0.86	-5.16	n/a
HTR5A	Serotonin 5a (5-HT5a) receptor	6	n/a	CerebellumPeduncles	7.45	0.92	5.52	n/a
HTR5A	Serotonin 5a (5-HT5a) receptor	6	n/a	CiliaryGanglion	4.45	-1.35	-8.11	n/a
HTR5A	Serotonin 5a (5-HT5a) receptor	6	n/a	CingulateCortex	6.40	0.12	0.75	n/a
HTR5A	Serotonin 5a (5-HT5a) receptor	6	n/a	Colon	6.10	-0.10	-0.62	n/a
HTR5A	Serotonin 5a (5-HT5a) receptor	6	n/a	DorsalRootGanglion	4.60	-1.24	-7.43	n/a
HTR5A	Serotonin 5a (5-HT5a) receptor	6	n/a	Fetalbrain	5.90	-0.25	-1.52	n/a
HTR5A	Serotonin 5a (5-HT5a) receptor	6	n/a	Fetalliver	5.35	-0.67	-4.02	n/a
HTR5A	Serotonin 5a (5-HT5a) receptor	6	n/a	Fetallung	4.95	-0.97	-5.84	n/a
HTR5A	Serotonin 5a (5-HT5a) receptor	6	n/a	FetalThyroid	5.95	-0.22	-1.30	n/a
HTR5A	Serotonin 5a (5-HT5a) receptor	6	n/a	GlobusPallidus	4.60	-1.24	-7.43	n/a
HTR5A	Serotonin 5a (5-HT5a) receptor	6	n/a	Heart	9.60	2.55	15.28	n/a
HTR5A	Serotonin 5a (5-HT5a) receptor	6	n/a	Hypothalamus	6.40	0.12	0.75	n/a
HTR5A	Serotonin 5a (5-HT5a) receptor	6	n/a	Kidney	6.45	0.16	0.97	n/a
HTR5A	Serotonin 5a (5-HT5a) receptor	6	n/a	Liver	9.65	2.58	15.51	n/a
HTR5A	Serotonin 5a (5-HT5a) receptor	6	n/a	Lung	6.55	0.24	1.43	n/a
HTR5A	Serotonin 5a (5-HT5a) receptor	6	n/a	Lymphnode	5.25	-0.75	-4.48	n/a
HTR5A	Serotonin 5a (5-HT5a) receptor	6	n/a	MedullaOblongata	5.45	-0.59	-3.57	n/a
HTR5A	Serotonin 5a (5-HT5a) receptor	6	n/a	OccipitalLobe	5.60	-0.48	-2.89	n/a
HTR5A	Serotonin 5a (5-HT5a) receptor	6	n/a	OlfactoryBulb	4.75	-1.12	-6.75	n/a
HTR5A	Serotonin 5a (5-HT5a) receptor	6	n/a	Ovary	4.10	-1.62	-9.70	n/a
HTR5A	Serotonin 5a (5-HT5a) receptor	6	n/a	Pancreas	4.95	-0.97	-5.84	n/a
HTR5A	Serotonin 5a (5-HT5a) receptor	6	n/a	PancreaticIslet	6.45	0.16	0.97	n/a
HTR5A	Serotonin 5a (5-HT5a) receptor	6	n/a	ParietalLobe	6.90	0.50	3.02	n/a
HTR5A	Serotonin 5a (5-HT5a) receptor	6	n/a	Pineal_day	7.36	0.85	5.11	n/a

S2 Table. Target-tissue analysis for clozapine and chlorpromazine

Gene Symbol	Gene Name	Affinity (-log10(Ki))		Tissue	Expression		Combined Score (-log10(Ki)*Z)	
		clozapine	chlorpromazine		Raw-numbers	Z-score	clozapine	chlorpromazine
HTR5A	Serotonin 5a (5-HT5a) receptor	6	n/a	Pineal_night	7.22	0.75	4.47	n/a
HTR5A	Serotonin 5a (5-HT5a) receptor	6	n/a	Pituitary	8.40	1.64	9.83	n/a
HTR5A	Serotonin 5a (5-HT5a) receptor	6	n/a	Placenta	5.95	-0.22	-1.30	n/a
HTR5A	Serotonin 5a (5-HT5a) receptor	6	n/a	Pons	5.95	-0.22	-1.30	n/a
HTR5A	Serotonin 5a (5-HT5a) receptor	6	n/a	PrefrontalCortex	7.40	0.88	5.29	n/a
HTR5A	Serotonin 5a (5-HT5a) receptor	6	n/a	Prostate	6.95	0.54	3.25	n/a
HTR5A	Serotonin 5a (5-HT5a) receptor	6	n/a	Retina	7.30	0.81	4.84	n/a
HTR5A	Serotonin 5a (5-HT5a) receptor	6	n/a	Salivarygland	5.60	-0.48	-2.89	n/a
HTR5A	Serotonin 5a (5-HT5a) receptor	6	n/a	SkeletalMuscle	12.30	4.59	27.55	n/a
HTR5A	Serotonin 5a (5-HT5a) receptor	6	n/a	Skin	4.60	-1.24	-7.43	n/a
HTR5A	Serotonin 5a (5-HT5a) receptor	6	n/a	Small_intestine	5.90	-0.25	-1.52	n/a
HTR5A	Serotonin 5a (5-HT5a) receptor	6	n/a	SmoothMuscle	6.75	0.39	2.34	n/a
HTR5A	Serotonin 5a (5-HT5a) receptor	6	n/a	Spinalcord	6.50	0.20	1.20	n/a
HTR5A	Serotonin 5a (5-HT5a) receptor	6	n/a	SubthalamicNucleus	5.50	-0.56	-3.34	n/a
HTR5A	Serotonin 5a (5-HT5a) receptor	6	n/a	SuperiorCervicalGanglion	8.10	1.41	8.47	n/a
HTR5A	Serotonin 5a (5-HT5a) receptor	6	n/a	TemporalLobe	5.65	-0.44	-2.66	n/a
HTR5A	Serotonin 5a (5-HT5a) receptor	6	n/a	Testis	5.20	-0.78	-4.70	n/a
HTR5A	Serotonin 5a (5-HT5a) receptor	6	n/a	TestisGermCell	4.95	-0.97	-5.84	n/a
HTR5A	Serotonin 5a (5-HT5a) receptor	6	n/a	TestisIntersitial	4.95	-0.97	-5.84	n/a
HTR5A	Serotonin 5a (5-HT5a) receptor	6	n/a	TestisLeydigCell	6.05	-0.14	-0.84	n/a
HTR5A	Serotonin 5a (5-HT5a) receptor	6	n/a	TestisSeminiferousTubule	5.40	-0.63	-3.79	n/a
HTR5A	Serotonin 5a (5-HT5a) receptor	6	n/a	Thalamus	6.20	-0.03	-0.16	n/a
HTR5A	Serotonin 5a (5-HT5a) receptor	6	n/a	Thymus	4.75	-1.12	-6.75	n/a
HTR5A	Serotonin 5a (5-HT5a) receptor	6	n/a	Thyroid	7.40	0.88	5.29	n/a
HTR5A	Serotonin 5a (5-HT5a) receptor	6	n/a	Tongue	7.30	0.81	4.84	n/a
HTR5A	Serotonin 5a (5-HT5a) receptor	6	n/a	Tonsil	5.95	-0.22	-1.30	n/a
HTR5A	Serotonin 5a (5-HT5a) receptor	6	n/a	Trachea	5.05	-0.90	-5.38	n/a
HTR5A	Serotonin 5a (5-HT5a) receptor	6	n/a	TrigeminalGanglion	6.20	-0.03	-0.16	n/a
HTR5A	Serotonin 5a (5-HT5a) receptor	6	n/a	Uterus	4.95	-0.97	-5.84	n/a
HTR5A	Serotonin 5a (5-HT5a) receptor	6	n/a	UterusCorpus	7.05	0.62	3.70	n/a
HTR5A	Serotonin 5a (5-HT5a) receptor	6	n/a	WholeBlood	6.45	0.16	0.97	n/a
HTR5A	Serotonin 5a (5-HT5a) receptor	6	n/a	Wholebrain	5.05	-0.90	-5.38	n/a
HTR6	Serotonin 6 (5-HT6) receptor	8.397940009	8.397940009	Adipocyte	40.15	-0.18	-1.55	-1.55
HTR6	Serotonin 6 (5-HT6) receptor	8.397940009	8.397940009	AdrenalCortex	58.15	1.55	12.98	12.98
HTR6	Serotonin 6 (5-HT6) receptor	8.397940009	8.397940009	Adrenalgland	38.15	-0.38	-3.16	-3.16
HTR6	Serotonin 6 (5-HT6) receptor	8.397940009	8.397940009	Amygdala	33.80	-0.79	-6.67	-6.67
HTR6	Serotonin 6 (5-HT6) receptor	8.397940009	8.397940009	Appendix	44.50	0.23	1.96	1.96
HTR6	Serotonin 6 (5-HT6) receptor	8.397940009	8.397940009	AtrioventricularNode	63.45	2.05	17.26	17.26
HTR6	Serotonin 6 (5-HT6) receptor	8.397940009	8.397940009	BDCA4+_DentriticCells	24.80	-1.66	-13.94	-13.94
HTR6	Serotonin 6 (5-HT6) receptor	8.397940009	8.397940009	Bonemarrow	41.70	-0.04	-0.30	-0.30
HTR6	Serotonin 6 (5-HT6) receptor	8.397940009	8.397940009	BronchialEpithelialCells	33.90	-0.78	-6.59	-6.59
HTR6	Serotonin 6 (5-HT6) receptor	8.397940009	8.397940009	CardiacMyocytes	72.70	2.94	24.72	24.72
HTR6	Serotonin 6 (5-HT6) receptor	8.397940009	8.397940009	Caudatenucleus	36.50	-0.54	-4.49	-4.49
HTR6	Serotonin 6 (5-HT6) receptor	8.397940009	8.397940009	CD105+_Endothelial	33.10	-0.86	-7.24	-7.24
HTR6	Serotonin 6 (5-HT6) receptor	8.397940009	8.397940009	CD14+_Monocytes	36.20	-0.56	-4.74	-4.74
HTR6	Serotonin 6 (5-HT6) receptor	8.397940009	8.397940009	CD19+_BCells(neg._sel.)	38.60	-0.33	-2.80	-2.80
HTR6	Serotonin 6 (5-HT6) receptor	8.397940009	8.397940009	CD33+_Myeloid	38.90	-0.30	-2.56	-2.56
HTR6	Serotonin 6 (5-HT6) receptor	8.397940009	8.397940009	CD34+	39.25	-0.27	-2.27	-2.27
HTR6	Serotonin 6 (5-HT6) receptor	8.397940009	8.397940009	CD4+_Tcells	36.75	-0.51	-4.29	-4.29
HTR6	Serotonin 6 (5-HT6) receptor	8.397940009	8.397940009	CD56+_NKCells	30.50	-1.11	-9.34	-9.34
HTR6	Serotonin 6 (5-HT6) receptor	8.397940009	8.397940009	CD71+_EarlyErythroid	38.60	-0.33	-2.80	-2.80
HTR6	Serotonin 6 (5-HT6) receptor	8.397940009	8.397940009	CD8+_Tcells	28.35	-1.32	-11.07	-11.07
HTR6	Serotonin 6 (5-HT6) receptor	8.397940009	8.397940009	Cerebellum	38.40	-0.35	-2.96	-2.96

S2 Table. Target-tissue analysis for clozapine and chlorpromazine

Gene Symbol	Gene Name	Affinity (-log10(Ki))		Tissue	Expression		Combined Score (-log10(Ki)*Z)	
		clozapine	chlorpromazine		Raw-numbers	Z-score	clozapine	chlorpromazine
HTR6	Serotonin 6 (5-HT6) receptor	8.397940009	8.397940009	CerebellumPeduncles	53.75	1.12	9.43	9.43
HTR6	Serotonin 6 (5-HT6) receptor	8.397940009	8.397940009	CiliaryGanglion	48.75	0.64	5.39	5.39
HTR6	Serotonin 6 (5-HT6) receptor	8.397940009	8.397940009	CingulateCortex	55.15	1.26	10.56	10.56
HTR6	Serotonin 6 (5-HT6) receptor	8.397940009	8.397940009	Colon	27.65	-1.39	-11.64	-11.64
HTR6	Serotonin 6 (5-HT6) receptor	8.397940009	8.397940009	DorsalRootGanglion	45.75	0.35	2.97	2.97
HTR6	Serotonin 6 (5-HT6) receptor	8.397940009	8.397940009	Fetalbrain	40.85	-0.12	-0.98	-0.98
HTR6	Serotonin 6 (5-HT6) receptor	8.397940009	8.397940009	Fetalliver	34.75	-0.70	-5.91	-5.91
HTR6	Serotonin 6 (5-HT6) receptor	8.397940009	8.397940009	Fetallung	33.90	-0.78	-6.59	-6.59
HTR6	Serotonin 6 (5-HT6) receptor	8.397940009	8.397940009	FetalThyroid	41.80	-0.03	-0.22	-0.22
HTR6	Serotonin 6 (5-HT6) receptor	8.397940009	8.397940009	GlobusPallidus	47.85	0.56	4.67	4.67
HTR6	Serotonin 6 (5-HT6) receptor	8.397940009	8.397940009	Heart	66.40	2.34	19.64	19.64
HTR6	Serotonin 6 (5-HT6) receptor	8.397940009	8.397940009	Hypothalamus	43.25	0.11	0.95	0.95
HTR6	Serotonin 6 (5-HT6) receptor	8.397940009	8.397940009	Kidney	42.00	-0.01	-0.06	-0.06
HTR6	Serotonin 6 (5-HT6) receptor	8.397940009	8.397940009	Liver	52.95	1.05	8.78	8.78
HTR6	Serotonin 6 (5-HT6) receptor	8.397940009	8.397940009	Lung	41.05	-0.10	-0.82	-0.82
HTR6	Serotonin 6 (5-HT6) receptor	8.397940009	8.397940009	Lymphnode	32.95	-0.88	-7.36	-7.36
HTR6	Serotonin 6 (5-HT6) receptor	8.397940009	8.397940009	MedullaOblongata	40.45	-0.16	-1.31	-1.31
HTR6	Serotonin 6 (5-HT6) receptor	8.397940009	8.397940009	OccipitalLobe	40.85	-0.12	-0.98	-0.98
HTR6	Serotonin 6 (5-HT6) receptor	8.397940009	8.397940009	OlfactoryBulb	31.70	-1.00	-8.37	-8.37
HTR6	Serotonin 6 (5-HT6) receptor	8.397940009	8.397940009	Ovary	33.15	-0.86	-7.20	-7.20
HTR6	Serotonin 6 (5-HT6) receptor	8.397940009	8.397940009	Pancreas	34.35	-0.74	-6.23	-6.23
HTR6	Serotonin 6 (5-HT6) receptor	8.397940009	8.397940009	PancreaticIslet	44.20	0.20	1.72	1.72
HTR6	Serotonin 6 (5-HT6) receptor	8.397940009	8.397940009	ParietalLobe	47.35	0.51	4.26	4.26
HTR6	Serotonin 6 (5-HT6) receptor	8.397940009	8.397940009	Pineal_day	41.90	-0.02	-0.14	-0.14
HTR6	Serotonin 6 (5-HT6) receptor	8.397940009	8.397940009	Pineal_night	37.18	-0.47	-3.94	-3.94
HTR6	Serotonin 6 (5-HT6) receptor	8.397940009	8.397940009	Pituitary	53.20	1.07	8.98	8.98
HTR6	Serotonin 6 (5-HT6) receptor	8.397940009	8.397940009	Placenta	34.35	-0.74	-6.23	-6.23
HTR6	Serotonin 6 (5-HT6) receptor	8.397940009	8.397940009	Pons	41.50	-0.05	-0.46	-0.46
HTR6	Serotonin 6 (5-HT6) receptor	8.397940009	8.397940009	PrefrontalCortex	42.90	0.08	0.67	0.67
HTR6	Serotonin 6 (5-HT6) receptor	8.397940009	8.397940009	Prostate	44.95	0.28	2.33	2.33
HTR6	Serotonin 6 (5-HT6) receptor	8.397940009	8.397940009	Retina	48.43	0.61	5.13	5.13
HTR6	Serotonin 6 (5-HT6) receptor	8.397940009	8.397940009	Salivarygland	36.80	-0.51	-4.25	-4.25
HTR6	Serotonin 6 (5-HT6) receptor	8.397940009	8.397940009	SkeletalMuscle	75.80	3.24	27.22	27.22
HTR6	Serotonin 6 (5-HT6) receptor	8.397940009	8.397940009	Skin	39.25	-0.27	-2.27	-2.27
HTR6	Serotonin 6 (5-HT6) receptor	8.397940009	8.397940009	Small_intestine	29.55	-1.20	-10.10	-10.10
HTR6	Serotonin 6 (5-HT6) receptor	8.397940009	8.397940009	SmoothMuscle	43.35	0.12	1.03	1.03
HTR6	Serotonin 6 (5-HT6) receptor	8.397940009	8.397940009	Spinalcord	42.35	0.03	0.23	0.23
HTR6	Serotonin 6 (5-HT6) receptor	8.397940009	8.397940009	SubthalamicNucleus	44.20	0.20	1.72	1.72
HTR6	Serotonin 6 (5-HT6) receptor	8.397940009	8.397940009	SuperiorCervicalGanglion	62.15	1.93	16.21	16.21
HTR6	Serotonin 6 (5-HT6) receptor	8.397940009	8.397940009	TemporalLobe	46.00	0.38	3.17	3.17
HTR6	Serotonin 6 (5-HT6) receptor	8.397940009	8.397940009	Testis	34.55	-0.72	-6.07	-6.07
HTR6	Serotonin 6 (5-HT6) receptor	8.397940009	8.397940009	TestisGermCell	33.85	-0.79	-6.63	-6.63
HTR6	Serotonin 6 (5-HT6) receptor	8.397940009	8.397940009	TestisIntersitial	38.45	-0.35	-2.92	-2.92
HTR6	Serotonin 6 (5-HT6) receptor	8.397940009	8.397940009	TestisLeydigCell	44.15	0.20	1.68	1.68
HTR6	Serotonin 6 (5-HT6) receptor	8.397940009	8.397940009	TestisSeminiferousTubule	42.10	0.00	0.03	0.03
HTR6	Serotonin 6 (5-HT6) receptor	8.397940009	8.397940009	Thalamus	46.45	0.42	3.54	3.54
HTR6	Serotonin 6 (5-HT6) receptor	8.397940009	8.397940009	Thymus	26.45	-1.50	-12.60	-12.60
HTR6	Serotonin 6 (5-HT6) receptor	8.397940009	8.397940009	Thyroid	40.85	-0.12	-0.98	-0.98
HTR6	Serotonin 6 (5-HT6) receptor	8.397940009	8.397940009	Tongue	55.00	1.24	10.44	10.44
HTR6	Serotonin 6 (5-HT6) receptor	8.397940009	8.397940009	Tonsil	47.95	0.57	4.75	4.75
HTR6	Serotonin 6 (5-HT6) receptor	8.397940009	8.397940009	Trachea	34.95	-0.68	-5.74	-5.74
HTR6	Serotonin 6 (5-HT6) receptor	8.397940009	8.397940009	TrigeminalGanglion	67.65	2.46	20.65	20.65
HTR6	Serotonin 6 (5-HT6) receptor	8.397940009	8.397940009	Uterus	29.05	-1.25	-10.51	-10.51

S2 Table. Target-tissue analysis for clozapine and chlorpromazine

Gene Symbol	Gene Name	Affinity (-log10(Ki))		Tissue	Expression		Combined Score (-log10(Ki)*Z)	
		clozapine	chlorpromazine		Raw-numbers	Z-score	clozapine	chlorpromazine
HTR6	Serotonin 6 (5-HT6) receptor	8.397940009	8.397940009	UterusCorpus	39.50	-0.25	-2.07	-2.07
HTR6	Serotonin 6 (5-HT6) receptor	8.397940009	8.397940009	WholeBlood	41.05	-0.10	-0.82	-0.82
HTR6	Serotonin 6 (5-HT6) receptor	8.397940009	8.397940009	Wholebrain	26.10	-1.53	-12.89	-12.89
HTR7	Serotonin 7 (5-HT7) receptor	8.045757491	7.568636236	Adipocyte	7.25	-0.47	-3.75	-3.52
HTR7	Serotonin 7 (5-HT7) receptor	8.045757491	7.568636236	AdrenalCortex	9.45	0.82	6.61	6.22
HTR7	Serotonin 7 (5-HT7) receptor	8.045757491	7.568636236	Adrenalgland	7.20	-0.49	-3.98	-3.74
HTR7	Serotonin 7 (5-HT7) receptor	8.045757491	7.568636236	Amygdala	7.70	-0.20	-1.63	-1.53
HTR7	Serotonin 7 (5-HT7) receptor	8.045757491	7.568636236	Appendix	9.50	0.85	6.85	6.44
HTR7	Serotonin 7 (5-HT7) receptor	8.045757491	7.568636236	AtrioventricularNode	8.35	0.18	1.43	1.35
HTR7	Serotonin 7 (5-HT7) receptor	8.045757491	7.568636236	BDCA4+_DentriticCells	7.35	-0.41	-3.27	-3.08
HTR7	Serotonin 7 (5-HT7) receptor	8.045757491	7.568636236	Bonemarrow	8.65	0.35	2.84	2.68
HTR7	Serotonin 7 (5-HT7) receptor	8.045757491	7.568636236	BronchialEpithelialCells	7.25	-0.47	-3.75	-3.52
HTR7	Serotonin 7 (5-HT7) receptor	8.045757491	7.568636236	CardiacMyocytes	14.90	4.01	32.26	30.35
HTR7	Serotonin 7 (5-HT7) receptor	8.045757491	7.568636236	Caudatenucleus	6.95	-0.64	-5.16	-4.85
HTR7	Serotonin 7 (5-HT7) receptor	8.045757491	7.568636236	CD105+_Endothelial	7.55	-0.29	-2.33	-2.19
HTR7	Serotonin 7 (5-HT7) receptor	8.045757491	7.568636236	CD14+_Monocytes	8.20	0.09	0.73	0.68
HTR7	Serotonin 7 (5-HT7) receptor	8.045757491	7.568636236	CD19+_BCells(neg._sel.)	7.80	-0.14	-1.16	-1.09
HTR7	Serotonin 7 (5-HT7) receptor	8.045757491	7.568636236	CD33+_Myeloid	9.10	0.62	4.96	4.67
HTR7	Serotonin 7 (5-HT7) receptor	8.045757491	7.568636236	CD34+	9.10	0.62	4.96	4.67
HTR7	Serotonin 7 (5-HT7) receptor	8.045757491	7.568636236	CD4+_Tcells	7.70	-0.20	-1.63	-1.53
HTR7	Serotonin 7 (5-HT7) receptor	8.045757491	7.568636236	CD56+_NKCells	8.35	0.18	1.43	1.35
HTR7	Serotonin 7 (5-HT7) receptor	8.045757491	7.568636236	CD71+_EarlyErythroid	7.55	-0.29	-2.33	-2.19
HTR7	Serotonin 7 (5-HT7) receptor	8.045757491	7.568636236	CD8+_Tcells	6.75	-0.76	-6.10	-5.74
HTR7	Serotonin 7 (5-HT7) receptor	8.045757491	7.568636236	Cerebellum	6.60	-0.85	-6.80	-6.40
HTR7	Serotonin 7 (5-HT7) receptor	8.045757491	7.568636236	CerebellumPeduncles	9.55	0.88	7.08	6.66
HTR7	Serotonin 7 (5-HT7) receptor	8.045757491	7.568636236	CiliaryGanglion	6.50	-0.90	-7.28	-6.84
HTR7	Serotonin 7 (5-HT7) receptor	8.045757491	7.568636236	CingulateCortex	8.95	0.53	4.26	4.00
HTR7	Serotonin 7 (5-HT7) receptor	8.045757491	7.568636236	Colon	7.65	-0.23	-1.86	-1.75
HTR7	Serotonin 7 (5-HT7) receptor	8.045757491	7.568636236	DorsalRootGanglion	7.35	-0.41	-3.27	-3.08
HTR7	Serotonin 7 (5-HT7) receptor	8.045757491	7.568636236	Fetalbrain	7.25	-0.47	-3.75	-3.52
HTR7	Serotonin 7 (5-HT7) receptor	8.045757491	7.568636236	Fetalliver	7.05	-0.58	-4.69	-4.41
HTR7	Serotonin 7 (5-HT7) receptor	8.045757491	7.568636236	Fetallung	6.05	-1.17	-9.39	-8.84
HTR7	Serotonin 7 (5-HT7) receptor	8.045757491	7.568636236	FetalThyroid	8.45	0.24	1.90	1.79
HTR7	Serotonin 7 (5-HT7) receptor	8.045757491	7.568636236	GlobusPallidus	6.30	-1.02	-8.22	-7.73
HTR7	Serotonin 7 (5-HT7) receptor	8.045757491	7.568636236	Heart	14.60	3.83	30.85	29.02
HTR7	Serotonin 7 (5-HT7) receptor	8.045757491	7.568636236	Hypothalamus	7.85	-0.11	-0.92	-0.87
HTR7	Serotonin 7 (5-HT7) receptor	8.045757491	7.568636236	Kidney	7.35	-0.41	-3.27	-3.08
HTR7	Serotonin 7 (5-HT7) receptor	8.045757491	7.568636236	Liver	12.55	2.64	21.20	19.94
HTR7	Serotonin 7 (5-HT7) receptor	8.045757491	7.568636236	Lung	8.30	0.15	1.20	1.13
HTR7	Serotonin 7 (5-HT7) receptor	8.045757491	7.568636236	Lymphnode	6.15	-1.11	-8.92	-8.39
HTR7	Serotonin 7 (5-HT7) receptor	8.045757491	7.568636236	MedullaOblongata	6.85	-0.70	-5.63	-5.29
HTR7	Serotonin 7 (5-HT7) receptor	8.045757491	7.568636236	OccipitalLobe	6.85	-0.70	-5.63	-5.29
HTR7	Serotonin 7 (5-HT7) receptor	8.045757491	7.568636236	OlfactoryBulb	5.90	-1.26	-10.10	-9.50
HTR7	Serotonin 7 (5-HT7) receptor	8.045757491	7.568636236	Ovary	6.60	-0.85	-6.80	-6.40
HTR7	Serotonin 7 (5-HT7) receptor	8.045757491	7.568636236	Pancreas	6.80	-0.73	-5.86	-5.52
HTR7	Serotonin 7 (5-HT7) receptor	8.045757491	7.568636236	PancreaticIslet	8.80	0.44	3.55	3.34
HTR7	Serotonin 7 (5-HT7) receptor	8.045757491	7.568636236	ParietalLobe	9.00	0.56	4.49	4.23
HTR7	Serotonin 7 (5-HT7) receptor	8.045757491	7.568636236	Pineal_day	9.44	0.82	6.56	6.17
HTR7	Serotonin 7 (5-HT7) receptor	8.045757491	7.568636236	Pineal_night	9.20	0.68	5.43	5.11
HTR7	Serotonin 7 (5-HT7) receptor	8.045757491	7.568636236	Pituitary	9.00	0.56	4.49	4.23
HTR7	Serotonin 7 (5-HT7) receptor	8.045757491	7.568636236	Placenta	7.45	-0.35	-2.80	-2.64
HTR7	Serotonin 7 (5-HT7) receptor	8.045757491	7.568636236	Pons	7.85	-0.11	-0.92	-0.87
HTR7	Serotonin 7 (5-HT7) receptor	8.045757491	7.568636236	PrefrontalCortex	9.10	0.62	4.96	4.67

S2 Table. Target-tissue analysis for clozapine and chlorpromazine

Gene Symbol	Gene Name	Affinity (-log10(Ki))		Tissue	Expression		Combined Score (-log10(Ki)*Z)	
		clozapine	chlorpromazine		Raw-numbers	Z-score	clozapine	chlorpromazine
HTR7	Serotonin 7 (5-HT7) receptor	8.045757491	7.568636236	Prostate	8.60	0.32	2.61	2.45
HTR7	Serotonin 7 (5-HT7) receptor	8.045757491	7.568636236	Retina	9.83	1.04	8.38	7.88
HTR7	Serotonin 7 (5-HT7) receptor	8.045757491	7.568636236	Salivarygland	6.65	-0.82	-6.57	-6.18
HTR7	Serotonin 7 (5-HT7) receptor	8.045757491	7.568636236	SkeletalMuscle	11.40	1.96	15.79	14.85
HTR7	Serotonin 7 (5-HT7) receptor	8.045757491	7.568636236	Skin	7.25	-0.47	-3.75	-3.52
HTR7	Serotonin 7 (5-HT7) receptor	8.045757491	7.568636236	Small_intestine	7.25	-0.47	-3.75	-3.52
HTR7	Serotonin 7 (5-HT7) receptor	8.045757491	7.568636236	SmoothMuscle	8.65	0.35	2.84	2.68
HTR7	Serotonin 7 (5-HT7) receptor	8.045757491	7.568636236	Spinalcord	8.25	0.12	0.96	0.90
HTR7	Serotonin 7 (5-HT7) receptor	8.045757491	7.568636236	SubthalamicNucleus	7.60	-0.26	-2.10	-1.97
HTR7	Serotonin 7 (5-HT7) receptor	8.045757491	7.568636236	SuperiorCervicalGanglion	10.80	1.61	12.96	12.20
HTR7	Serotonin 7 (5-HT7) receptor	8.045757491	7.568636236	TemporalLobe	7.45	-0.35	-2.80	-2.64
HTR7	Serotonin 7 (5-HT7) receptor	8.045757491	7.568636236	Testis	6.55	-0.88	-7.04	-6.62
HTR7	Serotonin 7 (5-HT7) receptor	8.045757491	7.568636236	TestisGermCell	5.90	-1.26	-10.10	-9.50
HTR7	Serotonin 7 (5-HT7) receptor	8.045757491	7.568636236	TestisIntersitial	7.15	-0.52	-4.22	-3.97
HTR7	Serotonin 7 (5-HT7) receptor	8.045757491	7.568636236	TestisLeydigCell	8.15	0.06	0.49	0.46
HTR7	Serotonin 7 (5-HT7) receptor	8.045757491	7.568636236	TestisSeminiferousTubule	6.55	-0.88	-7.04	-6.62
HTR7	Serotonin 7 (5-HT7) receptor	8.045757491	7.568636236	Thalamus	8.35	0.18	1.43	1.35
HTR7	Serotonin 7 (5-HT7) receptor	8.045757491	7.568636236	Thymus	5.50	-1.49	-11.98	-11.27
HTR7	Serotonin 7 (5-HT7) receptor	8.045757491	7.568636236	Thyroid	9.10	0.62	4.96	4.67
HTR7	Serotonin 7 (5-HT7) receptor	8.045757491	7.568636236	Tongue	8.85	0.47	3.79	3.56
HTR7	Serotonin 7 (5-HT7) receptor	8.045757491	7.568636236	Tonsil	7.70	-0.20	-1.63	-1.53
HTR7	Serotonin 7 (5-HT7) receptor	8.045757491	7.568636236	Trachea	6.35	-0.99	-7.98	-7.51
HTR7	Serotonin 7 (5-HT7) receptor	8.045757491	7.568636236	TrigeminalGanglion	9.70	0.97	7.79	7.33
HTR7	Serotonin 7 (5-HT7) receptor	8.045757491	7.568636236	Uterus	5.95	-1.23	-9.86	-9.28
HTR7	Serotonin 7 (5-HT7) receptor	8.045757491	7.568636236	UterusCorpus	7.80	-0.14	-1.16	-1.09
HTR7	Serotonin 7 (5-HT7) receptor	8.045757491	7.568636236	WholeBlood	8.25	0.12	0.96	0.90
HTR7	Serotonin 7 (5-HT7) receptor	8.045757491	7.568636236	Wholebrain	5.95	-1.23	-9.86	-9.28
KCNH2	HERG	5.511449283	5.321090295	Adipocyte	6.40	-0.44	-2.44	-2.35
KCNH2	HERG	5.511449283	5.321090295	AdrenalCortex	6.38	-0.45	-2.49	-2.40
KCNH2	HERG	5.511449283	5.321090295	Adrenalgland	6.33	-0.47	-2.59	-2.50
KCNH2	HERG	5.511449283	5.321090295	Amygdala	6.35	-0.46	-2.54	-2.45
KCNH2	HERG	5.511449283	5.321090295	Appendix	6.15	-0.53	-2.94	-2.84
KCNH2	HERG	5.511449283	5.321090295	AtrioventricularNode	6.15	-0.53	-2.94	-2.84
KCNH2	HERG	5.511449283	5.321090295	BDCA4+_DentriticCells	6.58	-0.38	-2.08	-2.01
KCNH2	HERG	5.511449283	5.321090295	Bonemarrow	9.85	0.82	4.53	4.37
KCNH2	HERG	5.511449283	5.321090295	BronchialEpithelialCells	6.25	-0.50	-2.74	-2.64
KCNH2	HERG	5.511449283	5.321090295	CardiacMyocytes	5.93	-0.62	-3.40	-3.28
KCNH2	HERG	5.511449283	5.321090295	Caudatenucleus	6.60	-0.37	-2.03	-1.96
KCNH2	HERG	5.511449283	5.321090295	CD105+_Endothelial	18.05	3.83	21.08	20.36
KCNH2	HERG	5.511449283	5.321090295	CD14+_Monocytes	6.43	-0.43	-2.39	-2.30
KCNH2	HERG	5.511449283	5.321090295	CD19+_BCells(neg._sel.)	6.10	-0.55	-3.04	-2.94
KCNH2	HERG	5.511449283	5.321090295	CD33+_Myeloid	8.88	0.46	2.56	2.47
KCNH2	HERG	5.511449283	5.321090295	CD34+	21.58	5.12	28.20	27.23
KCNH2	HERG	5.511449283	5.321090295	CD4+_Tcells	10.23	0.96	5.29	5.10
KCNH2	HERG	5.511449283	5.321090295	CD56+_NKCells	8.45	0.31	1.70	1.64
KCNH2	HERG	5.511449283	5.321090295	CD71+_EarlyErythroid	15.68	2.96	16.29	15.73
KCNH2	HERG	5.511449283	5.321090295	CD8+_Tcells	10.48	1.05	5.79	5.59
KCNH2	HERG	5.511449283	5.321090295	Cerebellum	8.20	0.22	1.20	1.16
KCNH2	HERG	5.511449283	5.321090295	CerebellumPeduncles	8.85	0.46	2.51	2.42
KCNH2	HERG	5.511449283	5.321090295	CiliaryGanglion	6.18	-0.52	-2.89	-2.79
KCNH2	HERG	5.511449283	5.321090295	CingulateCortex	7.10	-0.19	-1.02	-0.99
KCNH2	HERG	5.511449283	5.321090295	Colon	6.23	-0.51	-2.79	-2.69
KCNH2	HERG	5.511449283	5.321090295	DorsalRootGanglion	6.23	-0.51	-2.79	-2.69

S2 Table. Target-tissue analysis for clozapine and chlorpromazine

Gene Symbol	Gene Name	Affinity (-log10(Ki))		Tissue	Expression		Combined Score (-log10(Ki)*Z)	
		clozapine	chlorpromazine		Raw-numbers	Z-score	clozapine	chlorpromazine
KCNH2	HERG	5.511449283	5.321090295	Fetalbrain	6.85	-0.28	-1.53	-1.47
KCNH2	HERG	5.511449283	5.321090295	Fetalliver	8.85	0.46	2.51	2.42
KCNH2	HERG	5.511449283	5.321090295	Fetallung	6.28	-0.49	-2.69	-2.60
KCNH2	HERG	5.511449283	5.321090295	FetalThyroid	6.38	-0.45	-2.49	-2.40
KCNH2	HERG	5.511449283	5.321090295	GlobusPallidus	6.23	-0.51	-2.79	-2.69
KCNH2	HERG	5.511449283	5.321090295	Heart	9.75	0.79	4.33	4.18
KCNH2	HERG	5.511449283	5.321090295	Hypothalamus	6.43	-0.43	-2.39	-2.30
KCNH2	HERG	5.511449283	5.321090295	Kidney	6.30	-0.48	-2.64	-2.55
KCNH2	HERG	5.511449283	5.321090295	Liver	6.30	-0.48	-2.64	-2.55
KCNH2	HERG	5.511449283	5.321090295	Lung	6.18	-0.52	-2.89	-2.79
KCNH2	HERG	5.511449283	5.321090295	Lymphnode	6.23	-0.51	-2.79	-2.69
KCNH2	HERG	5.511449283	5.321090295	MedullaOblongata	6.23	-0.51	-2.79	-2.69
KCNH2	HERG	5.511449283	5.321090295	OccipitalLobe	6.35	-0.46	-2.54	-2.45
KCNH2	HERG	5.511449283	5.321090295	OlfactoryBulb	6.60	-0.37	-2.03	-1.96
KCNH2	HERG	5.511449283	5.321090295	Ovary	6.30	-0.48	-2.64	-2.55
KCNH2	HERG	5.511449283	5.321090295	Pancreas	6.33	-0.47	-2.59	-2.50
KCNH2	HERG	5.511449283	5.321090295	PancreaticIslet	7.58	-0.01	-0.06	-0.06
KCNH2	HERG	5.511449283	5.321090295	ParietalLobe	6.35	-0.46	-2.54	-2.45
KCNH2	HERG	5.511449283	5.321090295	Pineal_day	11.92	1.58	8.71	8.41
KCNH2	HERG	5.511449283	5.321090295	Pineal_night	13.33	2.10	11.55	11.16
KCNH2	HERG	5.511449283	5.321090295	Pituitary	8.73	0.41	2.26	2.18
KCNH2	HERG	5.511449283	5.321090295	Placenta	7.83	0.08	0.44	0.43
KCNH2	HERG	5.511449283	5.321090295	Pons	6.35	-0.46	-2.54	-2.45
KCNH2	HERG	5.511449283	5.321090295	PrefrontalCortex	8.23	0.23	1.25	1.21
KCNH2	HERG	5.511449283	5.321090295	Prostate	6.53	-0.40	-2.18	-2.11
KCNH2	HERG	5.511449283	5.321090295	Retina	7.34	-0.10	-0.54	-0.52
KCNH2	HERG	5.511449283	5.321090295	Salivarygland	6.18	-0.52	-2.89	-2.79
KCNH2	HERG	5.511449283	5.321090295	SkeletalMuscle	6.28	-0.49	-2.69	-2.60
KCNH2	HERG	5.511449283	5.321090295	Skin	6.20	-0.52	-2.84	-2.74
KCNH2	HERG	5.511449283	5.321090295	Small_intestine	6.28	-0.49	-2.69	-2.60
KCNH2	HERG	5.511449283	5.321090295	SmoothMuscle	6.23	-0.51	-2.79	-2.69
KCNH2	HERG	5.511449283	5.321090295	Spinalcord	7.43	-0.07	-0.37	-0.35
KCNH2	HERG	5.511449283	5.321090295	SubthalamicNucleus	6.68	-0.34	-1.88	-1.82
KCNH2	HERG	5.511449283	5.321090295	SuperiorCervicalGanglion	6.28	-0.49	-2.69	-2.60
KCNH2	HERG	5.511449283	5.321090295	TemporalLobe	6.23	-0.51	-2.79	-2.69
KCNH2	HERG	5.511449283	5.321090295	Testis	8.60	0.36	2.01	1.94
KCNH2	HERG	5.511449283	5.321090295	TestisGermCell	12.68	1.86	10.23	9.88
KCNH2	HERG	5.511449283	5.321090295	TestisIntersitial	6.30	-0.48	-2.64	-2.55
KCNH2	HERG	5.511449283	5.321090295	TestisLeydigCell	6.18	-0.52	-2.89	-2.79
KCNH2	HERG	5.511449283	5.321090295	TestisSeminiferousTubule	7.35	-0.09	-0.52	-0.50
KCNH2	HERG	5.511449283	5.321090295	Thalamus	7.90	0.11	0.59	0.57
KCNH2	HERG	5.511449283	5.321090295	Thymus	6.25	-0.50	-2.74	-2.64
KCNH2	HERG	5.511449283	5.321090295	Thyroid	6.35	-0.46	-2.54	-2.45
KCNH2	HERG	5.511449283	5.321090295	Tongue	6.28	-0.49	-2.69	-2.60
KCNH2	HERG	5.511449283	5.321090295	Tonsil	6.25	-0.50	-2.74	-2.64
KCNH2	HERG	5.511449283	5.321090295	Trachea	6.23	-0.51	-2.79	-2.69
KCNH2	HERG	5.511449283	5.321090295	TrigeminalGanglion	6.20	-0.52	-2.84	-2.74
KCNH2	HERG	5.511449283	5.321090295	Uterus	8.33	0.26	1.45	1.40
KCNH2	HERG	5.511449283	5.321090295	UterusCorpus	6.15	-0.53	-2.94	-2.84
KCNH2	HERG	5.511449283	5.321090295	WholeBlood	6.38	-0.45	-2.49	-2.40
KCNH2	HERG	5.511449283	5.321090295	Wholebrain	7.28	-0.12	-0.67	-0.65
MC3R	Melanocortin receptor 3	n/a	4.672763707	Adipocyte	4.65	0.01	n/a	0.02
MC3R	Melanocortin receptor 3	n/a	4.672763707	AdrenalCortex	5.00	0.49	n/a	2.27

S2 Table. Target-tissue analysis for clozapine and chlorpromazine

Gene Symbol	Gene Name	Affinity (-log10(Ki))		Tissue	Expression		Combined Score (-log10(Ki)*Z)	
		clozapine	chlorpromazine		Raw-numbers	Z-score	clozapine	chlorpromazine
MC3R	Melanocortin receptor 3	n/a	4.672763707	Adrenalgland	3.90	-1.03	n/a	-4.79
MC3R	Melanocortin receptor 3	n/a	4.672763707	Amygdala	4.90	0.35	n/a	1.63
MC3R	Melanocortin receptor 3	n/a	4.672763707	Appendix	4.85	0.28	n/a	1.31
MC3R	Melanocortin receptor 3	n/a	4.672763707	AtrioventricularNode	3.85	-1.09	n/a	-5.11
MC3R	Melanocortin receptor 3	n/a	4.672763707	BDCA4+_DentriticCells	5.15	0.69	n/a	3.23
MC3R	Melanocortin receptor 3	n/a	4.672763707	Bonemarrow	4.80	0.21	n/a	0.99
MC3R	Melanocortin receptor 3	n/a	4.672763707	BronchialEpithelialCells	4.65	0.01	n/a	0.02
MC3R	Melanocortin receptor 3	n/a	4.672763707	CardiacMyocytes	6.20	2.13	n/a	9.98
MC3R	Melanocortin receptor 3	n/a	4.672763707	Caudatenucleus	4.15	-0.68	n/a	-3.19
MC3R	Melanocortin receptor 3	n/a	4.672763707	CD105+_Endothelial	4.75	0.14	n/a	0.67
MC3R	Melanocortin receptor 3	n/a	4.672763707	CD14+_Monocytes	4.95	0.42	n/a	1.95
MC3R	Melanocortin receptor 3	n/a	4.672763707	CD19+_BCells(neg._sel.)	5.05	0.55	n/a	2.59
MC3R	Melanocortin receptor 3	n/a	4.672763707	CD33+_Myeloid	5.80	1.59	n/a	7.41
MC3R	Melanocortin receptor 3	n/a	4.672763707	CD34+	5.85	1.65	n/a	7.73
MC3R	Melanocortin receptor 3	n/a	4.672763707	CD4+_Tcells	4.95	0.42	n/a	1.95
MC3R	Melanocortin receptor 3	n/a	4.672763707	CD56+_NKCells	5.50	1.17	n/a	5.48
MC3R	Melanocortin receptor 3	n/a	4.672763707	CD71+_EarlyErythroid	4.35	-0.41	n/a	-1.90
MC3R	Melanocortin receptor 3	n/a	4.672763707	CD8+_Tcells	4.40	-0.34	n/a	-1.58
MC3R	Melanocortin receptor 3	n/a	4.672763707	Cerebellum	3.75	-1.23	n/a	-5.75
MC3R	Melanocortin receptor 3	n/a	4.672763707	CerebellumPeduncles	5.30	0.90	n/a	4.20
MC3R	Melanocortin receptor 3	n/a	4.672763707	CiliaryGanglion	3.40	-1.71	n/a	-8.00
MC3R	Melanocortin receptor 3	n/a	4.672763707	CingulateCortex	4.80	0.21	n/a	0.99
MC3R	Melanocortin receptor 3	n/a	4.672763707	Colon	4.70	0.07	n/a	0.34
MC3R	Melanocortin receptor 3	n/a	4.672763707	DorsalRootGanglion	3.65	-1.37	n/a	-6.40
MC3R	Melanocortin receptor 3	n/a	4.672763707	Fetalbrain	4.70	0.07	n/a	0.34
MC3R	Melanocortin receptor 3	n/a	4.672763707	Fetalliver	4.05	-0.82	n/a	-3.83
MC3R	Melanocortin receptor 3	n/a	4.672763707	Fetallung	3.80	-1.16	n/a	-5.43
MC3R	Melanocortin receptor 3	n/a	4.672763707	FetalThyroid	4.50	-0.20	n/a	-0.94
MC3R	Melanocortin receptor 3	n/a	4.672763707	GlobusPallidus	3.45	-1.64	n/a	-7.68
MC3R	Melanocortin receptor 3	n/a	4.672763707	Heart	6.10	2.00	n/a	9.33
MC3R	Melanocortin receptor 3	n/a	4.672763707	Hypothalamus	5.00	0.49	n/a	2.27
MC3R	Melanocortin receptor 3	n/a	4.672763707	Kidney	3.80	-1.16	n/a	-5.43
MC3R	Melanocortin receptor 3	n/a	4.672763707	Liver	6.15	2.07	n/a	9.65
MC3R	Melanocortin receptor 3	n/a	4.672763707	Lung	5.15	0.69	n/a	3.23
MC3R	Melanocortin receptor 3	n/a	4.672763707	Lymphnode	4.05	-0.82	n/a	-3.83
MC3R	Melanocortin receptor 3	n/a	4.672763707	MedullaOblongata	4.30	-0.48	n/a	-2.22
MC3R	Melanocortin receptor 3	n/a	4.672763707	OccipitalLobe	4.20	-0.61	n/a	-2.87
MC3R	Melanocortin receptor 3	n/a	4.672763707	OlfactoryBulb	3.65	-1.37	n/a	-6.40
MC3R	Melanocortin receptor 3	n/a	4.672763707	Ovary	3.15	-2.06	n/a	-9.61
MC3R	Melanocortin receptor 3	n/a	4.672763707	Pancreas	3.75	-1.23	n/a	-5.75
MC3R	Melanocortin receptor 3	n/a	4.672763707	PancreaticIslet	5.00	0.49	n/a	2.27
MC3R	Melanocortin receptor 3	n/a	4.672763707	ParietalLobe	4.95	0.42	n/a	1.95
MC3R	Melanocortin receptor 3	n/a	4.672763707	Pineal_day	5.76	1.53	n/a	7.15
MC3R	Melanocortin receptor 3	n/a	4.672763707	Pineal_night	5.58	1.28	n/a	6.00
MC3R	Melanocortin receptor 3	n/a	4.672763707	Pituitary	5.45	1.10	n/a	5.16
MC3R	Melanocortin receptor 3	n/a	4.672763707	Placenta	4.75	0.14	n/a	0.67
MC3R	Melanocortin receptor 3	n/a	4.672763707	Pons	4.40	-0.34	n/a	-1.58
MC3R	Melanocortin receptor 3	n/a	4.672763707	PrefrontalCortex	5.70	1.45	n/a	6.77
MC3R	Melanocortin receptor 3	n/a	4.672763707	Prostate	5.40	1.04	n/a	4.84
MC3R	Melanocortin receptor 3	n/a	4.672763707	Retina	5.63	1.34	n/a	6.28
MC3R	Melanocortin receptor 3	n/a	4.672763707	Salivarygland	3.75	-1.23	n/a	-5.75
MC3R	Melanocortin receptor 3	n/a	4.672763707	SkeletalMuscle	5.75	1.52	n/a	7.09
MC3R	Melanocortin receptor 3	n/a	4.672763707	Skin	3.75	-1.23	n/a	-5.75

S2 Table. Target-tissue analysis for clozapine and chlorpromazine

Gene Symbol	Gene Name	Affinity (-log10(Ki))		Tissue	Expression		Combined Score (-log10(Ki)*Z)	
		clozapine	chlorpromazine		Raw-numbers	Z-score	clozapine	chlorpromazine
MC3R	Melanocortin receptor 3	n/a	4.672763707	Small_intestine	4.45	-0.27	n/a	-1.26
MC3R	Melanocortin receptor 3	n/a	4.672763707	SmoothMuscle	5.30	0.90	n/a	4.20
MC3R	Melanocortin receptor 3	n/a	4.672763707	Spinalcord	4.95	0.42	n/a	1.95
MC3R	Melanocortin receptor 3	n/a	4.672763707	SubthalamicNucleus	4.25	-0.54	n/a	-2.54
MC3R	Melanocortin receptor 3	n/a	4.672763707	SuperiorCervicalGanglion	5.20	0.76	n/a	3.56
MC3R	Melanocortin receptor 3	n/a	4.672763707	TemporalLobe	4.25	-0.54	n/a	-2.54
MC3R	Melanocortin receptor 3	n/a	4.672763707	Testis	4.00	-0.89	n/a	-4.15
MC3R	Melanocortin receptor 3	n/a	4.672763707	TestisGermCell	3.95	-0.96	n/a	-4.47
MC3R	Melanocortin receptor 3	n/a	4.672763707	TestisIntersitial	4.50	-0.20	n/a	-0.94
MC3R	Melanocortin receptor 3	n/a	4.672763707	TestisLeydigCell	4.65	0.01	n/a	0.02
MC3R	Melanocortin receptor 3	n/a	4.672763707	TestisSeminiferousTubule	4.05	-0.82	n/a	-3.83
MC3R	Melanocortin receptor 3	n/a	4.672763707	Thalamus	4.70	0.07	n/a	0.34
MC3R	Melanocortin receptor 3	n/a	4.672763707	Thymus	3.70	-1.30	n/a	-6.08
MC3R	Melanocortin receptor 3	n/a	4.672763707	Thyroid	5.75	1.52	n/a	7.09
MC3R	Melanocortin receptor 3	n/a	4.672763707	Tongue	4.70	0.07	n/a	0.34
MC3R	Melanocortin receptor 3	n/a	4.672763707	Tonsil	4.60	-0.06	n/a	-0.30
MC3R	Melanocortin receptor 3	n/a	4.672763707	Trachea	3.90	-1.03	n/a	-4.79
MC3R	Melanocortin receptor 3	n/a	4.672763707	TrigeminalGanglion	4.95	0.42	n/a	1.95
MC3R	Melanocortin receptor 3	n/a	4.672763707	Uterus	3.75	-1.23	n/a	-5.75
MC3R	Melanocortin receptor 3	n/a	4.672763707	UterusCorpus	4.15	-0.68	n/a	-3.19
MC3R	Melanocortin receptor 3	n/a	4.672763707	WholeBlood	5.05	0.55	n/a	2.59
MC3R	Melanocortin receptor 3	n/a	4.672763707	Wholebrain	4.00	-0.89	n/a	-4.15
MC4R	Melanocortin receptor 4	n/a	4.835082334	Adipocyte	3.40	-0.07	n/a	-0.35
MC4R	Melanocortin receptor 4	n/a	4.835082334	AdrenalCortex	3.65	0.38	n/a	1.85
MC4R	Melanocortin receptor 4	n/a	4.835082334	Adrenalgland	2.90	-0.99	n/a	-4.77
MC4R	Melanocortin receptor 4	n/a	4.835082334	Amygdala	3.60	0.29	n/a	1.41
MC4R	Melanocortin receptor 4	n/a	4.835082334	Appendix	3.60	0.29	n/a	1.41
MC4R	Melanocortin receptor 4	n/a	4.835082334	AtrioventricularNode	2.60	-1.53	n/a	-7.42
MC4R	Melanocortin receptor 4	n/a	4.835082334	BDCA4+_DentriticCells	3.85	0.75	n/a	3.62
MC4R	Melanocortin receptor 4	n/a	4.835082334	Bonemarrow	3.40	-0.07	n/a	-0.35
MC4R	Melanocortin receptor 4	n/a	4.835082334	BronchialEpithelialCells	3.45	0.02	n/a	0.09
MC4R	Melanocortin receptor 4	n/a	4.835082334	CardiacMyocytes	4.45	1.84	n/a	8.92
MC4R	Melanocortin receptor 4	n/a	4.835082334	Caudatenucleus	3.10	-0.62	n/a	-3.00
MC4R	Melanocortin receptor 4	n/a	4.835082334	CD105+_Endothelial	3.50	0.11	n/a	0.53
MC4R	Melanocortin receptor 4	n/a	4.835082334	CD14+_Monocytes	3.70	0.47	n/a	2.30
MC4R	Melanocortin receptor 4	n/a	4.835082334	CD19+_BCells(neg._sel.)	3.70	0.47	n/a	2.30
MC4R	Melanocortin receptor 4	n/a	4.835082334	CD33+_Myeloid	4.45	1.84	n/a	8.92
MC4R	Melanocortin receptor 4	n/a	4.835082334	CD34+	4.40	1.75	n/a	8.48
MC4R	Melanocortin receptor 4	n/a	4.835082334	CD4+_Tcells	3.80	0.66	n/a	3.18
MC4R	Melanocortin receptor 4	n/a	4.835082334	CD56+_NKCells	4.15	1.30	n/a	6.27
MC4R	Melanocortin receptor 4	n/a	4.835082334	CD71+_EarlyErythroid	3.25	-0.35	n/a	-1.68
MC4R	Melanocortin receptor 4	n/a	4.835082334	CD8+_Tcells	3.35	-0.16	n/a	-0.80
MC4R	Melanocortin receptor 4	n/a	4.835082334	Cerebellum	2.80	-1.17	n/a	-5.65
MC4R	Melanocortin receptor 4	n/a	4.835082334	CerebellumPeduncles	3.95	0.93	n/a	4.50
MC4R	Melanocortin receptor 4	n/a	4.835082334	CiliaryGanglion	2.50	-1.72	n/a	-8.30
MC4R	Melanocortin receptor 4	n/a	4.835082334	CingulateCortex	3.55	0.20	n/a	0.97
MC4R	Melanocortin receptor 4	n/a	4.835082334	Colon	3.50	0.11	n/a	0.53
MC4R	Melanocortin receptor 4	n/a	4.835082334	DorsalRootGanglion	2.70	-1.35	n/a	-6.53
MC4R	Melanocortin receptor 4	n/a	4.835082334	Fetalbrain	3.50	0.11	n/a	0.53
MC4R	Melanocortin receptor 4	n/a	4.835082334	Fetalliver	3.00	-0.80	n/a	-3.89
MC4R	Melanocortin receptor 4	n/a	4.835082334	Fetallung	2.90	-0.99	n/a	-4.77
MC4R	Melanocortin receptor 4	n/a	4.835082334	FetalThyroid	3.35	-0.16	n/a	-0.80
MC4R	Melanocortin receptor 4	n/a	4.835082334	GlobusPallidus	2.50	-1.72	n/a	-8.30

S2 Table. Target-tissue analysis for clozapine and chlorpromazine

Gene Symbol	Gene Name	Affinity (-log10(Ki))		Tissue	Expression		Combined Score (-log10(Ki)*Z)	
		clozapine	chlorpromazine		Raw-numbers	Z-score	clozapine	chlorpromazine
MC4R	Melanocortin receptor 4	n/a	4.835082334	Heart	4.35	1.66	n/a	8.03
MC4R	Melanocortin receptor 4	n/a	4.835082334	Hypothalamus	3.80	0.66	n/a	3.18
MC4R	Melanocortin receptor 4	n/a	4.835082334	Kidney	2.85	-1.08	n/a	-5.21
MC4R	Melanocortin receptor 4	n/a	4.835082334	Liver	4.55	2.03	n/a	9.80
MC4R	Melanocortin receptor 4	n/a	4.835082334	Lung	3.85	0.75	n/a	3.62
MC4R	Melanocortin receptor 4	n/a	4.835082334	Lymphnode	3.00	-0.80	n/a	-3.89
MC4R	Melanocortin receptor 4	n/a	4.835082334	MedullaOblongata	3.15	-0.53	n/a	-2.56
MC4R	Melanocortin receptor 4	n/a	4.835082334	OccipitalLobe	3.10	-0.62	n/a	-3.00
MC4R	Melanocortin receptor 4	n/a	4.835082334	OlfactoryBulb	2.75	-1.26	n/a	-6.09
MC4R	Melanocortin receptor 4	n/a	4.835082334	Ovary	2.35	-1.99	n/a	-9.63
MC4R	Melanocortin receptor 4	n/a	4.835082334	Pancreas	2.80	-1.17	n/a	-5.65
MC4R	Melanocortin receptor 4	n/a	4.835082334	PancreaticIslet	3.75	0.57	n/a	2.74
MC4R	Melanocortin receptor 4	n/a	4.835082334	ParietalLobe	3.65	0.38	n/a	1.85
MC4R	Melanocortin receptor 4	n/a	4.835082334	Pineal_day	4.32	1.61	n/a	7.77
MC4R	Melanocortin receptor 4	n/a	4.835082334	Pineal_night	4.24	1.46	n/a	7.06
MC4R	Melanocortin receptor 4	n/a	4.835082334	Pituitary	4.05	1.11	n/a	5.39
MC4R	Melanocortin receptor 4	n/a	4.835082334	Placenta	3.60	0.29	n/a	1.41
MC4R	Melanocortin receptor 4	n/a	4.835082334	Pons	3.30	-0.26	n/a	-1.24
MC4R	Melanocortin receptor 4	n/a	4.835082334	PrefrontalCortex	4.35	1.66	n/a	8.03
MC4R	Melanocortin receptor 4	n/a	4.835082334	Prostate	4.00	1.02	n/a	4.94
MC4R	Melanocortin receptor 4	n/a	4.835082334	Retina	4.18	1.34	n/a	6.49
MC4R	Melanocortin receptor 4	n/a	4.835082334	Salivarygland	2.80	-1.17	n/a	-5.65
MC4R	Melanocortin receptor 4	n/a	4.835082334	SkeletalMuscle	3.95	0.93	n/a	4.50
MC4R	Melanocortin receptor 4	n/a	4.835082334	Skin	2.65	-1.44	n/a	-6.98
MC4R	Melanocortin receptor 4	n/a	4.835082334	Small_intestine	3.35	-0.16	n/a	-0.80
MC4R	Melanocortin receptor 4	n/a	4.835082334	SmoothMuscle	3.90	0.84	n/a	4.06
MC4R	Melanocortin receptor 4	n/a	4.835082334	Spinalcord	3.70	0.47	n/a	2.30
MC4R	Melanocortin receptor 4	n/a	4.835082334	SubthalamicNucleus	3.15	-0.53	n/a	-2.56
MC4R	Melanocortin receptor 4	n/a	4.835082334	SuperiorCervicalGanglion	3.90	0.84	n/a	4.06
MC4R	Melanocortin receptor 4	n/a	4.835082334	TemporalLobe	3.15	-0.53	n/a	-2.56
MC4R	Melanocortin receptor 4	n/a	4.835082334	Testis	3.00	-0.80	n/a	-3.89
MC4R	Melanocortin receptor 4	n/a	4.835082334	TestisGermCell	2.90	-0.99	n/a	-4.77
MC4R	Melanocortin receptor 4	n/a	4.835082334	TestisIntersitial	2.90	-0.99	n/a	-4.77
MC4R	Melanocortin receptor 4	n/a	4.835082334	TestisLeydigCell	3.45	0.02	n/a	0.09
MC4R	Melanocortin receptor 4	n/a	4.835082334	TestisSeminiferousTubule	2.95	-0.89	n/a	-4.33
MC4R	Melanocortin receptor 4	n/a	4.835082334	Thalamus	3.55	0.20	n/a	0.97
MC4R	Melanocortin receptor 4	n/a	4.835082334	Thymus	2.75	-1.26	n/a	-6.09
MC4R	Melanocortin receptor 4	n/a	4.835082334	Thyroid	4.35	1.66	n/a	8.03
MC4R	Melanocortin receptor 4	n/a	4.835082334	Tongue	3.35	-0.16	n/a	-0.80
MC4R	Melanocortin receptor 4	n/a	4.835082334	Tonsil	3.35	-0.16	n/a	-0.80
MC4R	Melanocortin receptor 4	n/a	4.835082334	Trachea	2.90	-0.99	n/a	-4.77
MC4R	Melanocortin receptor 4	n/a	4.835082334	TrigeminalGanglion	3.50	0.11	n/a	0.53
MC4R	Melanocortin receptor 4	n/a	4.835082334	Uterus	2.90	-0.99	n/a	-4.77
MC4R	Melanocortin receptor 4	n/a	4.835082334	UterusCorpus	3.15	-0.53	n/a	-2.56
MC4R	Melanocortin receptor 4	n/a	4.835082334	WholeBlood	3.80	0.66	n/a	3.18
MC4R	Melanocortin receptor 4	n/a	4.835082334	Wholebrain	3.00	-0.80	n/a	-3.89
MC5R	Melanocortin receptor 5	n/a	5.145997767	Adipocyte	4.95	-0.06	n/a	-0.30
MC5R	Melanocortin receptor 5	n/a	5.145997767	AdrenalCortex	5.25	0.30	n/a	1.54
MC5R	Melanocortin receptor 5	n/a	5.145997767	Adrenalgland	4.20	-0.95	n/a	-4.90
MC5R	Melanocortin receptor 5	n/a	5.145997767	Amygdala	5.30	0.36	n/a	1.84
MC5R	Melanocortin receptor 5	n/a	5.145997767	Appendix	5.00	0.00	n/a	0.01
MC5R	Melanocortin receptor 5	n/a	5.145997767	AtrioventricularNode	3.80	-1.43	n/a	-7.35
MC5R	Melanocortin receptor 5	n/a	5.145997767	BDCA4+_DentriticCells	5.35	0.42	n/a	2.15

S2 Table. Target-tissue analysis for clozapine and chlorpromazine

Gene Symbol	Gene Name	Affinity (-log10(Ki))		Tissue	Expression		Combined Score (-log10(Ki)*Z)	
		clozapine	chlorpromazine		Raw-numbers	Z-score	clozapine	chlorpromazine
MC5R	Melanocortin receptor 5	n/a	5.145997767	Bonemarrow	4.90	-0.12	n/a	-0.61
MC5R	Melanocortin receptor 5	n/a	5.145997767	BronchialEpithelialCells	4.95	-0.06	n/a	-0.30
MC5R	Melanocortin receptor 5	n/a	5.145997767	CardiacMyocytes	6.45	1.73	n/a	8.89
MC5R	Melanocortin receptor 5	n/a	5.145997767	Caudatenucleus	4.50	-0.59	n/a	-3.06
MC5R	Melanocortin receptor 5	n/a	5.145997767	CD105+_Endothelial	5.05	0.06	n/a	0.31
MC5R	Melanocortin receptor 5	n/a	5.145997767	CD14+_Monocytes	5.35	0.42	n/a	2.15
MC5R	Melanocortin receptor 5	n/a	5.145997767	CD19+_BCells(neg._sel.)	5.40	0.48	n/a	2.46
MC5R	Melanocortin receptor 5	n/a	5.145997767	CD33+_Myeloid	6.50	1.79	n/a	9.20
MC5R	Melanocortin receptor 5	n/a	5.145997767	CD34+	6.45	1.73	n/a	8.89
MC5R	Melanocortin receptor 5	n/a	5.145997767	CD4+_Tcells	5.45	0.54	n/a	2.76
MC5R	Melanocortin receptor 5	n/a	5.145997767	CD56+_NKCells	6.05	1.25	n/a	6.44
MC5R	Melanocortin receptor 5	n/a	5.145997767	CD71+_EarlyErythroid	4.75	-0.30	n/a	-1.53
MC5R	Melanocortin receptor 5	n/a	5.145997767	CD8+_Tcells	4.85	-0.18	n/a	-0.91
MC5R	Melanocortin receptor 5	n/a	5.145997767	Cerebellum	4.05	-1.13	n/a	-5.82
MC5R	Melanocortin receptor 5	n/a	5.145997767	CerebellumPeduncles	5.65	0.77	n/a	3.99
MC5R	Melanocortin receptor 5	n/a	5.145997767	CiliaryGanglion	3.50	-1.79	n/a	-9.19
MC5R	Melanocortin receptor 5	n/a	5.145997767	CingulateCortex	5.10	0.12	n/a	0.62
MC5R	Melanocortin receptor 5	n/a	5.145997767	Colon	5.05	0.06	n/a	0.31
MC5R	Melanocortin receptor 5	n/a	5.145997767	DorsalRootGanglion	3.85	-1.37	n/a	-7.04
MC5R	Melanocortin receptor 5	n/a	5.145997767	Fetalbrain	5.05	0.06	n/a	0.31
MC5R	Melanocortin receptor 5	n/a	5.145997767	Fetalliver	4.30	-0.83	n/a	-4.28
MC5R	Melanocortin receptor 5	n/a	5.145997767	Fetallung	4.20	-0.95	n/a	-4.90
MC5R	Melanocortin receptor 5	n/a	5.145997767	FetalThyroid	4.90	-0.12	n/a	-0.61
MC5R	Melanocortin receptor 5	n/a	5.145997767	GlobusPallidus	3.70	-1.55	n/a	-7.96
MC5R	Melanocortin receptor 5	n/a	5.145997767	Heart	6.35	1.61	n/a	8.28
MC5R	Melanocortin receptor 5	n/a	5.145997767	Hypothalamus	5.45	0.54	n/a	2.76
MC5R	Melanocortin receptor 5	n/a	5.145997767	Kidney	4.15	-1.01	n/a	-5.20
MC5R	Melanocortin receptor 5	n/a	5.145997767	Liver	6.50	1.79	n/a	9.20
MC5R	Melanocortin receptor 5	n/a	5.145997767	Lung	5.55	0.66	n/a	3.38
MC5R	Melanocortin receptor 5	n/a	5.145997767	Lymphnode	4.35	-0.77	n/a	-3.98
MC5R	Melanocortin receptor 5	n/a	5.145997767	MedullaOblongata	4.60	-0.48	n/a	-2.45
MC5R	Melanocortin receptor 5	n/a	5.145997767	OccipitalLobe	4.50	-0.59	n/a	-3.06
MC5R	Melanocortin receptor 5	n/a	5.145997767	OlfactoryBulb	3.95	-1.25	n/a	-6.43
MC5R	Melanocortin receptor 5	n/a	5.145997767	Ovary	3.30	-2.02	n/a	-10.41
MC5R	Melanocortin receptor 5	n/a	5.145997767	Pancreas	4.00	-1.19	n/a	-6.12
MC5R	Melanocortin receptor 5	n/a	5.145997767	PancreaticIslet	5.35	0.42	n/a	2.15
MC5R	Melanocortin receptor 5	n/a	5.145997767	ParietalLobe	5.30	0.36	n/a	1.84
MC5R	Melanocortin receptor 5	n/a	5.145997767	Pineal_day	6.30	1.55	n/a	7.97
MC5R	Melanocortin receptor 5	n/a	5.145997767	Pineal_night	6.16	1.38	n/a	7.11
MC5R	Melanocortin receptor 5	n/a	5.145997767	Pituitary	5.90	1.07	n/a	5.52
MC5R	Melanocortin receptor 5	n/a	5.145997767	Placenta	5.20	0.24	n/a	1.23
MC5R	Melanocortin receptor 5	n/a	5.145997767	Pons	4.75	-0.30	n/a	-1.53
MC5R	Melanocortin receptor 5	n/a	5.145997767	PrefrontalCortex	6.25	1.49	n/a	7.66
MC5R	Melanocortin receptor 5	n/a	5.145997767	Prostate	5.90	1.07	n/a	5.52
MC5R	Melanocortin receptor 5	n/a	5.145997767	Retina	6.08	1.28	n/a	6.59
MC5R	Melanocortin receptor 5	n/a	5.145997767	Salivarygland	4.00	-1.19	n/a	-6.12
MC5R	Melanocortin receptor 5	n/a	5.145997767	SkeletalMuscle	5.75	0.89	n/a	4.60
MC5R	Melanocortin receptor 5	n/a	5.145997767	Skin	3.85	-1.37	n/a	-7.04
MC5R	Melanocortin receptor 5	n/a	5.145997767	Small_intestine	4.85	-0.18	n/a	-0.91
MC5R	Melanocortin receptor 5	n/a	5.145997767	SmoothMuscle	5.75	0.89	n/a	4.60
MC5R	Melanocortin receptor 5	n/a	5.145997767	Spinalcord	5.35	0.42	n/a	2.15
MC5R	Melanocortin receptor 5	n/a	5.145997767	SubthalamicNucleus	4.55	-0.53	n/a	-2.75
MC5R	Melanocortin receptor 5	n/a	5.145997767	SuperiorCervicalGanglion	6.75	2.08	n/a	10.73

Gene Symbol	Gene Name	Affinity (-log10(Ki))		Tissue	Expression		Combined Score (-log10(Ki)*Z)	
		clozapine	chlorpromazine		Raw-numbers	Z-score	clozapine	chlorpromazine
MC5R	Melanocortin receptor 5	n/a	5.145997767	TemporalLobe	4.60	-0.48	n/a	-2.45
MC5R	Melanocortin receptor 5	n/a	5.145997767	Testis	4.35	-0.77	n/a	-3.98
MC5R	Melanocortin receptor 5	n/a	5.145997767	TestisGermCell	4.15	-1.01	n/a	-5.20
MC5R	Melanocortin receptor 5	n/a	5.145997767	TestisIntersitial	4.20	-0.95	n/a	-4.90
MC5R	Melanocortin receptor 5	n/a	5.145997767	TestisLeydigCell	4.90	-0.12	n/a	-0.61
MC5R	Melanocortin receptor 5	n/a	5.145997767	TestisSeminiferousTubule	4.30	-0.83	n/a	-4.28
MC5R	Melanocortin receptor 5	n/a	5.145997767	Thalamus	5.05	0.06	n/a	0.31
MC5R	Melanocortin receptor 5	n/a	5.145997767	Thymus	3.95	-1.25	n/a	-6.43
MC5R	Melanocortin receptor 5	n/a	5.145997767	Thyroid	6.35	1.61	n/a	8.28
MC5R	Melanocortin receptor 5	n/a	5.145997767	Tongue	4.90	-0.12	n/a	-0.61
MC5R	Melanocortin receptor 5	n/a	5.145997767	Tonsil	4.90	-0.12	n/a	-0.61
MC5R	Melanocortin receptor 5	n/a	5.145997767	Trachea	4.20	-0.95	n/a	-4.90
MC5R	Melanocortin receptor 5	n/a	5.145997767	TrigeminalGanglion	6.25	1.49	n/a	7.66
MC5R	Melanocortin receptor 5	n/a	5.145997767	Uterus	4.15	-1.01	n/a	-5.20
MC5R	Melanocortin receptor 5	n/a	5.145997767	UterusCorpus	4.40	-0.71	n/a	-3.67
MC5R	Melanocortin receptor 5	n/a	5.145997767	WholeBlood	5.45	0.54	n/a	2.76
MC5R	Melanocortin receptor 5	n/a	5.145997767	Wholebrain	4.25	-0.89	n/a	-4.59
OPRD1	Delta opioid receptor	n/a	5.132827249	Adipocyte	4.90	-0.08	n/a	-0.42
OPRD1	Delta opioid receptor	n/a	5.132827249	AdrenalCortex	5.35	0.41	n/a	2.10
OPRD1	Delta opioid receptor	n/a	5.132827249	Adrenalgland	4.15	-0.90	n/a	-4.63
OPRD1	Delta opioid receptor	n/a	5.132827249	Amygdala	5.15	0.19	n/a	0.98
OPRD1	Delta opioid receptor	n/a	5.132827249	Appendix	5.00	0.03	n/a	0.14
OPRD1	Delta opioid receptor	n/a	5.132827249	AtrioventricularNode	3.85	-1.23	n/a	-6.31
OPRD1	Delta opioid receptor	n/a	5.132827249	BDCA4+_DentriticCells	5.20	0.24	n/a	1.26
OPRD1	Delta opioid receptor	n/a	5.132827249	Bonemarrow	4.90	-0.08	n/a	-0.42
OPRD1	Delta opioid receptor	n/a	5.132827249	BronchialEpithelialCells	4.85	-0.14	n/a	-0.71
OPRD1	Delta opioid receptor	n/a	5.132827249	CardiacMyocytes	6.25	1.39	n/a	7.14
OPRD1	Delta opioid receptor	n/a	5.132827249	Caudatenucleus	5.05	0.08	n/a	0.42
OPRD1	Delta opioid receptor	n/a	5.132827249	CD105+_Endothelial	5.05	0.08	n/a	0.42
OPRD1	Delta opioid receptor	n/a	5.132827249	CD14+_Monocytes	5.35	0.41	n/a	2.10
OPRD1	Delta opioid receptor	n/a	5.132827249	CD19+_BCells(neg._sel.)	5.40	0.46	n/a	2.38
OPRD1	Delta opioid receptor	n/a	5.132827249	CD33+_Myeloid	6.40	1.56	n/a	7.98
OPRD1	Delta opioid receptor	n/a	5.132827249	CD34+	6.05	1.17	n/a	6.02
OPRD1	Delta opioid receptor	n/a	5.132827249	CD4+_Tcells	5.40	0.46	n/a	2.38
OPRD1	Delta opioid receptor	n/a	5.132827249	CD56+_NKCells	5.65	0.74	n/a	3.78
OPRD1	Delta opioid receptor	n/a	5.132827249	CD71+_EarlyErythroid	4.65	-0.36	n/a	-1.83
OPRD1	Delta opioid receptor	n/a	5.132827249	CD8+_Tcells	4.80	-0.19	n/a	-0.99
OPRD1	Delta opioid receptor	n/a	5.132827249	Cerebellum	4.00	-1.07	n/a	-5.47
OPRD1	Delta opioid receptor	n/a	5.132827249	CerebellumPeduncles	5.65	0.74	n/a	3.78
OPRD1	Delta opioid receptor	n/a	5.132827249	CiliaryGanglion	3.65	-1.45	n/a	-7.43
OPRD1	Delta opioid receptor	n/a	5.132827249	CingulateCortex	5.05	0.08	n/a	0.42
OPRD1	Delta opioid receptor	n/a	5.132827249	Colon	4.95	-0.03	n/a	-0.14
OPRD1	Delta opioid receptor	n/a	5.132827249	DorsalRootGanglion	3.80	-1.28	n/a	-6.59
OPRD1	Delta opioid receptor	n/a	5.132827249	Fetalbrain	4.95	-0.03	n/a	-0.14
OPRD1	Delta opioid receptor	n/a	5.132827249	Fetalliver	4.30	-0.74	n/a	-3.79
OPRD1	Delta opioid receptor	n/a	5.132827249	Fetallung	4.00	-1.07	n/a	-5.47
OPRD1	Delta opioid receptor	n/a	5.132827249	FetalThyroid	4.80	-0.19	n/a	-0.99
OPRD1	Delta opioid receptor	n/a	5.132827249	GlobusPallidus	3.70	-1.39	n/a	-7.15
OPRD1	Delta opioid receptor	n/a	5.132827249	Heart	6.25	1.39	n/a	7.14
OPRD1	Delta opioid receptor	n/a	5.132827249	Hypothalamus	5.40	0.46	n/a	2.38
OPRD1	Delta opioid receptor	n/a	5.132827249	Kidney	4.05	-1.01	n/a	-5.19
OPRD1	Delta opioid receptor	n/a	5.132827249	Liver	6.50	1.66	n/a	8.54
OPRD1	Delta opioid receptor	n/a	5.132827249	Lung	5.40	0.46	n/a	2.38

S2 Table. Target-tissue analysis for clozapine and chlorpromazine

Gene Symbol	Gene Name	Affinity (-log10(Ki))		Tissue	Expression		Combined Score (-log10(Ki)*Z)	
		clozapine	chlorpromazine		Raw-numbers	Z-score	clozapine	chlorpromazine
OPRD1	Delta opioid receptor	n/a	5.132827249	Lymphnode	4.25	-0.79	n/a	-4.07
OPRD1	Delta opioid receptor	n/a	5.132827249	MedullaOblongata	4.55	-0.46	n/a	-2.39
OPRD1	Delta opioid receptor	n/a	5.132827249	OccipitalLobe	4.50	-0.52	n/a	-2.67
OPRD1	Delta opioid receptor	n/a	5.132827249	OlfactoryBulb	3.90	-1.17	n/a	-6.03
OPRD1	Delta opioid receptor	n/a	5.132827249	Ovary	3.25	-1.88	n/a	-9.67
OPRD1	Delta opioid receptor	n/a	5.132827249	Pancreas	4.00	-1.07	n/a	-5.47
OPRD1	Delta opioid receptor	n/a	5.132827249	PancreaticIslet	5.30	0.35	n/a	1.82
OPRD1	Delta opioid receptor	n/a	5.132827249	ParietalLobe	5.30	0.35	n/a	1.82
OPRD1	Delta opioid receptor	n/a	5.132827249	Pineal_day	6.26	1.40	n/a	7.20
OPRD1	Delta opioid receptor	n/a	5.132827249	Pineal_night	6.10	1.23	n/a	6.30
OPRD1	Delta opioid receptor	n/a	5.132827249	Pituitary	5.75	0.85	n/a	4.34
OPRD1	Delta opioid receptor	n/a	5.132827249	Placenta	4.95	-0.03	n/a	-0.14
OPRD1	Delta opioid receptor	n/a	5.132827249	Pons	4.75	-0.25	n/a	-1.27
OPRD1	Delta opioid receptor	n/a	5.132827249	PrefrontalCortex	5.95	1.06	n/a	5.46
OPRD1	Delta opioid receptor	n/a	5.132827249	Prostate	5.75	0.85	n/a	4.34
OPRD1	Delta opioid receptor	n/a	5.132827249	Retina	5.98	1.09	n/a	5.60
OPRD1	Delta opioid receptor	n/a	5.132827249	Salivarygland	4.00	-1.07	n/a	-5.47
OPRD1	Delta opioid receptor	n/a	5.132827249	SkeletalMuscle	5.90	1.01	n/a	5.18
OPRD1	Delta opioid receptor	n/a	5.132827249	Skin	3.90	-1.17	n/a	-6.03
OPRD1	Delta opioid receptor	n/a	5.132827249	Small_intestine	4.80	-0.19	n/a	-0.99
OPRD1	Delta opioid receptor	n/a	5.132827249	SmoothMuscle	5.65	0.74	n/a	3.78
OPRD1	Delta opioid receptor	n/a	5.132827249	Spinalcord	5.35	0.41	n/a	2.10
OPRD1	Delta opioid receptor	n/a	5.132827249	SubthalamicNucleus	4.50	-0.52	n/a	-2.67
OPRD1	Delta opioid receptor	n/a	5.132827249	SuperiorCervicalGanglion	5.70	0.79	n/a	4.06
OPRD1	Delta opioid receptor	n/a	5.132827249	TemporalLobe	4.55	-0.46	n/a	-2.39
OPRD1	Delta opioid receptor	n/a	5.132827249	Testis	4.20	-0.85	n/a	-4.35
OPRD1	Delta opioid receptor	n/a	5.132827249	TestisGermCell	4.80	-0.19	n/a	-0.99
OPRD1	Delta opioid receptor	n/a	5.132827249	TestisIntersitial	4.35	-0.68	n/a	-3.51
OPRD1	Delta opioid receptor	n/a	5.132827249	TestisLeydigCell	4.90	-0.08	n/a	-0.42
OPRD1	Delta opioid receptor	n/a	5.132827249	TestisSeminiferousTubule	4.25	-0.79	n/a	-4.07
OPRD1	Delta opioid receptor	n/a	5.132827249	Thalamus	9.35	4.78	n/a	24.52
OPRD1	Delta opioid receptor	n/a	5.132827249	Thymus	3.85	-1.23	n/a	-6.31
OPRD1	Delta opioid receptor	n/a	5.132827249	Thyroid	6.10	1.23	n/a	6.30
OPRD1	Delta opioid receptor	n/a	5.132827249	Tongue	4.90	-0.08	n/a	-0.42
OPRD1	Delta opioid receptor	n/a	5.132827249	Tonsil	4.80	-0.19	n/a	-0.99
OPRD1	Delta opioid receptor	n/a	5.132827249	Trachea	4.15	-0.90	n/a	-4.63
OPRD1	Delta opioid receptor	n/a	5.132827249	TrigeminalGanglion	5.00	0.03	n/a	0.14
OPRD1	Delta opioid receptor	n/a	5.132827249	Uterus	3.95	-1.12	n/a	-5.75
OPRD1	Delta opioid receptor	n/a	5.132827249	UterusCorpus	4.40	-0.63	n/a	-3.23
OPRD1	Delta opioid receptor	n/a	5.132827249	WholeBlood	5.20	0.24	n/a	1.26
OPRD1	Delta opioid receptor	n/a	5.132827249	Wholebrain	4.20	-0.85	n/a	-4.35
OPRK1	Kappa opioid receptor	n/a	5.353302269	Adipocyte	4.35	-0.08	n/a	-0.44
OPRK1	Kappa opioid receptor	n/a	5.353302269	AdrenalCortex	4.65	0.35	n/a	1.86
OPRK1	Kappa opioid receptor	n/a	5.353302269	Adrenalgland	3.70	-1.01	n/a	-5.42
OPRK1	Kappa opioid receptor	n/a	5.353302269	Amygdala	4.75	0.49	n/a	2.63
OPRK1	Kappa opioid receptor	n/a	5.353302269	Appendix	4.65	0.35	n/a	1.86
OPRK1	Kappa opioid receptor	n/a	5.353302269	AtrioventricularNode	3.40	-1.44	n/a	-7.72
OPRK1	Kappa opioid receptor	n/a	5.353302269	BDCA4+_DentriticCells	4.70	0.42	n/a	2.25
OPRK1	Kappa opioid receptor	n/a	5.353302269	Bonemarrow	4.50	0.13	n/a	0.71
OPRK1	Kappa opioid receptor	n/a	5.353302269	BronchialEpithelialCells	4.35	-0.08	n/a	-0.44
OPRK1	Kappa opioid receptor	n/a	5.353302269	CardiacMyocytes	5.85	2.07	n/a	11.06
OPRK1	Kappa opioid receptor	n/a	5.353302269	Caudatenucleus	3.95	-0.65	n/a	-3.50
OPRK1	Kappa opioid receptor	n/a	5.353302269	CD105+_Endothelial	4.40	-0.01	n/a	-0.05

S2 Table. Target-tissue analysis for clozapine and chlorpromazine

Gene Symbol	Gene Name	Affinity (-log10(Ki))		Tissue	Expression		Combined Score (-log10(Ki)*Z)	
		clozapine	chlorpromazine		Raw-numbers	Z-score	clozapine	chlorpromazine
OPRK1	Kappa opioid receptor	n/a	5.353302269	CD14+_Monocytes	4.65	0.35	n/a	1.86
OPRK1	Kappa opioid receptor	n/a	5.353302269	CD19+_BCells(neg._sel.)	4.60	0.28	n/a	1.48
OPRK1	Kappa opioid receptor	n/a	5.353302269	CD33+_Myeloid	5.50	1.56	n/a	8.37
OPRK1	Kappa opioid receptor	n/a	5.353302269	CD34+	5.70	1.85	n/a	9.91
OPRK1	Kappa opioid receptor	n/a	5.353302269	CD4+_Tcells	4.80	0.56	n/a	3.01
OPRK1	Kappa opioid receptor	n/a	5.353302269	CD56+_NKCells	5.05	0.92	n/a	4.93
OPRK1	Kappa opioid receptor	n/a	5.353302269	CD71+_EarlyErythroid	4.10	-0.44	n/a	-2.35
OPRK1	Kappa opioid receptor	n/a	5.353302269	CD8+_Tcells	4.20	-0.30	n/a	-1.59
OPRK1	Kappa opioid receptor	n/a	5.353302269	Cerebellum	3.60	-1.16	n/a	-6.18
OPRK1	Kappa opioid receptor	n/a	5.353302269	CerebellumPeduncles	5.15	1.06	n/a	5.69
OPRK1	Kappa opioid receptor	n/a	5.353302269	CiliaryGanglion	3.15	-1.80	n/a	-9.63
OPRK1	Kappa opioid receptor	n/a	5.353302269	CingulateCortex	4.50	0.13	n/a	0.71
OPRK1	Kappa opioid receptor	n/a	5.353302269	Colon	4.45	0.06	n/a	0.33
OPRK1	Kappa opioid receptor	n/a	5.353302269	DorsalRootGanglion	3.55	-1.23	n/a	-6.57
OPRK1	Kappa opioid receptor	n/a	5.353302269	Fetalbrain	4.45	0.06	n/a	0.33
OPRK1	Kappa opioid receptor	n/a	5.353302269	Fetalliver	3.95	-0.65	n/a	-3.50
OPRK1	Kappa opioid receptor	n/a	5.353302269	Fetallung	3.75	-0.94	n/a	-5.03
OPRK1	Kappa opioid receptor	n/a	5.353302269	FetalThyroid	4.30	-0.15	n/a	-0.82
OPRK1	Kappa opioid receptor	n/a	5.353302269	GlobusPallidus	3.35	-1.51	n/a	-8.10
OPRK1	Kappa opioid receptor	n/a	5.353302269	Heart	5.55	1.64	n/a	8.76
OPRK1	Kappa opioid receptor	n/a	5.353302269	Hypothalamus	4.75	0.49	n/a	2.63
OPRK1	Kappa opioid receptor	n/a	5.353302269	Kidney	3.70	-1.01	n/a	-5.42
OPRK1	Kappa opioid receptor	n/a	5.353302269	Liver	5.80	1.99	n/a	10.67
OPRK1	Kappa opioid receptor	n/a	5.353302269	Lung	4.90	0.71	n/a	3.78
OPRK1	Kappa opioid receptor	n/a	5.353302269	Lymphnode	3.85	-0.80	n/a	-4.27
OPRK1	Kappa opioid receptor	n/a	5.353302269	MedullaOblongata	4.30	-0.15	n/a	-0.82
OPRK1	Kappa opioid receptor	n/a	5.353302269	OccipitalLobe	4.00	-0.58	n/a	-3.12
OPRK1	Kappa opioid receptor	n/a	5.353302269	OlfactoryBulb	3.45	-1.37	n/a	-7.33
OPRK1	Kappa opioid receptor	n/a	5.353302269	Ovary	3.00	-2.01	n/a	-10.78
OPRK1	Kappa opioid receptor	n/a	5.353302269	Pancreas	3.55	-1.23	n/a	-6.57
OPRK1	Kappa opioid receptor	n/a	5.353302269	PancreaticIslet	4.75	0.49	n/a	2.63
OPRK1	Kappa opioid receptor	n/a	5.353302269	ParietalLobe	4.70	0.42	n/a	2.25
OPRK1	Kappa opioid receptor	n/a	5.353302269	Pineal_day	5.46	1.51	n/a	8.07
OPRK1	Kappa opioid receptor	n/a	5.353302269	Pineal_night	5.38	1.39	n/a	7.46
OPRK1	Kappa opioid receptor	n/a	5.353302269	Pituitary	5.20	1.14	n/a	6.08
OPRK1	Kappa opioid receptor	n/a	5.353302269	Placenta	4.70	0.42	n/a	2.25
OPRK1	Kappa opioid receptor	n/a	5.353302269	Pons	4.30	-0.15	n/a	-0.82
OPRK1	Kappa opioid receptor	n/a	5.353302269	PrefrontalCortex	5.60	1.71	n/a	9.14
OPRK1	Kappa opioid receptor	n/a	5.353302269	Prostate	5.30	1.28	n/a	6.84
OPRK1	Kappa opioid receptor	n/a	5.353302269	Retina	5.40	1.42	n/a	7.61
OPRK1	Kappa opioid receptor	n/a	5.353302269	Salivarygland	3.55	-1.23	n/a	-6.57
OPRK1	Kappa opioid receptor	n/a	5.353302269	SkeletalMuscle	5.25	1.21	n/a	6.46
OPRK1	Kappa opioid receptor	n/a	5.353302269	Skin	3.40	-1.44	n/a	-7.72
OPRK1	Kappa opioid receptor	n/a	5.353302269	Small_intestine	4.30	-0.15	n/a	-0.82
OPRK1	Kappa opioid receptor	n/a	5.353302269	SmoothMuscle	5.05	0.92	n/a	4.93
OPRK1	Kappa opioid receptor	n/a	5.353302269	Spinalcord	4.65	0.35	n/a	1.86
OPRK1	Kappa opioid receptor	n/a	5.353302269	SubthalamicNucleus	4.00	-0.58	n/a	-3.12
OPRK1	Kappa opioid receptor	n/a	5.353302269	SuperiorCervicalGanglion	5.35	1.35	n/a	7.23
OPRK1	Kappa opioid receptor	n/a	5.353302269	TemporalLobe	4.05	-0.51	n/a	-2.74
OPRK1	Kappa opioid receptor	n/a	5.353302269	Testis	3.80	-0.87	n/a	-4.65
OPRK1	Kappa opioid receptor	n/a	5.353302269	TestisGermCell	3.70	-1.01	n/a	-5.42
OPRK1	Kappa opioid receptor	n/a	5.353302269	TestisIntersitial	3.75	-0.94	n/a	-5.03
OPRK1	Kappa opioid receptor	n/a	5.353302269	TestisLeydigCell	4.40	-0.01	n/a	-0.05

Gene Symbol	Gene Name	Affinity (-log10(Ki))		Tissue	Expression		Combined Score (-log10(Ki)*Z)	
		clozapine	chlorpromazine		Raw-numbers	Z-score	clozapine	chlorpromazine
OPRK1	Kappa opioid receptor	n/a	5.353302269	TestisSeminiferousTubule	3.80	-0.87	n/a	-4.65
OPRK1	Kappa opioid receptor	n/a	5.353302269	Thalamus	4.45	0.06	n/a	0.33
OPRK1	Kappa opioid receptor	n/a	5.353302269	Thymus	3.60	-1.16	n/a	-6.18
OPRK1	Kappa opioid receptor	n/a	5.353302269	Thyroid	5.60	1.71	n/a	9.14
OPRK1	Kappa opioid receptor	n/a	5.353302269	Tongue	4.30	-0.15	n/a	-0.82
OPRK1	Kappa opioid receptor	n/a	5.353302269	Tonsil	4.30	-0.15	n/a	-0.82
OPRK1	Kappa opioid receptor	n/a	5.353302269	Trachea	3.70	-1.01	n/a	-5.42
OPRK1	Kappa opioid receptor	n/a	5.353302269	TrigeminalGanglion	4.40	-0.01	n/a	-0.05
OPRK1	Kappa opioid receptor	n/a	5.353302269	Uterus	3.70	-1.01	n/a	-5.42
OPRK1	Kappa opioid receptor	n/a	5.353302269	UterusCorpus	4.05	-0.51	n/a	-2.74
OPRK1	Kappa opioid receptor	n/a	5.353302269	WholeBlood	4.75	0.49	n/a	2.63
OPRK1	Kappa opioid receptor	n/a	5.353302269	Wholebrain	3.75	-0.94	n/a	-5.03
OPRM1	Mu opioid receptor	n/a	5.233289793	Adipocyte	5.10	-0.34	n/a	-1.80
OPRM1	Mu opioid receptor	n/a	5.233289793	AdrenalCortex	5.80	0.01	n/a	0.03
OPRM1	Mu opioid receptor	n/a	5.233289793	Adrenalgland	4.45	-0.67	n/a	-3.51
OPRM1	Mu opioid receptor	n/a	5.233289793	Amygdala	5.25	-0.27	n/a	-1.41
OPRM1	Mu opioid receptor	n/a	5.233289793	Appendix	9.95	2.08	n/a	10.90
OPRM1	Mu opioid receptor	n/a	5.233289793	AtrioventricularNode	4.35	-0.72	n/a	-3.77
OPRM1	Mu opioid receptor	n/a	5.233289793	BDCA4+_DentriticCells	5.30	-0.24	n/a	-1.28
OPRM1	Mu opioid receptor	n/a	5.233289793	Bonemarrow	5.30	-0.24	n/a	-1.28
OPRM1	Mu opioid receptor	n/a	5.233289793	BronchialEpithelialCells	5.00	-0.39	n/a	-2.06
OPRM1	Mu opioid receptor	n/a	5.233289793	CardiacMyocytes	6.90	0.56	n/a	2.91
OPRM1	Mu opioid receptor	n/a	5.233289793	Caudatenucleus	4.65	-0.57	n/a	-2.98
OPRM1	Mu opioid receptor	n/a	5.233289793	CD105+_Endothelial	5.10	-0.34	n/a	-1.80
OPRM1	Mu opioid receptor	n/a	5.233289793	CD14+_Monocytes	5.45	-0.17	n/a	-0.89
OPRM1	Mu opioid receptor	n/a	5.233289793	CD19+_BCells(neg._sel.)	5.35	-0.22	n/a	-1.15
OPRM1	Mu opioid receptor	n/a	5.233289793	CD33+_Myeloid	6.45	0.33	n/a	1.73
OPRM1	Mu opioid receptor	n/a	5.233289793	CD34+	6.30	0.26	n/a	1.34
OPRM1	Mu opioid receptor	n/a	5.233289793	CD4+_Tcells	5.40	-0.19	n/a	-1.02
OPRM1	Mu opioid receptor	n/a	5.233289793	CD56+_NKCells	5.80	0.01	n/a	0.03
OPRM1	Mu opioid receptor	n/a	5.233289793	CD71+_EarlyErythroid	4.85	-0.47	n/a	-2.46
OPRM1	Mu opioid receptor	n/a	5.233289793	CD8+_Tcells	4.70	-0.54	n/a	-2.85
OPRM1	Mu opioid receptor	n/a	5.233289793	Cerebellum	5.95	0.08	n/a	0.42
OPRM1	Mu opioid receptor	n/a	5.233289793	CerebellumPeduncles	6.05	0.13	n/a	0.69
OPRM1	Mu opioid receptor	n/a	5.233289793	CiliaryGanglion	13.20	3.71	n/a	19.42
OPRM1	Mu opioid receptor	n/a	5.233289793	CingulateCortex	5.35	-0.22	n/a	-1.15
OPRM1	Mu opioid receptor	n/a	5.233289793	Colon	5.15	-0.32	n/a	-1.67
OPRM1	Mu opioid receptor	n/a	5.233289793	DorsalRootGanglion	4.30	-0.74	n/a	-3.90
OPRM1	Mu opioid receptor	n/a	5.233289793	Fetalbrain	5.05	-0.37	n/a	-1.93
OPRM1	Mu opioid receptor	n/a	5.233289793	Fetalliver	4.55	-0.62	n/a	-3.24
OPRM1	Mu opioid receptor	n/a	5.233289793	Fetallung	4.10	-0.84	n/a	-4.42
OPRM1	Mu opioid receptor	n/a	5.233289793	FetalThyroid	5.00	-0.39	n/a	-2.06
OPRM1	Mu opioid receptor	n/a	5.233289793	GlobusPallidus	3.90	-0.95	n/a	-4.95
OPRM1	Mu opioid receptor	n/a	5.233289793	Heart	7.10	0.66	n/a	3.44
OPRM1	Mu opioid receptor	n/a	5.233289793	Hypothalamus	5.55	-0.12	n/a	-0.62
OPRM1	Mu opioid receptor	n/a	5.233289793	Kidney	6.45	0.33	n/a	1.73
OPRM1	Mu opioid receptor	n/a	5.233289793	Liver	7.10	0.66	n/a	3.44
OPRM1	Mu opioid receptor	n/a	5.233289793	Lung	5.60	-0.09	n/a	-0.49
OPRM1	Mu opioid receptor	n/a	5.233289793	Lymphnode	4.35	-0.72	n/a	-3.77
OPRM1	Mu opioid receptor	n/a	5.233289793	MedullaOblongata	7.05	0.63	n/a	3.31
OPRM1	Mu opioid receptor	n/a	5.233289793	OccipitalLobe	4.60	-0.59	n/a	-3.11
OPRM1	Mu opioid receptor	n/a	5.233289793	OlfactoryBulb	4.05	-0.87	n/a	-4.55
OPRM1	Mu opioid receptor	n/a	5.233289793	Ovary	4.35	-0.72	n/a	-3.77

S2 Table. Target-tissue analysis for clozapine and chlorpromazine

Gene Symbol	Gene Name	Affinity (-log10(Ki))		Tissue	Expression		Combined Score (-log10(Ki)*Z)	
		clozapine	chlorpromazine		Raw-numbers	Z-score	clozapine	chlorpromazine
OPRM1	Mu opioid receptor	n/a	5.233289793	Pancreas	4.20	-0.79	n/a	-4.16
OPRM1	Mu opioid receptor	n/a	5.233289793	PancreaticIslet	5.55	-0.12	n/a	-0.62
OPRM1	Mu opioid receptor	n/a	5.233289793	ParietalLobe	5.75	-0.02	n/a	-0.10
OPRM1	Mu opioid receptor	n/a	5.233289793	Pineal_day	6.32	0.27	n/a	1.39
OPRM1	Mu opioid receptor	n/a	5.233289793	Pineal_night	6.28	0.25	n/a	1.29
OPRM1	Mu opioid receptor	n/a	5.233289793	Pituitary	9.20	1.71	n/a	8.94
OPRM1	Mu opioid receptor	n/a	5.233289793	Placenta	5.15	-0.32	n/a	-1.67
OPRM1	Mu opioid receptor	n/a	5.233289793	Pons	8.40	1.31	n/a	6.84
OPRM1	Mu opioid receptor	n/a	5.233289793	PrefrontalCortex	6.25	0.23	n/a	1.21
OPRM1	Mu opioid receptor	n/a	5.233289793	Prostate	5.90	0.06	n/a	0.29
OPRM1	Mu opioid receptor	n/a	5.233289793	Retina	6.23	0.22	n/a	1.14
OPRM1	Mu opioid receptor	n/a	5.233289793	Salivarygland	4.20	-0.79	n/a	-4.16
OPRM1	Mu opioid receptor	n/a	5.233289793	SkeletalMuscle	14.50	4.36	n/a	22.82
OPRM1	Mu opioid receptor	n/a	5.233289793	Skin	4.25	-0.77	n/a	-4.03
OPRM1	Mu opioid receptor	n/a	5.233289793	Small_intestine	4.95	-0.42	n/a	-2.20
OPRM1	Mu opioid receptor	n/a	5.233289793	SmoothMuscle	5.75	-0.02	n/a	-0.10
OPRM1	Mu opioid receptor	n/a	5.233289793	Spinalcord	5.50	-0.14	n/a	-0.75
OPRM1	Mu opioid receptor	n/a	5.233289793	SubthalamicNucleus	4.75	-0.52	n/a	-2.72
OPRM1	Mu opioid receptor	n/a	5.233289793	SuperiorCervicalGanglion	13.70	3.96	n/a	20.73
OPRM1	Mu opioid receptor	n/a	5.233289793	TemporalLobe	7.05	0.63	n/a	3.31
OPRM1	Mu opioid receptor	n/a	5.233289793	Testis	4.40	-0.69	n/a	-3.64
OPRM1	Mu opioid receptor	n/a	5.233289793	TestisGermCell	4.15	-0.82	n/a	-4.29
OPRM1	Mu opioid receptor	n/a	5.233289793	TestisIntersitial	5.90	0.06	n/a	0.29
OPRM1	Mu opioid receptor	n/a	5.233289793	TestisLeydigCell	5.20	-0.29	n/a	-1.54
OPRM1	Mu opioid receptor	n/a	5.233289793	TestisSeminiferousTubule	4.40	-0.69	n/a	-3.64
OPRM1	Mu opioid receptor	n/a	5.233289793	Thalamus	5.30	-0.24	n/a	-1.28
OPRM1	Mu opioid receptor	n/a	5.233289793	Thymus	3.95	-0.92	n/a	-4.81
OPRM1	Mu opioid receptor	n/a	5.233289793	Thyroid	6.35	0.28	n/a	1.47
OPRM1	Mu opioid receptor	n/a	5.233289793	Tongue	5.30	-0.24	n/a	-1.28
OPRM1	Mu opioid receptor	n/a	5.233289793	Tonsil	5.00	-0.39	n/a	-2.06
OPRM1	Mu opioid receptor	n/a	5.233289793	Trachea	4.30	-0.74	n/a	-3.90
OPRM1	Mu opioid receptor	n/a	5.233289793	TrigeminalGanglion	5.50	-0.14	n/a	-0.75
OPRM1	Mu opioid receptor	n/a	5.233289793	Uterus	4.15	-0.82	n/a	-4.29
OPRM1	Mu opioid receptor	n/a	5.233289793	UterusCorpus	7.90	1.06	n/a	5.53
OPRM1	Mu opioid receptor	n/a	5.233289793	WholeBlood	5.55	-0.12	n/a	-0.62
OPRM1	Mu opioid receptor	n/a	5.233289793	Wholebrain	4.20	-0.79	n/a	-4.16
SIGMAR1	Sigma opioid receptor	5.070581074	6.723538196	Adipocyte	37.43	1.04	5.28	7.00
SIGMAR1	Sigma opioid receptor	5.070581074	6.723538196	AdrenalCortex	27.78	0.37	1.86	2.47
SIGMAR1	Sigma opioid receptor	5.070581074	6.723538196	Adrenalgland	18.55	-0.28	-1.40	-1.86
SIGMAR1	Sigma opioid receptor	5.070581074	6.723538196	Amygdala	14.13	-0.58	-2.96	-3.93
SIGMAR1	Sigma opioid receptor	5.070581074	6.723538196	Appendix	16.93	-0.39	-1.97	-2.62
SIGMAR1	Sigma opioid receptor	5.070581074	6.723538196	AtrioventricularNode	15.68	-0.48	-2.42	-3.20
SIGMAR1	Sigma opioid receptor	5.070581074	6.723538196	BDCA4+_DentriticCells	24.90	0.17	0.85	1.12
SIGMAR1	Sigma opioid receptor	5.070581074	6.723538196	Bonemarrow	16.83	-0.40	-2.01	-2.67
SIGMAR1	Sigma opioid receptor	5.070581074	6.723538196	BronchialEpithelialCells	38.88	1.14	5.79	7.68
SIGMAR1	Sigma opioid receptor	5.070581074	6.723538196	CardiacMyocytes	33.28	0.75	3.81	5.05
SIGMAR1	Sigma opioid receptor	5.070581074	6.723538196	Caudatenucleus	13.78	-0.61	-3.09	-4.10
SIGMAR1	Sigma opioid receptor	5.070581074	6.723538196	CD105+_Endothelial	77.00	3.80	19.27	25.55
SIGMAR1	Sigma opioid receptor	5.070581074	6.723538196	CD14+_Monocytes	45.88	1.63	8.26	10.96
SIGMAR1	Sigma opioid receptor	5.070581074	6.723538196	CD19+_BCells(neg._sel.)	29.10	0.46	2.33	3.09
SIGMAR1	Sigma opioid receptor	5.070581074	6.723538196	CD33+_Myeloid	18.50	-0.28	-1.42	-1.88
SIGMAR1	Sigma opioid receptor	5.070581074	6.723538196	CD34+	60.13	2.62	13.30	17.64
SIGMAR1	Sigma opioid receptor	5.070581074	6.723538196	CD4+_Tcells	22.53	0.00	0.01	0.01

S2 Table. Target-tissue analysis for clozapine and chlorpromazine

Gene Symbol	Gene Name	Affinity (-log10(Ki))		Tissue	Expression		Combined Score (-log10(Ki)*Z)	
		clozapine	chlorpromazine		Raw-numbers	Z-score	clozapine	chlorpromazine
SIGMAR1	Sigma opioid receptor	5.070581074	6.723538196	CD56+_NKCells	37.35	1.04	5.25	6.96
SIGMAR1	Sigma opioid receptor	5.070581074	6.723538196	CD71+_EarlyErythroid	43.70	1.48	7.49	9.94
SIGMAR1	Sigma opioid receptor	5.070581074	6.723538196	CD8+_Tcells	16.45	-0.42	-2.14	-2.84
SIGMAR1	Sigma opioid receptor	5.070581074	6.723538196	Cerebellum	12.23	-0.72	-3.64	-4.82
SIGMAR1	Sigma opioid receptor	5.070581074	6.723538196	CerebellumPeduncles	23.18	0.05	0.24	0.31
SIGMAR1	Sigma opioid receptor	5.070581074	6.723538196	CiliaryGanglion	15.15	-0.51	-2.60	-3.45
SIGMAR1	Sigma opioid receptor	5.070581074	6.723538196	CingulateCortex	19.40	-0.22	-1.10	-1.46
SIGMAR1	Sigma opioid receptor	5.070581074	6.723538196	Colon	19.55	-0.21	-1.05	-1.39
SIGMAR1	Sigma opioid receptor	5.070581074	6.723538196	DorsalRootGanglion	13.33	-0.64	-3.25	-4.31
SIGMAR1	Sigma opioid receptor	5.070581074	6.723538196	Fetalbrain	17.93	-0.32	-1.62	-2.15
SIGMAR1	Sigma opioid receptor	5.070581074	6.723538196	Fetalliver	35.18	0.88	4.48	5.94
SIGMAR1	Sigma opioid receptor	5.070581074	6.723538196	Fetallung	13.13	-0.65	-3.32	-4.40
SIGMAR1	Sigma opioid receptor	5.070581074	6.723538196	FetalThyroid	18.65	-0.27	-1.36	-1.81
SIGMAR1	Sigma opioid receptor	5.070581074	6.723538196	GlobusPallidus	14.43	-0.56	-2.86	-3.79
SIGMAR1	Sigma opioid receptor	5.070581074	6.723538196	Heart	25.35	0.20	1.01	1.33
SIGMAR1	Sigma opioid receptor	5.070581074	6.723538196	Hypothalamus	17.95	-0.32	-1.61	-2.14
SIGMAR1	Sigma opioid receptor	5.070581074	6.723538196	Kidney	14.33	-0.57	-2.89	-3.84
SIGMAR1	Sigma opioid receptor	5.070581074	6.723538196	Liver	103.38	5.64	28.60	37.92
SIGMAR1	Sigma opioid receptor	5.070581074	6.723538196	Lung	17.58	-0.34	-1.74	-2.31
SIGMAR1	Sigma opioid receptor	5.070581074	6.723538196	Lymphnode	13.00	-0.66	-3.36	-4.46
SIGMAR1	Sigma opioid receptor	5.070581074	6.723538196	MedullaOblongata	14.18	-0.58	-2.95	-3.91
SIGMAR1	Sigma opioid receptor	5.070581074	6.723538196	OccipitalLobe	18.23	-0.30	-1.51	-2.01
SIGMAR1	Sigma opioid receptor	5.070581074	6.723538196	OlfactoryBulb	17.28	-0.37	-1.85	-2.45
SIGMAR1	Sigma opioid receptor	5.070581074	6.723538196	Ovary	13.98	-0.60	-3.02	-4.00
SIGMAR1	Sigma opioid receptor	5.070581074	6.723538196	Pancreas	15.40	-0.50	-2.51	-3.33
SIGMAR1	Sigma opioid receptor	5.070581074	6.723538196	PancreaticIslet	17.53	-0.35	-1.76	-2.34
SIGMAR1	Sigma opioid receptor	5.070581074	6.723538196	ParietalLobe	18.08	-0.31	-1.57	-2.08
SIGMAR1	Sigma opioid receptor	5.070581074	6.723538196	Pineal_day	25.75	0.23	1.15	1.52
SIGMAR1	Sigma opioid receptor	5.070581074	6.723538196	Pineal_night	22.88	0.03	0.13	0.17
SIGMAR1	Sigma opioid receptor	5.070581074	6.723538196	Pituitary	17.68	-0.34	-1.71	-2.27
SIGMAR1	Sigma opioid receptor	5.070581074	6.723538196	Placenta	29.03	0.45	2.30	3.06
SIGMAR1	Sigma opioid receptor	5.070581074	6.723538196	Pons	17.20	-0.37	-1.88	-2.49
SIGMAR1	Sigma opioid receptor	5.070581074	6.723538196	PrefrontalCortex	20.00	-0.17	-0.89	-1.18
SIGMAR1	Sigma opioid receptor	5.070581074	6.723538196	Prostate	26.80	0.30	1.52	2.01
SIGMAR1	Sigma opioid receptor	5.070581074	6.723538196	Retina	25.31	0.20	0.99	1.32
SIGMAR1	Sigma opioid receptor	5.070581074	6.723538196	Salivarygland	12.60	-0.69	-3.50	-4.65
SIGMAR1	Sigma opioid receptor	5.070581074	6.723538196	SkeletalMuscle	23.75	0.09	0.44	0.58
SIGMAR1	Sigma opioid receptor	5.070581074	6.723538196	Skin	14.65	-0.55	-2.78	-3.69
SIGMAR1	Sigma opioid receptor	5.070581074	6.723538196	Small_intestine	28.73	0.43	2.20	2.92
SIGMAR1	Sigma opioid receptor	5.070581074	6.723538196	SmoothMuscle	24.20	0.12	0.60	0.79
SIGMAR1	Sigma opioid receptor	5.070581074	6.723538196	Spinalcord	21.80	-0.05	-0.25	-0.33
SIGMAR1	Sigma opioid receptor	5.070581074	6.723538196	SubthalamicNucleus	15.08	-0.52	-2.63	-3.49
SIGMAR1	Sigma opioid receptor	5.070581074	6.723538196	SuperiorCervicalGanglion	20.88	-0.11	-0.58	-0.77
SIGMAR1	Sigma opioid receptor	5.070581074	6.723538196	TemporalLobe	22.63	0.01	0.04	0.05
SIGMAR1	Sigma opioid receptor	5.070581074	6.723538196	Testis	12.05	-0.73	-3.70	-4.90
SIGMAR1	Sigma opioid receptor	5.070581074	6.723538196	TestisGermCell	15.40	-0.50	-2.51	-3.33
SIGMAR1	Sigma opioid receptor	5.070581074	6.723538196	TestisIntersitial	13.18	-0.65	-3.30	-4.38
SIGMAR1	Sigma opioid receptor	5.070581074	6.723538196	TestisLeydigCell	18.28	-0.30	-1.50	-1.99
SIGMAR1	Sigma opioid receptor	5.070581074	6.723538196	TestisSeminiferousTubule	14.25	-0.58	-2.92	-3.87
SIGMAR1	Sigma opioid receptor	5.070581074	6.723538196	Thalamus	21.68	-0.06	-0.29	-0.39
SIGMAR1	Sigma opioid receptor	5.070581074	6.723538196	Thymus	12.53	-0.70	-3.53	-4.68
SIGMAR1	Sigma opioid receptor	5.070581074	6.723538196	Thyroid	22.48	0.00	-0.01	-0.02
SIGMAR1	Sigma opioid receptor	5.070581074	6.723538196	Tongue	15.75	-0.47	-2.39	-3.17

S2 Table. Target-tissue analysis for clozapine and chlorpromazine

Gene Symbol	Gene Name	Affinity (-log10(Ki))		Tissue	Expression		Combined Score (-log10(Ki)*Z)	
		clozapine	chlorpromazine		Raw-numbers	Z-score	clozapine	chlorpromazine
SIGMAR1	Sigma opioid receptor	5.070581074	6.723538196	Tonsil	16.83	-0.40	-2.01	-2.67
SIGMAR1	Sigma opioid receptor	5.070581074	6.723538196	Trachea	12.38	-0.71	-3.58	-4.75
SIGMAR1	Sigma opioid receptor	5.070581074	6.723538196	TrigeminalGanglion	18.65	-0.27	-1.36	-1.81
SIGMAR1	Sigma opioid receptor	5.070581074	6.723538196	Uterus	11.28	-0.78	-3.97	-5.27
SIGMAR1	Sigma opioid receptor	5.070581074	6.723538196	UterusCorpus	12.83	-0.68	-3.42	-4.54
SIGMAR1	Sigma opioid receptor	5.070581074	6.723538196	WholeBlood	14.18	-0.58	-2.95	-3.91
SIGMAR1	Sigma opioid receptor	5.070581074	6.723538196	Wholebrain	15.40	-0.50	-2.51	-3.33
SLC6A2	Norepinephrine transporter	5.836242476	7.721246399	Adipocyte	5.70	-0.33	-1.91	-2.52
SLC6A2	Norepinephrine transporter	5.836242476	7.721246399	AdrenalCortex	6.90	0.80	4.69	6.21
SLC6A2	Norepinephrine transporter	5.836242476	7.721246399	Adrenalgland	5.15	-0.85	-4.93	-6.53
SLC6A2	Norepinephrine transporter	5.836242476	7.721246399	Amygdala	6.05	0.00	0.02	0.02
SLC6A2	Norepinephrine transporter	5.836242476	7.721246399	Appendix	6.70	0.62	3.59	4.75
SLC6A2	Norepinephrine transporter	5.836242476	7.721246399	AtrioventricularNode	5.85	-0.19	-1.08	-1.43
SLC6A2	Norepinephrine transporter	5.836242476	7.721246399	BDCA4+_DentriticCells	5.85	-0.19	-1.08	-1.43
SLC6A2	Norepinephrine transporter	5.836242476	7.721246399	Bonemarrow	6.65	0.57	3.32	4.39
SLC6A2	Norepinephrine transporter	5.836242476	7.721246399	BronchialEpithelialCells	5.60	-0.42	-2.46	-3.25
SLC6A2	Norepinephrine transporter	5.836242476	7.721246399	CardiacMyocytes	8.00	1.84	10.74	14.21
SLC6A2	Norepinephrine transporter	5.836242476	7.721246399	Caudatenucleus	5.25	-0.75	-4.38	-5.80
SLC6A2	Norepinephrine transporter	5.836242476	7.721246399	CD105+_Endothelial	5.80	-0.23	-1.36	-1.80
SLC6A2	Norepinephrine transporter	5.836242476	7.721246399	CD14+_Monocytes	6.20	0.14	0.84	1.11
SLC6A2	Norepinephrine transporter	5.836242476	7.721246399	CD19+_BCells(neg._sel.)	6.25	0.19	1.12	1.48
SLC6A2	Norepinephrine transporter	5.836242476	7.721246399	CD33+_Myeloid	7.50	1.37	7.99	10.58
SLC6A2	Norepinephrine transporter	5.836242476	7.721246399	CD34+	7.35	1.23	7.17	9.48
SLC6A2	Norepinephrine transporter	5.836242476	7.721246399	CD4+_Tcells	5.95	-0.09	-0.53	-0.71
SLC6A2	Norepinephrine transporter	5.836242476	7.721246399	CD56+_NKCells	6.95	0.85	4.97	6.57
SLC6A2	Norepinephrine transporter	5.836242476	7.721246399	CD71+_EarlyErythroid	5.50	-0.52	-3.01	-3.98
SLC6A2	Norepinephrine transporter	5.836242476	7.721246399	CD8+_Tcells	5.30	-0.70	-4.11	-5.44
SLC6A2	Norepinephrine transporter	5.836242476	7.721246399	Cerebellum	5.05	-0.94	-5.48	-7.26
SLC6A2	Norepinephrine transporter	5.836242476	7.721246399	CerebellumPeduncles	7.50	1.37	7.99	10.58
SLC6A2	Norepinephrine transporter	5.836242476	7.721246399	CiliaryGanglion	5.00	-0.99	-5.76	-7.62
SLC6A2	Norepinephrine transporter	5.836242476	7.721246399	CingulateCortex	6.05	0.00	0.02	0.02
SLC6A2	Norepinephrine transporter	5.836242476	7.721246399	Colon	5.80	-0.23	-1.36	-1.80
SLC6A2	Norepinephrine transporter	5.836242476	7.721246399	DorsalRootGanglion	5.60	-0.42	-2.46	-3.25
SLC6A2	Norepinephrine transporter	5.836242476	7.721246399	Fetalbrain	5.75	-0.28	-1.63	-2.16
SLC6A2	Norepinephrine transporter	5.836242476	7.721246399	Fetalliver	5.25	-0.75	-4.38	-5.80
SLC6A2	Norepinephrine transporter	5.836242476	7.721246399	Fetallung	4.60	-1.36	-7.96	-10.53
SLC6A2	Norepinephrine transporter	5.836242476	7.721246399	FetalThyroid	5.70	-0.33	-1.91	-2.52
SLC6A2	Norepinephrine transporter	5.836242476	7.721246399	GlobusPallidus	4.70	-1.27	-7.41	-9.80
SLC6A2	Norepinephrine transporter	5.836242476	7.721246399	Heart	8.15	1.98	11.57	15.31
SLC6A2	Norepinephrine transporter	5.836242476	7.721246399	Hypothalamus	6.30	0.24	1.39	1.84
SLC6A2	Norepinephrine transporter	5.836242476	7.721246399	Kidney	4.95	-1.03	-6.03	-7.98
SLC6A2	Norepinephrine transporter	5.836242476	7.721246399	Liver	8.40	2.22	12.94	17.13
SLC6A2	Norepinephrine transporter	5.836242476	7.721246399	Lung	6.45	0.38	2.22	2.93
SLC6A2	Norepinephrine transporter	5.836242476	7.721246399	Lymphnode	4.80	-1.18	-6.86	-9.07
SLC6A2	Norepinephrine transporter	5.836242476	7.721246399	MedullaOblongata	5.30	-0.70	-4.11	-5.44
SLC6A2	Norepinephrine transporter	5.836242476	7.721246399	OccipitalLobe	5.30	-0.70	-4.11	-5.44
SLC6A2	Norepinephrine transporter	5.836242476	7.721246399	OlfactoryBulb	4.85	-1.13	-6.58	-8.71
SLC6A2	Norepinephrine transporter	5.836242476	7.721246399	Ovary	4.55	-1.41	-8.23	-10.89
SLC6A2	Norepinephrine transporter	5.836242476	7.721246399	Pancreas	4.95	-1.03	-6.03	-7.98
SLC6A2	Norepinephrine transporter	5.836242476	7.721246399	PancreaticIslet	6.20	0.14	0.84	1.11
SLC6A2	Norepinephrine transporter	5.836242476	7.721246399	ParietalLobe	6.55	0.47	2.77	3.66
SLC6A2	Norepinephrine transporter	5.836242476	7.721246399	Pineal_day	7.04	0.94	5.46	7.23
SLC6A2	Norepinephrine transporter	5.836242476	7.721246399	Pineal_night	6.92	0.82	4.80	6.35

S2 Table. Target-tissue analysis for clozapine and chlorpromazine

Gene Symbol	Gene Name	Affinity (-log10(Ki))		Tissue	Expression		Combined Score (-log10(Ki)*Z)	
		clozapine	chlorpromazine		Raw-numbers	Z-score	clozapine	chlorpromazine
SLC6A2	Norepinephrine transporter	5.836242476	7.721246399	Pituitary	6.75	0.66	3.87	5.12
SLC6A2	Norepinephrine transporter	5.836242476	7.721246399	Placenta	8.15	1.98	11.57	15.31
SLC6A2	Norepinephrine transporter	5.836242476	7.721246399	Pons	5.90	-0.14	-0.81	-1.07
SLC6A2	Norepinephrine transporter	5.836242476	7.721246399	PrefrontalCortex	6.95	0.85	4.97	6.57
SLC6A2	Norepinephrine transporter	5.836242476	7.721246399	Prostate	6.70	0.62	3.59	4.75
SLC6A2	Norepinephrine transporter	5.836242476	7.721246399	Retina	7.00	0.90	5.24	6.94
SLC6A2	Norepinephrine transporter	5.836242476	7.721246399	Salivarygland	4.65	-1.32	-7.68	-10.17
SLC6A2	Norepinephrine transporter	5.836242476	7.721246399	SkeletalMuscle	9.85	3.58	20.92	27.68
SLC6A2	Norepinephrine transporter	5.836242476	7.721246399	Skin	5.50	-0.52	-3.01	-3.98
SLC6A2	Norepinephrine transporter	5.836242476	7.721246399	Small_intestine	5.60	-0.42	-2.46	-3.25
SLC6A2	Norepinephrine transporter	5.836242476	7.721246399	SmoothMuscle	6.60	0.52	3.04	4.03
SLC6A2	Norepinephrine transporter	5.836242476	7.721246399	Spinalcord	6.15	0.10	0.57	0.75
SLC6A2	Norepinephrine transporter	5.836242476	7.721246399	SubthalamicNucleus	6.15	0.10	0.57	0.75
SLC6A2	Norepinephrine transporter	5.836242476	7.721246399	SuperiorCervicalGanglion	8.25	2.08	12.12	16.03
SLC6A2	Norepinephrine transporter	5.836242476	7.721246399	TemporalLobe	5.45	-0.56	-3.28	-4.34
SLC6A2	Norepinephrine transporter	5.836242476	7.721246399	Testis	5.00	-0.99	-5.76	-7.62
SLC6A2	Norepinephrine transporter	5.836242476	7.721246399	TestisGermCell	4.85	-1.13	-6.58	-8.71
SLC6A2	Norepinephrine transporter	5.836242476	7.721246399	TestisIntersitial	5.65	-0.37	-2.18	-2.89
SLC6A2	Norepinephrine transporter	5.836242476	7.721246399	TestisLeydigCell	6.20	0.14	0.84	1.11
SLC6A2	Norepinephrine transporter	5.836242476	7.721246399	TestisSeminiferousTubule	5.00	-0.99	-5.76	-7.62
SLC6A2	Norepinephrine transporter	5.836242476	7.721246399	Thalamus	5.95	-0.09	-0.53	-0.71
SLC6A2	Norepinephrine transporter	5.836242476	7.721246399	Thymus	4.65	-1.32	-7.68	-10.17
SLC6A2	Norepinephrine transporter	5.836242476	7.721246399	Thyroid	7.00	0.90	5.24	6.94
SLC6A2	Norepinephrine transporter	5.836242476	7.721246399	Tongue	6.55	0.47	2.77	3.66
SLC6A2	Norepinephrine transporter	5.836242476	7.721246399	Tonsil	5.70	-0.33	-1.91	-2.52
SLC6A2	Norepinephrine transporter	5.836242476	7.721246399	Trachea	4.85	-1.13	-6.58	-8.71
SLC6A2	Norepinephrine transporter	5.836242476	7.721246399	TrigeminalGanglion	6.85	0.76	4.42	5.84
SLC6A2	Norepinephrine transporter	5.836242476	7.721246399	Uterus	4.55	-1.41	-8.23	-10.89
SLC6A2	Norepinephrine transporter	5.836242476	7.721246399	UterusCorpus	5.70	-0.33	-1.91	-2.52
SLC6A2	Norepinephrine transporter	5.836242476	7.721246399	WholeBlood	6.40	0.33	1.94	2.57
SLC6A2	Norepinephrine transporter	5.836242476	7.721246399	Wholebrain	4.85	-1.13	-6.58	-8.71
SLC6A3	Dopamine transporter	n/a	5.677780705	Adipocyte	4.25	0.55	n/a	3.12
SLC6A3	Dopamine transporter	n/a	5.677780705	AdrenalCortex	4.15	0.42	n/a	2.36
SLC6A3	Dopamine transporter	n/a	5.677780705	Adrenalgland	3.10	-0.99	n/a	-5.63
SLC6A3	Dopamine transporter	n/a	5.677780705	Amygdala	3.90	0.08	n/a	0.46
SLC6A3	Dopamine transporter	n/a	5.677780705	Appendix	3.80	-0.05	n/a	-0.30
SLC6A3	Dopamine transporter	n/a	5.677780705	AtrioventricularNode	3.20	-0.86	n/a	-4.87
SLC6A3	Dopamine transporter	n/a	5.677780705	BDCA4+_DentriticCells	4.10	0.35	n/a	1.98
SLC6A3	Dopamine transporter	n/a	5.677780705	Bonemarrow	3.60	-0.32	n/a	-1.83
SLC6A3	Dopamine transporter	n/a	5.677780705	BronchialEpithelialCells	3.65	-0.25	n/a	-1.45
SLC6A3	Dopamine transporter	n/a	5.677780705	CardiacMyocytes	4.55	0.95	n/a	5.41
SLC6A3	Dopamine transporter	n/a	5.677780705	Caudatenucleus	3.35	-0.66	n/a	-3.73
SLC6A3	Dopamine transporter	n/a	5.677780705	CD105+_Endothelial	3.75	-0.12	n/a	-0.68
SLC6A3	Dopamine transporter	n/a	5.677780705	CD14+_Monocytes	4.00	0.21	n/a	1.22
SLC6A3	Dopamine transporter	n/a	5.677780705	CD19+_BCells(neg._sel.)	3.95	0.15	n/a	0.84
SLC6A3	Dopamine transporter	n/a	5.677780705	CD33+_Myeloid	4.80	1.29	n/a	7.31
SLC6A3	Dopamine transporter	n/a	5.677780705	CD34+	4.80	1.29	n/a	7.31
SLC6A3	Dopamine transporter	n/a	5.677780705	CD4+_Tcells	4.05	0.28	n/a	1.60
SLC6A3	Dopamine transporter	n/a	5.677780705	CD56+_NKCells	4.45	0.82	n/a	4.65
SLC6A3	Dopamine transporter	n/a	5.677780705	CD71+_EarlyErythroid	3.45	-0.52	n/a	-2.97
SLC6A3	Dopamine transporter	n/a	5.677780705	CD8+_Tcells	3.60	-0.32	n/a	-1.83
SLC6A3	Dopamine transporter	n/a	5.677780705	Cerebellum	3.00	-1.13	n/a	-6.39
SLC6A3	Dopamine transporter	n/a	5.677780705	CerebellumPeduncles	4.65	1.09	n/a	6.17

S2 Table. Target-tissue analysis for clozapine and chlorpromazine

Gene Symbol	Gene Name	Affinity (-log10(Ki))		Tissue	Expression		Combined Score (-log10(Ki)*Z)	
		clozapine	chlorpromazine		Raw-numbers	Z-score	clozapine	chlorpromazine
SLC6A3	Dopamine transporter	n/a	5.677780705	CiliaryGanglion	4.90	1.42	n/a	8.07
SLC6A3	Dopamine transporter	n/a	5.677780705	CingulateCortex	3.90	0.08	n/a	0.46
SLC6A3	Dopamine transporter	n/a	5.677780705	Colon	3.70	-0.19	n/a	-1.06
SLC6A3	Dopamine transporter	n/a	5.677780705	DorsalRootGanglion	3.60	-0.32	n/a	-1.83
SLC6A3	Dopamine transporter	n/a	5.677780705	Fetalbrain	3.65	-0.25	n/a	-1.45
SLC6A3	Dopamine transporter	n/a	5.677780705	Fetalliver	3.15	-0.93	n/a	-5.25
SLC6A3	Dopamine transporter	n/a	5.677780705	Fetallung	3.15	-0.93	n/a	-5.25
SLC6A3	Dopamine transporter	n/a	5.677780705	FetalThyroid	3.90	0.08	n/a	0.46
SLC6A3	Dopamine transporter	n/a	5.677780705	GlobusPallidus	2.75	-1.46	n/a	-8.30
SLC6A3	Dopamine transporter	n/a	5.677780705	Heart	4.60	1.02	n/a	5.79
SLC6A3	Dopamine transporter	n/a	5.677780705	Hypothalamus	7.45	4.84	n/a	27.49
SLC6A3	Dopamine transporter	n/a	5.677780705	Kidney	3.00	-1.13	n/a	-6.39
SLC6A3	Dopamine transporter	n/a	5.677780705	Liver	4.85	1.35	n/a	7.69
SLC6A3	Dopamine transporter	n/a	5.677780705	Lung	4.10	0.35	n/a	1.98
SLC6A3	Dopamine transporter	n/a	5.677780705	Lymphnode	3.15	-0.93	n/a	-5.25
SLC6A3	Dopamine transporter	n/a	5.677780705	MedullaOblongata	3.40	-0.59	n/a	-3.35
SLC6A3	Dopamine transporter	n/a	5.677780705	OccipitalLobe	3.40	-0.59	n/a	-3.35
SLC6A3	Dopamine transporter	n/a	5.677780705	OlfactoryBulb	3.00	-1.13	n/a	-6.39
SLC6A3	Dopamine transporter	n/a	5.677780705	Ovary	2.45	-1.86	n/a	-10.58
SLC6A3	Dopamine transporter	n/a	5.677780705	Pancreas	3.25	-0.79	n/a	-4.49
SLC6A3	Dopamine transporter	n/a	5.677780705	PancreaticIslet	4.00	0.21	n/a	1.22
SLC6A3	Dopamine transporter	n/a	5.677780705	ParietalLobe	3.95	0.15	n/a	0.84
SLC6A3	Dopamine transporter	n/a	5.677780705	Pineal_day	4.60	1.02	n/a	5.79
SLC6A3	Dopamine transporter	n/a	5.677780705	Pineal_night	4.54	0.94	n/a	5.33
SLC6A3	Dopamine transporter	n/a	5.677780705	Pituitary	4.40	0.75	n/a	4.27
SLC6A3	Dopamine transporter	n/a	5.677780705	Placenta	3.90	0.08	n/a	0.46
SLC6A3	Dopamine transporter	n/a	5.677780705	Pons	3.70	-0.19	n/a	-1.06
SLC6A3	Dopamine transporter	n/a	5.677780705	PrefrontalCortex	4.60	1.02	n/a	5.79
SLC6A3	Dopamine transporter	n/a	5.677780705	Prostate	4.35	0.68	n/a	3.88
SLC6A3	Dopamine transporter	n/a	5.677780705	Retina	4.48	0.85	n/a	4.84
SLC6A3	Dopamine transporter	n/a	5.677780705	Salivarygland	3.10	-0.99	n/a	-5.63
SLC6A3	Dopamine transporter	n/a	5.677780705	SkeletalMuscle	5.85	2.70	n/a	15.31
SLC6A3	Dopamine transporter	n/a	5.677780705	Skin	2.85	-1.33	n/a	-7.54
SLC6A3	Dopamine transporter	n/a	5.677780705	Small_intestine	3.60	-0.32	n/a	-1.83
SLC6A3	Dopamine transporter	n/a	5.677780705	SmoothMuscle	4.25	0.55	n/a	3.12
SLC6A3	Dopamine transporter	n/a	5.677780705	Spinalcord	3.95	0.15	n/a	0.84
SLC6A3	Dopamine transporter	n/a	5.677780705	SubthalamicNucleus	3.60	-0.32	n/a	-1.83
SLC6A3	Dopamine transporter	n/a	5.677780705	SuperiorCervicalGanglion	4.15	0.42	n/a	2.36
SLC6A3	Dopamine transporter	n/a	5.677780705	TemporalLobe	3.40	-0.59	n/a	-3.35
SLC6A3	Dopamine transporter	n/a	5.677780705	Testis	3.20	-0.86	n/a	-4.87
SLC6A3	Dopamine transporter	n/a	5.677780705	TestisGermCell	3.20	-0.86	n/a	-4.87
SLC6A3	Dopamine transporter	n/a	5.677780705	TestisIntersitial	3.10	-0.99	n/a	-5.63
SLC6A3	Dopamine transporter	n/a	5.677780705	TestisLeydigCell	3.65	-0.25	n/a	-1.45
SLC6A3	Dopamine transporter	n/a	5.677780705	TestisSeminiferousTubule	3.20	-0.86	n/a	-4.87
SLC6A3	Dopamine transporter	n/a	5.677780705	Thalamus	3.90	0.08	n/a	0.46
SLC6A3	Dopamine transporter	n/a	5.677780705	Thymus	3.30	-0.72	n/a	-4.11
SLC6A3	Dopamine transporter	n/a	5.677780705	Thyroid	4.65	1.09	n/a	6.17
SLC6A3	Dopamine transporter	n/a	5.677780705	Tongue	3.65	-0.25	n/a	-1.45
SLC6A3	Dopamine transporter	n/a	5.677780705	Tonsil	3.60	-0.32	n/a	-1.83
SLC6A3	Dopamine transporter	n/a	5.677780705	Trachea	3.15	-0.93	n/a	-5.25
SLC6A3	Dopamine transporter	n/a	5.677780705	TrigeminalGanglion	4.40	0.75	n/a	4.27
SLC6A3	Dopamine transporter	n/a	5.677780705	Uterus	3.05	-1.06	n/a	-6.01
SLC6A3	Dopamine transporter	n/a	5.677780705	UterusCorpus	3.60	-0.32	n/a	-1.83

S2 Table. Target-tissue analysis for clozapine and chlorpromazine

Gene Symbol	Gene Name	Affinity (-log10(Ki))		Tissue	Expression		Combined Score (-log10(Ki)*Z)	
		clozapine	chlorpromazine		Raw-numbers	Z-score	clozapine	chlorpromazine
SLC6A3	Dopamine transporter	n/a	5.677780705	WholeBlood	4.05	0.28	n/a	1.60
SLC6A3	Dopamine transporter	n/a	5.677780705	Wholebrain	3.20	-0.86	n/a	-4.87
SLC6A4	Serotonin transporter	6.537602002	7.677780705	Adipocyte	4.85	-0.23	-1.51	-1.78
SLC6A4	Serotonin transporter	6.537602002	7.677780705	AdrenalCortex	5.25	-0.17	-1.08	-1.27
SLC6A4	Serotonin transporter	6.537602002	7.677780705	Adrenalgland	4.20	-0.34	-2.21	-2.60
SLC6A4	Serotonin transporter	6.537602002	7.677780705	Amygdala	5.20	-0.17	-1.14	-1.34
SLC6A4	Serotonin transporter	6.537602002	7.677780705	Appendix	5.10	-0.19	-1.24	-1.46
SLC6A4	Serotonin transporter	6.537602002	7.677780705	AtrioventricularNode	3.75	-0.41	-2.70	-3.17
SLC6A4	Serotonin transporter	6.537602002	7.677780705	BDCA4+_DentriticCells	4.95	-0.22	-1.41	-1.65
SLC6A4	Serotonin transporter	6.537602002	7.677780705	Bonemarrow	6.20	-0.01	-0.06	-0.07
SLC6A4	Serotonin transporter	6.537602002	7.677780705	BronchialEpithelialCells	4.80	-0.24	-1.57	-1.84
SLC6A4	Serotonin transporter	6.537602002	7.677780705	CardiacMyocytes	6.10	-0.03	-0.17	-0.20
SLC6A4	Serotonin transporter	6.537602002	7.677780705	Caudatenucleus	5.55	-0.12	-0.76	-0.89
SLC6A4	Serotonin transporter	6.537602002	7.677780705	CD105+_Endothelial	4.85	-0.23	-1.51	-1.78
SLC6A4	Serotonin transporter	6.537602002	7.677780705	CD14+_Monocytes	5.10	-0.19	-1.24	-1.46
SLC6A4	Serotonin transporter	6.537602002	7.677780705	CD19+_BCells(neg._sel.)	5.20	-0.17	-1.14	-1.34
SLC6A4	Serotonin transporter	6.537602002	7.677780705	CD33+_Myeloid	6.25	0.00	-0.01	-0.01
SLC6A4	Serotonin transporter	6.537602002	7.677780705	CD34+	6.25	0.00	-0.01	-0.01
SLC6A4	Serotonin transporter	6.537602002	7.677780705	CD4+_Tcells	5.35	-0.15	-0.98	-1.15
SLC6A4	Serotonin transporter	6.537602002	7.677780705	CD56+_NKCells	5.80	-0.08	-0.49	-0.58
SLC6A4	Serotonin transporter	6.537602002	7.677780705	CD71+_EarlyErythroid	4.65	-0.26	-1.73	-2.03
SLC6A4	Serotonin transporter	6.537602002	7.677780705	CD8+_Tcells	4.50	-0.29	-1.89	-2.22
SLC6A4	Serotonin transporter	6.537602002	7.677780705	Cerebellum	9.85	0.59	3.86	4.53
SLC6A4	Serotonin transporter	6.537602002	7.677780705	CerebellumPeduncles	5.60	-0.11	-0.71	-0.83
SLC6A4	Serotonin transporter	6.537602002	7.677780705	CiliaryGanglion	3.55	-0.45	-2.91	-3.42
SLC6A4	Serotonin transporter	6.537602002	7.677780705	CingulateCortex	5.05	-0.20	-1.30	-1.53
SLC6A4	Serotonin transporter	6.537602002	7.677780705	Colon	4.95	-0.22	-1.41	-1.65
SLC6A4	Serotonin transporter	6.537602002	7.677780705	DorsalRootGanglion	4.25	-0.33	-2.16	-2.53
SLC6A4	Serotonin transporter	6.537602002	7.677780705	Fetalbrain	5.15	-0.18	-1.19	-1.40
SLC6A4	Serotonin transporter	6.537602002	7.677780705	Fetalliver	6.70	0.07	0.47	0.56
SLC6A4	Serotonin transporter	6.537602002	7.677780705	Fetallung	4.15	-0.35	-2.27	-2.66
SLC6A4	Serotonin transporter	6.537602002	7.677780705	FetalThyroid	4.80	-0.24	-1.57	-1.84
SLC6A4	Serotonin transporter	6.537602002	7.677780705	GlobusPallidus	3.65	-0.43	-2.80	-3.29
SLC6A4	Serotonin transporter	6.537602002	7.677780705	Heart	6.45	0.03	0.21	0.24
SLC6A4	Serotonin transporter	6.537602002	7.677780705	Hypothalamus	5.20	-0.17	-1.14	-1.34
SLC6A4	Serotonin transporter	6.537602002	7.677780705	Kidney	4.10	-0.35	-2.32	-2.72
SLC6A4	Serotonin transporter	6.537602002	7.677780705	Liver	7.10	0.14	0.90	1.06
SLC6A4	Serotonin transporter	6.537602002	7.677780705	Lung	10.45	0.69	4.50	5.29
SLC6A4	Serotonin transporter	6.537602002	7.677780705	Lymphnode	4.20	-0.34	-2.21	-2.60
SLC6A4	Serotonin transporter	6.537602002	7.677780705	MedullaOblongata	4.85	-0.23	-1.51	-1.78
SLC6A4	Serotonin transporter	6.537602002	7.677780705	OccipitalLobe	4.45	-0.30	-1.94	-2.28
SLC6A4	Serotonin transporter	6.537602002	7.677780705	OlfactoryBulb	3.90	-0.39	-2.53	-2.98
SLC6A4	Serotonin transporter	6.537602002	7.677780705	Ovary	3.30	-0.49	-3.18	-3.73
SLC6A4	Serotonin transporter	6.537602002	7.677780705	Pancreas	4.00	-0.37	-2.43	-2.85
SLC6A4	Serotonin transporter	6.537602002	7.677780705	PancreaticIslet	5.35	-0.15	-0.98	-1.15
SLC6A4	Serotonin transporter	6.537602002	7.677780705	ParietalLobe	5.55	-0.12	-0.76	-0.89
SLC6A4	Serotonin transporter	6.537602002	7.677780705	Pineal_day	17.88	1.91	12.48	14.66
SLC6A4	Serotonin transporter	6.537602002	7.677780705	Pineal_night	53.12	7.70	50.34	59.12
SLC6A4	Serotonin transporter	6.537602002	7.677780705	Pituitary	5.85	-0.07	-0.44	-0.52
SLC6A4	Serotonin transporter	6.537602002	7.677780705	Placenta	12.30	0.99	6.49	7.62
SLC6A4	Serotonin transporter	6.537602002	7.677780705	Pons	5.40	-0.14	-0.92	-1.08
SLC6A4	Serotonin transporter	6.537602002	7.677780705	PrefrontalCortex	6.10	-0.03	-0.17	-0.20
SLC6A4	Serotonin transporter	6.537602002	7.677780705	Prostate	5.65	-0.10	-0.65	-0.77

S2 Table. Target-tissue analysis for clozapine and chlorpromazine

Gene Symbol	Gene Name	Affinity (-log10(Ki))		Tissue	Expression		Combined Score (-log10(Ki)*Z)	
		clozapine	chlorpromazine		Raw-numbers	Z-score	clozapine	chlorpromazine
SLC6A4	Serotonin transporter	6.537602002	7.677780705	Retina	5.88	-0.06	-0.41	-0.48
SLC6A4	Serotonin transporter	6.537602002	7.677780705	Salivarygland	3.95	-0.38	-2.48	-2.91
SLC6A4	Serotonin transporter	6.537602002	7.677780705	SkeletalMuscle	5.90	-0.06	-0.39	-0.45
SLC6A4	Serotonin transporter	6.537602002	7.677780705	Skin	3.75	-0.41	-2.70	-3.17
SLC6A4	Serotonin transporter	6.537602002	7.677780705	Small_intestine	21.55	2.51	16.43	19.29
SLC6A4	Serotonin transporter	6.537602002	7.677780705	SmoothMuscle	5.45	-0.13	-0.87	-1.02
SLC6A4	Serotonin transporter	6.537602002	7.677780705	Spinalcord	5.30	-0.16	-1.03	-1.21
SLC6A4	Serotonin transporter	6.537602002	7.677780705	SubthalamicNucleus	4.60	-0.27	-1.78	-2.09
SLC6A4	Serotonin transporter	6.537602002	7.677780705	SuperiorCervicalGanglion	10.25	0.66	4.29	5.04
SLC6A4	Serotonin transporter	6.537602002	7.677780705	TemporalLobe	4.45	-0.30	-1.94	-2.28
SLC6A4	Serotonin transporter	6.537602002	7.677780705	Testis	4.30	-0.32	-2.10	-2.47
SLC6A4	Serotonin transporter	6.537602002	7.677780705	TestisGermCell	4.00	-0.37	-2.43	-2.85
SLC6A4	Serotonin transporter	6.537602002	7.677780705	TestisIntersitial	4.15	-0.35	-2.27	-2.66
SLC6A4	Serotonin transporter	6.537602002	7.677780705	TestisLeydigCell	4.85	-0.23	-1.51	-1.78
SLC6A4	Serotonin transporter	6.537602002	7.677780705	TestisSeminiferousTubule	4.45	-0.30	-1.94	-2.28
SLC6A4	Serotonin transporter	6.537602002	7.677780705	Thalamus	5.00	-0.21	-1.35	-1.59
SLC6A4	Serotonin transporter	6.537602002	7.677780705	Thymus	3.85	-0.40	-2.59	-3.04
SLC6A4	Serotonin transporter	6.537602002	7.677780705	Thyroid	6.00	-0.04	-0.28	-0.33
SLC6A4	Serotonin transporter	6.537602002	7.677780705	Tongue	4.85	-0.23	-1.51	-1.78
SLC6A4	Serotonin transporter	6.537602002	7.677780705	Tonsil	4.80	-0.24	-1.57	-1.84
SLC6A4	Serotonin transporter	6.537602002	7.677780705	Trachea	4.15	-0.35	-2.27	-2.66
SLC6A4	Serotonin transporter	6.537602002	7.677780705	TrigeminalGanglion	5.40	-0.14	-0.92	-1.08
SLC6A4	Serotonin transporter	6.537602002	7.677780705	Uterus	3.95	-0.38	-2.48	-2.91
SLC6A4	Serotonin transporter	6.537602002	7.677780705	UterusCorpus	4.75	-0.25	-1.62	-1.90
SLC6A4	Serotonin transporter	6.537602002	7.677780705	WholeBlood	5.30	-0.16	-1.03	-1.21
SLC6A4	Serotonin transporter	6.537602002	7.677780705	Wholebrain	4.25	-0.33	-2.16	-2.53
TACR2	Neurokinin 2 receptor	n/a	5.042632192	Adipocyte	5.05	-0.32	n/a	-1.62
TACR2	Neurokinin 2 receptor	n/a	5.042632192	AdrenalCortex	5.60	0.06	n/a	0.28
TACR2	Neurokinin 2 receptor	n/a	5.042632192	Adrenalgland	7.40	1.29	n/a	6.51
TACR2	Neurokinin 2 receptor	n/a	5.042632192	Amygdala	5.20	-0.22	n/a	-1.10
TACR2	Neurokinin 2 receptor	n/a	5.042632192	Appendix	5.75	0.16	n/a	0.80
TACR2	Neurokinin 2 receptor	n/a	5.042632192	AtrioventricularNode	6.25	0.50	n/a	2.53
TACR2	Neurokinin 2 receptor	n/a	5.042632192	BDCA4+_DentriticCells	5.40	-0.08	n/a	-0.41
TACR2	Neurokinin 2 receptor	n/a	5.042632192	Bonemarrow	5.60	0.06	n/a	0.28
TACR2	Neurokinin 2 receptor	n/a	5.042632192	BronchialEpithelialCells	4.90	-0.42	n/a	-2.14
TACR2	Neurokinin 2 receptor	n/a	5.042632192	CardiacMyocytes	6.55	0.71	n/a	3.57
TACR2	Neurokinin 2 receptor	n/a	5.042632192	Caudatenucleus	4.55	-0.67	n/a	-3.35
TACR2	Neurokinin 2 receptor	n/a	5.042632192	CD105+_Endothelial	5.05	-0.32	n/a	-1.62
TACR2	Neurokinin 2 receptor	n/a	5.042632192	CD14+_Monocytes	5.25	-0.18	n/a	-0.93
TACR2	Neurokinin 2 receptor	n/a	5.042632192	CD19+_BCells(neg._sel.)	5.30	-0.15	n/a	-0.76
TACR2	Neurokinin 2 receptor	n/a	5.042632192	CD33+_Myeloid	6.35	0.57	n/a	2.88
TACR2	Neurokinin 2 receptor	n/a	5.042632192	CD34+	6.25	0.50	n/a	2.53
TACR2	Neurokinin 2 receptor	n/a	5.042632192	CD4+_Tcells	5.40	-0.08	n/a	-0.41
TACR2	Neurokinin 2 receptor	n/a	5.042632192	CD56+_NKCells	5.75	0.16	n/a	0.80
TACR2	Neurokinin 2 receptor	n/a	5.042632192	CD71+_EarlyErythroid	4.70	-0.56	n/a	-2.84
TACR2	Neurokinin 2 receptor	n/a	5.042632192	CD8+_Tcells	4.70	-0.56	n/a	-2.84
TACR2	Neurokinin 2 receptor	n/a	5.042632192	Cerebellum	6.95	0.98	n/a	4.95
TACR2	Neurokinin 2 receptor	n/a	5.042632192	CerebellumPeduncles	5.85	0.23	n/a	1.15
TACR2	Neurokinin 2 receptor	n/a	5.042632192	CiliaryGanglion	4.05	-1.01	n/a	-5.09
TACR2	Neurokinin 2 receptor	n/a	5.042632192	CingulateCortex	14.05	5.86	n/a	29.54
TACR2	Neurokinin 2 receptor	n/a	5.042632192	Colon	7.40	1.29	n/a	6.51
TACR2	Neurokinin 2 receptor	n/a	5.042632192	DorsalRootGanglion	4.05	-1.01	n/a	-5.09
TACR2	Neurokinin 2 receptor	n/a	5.042632192	Fetalbrain	5.10	-0.29	n/a	-1.45

S2 Table. Target-tissue analysis for clozapine and chlorpromazine

Gene Symbol	Gene Name	Affinity (-log10(Ki))		Tissue	Expression		Combined Score (-log10(Ki)*Z)	
		clozapine	chlorpromazine		Raw-numbers	Z-score	clozapine	chlorpromazine
TACR2	Neurokinin 2 receptor	n/a	5.042632192	Fetalliver	4.45	-0.73	n/a	-3.70
TACR2	Neurokinin 2 receptor	n/a	5.042632192	Fetallung	4.00	-1.04	n/a	-5.26
TACR2	Neurokinin 2 receptor	n/a	5.042632192	FetalThyroid	5.00	-0.36	n/a	-1.80
TACR2	Neurokinin 2 receptor	n/a	5.042632192	GlobusPallidus	3.90	-1.11	n/a	-5.61
TACR2	Neurokinin 2 receptor	n/a	5.042632192	Heart	7.25	1.19	n/a	5.99
TACR2	Neurokinin 2 receptor	n/a	5.042632192	Hypothalamus	5.50	-0.01	n/a	-0.07
TACR2	Neurokinin 2 receptor	n/a	5.042632192	Kidney	10.80	3.63	n/a	18.29
TACR2	Neurokinin 2 receptor	n/a	5.042632192	Liver	6.95	0.98	n/a	4.95
TACR2	Neurokinin 2 receptor	n/a	5.042632192	Lung	5.60	0.06	n/a	0.28
TACR2	Neurokinin 2 receptor	n/a	5.042632192	Lymphnode	4.35	-0.80	n/a	-4.05
TACR2	Neurokinin 2 receptor	n/a	5.042632192	MedullaOblongata	4.80	-0.49	n/a	-2.49
TACR2	Neurokinin 2 receptor	n/a	5.042632192	OccipitalLobe	4.75	-0.53	n/a	-2.66
TACR2	Neurokinin 2 receptor	n/a	5.042632192	OlfactoryBulb	4.45	-0.73	n/a	-3.70
TACR2	Neurokinin 2 receptor	n/a	5.042632192	Ovary	3.55	-1.35	n/a	-6.82
TACR2	Neurokinin 2 receptor	n/a	5.042632192	Pancreas	4.15	-0.94	n/a	-4.74
TACR2	Neurokinin 2 receptor	n/a	5.042632192	PancreaticIslet	5.45	-0.05	n/a	-0.24
TACR2	Neurokinin 2 receptor	n/a	5.042632192	ParietalLobe	5.90	0.26	n/a	1.32
TACR2	Neurokinin 2 receptor	n/a	5.042632192	Pineal_day	6.20	0.47	n/a	2.36
TACR2	Neurokinin 2 receptor	n/a	5.042632192	Pineal_night	6.08	0.39	n/a	1.94
TACR2	Neurokinin 2 receptor	n/a	5.042632192	Pituitary	6.30	0.54	n/a	2.70
TACR2	Neurokinin 2 receptor	n/a	5.042632192	Placenta	5.30	-0.15	n/a	-0.76
TACR2	Neurokinin 2 receptor	n/a	5.042632192	Pons	5.65	0.09	n/a	0.45
TACR2	Neurokinin 2 receptor	n/a	5.042632192	PrefrontalCortex	6.30	0.54	n/a	2.70
TACR2	Neurokinin 2 receptor	n/a	5.042632192	Prostate	5.75	0.16	n/a	0.80
TACR2	Neurokinin 2 receptor	n/a	5.042632192	Retina	6.03	0.35	n/a	1.75
TACR2	Neurokinin 2 receptor	n/a	5.042632192	Salivarygland	6.15	0.43	n/a	2.19
TACR2	Neurokinin 2 receptor	n/a	5.042632192	SkeletalMuscle	6.20	0.47	n/a	2.36
TACR2	Neurokinin 2 receptor	n/a	5.042632192	Skin	4.50	-0.70	n/a	-3.53
TACR2	Neurokinin 2 receptor	n/a	5.042632192	Small_intestine	4.90	-0.42	n/a	-2.14
TACR2	Neurokinin 2 receptor	n/a	5.042632192	SmoothMuscle	5.60	0.06	n/a	0.28
TACR2	Neurokinin 2 receptor	n/a	5.042632192	Spinalcord	5.45	-0.05	n/a	-0.24
TACR2	Neurokinin 2 receptor	n/a	5.042632192	SubthalamicNucleus	5.95	0.30	n/a	1.49
TACR2	Neurokinin 2 receptor	n/a	5.042632192	SuperiorCervicalGanglion	5.95	0.30	n/a	1.49
TACR2	Neurokinin 2 receptor	n/a	5.042632192	TemporalLobe	4.85	-0.46	n/a	-2.32
TACR2	Neurokinin 2 receptor	n/a	5.042632192	Testis	4.35	-0.80	n/a	-4.05
TACR2	Neurokinin 2 receptor	n/a	5.042632192	TestisGermCell	4.10	-0.97	n/a	-4.91
TACR2	Neurokinin 2 receptor	n/a	5.042632192	TestisIntersitial	4.25	-0.87	n/a	-4.39
TACR2	Neurokinin 2 receptor	n/a	5.042632192	TestisLeydigCell	5.20	-0.22	n/a	-1.10
TACR2	Neurokinin 2 receptor	n/a	5.042632192	TestisSeminiferousTubule	4.40	-0.77	n/a	-3.87
TACR2	Neurokinin 2 receptor	n/a	5.042632192	Thalamus	5.45	-0.05	n/a	-0.24
TACR2	Neurokinin 2 receptor	n/a	5.042632192	Thymus	3.95	-1.08	n/a	-5.43
TACR2	Neurokinin 2 receptor	n/a	5.042632192	Thyroid	6.10	0.40	n/a	2.01
TACR2	Neurokinin 2 receptor	n/a	5.042632192	Tongue	5.40	-0.08	n/a	-0.41
TACR2	Neurokinin 2 receptor	n/a	5.042632192	Tonsil	5.00	-0.36	n/a	-1.80
TACR2	Neurokinin 2 receptor	n/a	5.042632192	Trachea	4.25	-0.87	n/a	-4.39
TACR2	Neurokinin 2 receptor	n/a	5.042632192	TrigeminalGanglion	5.50	-0.01	n/a	-0.07
TACR2	Neurokinin 2 receptor	n/a	5.042632192	Uterus	6.25	0.50	n/a	2.53
TACR2	Neurokinin 2 receptor	n/a	5.042632192	UterusCorpus	4.80	-0.49	n/a	-2.49
TACR2	Neurokinin 2 receptor	n/a	5.042632192	WholeBlood	5.40	-0.08	n/a	-0.41
TACR2	Neurokinin 2 receptor	n/a	5.042632192	Wholebrain	4.10	-0.97	n/a	-4.91
TRPV1	Vanilloid receptor	n/a	6.283996656	Adipocyte	5.23	-0.06	n/a	-0.36
TRPV1	Vanilloid receptor	n/a	6.283996656	AdrenalCortex	5.68	0.51	n/a	3.20
TRPV1	Vanilloid receptor	n/a	6.283996656	Adrenalgland	4.48	-1.00	n/a	-6.29

S2 Table. Target-tissue analysis for clozapine and chlorpromazine

Gene Symbol	Gene Name	Affinity (-log10(Ki))		Tissue	Expression		Combined Score (-log10(Ki)*Z)	
		clozapine	chlorpromazine		Raw-numbers	Z-score	clozapine	chlorpromazine
TRPV1	Vanilloid receptor	n/a	6.283996656	Amygdala	5.45	0.23	n/a	1.42
TRPV1	Vanilloid receptor	n/a	6.283996656	Appendix	5.43	0.19	n/a	1.22
TRPV1	Vanilloid receptor	n/a	6.283996656	AtrioventricularNode	4.20	-1.35	n/a	-8.47
TRPV1	Vanilloid receptor	n/a	6.283996656	BDCA4+_DentriticCells	5.60	0.41	n/a	2.61
TRPV1	Vanilloid receptor	n/a	6.283996656	Bonemarrow	5.33	0.07	n/a	0.43
TRPV1	Vanilloid receptor	n/a	6.283996656	BronchialEpithelialCells	5.18	-0.12	n/a	-0.75
TRPV1	Vanilloid receptor	n/a	6.283996656	CardiacMyocytes	6.83	1.96	n/a	12.30
TRPV1	Vanilloid receptor	n/a	6.283996656	Caudatenucleus	4.73	-0.69	n/a	-4.31
TRPV1	Vanilloid receptor	n/a	6.283996656	CD105+_Endothelial	5.33	0.07	n/a	0.43
TRPV1	Vanilloid receptor	n/a	6.283996656	CD14+_Monocytes	5.58	0.38	n/a	2.41
TRPV1	Vanilloid receptor	n/a	6.283996656	CD19+_BCells(neg._sel.)	5.55	0.35	n/a	2.21
TRPV1	Vanilloid receptor	n/a	6.283996656	CD33+_Myeloid	6.68	1.77	n/a	11.11
TRPV1	Vanilloid receptor	n/a	6.283996656	CD34+	6.53	1.58	n/a	9.92
TRPV1	Vanilloid receptor	n/a	6.283996656	CD4+_Tcells	5.60	0.41	n/a	2.61
TRPV1	Vanilloid receptor	n/a	6.283996656	CD56+_NKCells	5.90	0.79	n/a	4.98
TRPV1	Vanilloid receptor	n/a	6.283996656	CD71+_EarlyErythroid	4.93	-0.43	n/a	-2.73
TRPV1	Vanilloid receptor	n/a	6.283996656	CD8+_Tcells	4.98	-0.37	n/a	-2.34
TRPV1	Vanilloid receptor	n/a	6.283996656	Cerebellum	4.28	-1.25	n/a	-7.87
TRPV1	Vanilloid receptor	n/a	6.283996656	CerebellumPeduncles	6.05	0.98	n/a	6.17
TRPV1	Vanilloid receptor	n/a	6.283996656	CiliaryGanglion	5.50	0.29	n/a	1.82
TRPV1	Vanilloid receptor	n/a	6.283996656	CingulateCortex	5.40	0.16	n/a	1.02
TRPV1	Vanilloid receptor	n/a	6.283996656	Colon	5.28	0.01	n/a	0.04
TRPV1	Vanilloid receptor	n/a	6.283996656	DorsalRootGanglion	4.20	-1.35	n/a	-8.47
TRPV1	Vanilloid receptor	n/a	6.283996656	Fetalbrain	5.35	0.10	n/a	0.63
TRPV1	Vanilloid receptor	n/a	6.283996656	Fetalliver	4.58	-0.88	n/a	-5.50
TRPV1	Vanilloid receptor	n/a	6.283996656	Fetallung	4.35	-1.16	n/a	-7.28
TRPV1	Vanilloid receptor	n/a	6.283996656	FetalThyroid	5.13	-0.18	n/a	-1.15
TRPV1	Vanilloid receptor	n/a	6.283996656	GlobusPallidus	3.88	-1.76	n/a	-11.04
TRPV1	Vanilloid receptor	n/a	6.283996656	Heart	6.95	2.11	n/a	13.28
TRPV1	Vanilloid receptor	n/a	6.283996656	Hypothalamus	5.75	0.60	n/a	3.79
TRPV1	Vanilloid receptor	n/a	6.283996656	Kidney	4.38	-1.13	n/a	-7.08
TRPV1	Vanilloid receptor	n/a	6.283996656	Liver	7.08	2.27	n/a	14.27
TRPV1	Vanilloid receptor	n/a	6.283996656	Lung	5.70	0.54	n/a	3.40
TRPV1	Vanilloid receptor	n/a	6.283996656	Lymphnode	4.45	-1.03	n/a	-6.49
TRPV1	Vanilloid receptor	n/a	6.283996656	MedullaOblongata	4.85	-0.53	n/a	-3.33
TRPV1	Vanilloid receptor	n/a	6.283996656	OccipitalLobe	4.70	-0.72	n/a	-4.51
TRPV1	Vanilloid receptor	n/a	6.283996656	OlfactoryBulb	4.20	-1.35	n/a	-8.47
TRPV1	Vanilloid receptor	n/a	6.283996656	Ovary	3.63	-2.07	n/a	-13.01
TRPV1	Vanilloid receptor	n/a	6.283996656	Pancreas	4.25	-1.28	n/a	-8.07
TRPV1	Vanilloid receptor	n/a	6.283996656	PancreaticIslet	5.70	0.54	n/a	3.40
TRPV1	Vanilloid receptor	n/a	6.283996656	ParietalLobe	5.65	0.48	n/a	3.00
TRPV1	Vanilloid receptor	n/a	6.283996656	Pineal_day	6.53	1.59	n/a	9.96
TRPV1	Vanilloid receptor	n/a	6.283996656	Pineal_night	6.27	1.26	n/a	7.91
TRPV1	Vanilloid receptor	n/a	6.283996656	Pituitary	6.13	1.08	n/a	6.76
TRPV1	Vanilloid receptor	n/a	6.283996656	Placenta	5.35	0.10	n/a	0.63
TRPV1	Vanilloid receptor	n/a	6.283996656	Pons	5.03	-0.31	n/a	-1.94
TRPV1	Vanilloid receptor	n/a	6.283996656	PrefrontalCortex	6.23	1.20	n/a	7.55
TRPV1	Vanilloid receptor	n/a	6.283996656	Prostate	6.15	1.11	n/a	6.96
TRPV1	Vanilloid receptor	n/a	6.283996656	Retina	6.35	1.36	n/a	8.54
TRPV1	Vanilloid receptor	n/a	6.283996656	Salivarygland	4.30	-1.22	n/a	-7.68
TRPV1	Vanilloid receptor	n/a	6.283996656	SkeletalMuscle	6.35	1.36	n/a	8.54
TRPV1	Vanilloid receptor	n/a	6.283996656	Skin	5.28	0.01	n/a	0.04
TRPV1	Vanilloid receptor	n/a	6.283996656	Small_intestine	5.08	-0.25	n/a	-1.55

S2 Table. Target-tissue analysis for clozapine and chlorpromazine

Gene Symbol	Gene Name	Affinity (-log10(Ki))		Tissue	Expression		Combined Score (-log10(Ki)*Z)	
		clozapine	chlorpromazine		Raw-numbers	Z-score	clozapine	chlorpromazine
TRPV1	Vanilloid receptor	n/a	6.283996656	SmoothMuscle	5.98	0.89	n/a	5.57
TRPV1	Vanilloid receptor	n/a	6.283996656	Spinalcord	5.68	0.51	n/a	3.20
TRPV1	Vanilloid receptor	n/a	6.283996656	SubthalamicNucleus	4.83	-0.56	n/a	-3.52
TRPV1	Vanilloid receptor	n/a	6.283996656	SuperiorCervicalGanglion	6.23	1.20	n/a	7.55
TRPV1	Vanilloid receptor	n/a	6.283996656	TemporalLobe	4.83	-0.56	n/a	-3.52
TRPV1	Vanilloid receptor	n/a	6.283996656	Testis	4.53	-0.94	n/a	-5.90
TRPV1	Vanilloid receptor	n/a	6.283996656	TestisGermCell	4.35	-1.16	n/a	-7.28
TRPV1	Vanilloid receptor	n/a	6.283996656	TestisIntersitial	4.45	-1.03	n/a	-6.49
TRPV1	Vanilloid receptor	n/a	6.283996656	TestisLeydigCell	5.23	-0.06	n/a	-0.36
TRPV1	Vanilloid receptor	n/a	6.283996656	TestisSeminiferousTubule	4.53	-0.94	n/a	-5.90
TRPV1	Vanilloid receptor	n/a	6.283996656	Thalamus	5.40	0.16	n/a	1.02
TRPV1	Vanilloid receptor	n/a	6.283996656	Thymus	4.15	-1.41	n/a	-8.86
TRPV1	Vanilloid receptor	n/a	6.283996656	Thyroid	6.53	1.58	n/a	9.92
TRPV1	Vanilloid receptor	n/a	6.283996656	Tongue	5.70	0.54	n/a	3.40
TRPV1	Vanilloid receptor	n/a	6.283996656	Tonsil	5.10	-0.21	n/a	-1.35
TRPV1	Vanilloid receptor	n/a	6.283996656	Trachea	4.43	-1.06	n/a	-6.69
TRPV1	Vanilloid receptor	n/a	6.283996656	TrigeminalGanglion	5.73	0.57	n/a	3.60
TRPV1	Vanilloid receptor	n/a	6.283996656	Uterus	4.28	-1.25	n/a	-7.87
TRPV1	Vanilloid receptor	n/a	6.283996656	UterusCorpus	4.75	-0.66	n/a	-4.12
TRPV1	Vanilloid receptor	n/a	6.283996656	WholeBlood	5.30	0.04	n/a	0.23
TRPV1	Vanilloid receptor	n/a	6.283996656	Wholebrain	4.45	-1.03	n/a	-6.49
